# Insecticidal and Repellent Properties of Rapid-Acting Fluorine-Containing Compounds against *Aedes aegypti* Mosquitoes

**DOI:** 10.1021/acsinfecdis.3c00161

**Published:** 2023-06-13

**Authors:** Xiaolong Zhu, Wilson Valbon, Mengdi Qiu, Chunhua T. Hu, Jingxiang Yang, Bryan Erriah, Milena Jankowska, Ke Dong, Michael D. Ward, Bart Kahr

**Affiliations:** ⊥ Department of Chemistry and Molecular Design Institute, New York University, 100 Washington Square East, New York, New York 10003 USA; ‡ Department of Biology, Duke University, 130 Science Drive, Durham, North Carolina 27708 USA; § Department of Animal Physiology and Neurobiology, Nicolaus Copernicus University, Lwowska 1 Street, Toruń 87-100, Poland

**Keywords:** contact insecticide, repellency, *Aedes aegypti*, fluorine, mosquito sensing, stereoselectivity, rapid knockdown

## Abstract

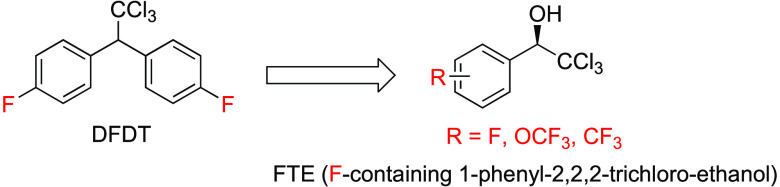

The development of safe and potent insecticides remains an integral part of a multifaceted strategy to effectively control human-disease-transmitting insect vectors. Incorporating fluorine can dramatically alter the physiochemical properties and bioavailability of insecticides. For example, 1,1,1-trichloro-2,2-bis(4-fluorophenyl)ethane (DFDT)—a difluoro congener of trichloro-2,2-bis(4-chlorophenyl)ethane (DDT)—was demonstrated previously to be 10-fold less toxic to mosquitoes than DDT in terms of LD_50_ values, but it exhibited a 4-fold faster knockdown. Described herein is the discovery of fluorine-containing 1-aryl-2,2,2-trichloro-ethan-1-ols (FTEs, for fluorophenyl-trichloromethyl-ethanols). FTEs, particularly per-fluorophenyl-trichloromethyl-ethanol (PFTE), exhibited rapid knockdown not only against *Drosophila melanogaster* but also against susceptible and resistant *Aedes aegypti* mosquitoes, major vectors of Dengue, Zika, yellow fever, and Chikungunya viruses. The *R* enantiomer of any chiral FTE, synthesized enantioselectively, exhibited faster knockdown than its corresponding *S* enantiomer. PFTE does not prolong the opening of mosquito sodium channels that are characteristic of the action of DDT and pyrethroid insecticides. In addition, pyrethroid/DDT-resistant *Ae. aegypti* strains having enhanced P450-mediated detoxification and/or carrying sodium channel mutations that confer knockdown resistance were not cross-resistant to PFTE. These results indicate a mechanism of PFTE insecticidal action distinct from that of pyrethroids or DDT. Furthermore, PFTE elicited spatial repellency at concentrations as low as 10 ppm in a hand-in-cage assay. PFTE and MFTE were found to possess low mammalian toxicity. These results suggest the substantial potential of FTEs as a new class of compounds for controlling insect vectors, including pyrethroid/DDT-resistant mosquitoes. Further investigations of FTE insecticidal and repellency mechanisms could provide important insights into how incorporation of fluorine influences the rapid lethality and mosquito sensing.

## Introduction

Following trends in medicinal chemistry,^1^ many new fluorine-containing insecticides^2^ and other pesticides^3−5^ have been introduced in recent decades, facilitated by developments in fluoro-organic chemistry and motivated by the need to mitigate metabolic resistance. Representative fluorine-containing insecticides across diverse structural classes include metofluthrin, bifenthrin, fipronil, flupyradifuron, and bistrifluoron, as well as the pro-insecticide chlorfenapyr. The introduction of fluorine atoms and trifluoromethyl groups can significantly improve insecticide bioavailability by enhancing lipophilicity, cellular membrane permeability, metabolic stability (e.g., against cytochrome P450 enzymatic oxidations), and binding affinity with minimal steric perturbation.^3,6−8^

Malariologists^9,10^ have emphasized the continuing need for new, inexpensive, for indoor residual spraying and insecticide-treated bed nets. Neonicotinoids, which were approved by the WHO in 2018 as a new class of insecticides for combatting pyrethroid resistance, quickly faltered as resistance developed, presumably a result of their heavy use in agriculture.^11^ There is an urgent need for new chemical agents that can mitigate vector-borne diseases and overcome resistant organisms.

Previously, we reinvestigated a difluoro congener of DDT, DFDT (1,1,1-trichloro-2,2-(bis(4,4′- fluorophenyl)ethane),^12^ and its chiral monofluoro analog, MFDT^13^ (1,1,1-trichloro-2,2-(4-chlorophenyl)-(4-fluorophenyl)ethane).^9^ DFDT acted more rapidly than DDT against *Drosophila* and *Anopheles quadrimaculatus*, a vector of malaria, as well as *Aedes aegypti*, a vector of Zika, Yellow fever, Dengue, and Chikungunya viruses. Despite the fact that DFDT’s neurophysiological efficacy *in vitro* is 10-fold smaller than that of DDT, as well as the observation that DDT and DFDT are cross-resistant, amorphous DFDT is approximately 4 times faster than amorphous DDT in knocking down *Aedes* and *Anopheles* mosquitoes.^14^ This difference may be attributed to the fluorine-enhanced lipophilicity^15^ and thus bioavailability of DFDT molecules traversing cuticle and cellular membranes.^6^

Major insecticides used for public health are crystalline, with contact action like that of DDT.^15^ The speed of action (knockdown time) of contact insecticides depends on the release of molecules from the solid surface of the insecticide, followed by the penetration of molecules into the insect cuticle.^16^ We have demonstrated that the speed of action of organochlorine,^17,18^ organofluorine,^9^ pyrethroid,^19^ and neonicotinoid^20^ insecticides can be increased in metastable solid-state forms of a given compound. Metastable solid forms of the same chemical compound have higher free energies, thereby surrendering toxicant molecules from their surfaces more readily to insect tarsi upon contact. The differential lethality of polymorphs demonstrates that the rate of uptake from the solid surface is consequential. For instance, a commercial formulation of deltamethrin dispersed on chalk that had been heated briefly to 120 °C killed 100% of various resistant mosquito strains from Burkina Faso, without exception, whereas the commercial material as obtained was ineffective.^21^ This increased activity was attributed to the heat-induced transformation of commercial deltamethrin crystals to another crystalline form with a higher bioavailability.^19^ The more active form persists for 13 months and counting.^21^ Crystalline polymorphs with faster uptake can remove resistant organisms from a population where slower uptake polymorphs are indifferent. For this reason we have focused on inexpensive compounds that work quickly, especially as the WHO is moving toward new antimalarial compounds with previously unexplored mechanisms of action.^22^

Having synthesized MFDT and evaluated its enantioselectivity in insect knockdown, we tested the synthetic intermediate 1-phenyl-2,2,2-trichloro-ethanol. Herein, we describe the synthesis and evaluation of a group of related fluorine-containing 1-phenyl-2,2,2-trichloro-ethanol compounds, including per-fluorophenyl-trichloromethyl-ethanol (PFTE), with a particular focus on their liquid, amorphous, and crystalline phases and the role of fluorine. We also examine the insecticidal and repellent actions of PFTE against *Ae. aegypti* mosquitoes. Our findings indicate that fluorine-containing 1-phenyl-2,2,2-trichloro-ethanols are promising for combatting vector-borne human diseases.

## Results and Discussion

### Fluorinated DFDT Congeners and 1-Phenyl-2,2,2-trichloro-ethanols

**Figure 1:**
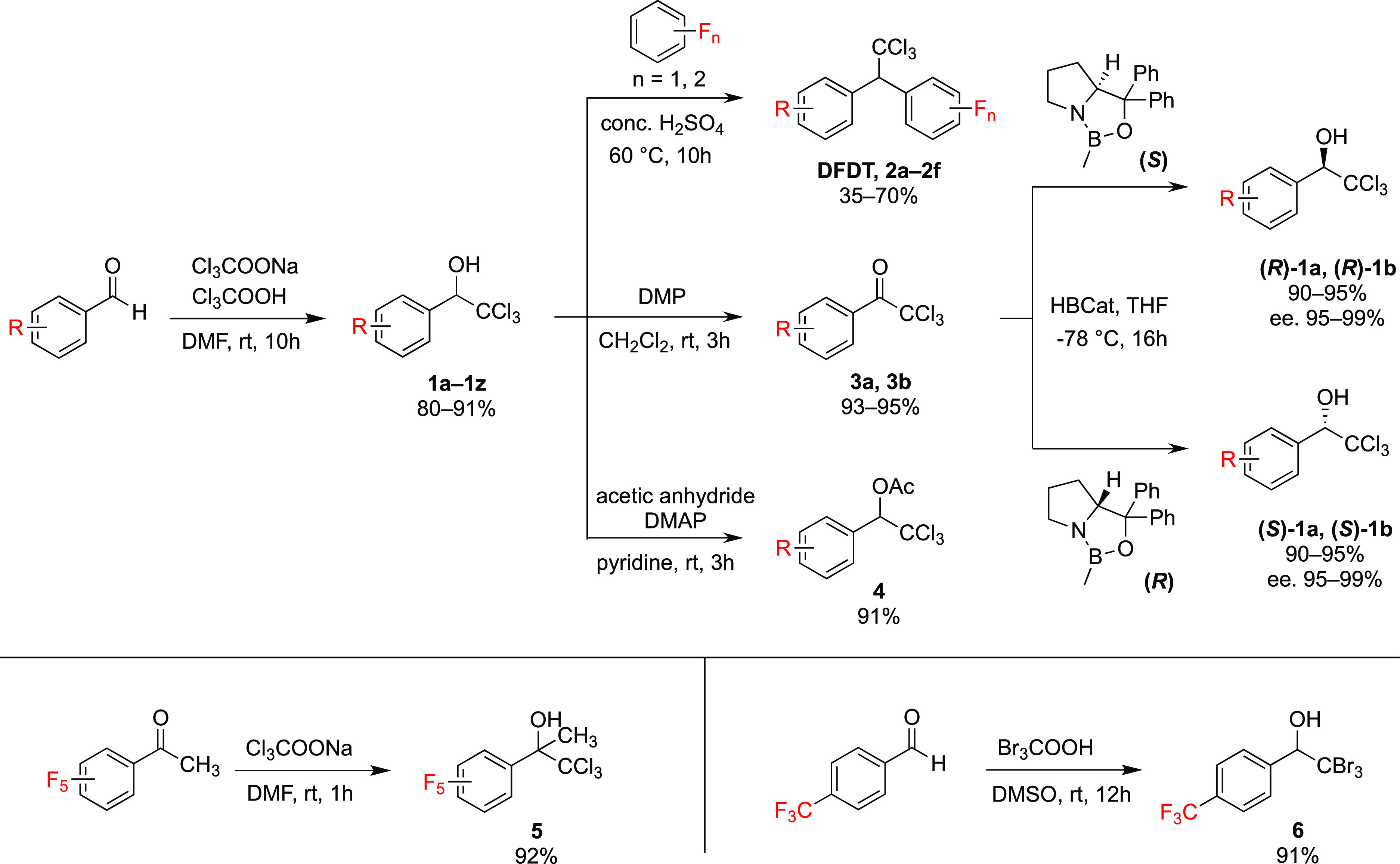
Synthetic routes of compounds with various chemical moieties.

**Figure 2:**
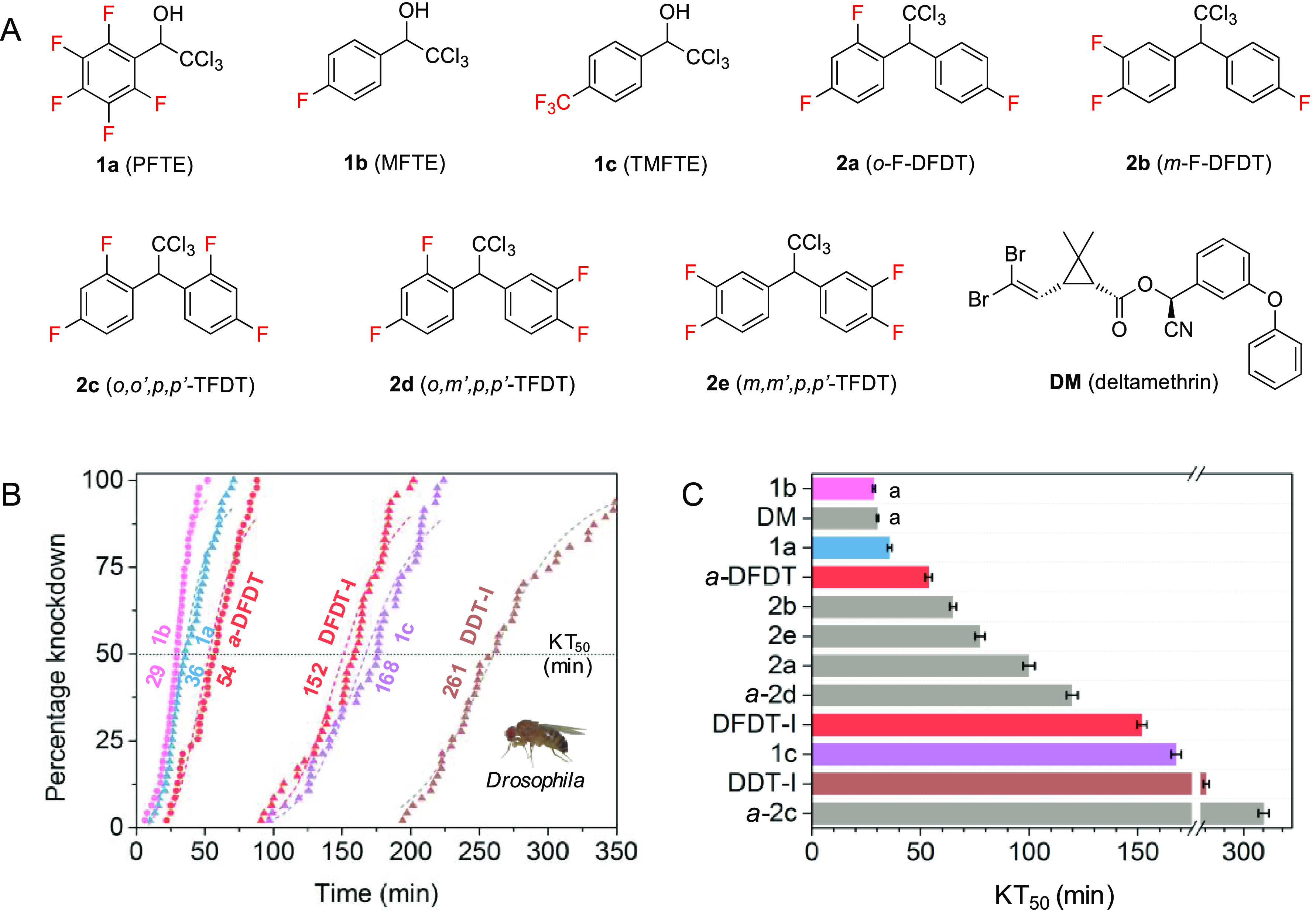
(A) Chemical structure of selected compounds. (B) Lethality of **1a** (crystals), **1b** (liquid), *a*-DFDT, DFDT Form I, **1c** (crystals), and DDT Form I against *D. melanogaster*. The median knockdown time (KT_50_) for each curve is denoted by its intersection with the horizontal KT_50_ marker. Inset: Photograph of a typical female *D. melanogaster*. (C) Comparison of the KT_50_ values. **2a**, **2b**, and **2e** were liquid, whereas **2c** and **2d** were amorphous. Error bars represent 95% confidence intervals (CI). Values with the same letter have overlapping 95% CIs, and differences are considered insignificant. Knockdown–time curves and logistic regression are available in the Supporting Information.

Given that DFDT only has fluorine on its *para* positions, the introduction of additional aromatic fluorine atoms may further improve its knockdown speed. On the other hand, prior studies have demonstrated that the insecticidal potency of DDT decreased substantially when the trichloromethyl group was replaced with a trifluoromethyl group,^23^ or when the α-hydrogen was substituted by fluorine.^24^ Based on these results, we synthesized fluorinated DFDT congeners **2a**–**2e** with aromatic fluorine substituents (Figures 1 and 2A, and Supporting Information).^25,26^ In addition, one synthetic intermediate, 1-(4-fluorophenyl)-2,2,2-trichloro-ethanol (**1b**; Figures 1 and 2A), was reported to have one- to two-fifths of DDT’s insecticidal activity against fruit flies (*Drosophila melanogaster*) in contact residual exposure bioassays.^27^ This active intermediate inspired the synthesis of compounds **1a** (PFTE) and **1c** with respective perfluoro- and trifluoromethyl-phenyl groups (Figures 1 and 2A, and Supporting Information).

**Figure 3:**
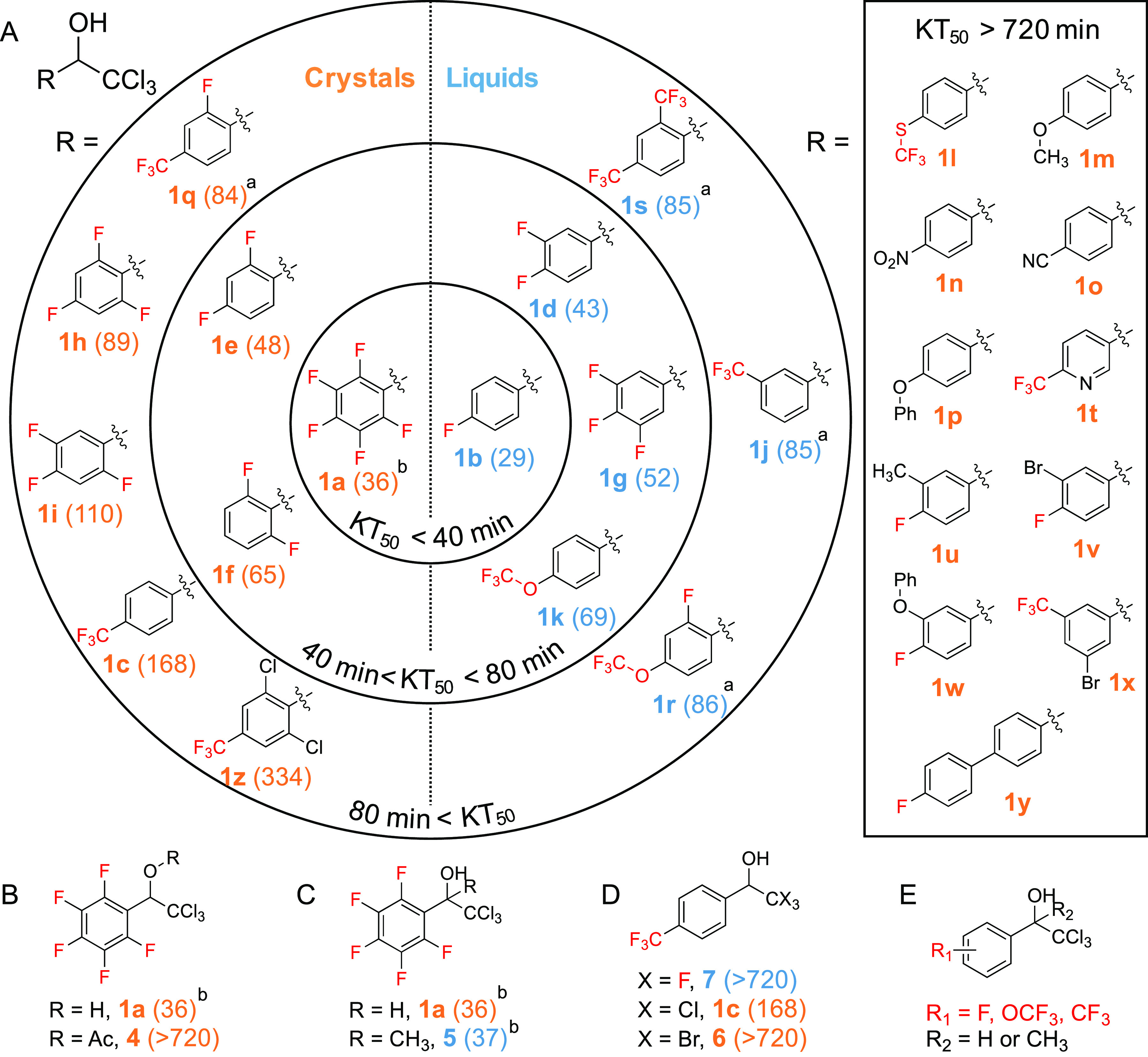
Structure–activity relationships. (A–D) Compounds with various chemical moieties. KT_50_ (min) for *D. melanogaster* are denoted in parentheses. Physical states are denoted by color: crystals, orange; liquid, blue. Values with the same letter have overlapping 95% confidence intervals, and differences are considered insignificant. Knockdown–time curves and logistic regression are available in the Supporting Information. (E) Chemical structure of compounds with good knockdown properties.

**Figure 4:**
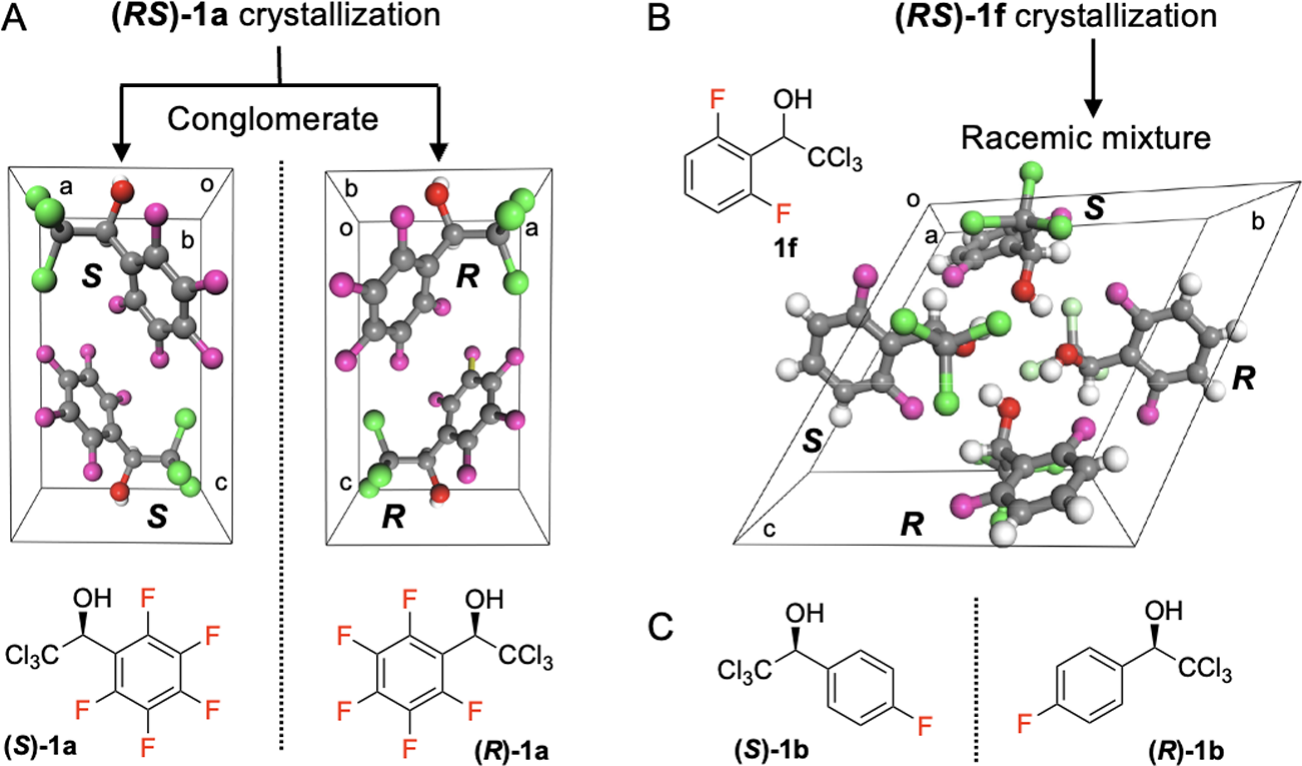
(A) **1a** crystallized into conglomerates with a chiral *P*2_1_ space group. The enantiomers could not be separated from the conglomerates and were instead prepared by the asymmetric synthesis shown in Figure 2. Atom colors: hydrogen, white; carbon, gray; fluorine, magenta; chlorine, green. (B) Fluorinated compounds shown in Figure 3A, except **1a**. **1f** crystallized as a racemate with a *P*1 space group (shown as an example). Other crystal structures are available in the Supporting Information. The apparently skewed unit cell is a consequence of a perspective view along a general direction. (C) Similar to **1a**, enantiomers of **1b** prepared by the asymmetric synthesis shown in Figure 2.

Figure 3 compares the median knockdown time (KT_50_) values for the compounds discussed throughout this paper. Because the knockdown speed of insecticides is highly dependent on their condensed phases,^9,30−33^ we prepared all compounds at room temperature to best reflect their efficacy in the field. Compounds **1a** and **1c** were always monomorphic from the evaporation of various solvents or from the melt (Figure 4A, Figures S1 and S2, and Table S1). **1b**, **2a**, **2b**, and **2e** were liquid at room temperature. Although **2c** and **2d** could grow into crystals at room temperature (Figures S1 and S2, and Table S1), their amorphous states, which were prepared by supercooling melts or fine mist spraying of solutions, persisted for at least 20 days at room temperature and were used for lethality measurements. Each crystalline form was ground into particles with sizes (50 μm) comparable to those of amorphous or liquid particles prepared by fine mist spraying. Female *Drosophila melanogaster*, an accepted proxy for evaluating insecticide potency against mosquitoes,^28^ were placed in Petri dishes and exposed to 2.0 mg (10.6 μg/cm^2^) of all compounds in Figure 2A in their amorphous, crystalline, or liquid forms. The experimental procedure was similar to that previously reported by our laboratory.^9,32^ The DDT crystalline polymorph designated Form I,^30^ the DFDT crystalline polymorph also designated Form I, amorphous DFDT (*a*-DFDT),^9^ and deltamethrin (DM, a leading pyrethroid^29^) likewise Form I^32^ were used for comparison (*a*-DFDT and DM were known for their rapid knockdown properties). Knockdown times were analyzed by measuring the motion of flies recorded with a video camera. The KT_50_ values, here the median knockdown times for a fly population, were calculated by performing logistic regression analysis of knockdown–time curves (Table S2).^30^ The KT_50_ values decreased in the order DDT-I (261 min) > **1c** (168 min) > DFDT-I (152 min) > *a*-DFDT (54 min) > **1a** (36 min) > DM-I (30 min) ≈ **1b** (29 min) (Figure 2B,C). Liquid **1b** was comparable to DM Form I in lethality, although the uptake of molecules from liquid would be expected to be more rapid than that from crystals.^31^ Notably, crystalline **1a** and **1c** both exhibited rapid knockdown, and the former was even faster in action than amorphous DFDT. In contrast, **2a**–**2e**, whether in their amorphous or liquid forms, were all less active than *a*-DFDT (Figure 2C and Figure S3). These results suggested that the fluorination of 1-phenyl-2,2,2-trichloro-ethanol is a viable strategy for the discovery of rapid-acting agents.

### Structure–Activity Relationships

Structure–activity relationship (SAR) studies were performed on **1a**–**1c** to determine the key functionalities or moieties that may influence the insecticidal activity of 1-phenyl-2,2,2-trichloro-ethanol. Compounds **1d**–**1z**, **4**–**6** with various chemical moieties were synthesized (Figure 1 and Supporting Information), and their individual knockdown times against female *Drosophila* were evaluated at 10.6 μg/cm^2^ (Figure 3, Figures S4 and S5, and Table S2). Female *Drosophila* in control groups typically began to become immobilized after 720 min; thus, compounds with KT_50_ values >720 min were considered inactive.

SAR studies revealed that aromatic substituents significantly affected lethality (Figure 3A). 1-Phenyl-2,2,2-trichloro-ethanols that contain aromatic substituents of F (**1a**, **1b**, **1d**–**1i**), OCF_3_ (**1k**), CF_3_ (**1c**, **1j**, **1s**), and a mixture of two fluorine functionalities (**1q**, **1r**) exhibited rapid knockdown. In general, the ability of aromatic substituents to improve knockdown decreased in the order F > OCF_3_ > CF_3_. Aromatic fluorine substituents substantially influenced the physiochemical properties of compounds as well. Most compounds with *ortho* or *para* substituents were crystalline at room temperature, and their crystals were each prepared in their thermodynamically stable forms for lethality measurements (Figures S1 and S2, and Table S1). In contrast, *meta* substituents or the addition of the flexible OCF_3_ substituent decreased the melting points of some compounds to the extent that they were liquids at room temperature. Compounds **1a** and **1b** were respectively the fastest-acting crystalline and liquid compounds. Additionally, aromatic substituents with various steric and electronic effects were compared. Compounds that contain common electron-donating (**1m**, **1p**) or -withdrawing (**1n**, **1o**) groups were inactive. The switch from an OCF_3_ (**1k**) group to an SCF_3_ (**1l**) group, or from phenyl (**1b**, **1c**) to other fluorinated aromatic systems (**1t**, **1y**), led to a loss of activity. Moreover, the introduction of Cl (**1z**), Br (**1v**, **1x**), CH_3_ (**1u**), or OPh (**1w**) substituent to fluoro- or trifluoromethylphenyl groups resulted in a significant lowering of activity.

Following the SAR studies for aromatic substituents, we investigated the effects of three moieties that were attached to the central carbon atom. Acetoxylation led to a loss of activity, corroborating the importance of free alcohol in conferring lethality (Figure 3B). In contrast, the switch from H (**1a**) to CH_3_ (**5**) substituent had little effect on knockdown speed (Figure 3C). A −CH_3_ substituent, however, prevented the crystallization of compound **5** at room temperature. The impact of halogenated methyl groups was also probed by changing the halogen (Figure 3D). CCl_3_ (**1c**) was superior to both CF_3_ (**7**) and CBr_3_ (**6**) substituents, presumably due to steric effects. In conclusion, the SAR studies showed that 1-aryl-2,2,2-trichloro-ethanols and -propanols containing one or more aromatic substituents of F, OCF_3_, or CF_3_ exhibited remarkable knockdown properties (Figure 3E).

### Enantioselective Toxicities against Flies

The stereoisomers of a given insecticide may have different activities due to their unique interactions with the metabolic enzymes or the target site.^40^ For example, our previous studies demonstrated that the monofluoro congener of DDT—named MFDT—exhibited enantioselective lethality against *Drosophila*.^9^ Moreover, different insecticide stereoisomers may have different environmental impacts.^32^ Thus, single or enriched stereoisomer formulations of insecticides are preferable, because they can have both increased potency and reduced environmental impact.^33^ We explored the difference in the activity of enantiomers of **1a** and **1b**, because their degrees of fluorination are on opposite ends of the spectrum.

Interestingly, racemic **1a** always crystallized as conglomerates^34^ (a mixture of homochiral enantiomer crystals) in the *P*2_1_ space group from solution or from melts (Figure 4A). In contrast, other racemates displayed in Figure 3 all crystallized as racemates^47^ (equimolar amounts of enantiomers in the crystal lattices; Figure 4B and Figure S2). Since the crystals in conglomerates of **1a** were tiny needles (<100 μm), the separation of enantiomer crystals based on crystal morphology (a Pasteur-like resolution) was not feasible. Instead, we prepared the enantiomers via asymmetric synthesis. Racemates were oxidized to yield ketones,^35^ which were reduced by catecholborane in the presence of chiral oxazaborolidine catalysts^36^ to yield **1a** and **1b** enantiomers, each having 99% and 95% enantiomeric excess (Figure 1, and Supporting Information). The absolute configuration of the **1a** enantiomers was assigned using single-crystal X-ray analysis (Figure 4A, and Supporting Information).

**Figure 5:**
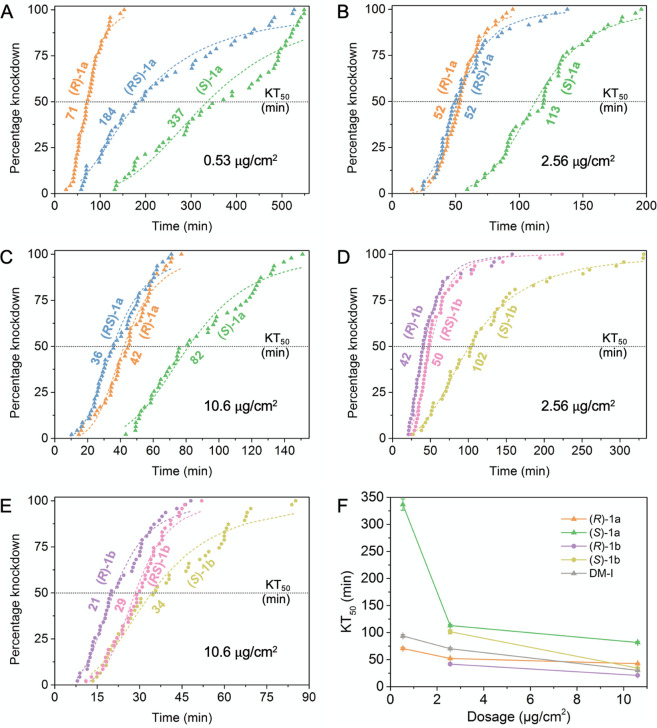
(A–E) Knockdown of **1a** enantiomers (crystals) and **1b** enantiomers (liquid) against *D. melanogaster* at various dosages. At 0.53 μg/cm^2^, both **1b** enantiomers had KT_50_ values >720 min. The median knockdown time for each curve is denoted by its intersection with the horizontal KT_50_ marker. (F) KT_50_ values versus dosage. Error bars represent 95% confidence intervals (CI).

Female *Drosophila* were exposed to racemates and enantiomers of both crystalline **1a** and liquid **1b** at various dosages. The *R* forms of **1a** and **1b** were always faster-acting than their respective *S* forms (Figure 5A–E and Table S2), suggesting enantioselectivity in insect uptake, metabolism, or neurotoxicity. (*R*)-**1a** was even faster-acting than DM Form I at very low dosages (Figure 5F and Figure S6). In contrast, both enantiomers of **1b** were less effective (KT_50_ > 720 min) at 0.53 μg/cm^2^, requiring higher lethal doses than of **1a**. Therefore, the rapid knockdown speeds of **1b** and other figures shown for Figure 3 are presumably dominated by the kinetics of uptake of molecules.

### Toxicity of PFTE against *Aedes aegypti* Mosquitoes

To determine whether PFTE induces rapid knockdown of mosquitoes, we conducted a contact-based bioassay as described above for flies. Adult females of an insecticide-susceptible wild-type *Ae. aegypti* strain, Rockefeller, were all knocked down in less than 5 min upon contact exposure to PFTE at the concentration of 12.5 μg/cm^2^. Furthermore, we observed 77% mortality after Rockefeller mosquitoes were exposed to vapor emitted from PFTE of 12.5 μg/cm^2^ for 24 h (Figure S1).

**Figure 6:**
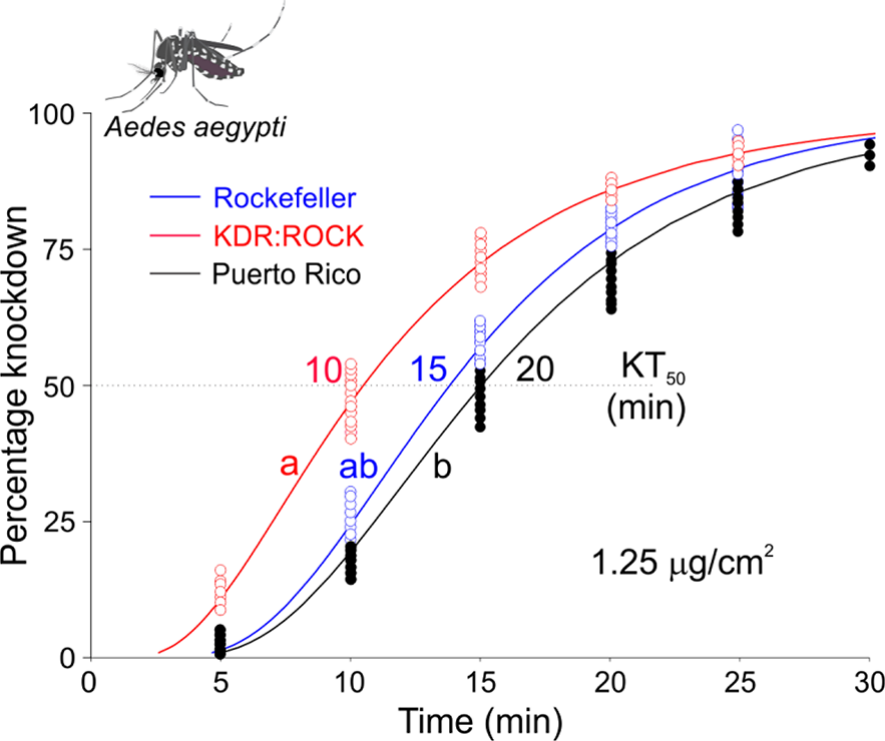
Knockdown of *Ae. aegypti* mosquitoes by crystalline PFTE. At the dosage of 1.25 μg/cm^2^, PFTE had KT_50_ values of 15, 10, and 20 min for Rockefeller, KDR:ROCK and Puerto Rico mosquitoes, respectively. The median knockdown time for each curve is denoted by its intersection with the horizontal KT_50_ dotted line. Curves with the same letter do not differ according to Log-Rank test, and dots denote the value of each biological replicate (mosquito). Rockefeller vs KDR:ROCK: Log Rank: χ^2^ = 3.11, *df* = 1, *P* = 0.08; Rockefeller vs Puerto Rico: Log Rank: χ^2^ = 3.22, *df* = 1, *P* = 0.073. KDR:ROCK vs Puerto Rico: χ^2^ = 13.13, *df* = 1, *P* < 0.001.

Given that PFTE induced very rapid knockdown at 12.5 μg/cm^2^, we also conducted the contact bioassay using 1.25 μg/cm^2^ and recorded the percentage of knockdown over the course of 1 h (Figure 6). Next, we examined two pyrethroid/DDT-resistant strains, KDR:ROCK^37^ and Puerto Rico,^38^ which carry different sodium channel mutations that confer knockdown resistance (kdr) to DDT and pyrethroids.^37,39^ The Puerto Rico strain also possesses an enhanced P450-mediated metabolic detoxification mechanism of resistance.^38^ As shown in Figure 6, the KT_50_ values of PFTE against these pyrethroid/DDT-resistant mosquitoes were not significantly different from those against the susceptible mosquitoes, although KDR:ROCK mosquitoes were more sensitive to PFTE than Puerto Rico mosquitoes. These results show that, unlike in the case of DFDT,^28^ there is no cross-resistance between PFTE, DDT, and pyrethroids. Further, our functional examination of mosquito sodium channels expressed in *Xenopus* oocytes indicates that, unlike pyrethroids and DDT, PFTE does not alter the gating of mosquito sodium channels (Figure S2).

These results indicate that PFTE may have unique target sites in the nervous system. An observation that may be related is that the miticide dicofol (2,2,2-trichloro-1,1-bis(4-chlorophenyl)ethanol), in which the α-carbon of DDT is hydroxylated, has a different mechanism of action than that of DDT itself.^40^ It is indifferent to houseflies with DDT-related kdr, and it elicits different symptoms of poisoning in susceptible flies, e.g., the absence of tremors. Subsequent research indicated that it may inhibit the octopamine-stimulated adenylate cyclase enzyme.^41^

Future studies using various nerve preparations are needed to elucidate the mechanism of PFTE action. Its perfluorination may not be subjected to detoxification by cytochrome P450 enzymes, but other metabolic detoxification pathways, such as glucuronidation, are still possible. The remarkably rapid knockdown by PFTE might be primarily attributed to the fact that fluorine significantly increases the lipophilicity, and thus bioavailability, of PFTE molecules, which are expected to rapidly traverse insect cuticle and cellular membranes.

### Repellency of PFTE against *Aedes aegypti* Mosquitoes

**Figure 7:**
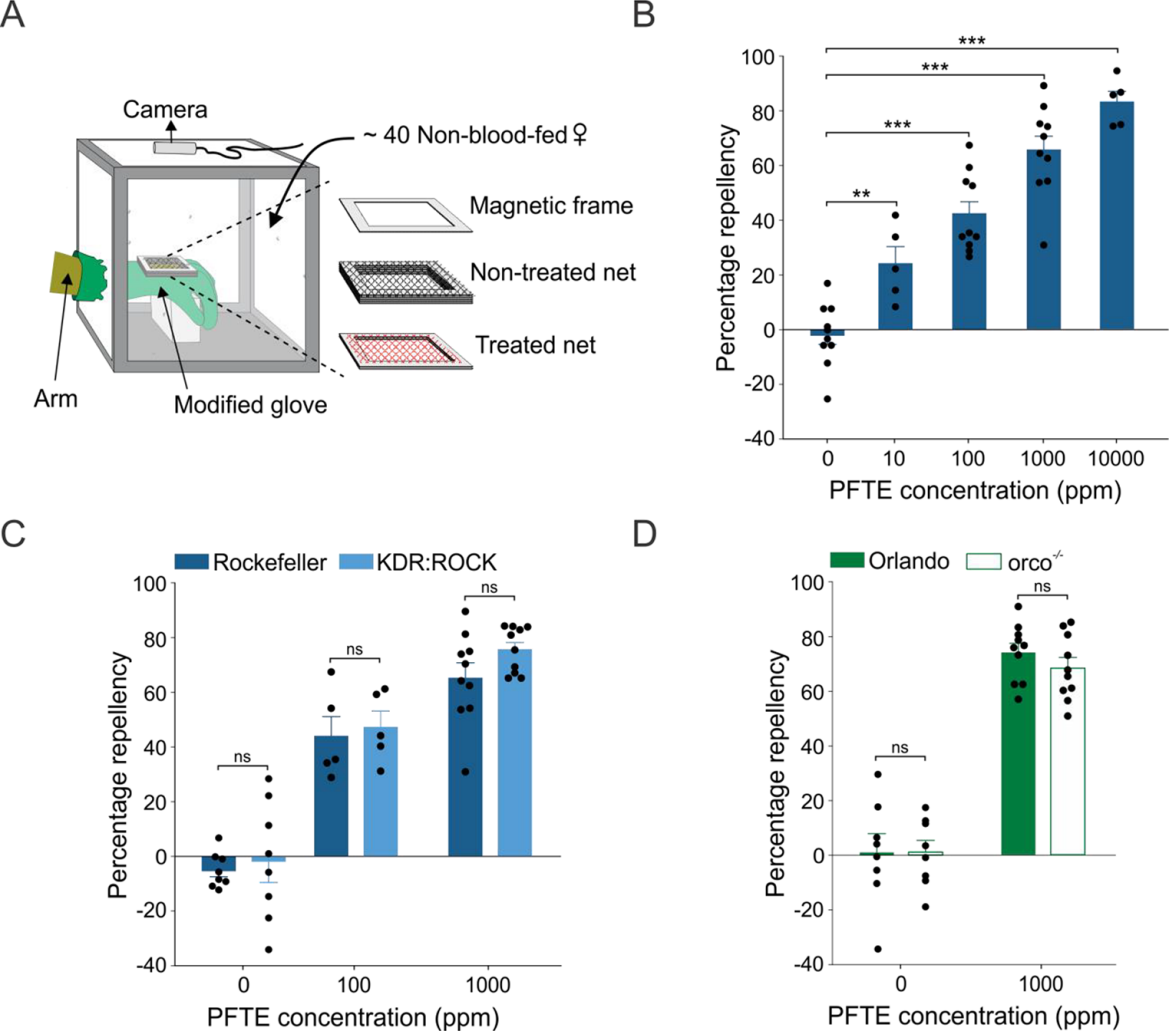
PFTE elicits spatial repellency in *Ae. aegypti* mosquitoes. (A) Schematic drawing illustrating the hand-in-cage setup used to evaluate spatial repellency. (B) Dose-dependent PFTE repellency in Rockefeller (wild-type) mosquitoes. Student’s *t*-test, 10 ppm: *t* = −3.85, *df* = 13, *P* = 0.802; 100 ppm: *t* = −7.50, *df* = 18, *P* < 0.001; 1000 ppm: *t* = −10.49, *df* = 18, *P* < 0.001; 10000 ppm: *t* = −14.35, *df* = 13, *P* < 0.001. *n* values for control (0) = 10 cages, 10 ppm = 5 cages, 100 and 1000 ppm = 10 cages each, 10 000 ppm = 5 cages. (C) PFTE repellency in Rockefeller and KDR:ROCK mosquitoes. Student’s *t*-test, control: *t* = −0.41, *df* = 14, *P* = 0.685; 100 ppm: *t* = −0.34, *df* = 8, *P* = 0.738; 1000 ppm: *t* = −1.69, *df* = 18, *P* = 0.107. *n* values for control (0) for both Rockefeller and KDR:ROCK = 8 cages, 100 ppm for both strain = 5 cages each, 1000 ppm for both strains = 10 cages each. (D) PFTE repellency in Orlando (wild-type) and orco^–/–^ mosquitoes. Student’s *t*-test, control: *t* = −0.02, *df* = 14, *P* = 0.982; 1000 ppm: *t* = 1.13, *df* = 18, *P* = 0.273. *n* values for control (0) for both Orlando and orco^–/–^ = 8 cages, 1000 ppm for both Orlando and orco^–/–^ = 10 cages. Data are presented as mean ± SEM. Dots over the bars represent individual replicate values. ns = not significant, **P < 0.01, ***P < 0.001.

The finding of PFTE vapor toxicity against *Ae. aegypti* prompted us to determine whether PFTE evokes spatial (noncontact) repellency using a hand-in-cage assay,^42^ as shown in Figure 7. PFTE was diluted in acetone and applied on a polyester netting (6.5 cm × 5.5 cm). After the acetone fully evaporated, the netting was fixed in a modified glove worn by a tester. Groups of female Rockefeller mosquitoes were exposed to PFTE vapor for 5 min at concentrations ranging from 10 to 10000 ppm. The repellency indexes at each concentration and control were determined by comparing the number of landings of mosquitoes in two trials: in a first trial with acetone-treated netting only and a second trial with PFTE-treated netting (see details in ref (42)). When the mosquitoes were exposed to PFTE-treated netting, significantly fewer landings were observed. The repellency by PFTE vapor was dose-dependent and observed at concentrations as low as 10 ppm of PFTE (Figure 7B). No mosquitoes were knocked down or exhibited locomotive modifications during the noncontact assay at all tested concentrations.

We showed in a recent study that activation of voltage-gated sodium channels alone by transfluthrin, a volatile pyrethroid, was sufficient to elicit spatial repellency in *Ae. aegypti* and that kdr mutations in sodium channels reduced transfluthrin repellency.^43^ Here, we found that KDR:ROCK mosquitoes exhibited similar levels of repellency compared to wild-type Rockefeller mosquitoes when exposed to PFTE (Figure 7D). This result indicates that, unlike transfluthrin repellency, the kdr mutations did not affect repellency by PFTE, consistent with our toxicity results from contact assays (Figure 6). Taken together, our findings indicate that the mechanism of PFTE repellency is different from that of transfluthrin repellency and appears to be independent of sodium channel activation.

Given that spatial repellency can be elicited by compounds that activate odorant receptors in mosquitoes’ antennae,^42,44^ we examined whether PFTE repellency is mediated by activation of odorant receptor (Or) using an *Ae. aegypti* mutant strain in which the odorant receptor co-receptor gene (*orco*) was mutated.^45^ Orco is required for Or-mediated olfactory activities, and the null mutation of *orco* resulted in impaired Or-mediated olfactory pathways.^45^ We found that PFTE repellency was not reduced in orco^–/–^ mosquitoes compared with wild-type (Orlando) mosquitoes. PFTE at 1000 ppm evoked around 70% repellency in both mosquito strains (Figure 7D). Our findings suggest that spatial repellency by PFTE is not mediated by activation of odorant receptors.

Compared with other compounds that were recently tested against Rockefeller mosquitoes in the hand-in-cage assay, the potency of PFTE in eliciting spatial repellency is comparable to those of plant-derived compounds, but lower than those of DEET and volatile pyrethroids.^46−48^ For instance, eucalyptol and camphor elicited 50–60% repellency at 100 ppm. Geranyl acetate elicited about 70–80% repellency at 100 ppm.^46,48^ PFTE is only slightly more potent than pyrethrin I and II which elicited 50–60% repellency at 1000 ppm.^46^ On the other hand, DEET, transfluthrin, and bioallethrin elicited ∼50% of mosquito repellency at 1–10 ppm.^47,49^

### Rat Oral Toxicity

Compounds **1a** and **1b** were chosen for mammalian oral toxicity screening because their structures and lethality were similar to, and representative of, other compounds identified in Figure 3A. Mammalian toxicity screening was conducted as per the Organization for Economic Co-operation and Development (OECD)’s guideline on the fixed dose procedure using female adult rats.^50^ Compounds **1a** and **1b** both had oral median lethal dose (LD_50_) values in the range of 300–2000 mg/kg and could be allocated to Category 4, according to the Globally Harmonized System (GHS).^51^ This is well above the recommended minimum lethal dosage for public health insecticides (>50 mg/kg; above the range of GHS Category 2).^52^ The results indicated that compounds **1a** and **1b** were *less toxic to mammals* than conventional insecticides, including organochlorines, organophosphates, carbamates, and pyrethroids, and they were comparable to neonicotinoids in mammalian toxicity.^40^

## Conclusions

Fluorine-containing 1-phenyl-2,2,2-trichloro-ethanol (FTEs) exhibit rapid knockdown properties, low mammalian toxicity, and no cross-resistance with DDT or pyrethroids. One enantiomer of any chiral FTE showed faster knockdown speed than its counterpart at various dosages, demonstrating chiral discrimination during the uptake of the molecule or when binding at target site. Furthermore, PFTE possesses both vapor toxicity and spatial repellent actions against *Ae. aegypti* mosquitoes. These results demonstrate the potential of FTEs for vector control, and they provide important insights regarding the unique role of fluorine in the design of active insecticidal and repellent compounds. Nevertheless, more toxicological, electrophysiological, ecological, and epidemiological studies must be performed to better understand the molecular mechanism of FTEs as insecticides and repellents and to fully anticipate unintended consequences, such as insecticide resistance.

## Methods

### Chemicals and Insects

All chemical reagents and solvents were purchased from Sigma-Aldrich or Fischer Chemical and used as supplied. 5 mL glass fine mist spray bottles, purchased from Infinity Jars, Inc., were used for spraying insecticide solutions. Fruit flies (*Drosophila melanogaster*) were raised in-house using standard protocols. We used five *Ae. aegypti* mosquito strains in this study: Rockefeller, KDR:ROCK, Puerto Rico, Orlando, and orco^–/–^. Rockefeller is an insecticide-susceptible wild-type strain. The KDR:ROCK strain was generated by crossing the pyrethroid-resistant strain Singapore^53^ with the Rockefeller strain, followed by four backcrosses to Rockefeller, and selected for the S989P+V1016G haplotype.^37^ The mechanism of resistance in the KDR:ROCK strain is solely due to the two kdr mutations (S989P and V1016G).^37^ Both Rockefeller and KDR:ROCK were kindly provided by J. G. Scott (Cornell University). Puerto Rico is a second pyrethroid-resistant strain (from BEI Resources at the National Institute of Allergy and Infectious Diseases (NIAID), NIH), which carries three kdr mutations, V410L, V1016G, and F1534C, and has an enhanced P450-mediated pyrethroid detoxification mechanism of resistance.^38^ Orlando (kindly provided by L. Vosshall, Rockefeller University) is another insecticide-susceptible wild-type strain, from which an Orco mutant, orco^–/–^ (orco^16^ from BEI Resources, NIAID, NIH), was generated.^45^ In orco^–/–^, the *orco* gene was mutated resulting in impaired Or-mediated olfactory responses. Mosquitoes were maintained in an environmental growth chamber at Duke Phytotron in the Department of Biology, Duke University, at 27 °C, with 50–60% relative humidity and a 12:12 h light/dark photoperiod. Larvae were fed with beef liver powder (NOW Foods), and adults were fed with 10% aqueous sucrose. Adult females were blood-fed 5 days after emergence using defibrinated sheep blood (Colorado Serum Company).

### Synthesis

Details of the chemical syntheses, ^1^H, ^13^C, and ^19^F NMR spectra, and chiral HPLC elution data of compounds **1a**–**1z**, (*R*)-**1a**, (*S*)-**1a**, (*R*)-**1b**, (*S*)-**1b**, **2a**–**2e**, **3a**, **3b**, and **4**–**6** can be found in the Supporting Information. ^1^H, ^13^C, and ^19^F NMR spectra were recorded on a Bruker AVIII 400 MHz spectrometer at 400, 100, and 377 MHz, respectively. Chemical shifts are reported in parts per million (ppm) relative to the residual deuterated chloroform peak (7.26 ppm for ^1^H NMR and 77.23 for proton-decoupled ^13^C NMR). ^19^F NMR chemical shifts are reported relative to the external standard α,α,α-trifluorotoluene, δ −63.72. Data are represented as follows: chemical shift, multiplicity (*s* = singlet, *d* = doublet, *t* = triplet, *q* = quartet, *m* = multiplet), coupling constants (*J*) in Hertz (Hz), integration. HPLC traces were obtained using an Agilent 1260 Infinity instrument with CHIRALPAK OJ-H or OD-H columns.

### Crystallization of Single Crystals

Single crystals of (*R*)-**1a**, (*S*)-**1a**, **1c**, **1e**, **1f**, **1h**, **1i**, **2c**, and **2d** for X-ray analysis were prepared in 20 mL glass vials by slow evaporation from saturated acetone, ethanol, or dichloromethane solutions at room temperature. The X-ray intensity data were recorded on a Bruker D8 APEX-II CCD system using graphite-monochromated and 0.5 mm MonoCap-collimated Mo Kα radiation (λ = 0.71073 Å) with the ω scan method at 100 K. The selected crystallographic parameters are listed in Table S1. Crystallographic information files (CIFs), including the HKL and RES data, are deposited in the CCDC with numbers 2102296 ((*R*)-**1a**), 2102297 ((*S*)-**1a**), 2102298 (**1c**), 2102299 (**1e**), 2102300 (**1f**), 2102301 (**1h**), 2102302 (**1i**), 2102303 (**2c**), and 2102304 (**2d**).

### Toxicological Assays

The lethality of solid-state forms of insecticide was determined by the residual exposure method. Each crystalline form was ground to a particle size similar to the size of the amorphous or liquid droplets prepared by fine mist spraying. For *D. melanogaster* flies, lethality measurements were performed in duplicate for each compound, each accompanied by two controls (no insecticide). Each microcrystalline form was added to a 10 cm diameter polystyrene Petri dish, which was subsequently shaken to disperse the microcrystals throughout the Petri dish. Amorphous or liquid forms were prepared by fine mist spraying a hexane stock solution, with various concentrations in 10 mL hexane, onto the top and bottom of 10 cm diameter polystyrene Petri dishes (two sprays = 0.280 mL) and allowing the hexane to evaporate at room temperature. Flies were sedated with carbon dioxide (for 30 s), and 25 females were transferred to each Petri dish. The top of the dish was then placed over the bottom, and the motion of the flies was recorded with a video camera (Sony HDR-CX455). The knockdown time was measured for each individual fly, with knockdown associated with an insect laying on the bottom surface of the Petri dish in a supine position without moving from its original position after 10 s.

For *Ae. aegypti* mosquitoes, lethality measurements were performed in similar conditions as described for flies. **1a** microcrystalline form (ranging from 0.1 to 1.0 mg) was added to a 9 cm diameter polystyrene Petri dish, which was subsequently shaken to disperse the microcrystals throughout the Petri dish, providing concentrations that ranged from 1.25 to 12.5 μg/cm^2^. Twenty (4–9 days old) female mosquitoes were sedated with carbon dioxide (for 20 s) and transferred to the 9 cm diameter polystyrene Petri dish which was then covered with a lid to prevent their escape. Three Petri dishes containing 20 female mosquitoes each (replicates) were used, totaling 60 mosquitoes for each strain. The number of mosquitoes that were knocked down (i.e., an insect laying on the bottom surface of the Petri dish in a supine position without moving from its original position after 10 s) was recorded every 5 min (for 40 min). For the vapor toxicity assay we used 1.0 mg of **1a** microcrystalline form, which was subsequently shaken to disperse the microcrystals throughout the Petri dish, providing concentrations of 12.5 μg/cm^2^. On top of the treated Petri dish a second modified Petri dish was placed. For that, a 9 cm diameter polystyrene Petri dish with a 7 cm diameter hole was used to create a window, and a polyester netting (Shason Textile Inc., part number WSB532-111, white; 8.0 cm of diameter) was fixed in the bottom of the modified Petri dish to prevent mosquitoes from making direct contact with the compound. This setup allowed the mosquitoes to be exposed to the vapor phase of compound **1a** only (Figure 7B). Twenty (4–9 days old) female mosquitoes were sedated with carbon dioxide (for 20 s) and transferred to the modified Petri dish which was then covered with a lid to prevent their escape. Five replicates (i.e., modified Petri dishes containing 20 female mosquitoes each) were used. The mortality was evaluated after 24 h of exposure.

### Electrophysiology Recordings

We evaluated whether compound **1a** possesses pyrethroid and DDT-like activities on the mosquito wild-type sodium channel (AaNa_v_1-1). AaNa_v_1-1 channels were expressed in *Xenopus* oocytes and functionally characterized using a two-electrode voltage clamp as previously described.^54,55^ Briefly, the tail-current induced by compound **1a** and deltamethrin (positive control) following a 100-pulse train of 5 ms depolarization from −120 mV to 0 mV with 10 ms was measured.^56^ We also tested the inhibitory effect of **1a** on channel inactivation in the DDT protocol^57^ by measuring the remaining current at the end of a 500 ms depolarization to −10 mV from a holding potential of −120 mV. For this protocol cells were previously incubated for 3–4 h in 100 μM PFTE. Six oocytes (i.e., six replicates) expressing AaNa_v_1-1 were tested for both control (i.e., DMSO) and compound **1a**.

### Repellency Assay

We assessed the spatial (i.e., noncontact) repellency elicited by compound **1a** using a hand-in-cage assay.^42^ Briefly, a human hand wearing a modified nitrile glove (Ansell Protective Products, part number 37-155) was placed inside a 30 cm × 30 cm × 30 cm mosquito cage (BioQuip) with a digital camera (e-con Systems Inc., model e-CAM51A) mounted on its top. The camera was connected to a laptop computer to record mosquito landings.^42^ The nitrile rubber glove worn by a tester was cut on its back to create a window (6 cm × 5 cm). A rectangular (6.5 cm × 5.5 cm) magnetic frame was glued onto the cut window, which served as a base for stacking additional magnetic window frames. One piece of test-compound-treated polyester netting (Shason Textile Inc., part number WS-B532-111, Walmart no. 567948282, white; slightly larger than the dimension of the window, but smaller than the outer edge dimensions of the magnetic frames) was placed on this fixed magnetic frame, which was ∼3.0 mm above the glove and hand. To the compound-treated netting was applied 500 μL of either solvent (acetone) or test compound (PFTE) in a glass Petri dish, in an adjacent room. After acetone was fully evaporated (approximately 7 min), the netting was assembled into the window created in the glove. The hand makes no contact with the treated netting. The second piece of the netting was untreated and was placed ∼8.0 mm above the treated netting using a stack of four magnetic frames. The stacked magnetic frames were further secured with two binder clips. The stacking creates sufficient space between the treated netting and the untreated netting so that mosquitoes that land on the open window were not able to contact the treated netting or contact and pierce the skin of the hand in the glove. Eighteen hours before the assay, 4- to 9-day-old female mosquitoes (about 40, mated, non-blood-fed) were transferred into each cage. Groups of 40 female mosquitoes were exposed for 4 min to PFTE at different dilutions ranging from 10 to 10 000 ppm, which correspond to 0.14, 1.4, 14.0, and 140 μg/cm^2^. The cages were kept under controlled conditions inside an environmental growth chamber (27 °C, relative humidity of ∼55%, and 12 h photoperiod). A cotton ball soaked with distilled water was placed on the top of each cage. Any cage that gave a low landing number (i.e., <50% of the average landing compared with other cages) in the first run was not continued with the second run. Repellency index was calculated using the following equation: Percentage repellency = [1 – (cumulative number of landings on the window of treatment/cumulative number of landings on the window of solvent treatment)] × 100). For each concentration or mosquito strain we used at least five cages (i.e., 5 replicates).

### Rat Oral Toxicity Test

The rat oral toxicity test was conducted at Envigo CRS GmbH, Germany, according to the OECD guideline for the fixed dose procedure,^42^ using female adults of the Wistar Ham strain. The test compounds were formulated at concentrations of 30 and 200 mg/mL in corn oil and administered at a constant dose volume of 10 mL/kg body weight. Clinical observations and inspections for morbidity/mortality were performed at least three times within the first 6 h after application, thereafter at least once daily for 14 days. If a rat displayed a high state of pain or distress, it was considered to be not surviving the treatment and was sacrificed immediately.

### Statistical Analyses

Logistic regression of knockdown–time curves was preformed to obtain the median knockdown time (KT_50_) of the test flies and mosquitoes, the 95% confidence intervals (CI), slopes, and standard errors (SE) using Qcal software.^41^ To evaluate the mosquito knockdown time we used the survival analysis and estimated the median lethal time (i.e., KT_50_) applying Kaplan–Meier estimators (Log-rank method) available in SigmaPlot 12.0 (Systat 218 Software, San Jose, California, USA). For the repellency data unpaired Student’s *t* tests were used to compare two sets of data. All statistical analyses and figure plotting were done using SigmaPlot v.12.5 (Systat Software).

## Supporting Information Available

Vapor toxicity and repellency actions, electrophysiology, crystallographic indices, crystal structures, selected crystal data, knockdown–time curves of fluorinated DFDT analogs against *Drosophila*, knockdown–time curves of crystalline and liquid compounds against *Drosophila*, knockdown–time curves of deltamethrin Form I, logistic regression parameters, synthetic procedures, chromatographic elution data for stereoisomers, NMR data, and NMR spectra (PDF)
X-ray crystallographic data for (*R*)-**1a** (CIF)
X-ray crystallographic data for (*S*)-**1a** (CIF)
X-ray crystallographic data for **1c** (CIF)
X-ray crystallographic data for **1e** (CIF)
X-ray crystallographic data for **1f** (CIF)
X-ray crystallographic data for **1h** (CIF)
X-ray crystallographic data for **1i** (CIF)
X-ray crystallographic data for **2c** (CIF)
X-ray crystallographic data for **2d** (CIF)

## Supplementary Material

id3c00161_si_001.pdf

id3c00161_si_002.cif

id3c00161_si_003.cif

id3c00161_si_004.cif

id3c00161_si_005.cif

id3c00161_si_006.cif

id3c00161_si_007.cif

id3c00161_si_008.cif

id3c00161_si_009.cif

id3c00161_si_010.cif
