# Supplementary material for: Insecticidal and Repellent Properties of Rapid-Acting Fluorine-Containing Compounds against Aedes aegypti Mosquitoes
Source: ACS Infect Dis. 2023 Jun 13;9(7):1396–407. doi: 10.1021/acsinfecdis.3c00161 (PMC10353007; doi:10.1021/acsinfecdis.3c00161)
Supplement: Supplementary file 1 — id3c00161_si_001.pdf [file id3c00161_si_001.pdf]

Supporting Information

Insecticidal and repellent properties of rapid-acting fluorine-containing compounds against *Aedes aegypti* mosquitoes

Xiaolong Zhu,[a] Wilson Valbon,[b] Mengdi, Qiu,[a] Chunhua T. Hu,[a] Jingxiang Yang,[a] Bryan Erriah,[a] Milena Jankowska,[bc] Ke Dong,[b]* Michael D. Ward,[a]* Bart Kahr[a]*

[a]Department of Chemistry and Molecular Design Institute, New York University, 100 Washington Square East, New York, NY 10003 USA

[b] Department of Biology, Duke University, 130 Science Drive, Durham, NC 27708 USA

[c]Department of Animal Physiology and Neurobiology, Nicolaus Copernicus University, Lwowska 1 Street Toruń, 87-100 Poland

Corresponding authors: Ke Dong; Michael D. Ward; Bart Kahr

Emails: ke.dong@duke.edu (K.D.)*; mdw3@nyu.edu (M.D.W.)*; bart.kahr@nyu.edu (B.K.)*

Table of Contents

Figure S1. Vapor toxicity and repellency actions of crystalline compound PFTE (1a) against *Ae. aegypti* mosquitoes.

Figure S2. Unlike pyrethroids and DDT, PFTE does not alter the gating of *Ae. aegypti* voltage-gated sodium channels (AaNav1-1) expressed in *Xenopus* oocytes

Figure S3. Single crystals with Miller indices.

Figure S4. Crystal packing diagrams in unit cells.

Table S1. Selected crystal data.

Figure S5. Knockdown-time curves of fluorinated DFDT analogs against *Drosophila*.

Figure S6. Knockdown-time curves of crystalline compounds against *Drosophila*.

Figure S7. Knockdown-time curves of liquid compounds against *Drosophila*.

Figure S8. Knockdown-time curves of deltamethrin Form I against *Drosophila*.

Table S2. Parameters obtained from logistic regression of knockdown-time curves of *Drosophila*.

Procedures for the synthesis of compounds 1a–1z, (*R*)-1a, (*S*)-1a, (*R*)-1b, (*S*)-1b, 2a–2e, 3a, 3b, and 4–6.

Elution data for asymmetric synthesis of 1a and 1b enantiomers.

NMR data

NMR spectra

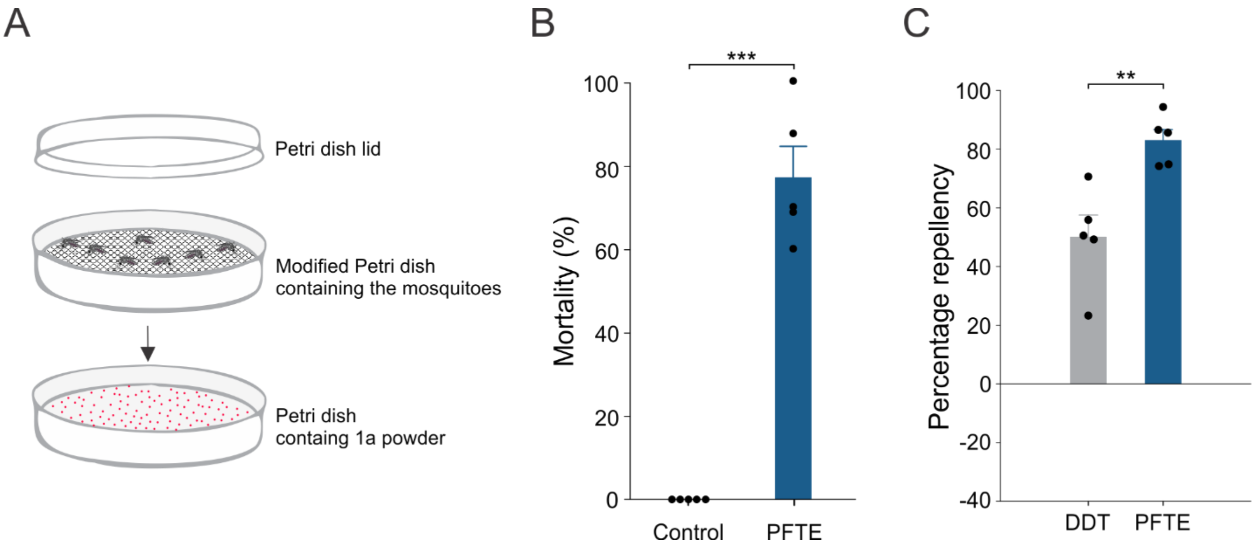

Figure S1. Vapor toxicity and repellency of PFTE against wild-type (Rockefeller) *Ae. aegypti* mosquitoes. (A) Schematic drawing of the vapor toxicity assay, containing the PFTE inside the bottom Petri dish, which was covered by a modified Petri dish containing the mosquitoes and covered by a lid to avoid insect escaping. The mosquitoes were exposed to the vapor phase of PFTE at 12.5 µg/cm^2. (B) Mortality (%) of Rockefeller mosquitoes after 24 h of exposure to PFTE; t = -10.68, df = 8, P < 0.001 n = 5 Petri dishes for control and PFTE. (C) Repellency of DDT and PFTE at 10000 ppm; t = 3.87, df = 8, P = 0.004; n = 5 cages for DDT and PFTE. Data are presented as mean ± SEM. Dots over the bars represent individual replicate values. **P < 0.01, ***P < 0.001.

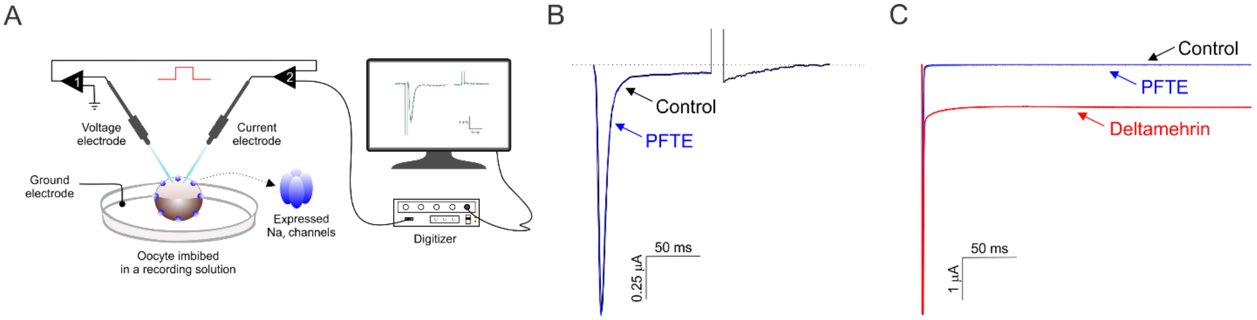

Figure S2. Unlike pyrethroids and DDT, PFTE does not alter the gating of *Ae. aegypti* voltage-gated sodium channels (AaNa$_v$1-1) expressed in *Xenopus* oocytes. (A) Schematic drawing of the two-electrode voltage clamp recording from *Xenopus* oocytes. (B) Representative sodium current traces from oocytes expressing AaNav1-1 channel before or after 3-4 h incubation with 100 µM of PFTE. The recording protocol was 500-ms depolarization to -10 mV from a holding potential of -120 mV. (C) Deltamethrin (3 µM) induced a tail current, but PFTE (100 µM) did not, when a 100-pulse train of 5-ms depolarization from -120 mV to 0 mV with 5-ms interval was applied to measure any current associated with repolarization. The number of oocytes for each treatment was six.

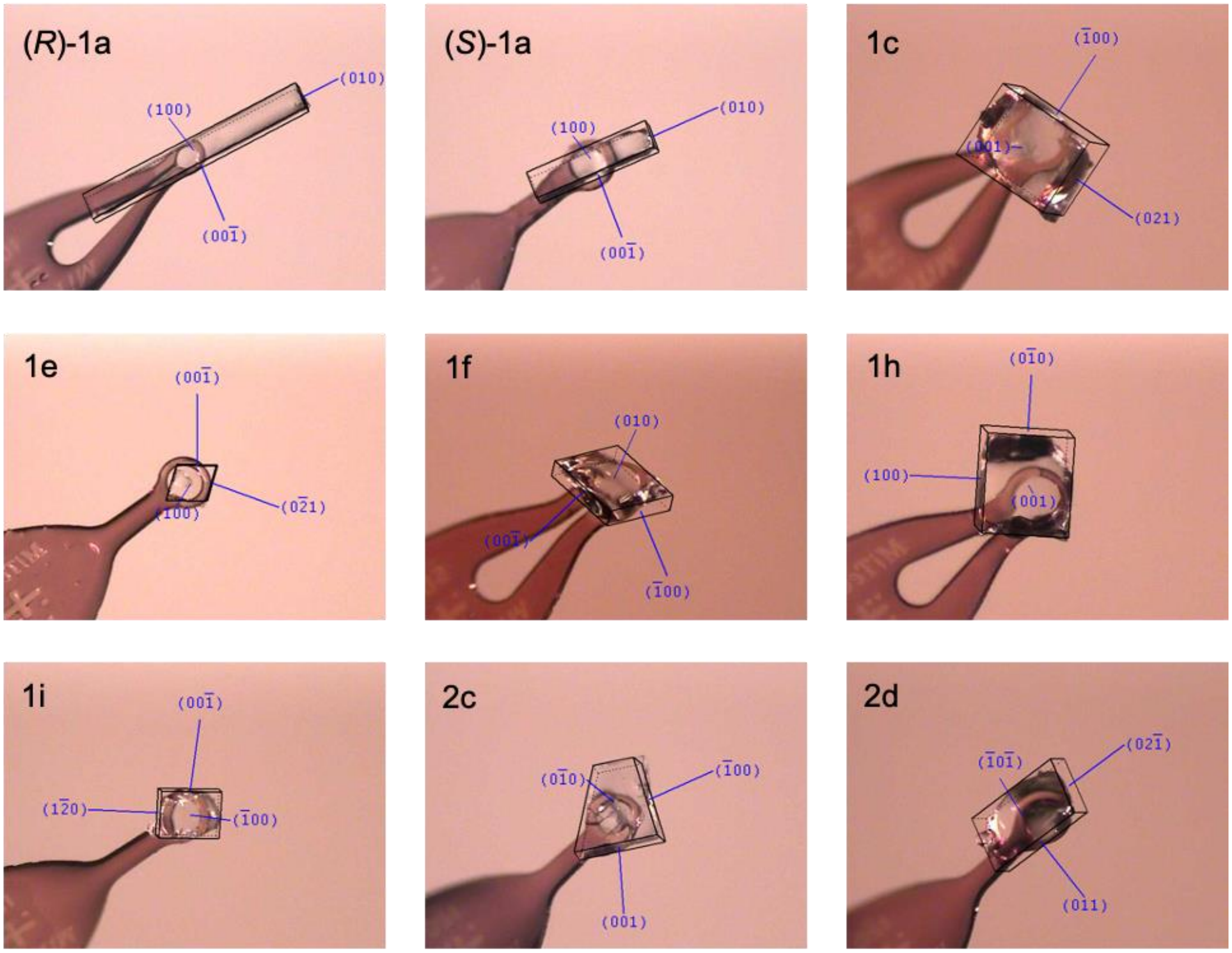

Figure S3. Single crystals with Miller indices.

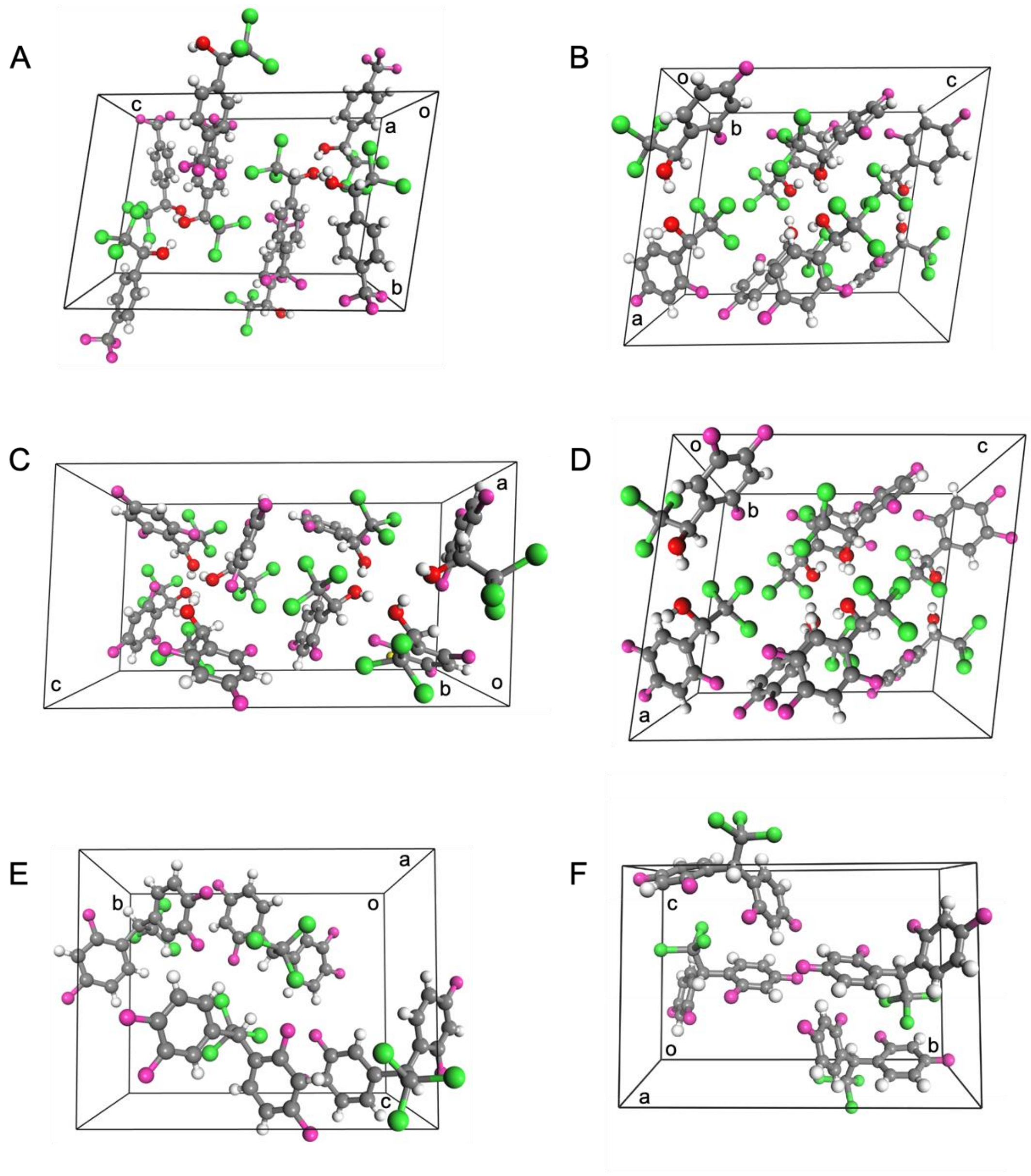

Figure S4. Crystal packing diagrams in unit cells. Compounds **1c** (A), **1e** (B), **1h** (C), **1i** (D), **2d** (E), and **2c** (F) all crystallized into racemic mixtures. Crystal structure of **1c** is disordered, minor domain is omitted for clarity. Atom color: carbon (gray); hydrogen (light gray); oxygen (red); nitrogen (cyan); chlorine (green).

Table S1. Selected crystal data.

	(*R*)-1a	(*S*)-1a	1c
Formula	$C_8H_2Cl_3F_5O$	$C_8H_2Cl_3F_5O$	$C_9H_6Cl_3F_3O$
Formula weight	315.45	315.45	293.49
Crystal system	Monoclinic	Monoclinic	Triclinic
Space group (no.)	$P2_1$ (4)	$P2_1$ (4)	$P\overline{1}$ (2)
a (Å)	7.3453(13)	7.3386(7)	10.3650(8)
b (Å)	5.7253(11)	5.7238(5)	11.7883(9)
c (Å)	12.306(2)	12.3016(11)	18.3335(14)
α (°)	90	90	80.9149(12)
β (°)	90.565(3)	90.5707(14)	87.0143(12)
γ (°)	90	90	89.8922(11)
V (Å^3)	517.51(17)	516.70(8)	2208.9(3)
Z	2	2	8
D_c (g cm^{-3})	2.024	2.028	1.765
μ (mm^{-1})	0.934	0.936	0.843
F(000)	308	308	1168
Total reflections	7827	7695	47345
Unique reflections	2572	2582	10989
R_{int}	0.0373	0.0314	0.0176
GOF	1.019	1.044	1.046
R_1[a] [$I > 2\sigma(I)$]	0.0310	0.0313	0.0315
wR_2[b] (all data)	0.0705	0.0688	0.0802

[a] $R_1 = \Sigma||F_o|-|F_c||/\Sigma|F_o|$; [b] $wR_2=\{\Sigma[w(F_o^2-F_c^2)^2]/\Sigma[w(F_o^2)^2]\}^{1/2}$

Table S1 (continued). Selected crystal data.

	1e	1f	1h
Formula	$C_8H_5Cl_3F_2O$	$C_8H_5Cl_3F_2O$	$C_8H_4Cl_3F_3O$
Formula weight	261.47	261.47	279.46
Crystal system	Monoclinic	Triclinic	Monoclinic
Space group (no.)	$P2_1/c$ (14)	$P\overline{1}$ (2)	$P2_1/c$ (14)
a (Å)	13.112(2)	9.7026(6)	11.2783(9)
b (Å)	9.9704(19)	11.0026(6)	8.4391(6)
c (Å)	15.393(3)	11.4241(6)	21.3669(16)
α (°)	90	115.9730(10)	90
β (°)	98.542(2)	104.8730(10)	92.5260(10)
γ (°)	90	100.9500(10)	90
V (Å^3)	1990.0(6)	993.43(10)	2031.7(3)
Z	8	4	8
D_c (g cm^{-3})	1.745	1.748	1.827
μ (mm^{-1})	0.910	0.912	0.912
F(000)	1040	520	1104
Total reflections	22605	21288	30765
Unique reflections	3516	4934	5051
R_{int}	0.0867	0.0196	0.0164
GOF	1.010	1.055	1.045
$R_1{}^a$ [$I > 2\sigma(I)$]	0.0417	0.0218	0.0222
$wR_2{}^b$ (all data)	0.1000	0.0579	0.0547

$^a R_1 = \Sigma||F_o|-|F_c||/\Sigma|F_o|$; $^b wR_2=\{\Sigma[w(F_o{}^2-F_c{}^2)^2]/\Sigma[w(F_o{}^2)^2]\}^{1/2}$

Table S1 (continued). Selected crystal data.

	1i	2c	2d
Formula	$C_8H_4Cl_3F_3O$	$C_{14}H_7Cl_3F_4$	$C_{14}H_7Cl_3F_4$
Formula weight	279.46	357.55	357.55
Crystal system	Monoclinic	Monoclinic	Monoclinic
Space group (no.)	$P2_1/c$ (14)	$P2_1/n$ (14)	$P2_1/n$ (14)
a (Å)	13.1055(10)	6.3648(6)	7.2069(8)
b (Å)	10.1395(8)	18.1002(17)	15.6466(17)
c (Å)	15.2432(12)	12.2902(11)	12.2228(13)
α (°)	90	90	90
β (°)	97.5290(10)	103.0010(10)	90.4039(16)
γ (°)	90	90	90
V (Å^3)	2008.1(3)	1379.6(2)	1378.3(3)
Z	8	4	4
D_c (g cm^{-3})	1.849	1.721	1.723
μ (mm^{-1})	0.922	0.697	0.698
F(000)	1104	712	712
Total reflections	30675	20185	21102
Unique reflections	5010	3423	3430
R_{int}	0.0292	0.0228	0.0331
GOF	1.038	1.071	1.046
R_1[a] [$I > 2\sigma(I)$]	0.0284	0.0302	0.0344
wR_2[b] (all data)	0.0758	0.0790	0.0956

[a] $R_1 = \Sigma||F_o|-|F_c||/\Sigma|F_o|$; [b] $wR_2=\{\Sigma[w(F_o^2-F_c^2)^2]/\Sigma[w(F_o^2)^2]\}^{1/2}$

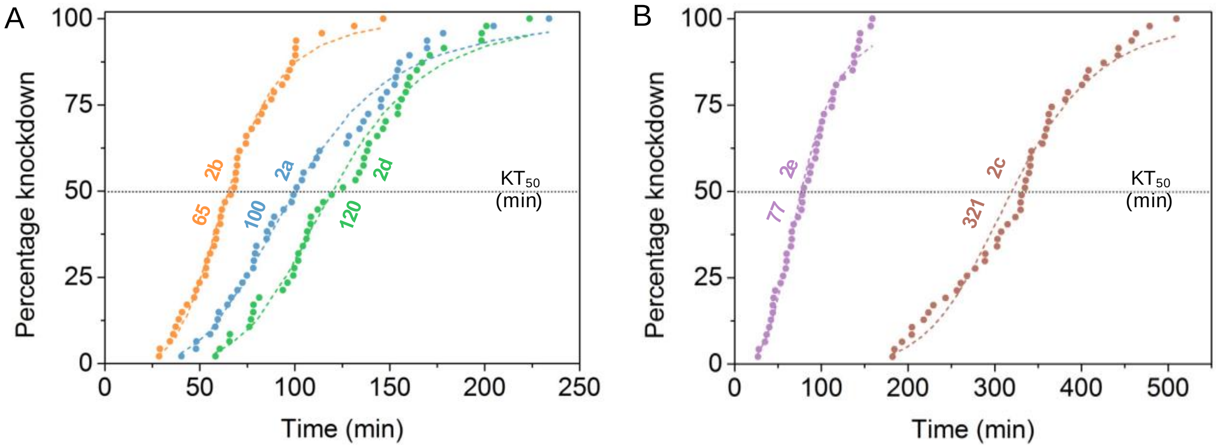

Figure S5. Knockdown-time curves of fluorinated DFDT analogs against *Drosophila*. The median knockdown time for each curve is denoted by its intersection with the horizontal KT_{50} marker. KT_{50} values of these knockdown-time curves are shown in Figure 3C.

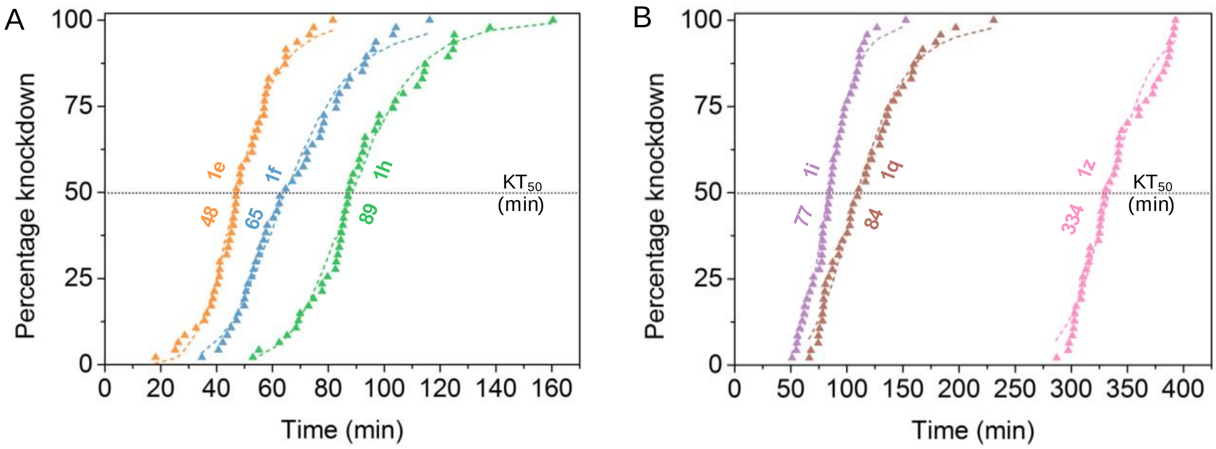

Figure S6. Knockdown-time curves of crystalline compounds against *Drosophila*. The median knockdown time for each curve is denoted by its intersection with the horizontal KT_{50} marker. KT_{50} values of these knockdown-time curves are shown in Figure 4.

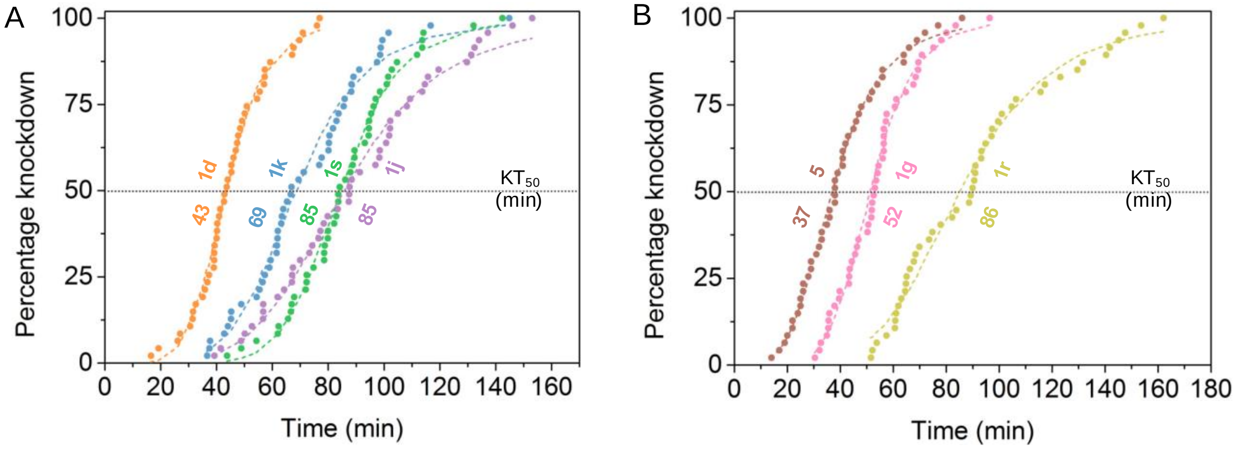

Figure S7. Knockdown-time curves of liquid compounds against *Drosophila*. The median knockdown time for each curve is denoted by its intersection with the horizontal KT_{50} marker. KT_{50} values of these knockdown-time curves are shown in Figure 4.

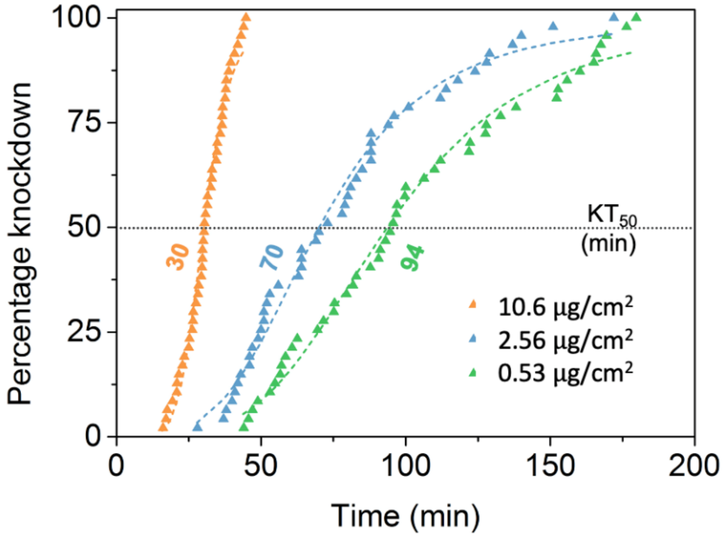

Figure S8. Knockdown-time curves of deltamethrin Form I against *Drosophila*. The median knockdown time for each curve is denoted by its intersection with the horizontal KT_{50} marker. KT_{50} values of these knockdown-time curves are shown in Figure 6F.

Table S2. **Parameters obtained from logistic regression of knockdown-time curves of *Drosophila*.** Median knockdown times (KT_{50}), 95% confidence intervals (CI), slopes, intercepts, and standard errors (SE) of the logistic regression. Values with the same superscript are identical within the 95% CIs.

Compound	Dose ($\mu g/cm^2$)	Physical State	KT_{50} (95%CI) (min)	Slope ± SE	Intercept ± SE
1a	10.6	crystal	35.71[a] (34.70–36.75)	3.56 ± 0.15	-12.72 ± 0.56
(*R*)-1a	10.6	crystal	42.48 (41.32–43.68)	4.15 ± 0.21	-15.55 ± 0.81
(*S*)-1a	10.6	crystal	81.74 (79.76–83.77)	4.16 ± 0.17	-18.33 ± 0.77
1a	2.65	crystal	51.59[b] (50.32–52.89)	0.49 ± 0.19	-16.12 ± 0.74
(*R*)-1a	2.65	crystal	52.12[b] (51.15–53.11)	5.41 ± 0.25	-21.41 ± 1.00
(*S*)-1a	2.65	crystal	112.96 (110.83–115.12)	5.38 ± 0.24	-25.45 ± 1.12
1a	0.53	crystal	183.60 (175.48–192.10)	2.26 ± 0.10	-11.79 ± 0.51
(*R*)-1a	0.53	crystal	70.58 (68.83–72.39)	4.06 ± 0.18	-17.27 ± 0.76
(*S*)-1a	0.53	crystal	336.62 (326.13–347.44)	3.31 ± 0.15	-19.24 ± 0.89
1b	10.6	liquid	28.54[b] (27.92–29.87)	4.71 ± 0.21	-15.79 ± 0.72
(*R*)-1b	10.6	liquid	20.93 (20.30–21.58)	3.36 ± 0.14	-10.21 ± 0.44
(*S*)-1b	10.6	liquid	34.33 (33.10–35.62)	2.86 ± 0.13	-10.13 ± 0.45
1b	2.65	liquid	50.17 (48.88–51.49)	3.92 ± 0.19	-15.36 ± 0.73
(*R*)-1b	2.65	liquid	41.83 (40.62–43.09)	3.46 ± 0.16	-12.93 ± 0.60
(*S*)-1b	2.65	liquid	101.66 (97.91–105.55)	2.72 ± 0.12	-12.56 ± 0.56
1c	10.6	crystal	167.75 (165.37–170.15)	7.22 ± 0.32	-37.00 ± 1.66
1d	10.6	liquid	43.48 (42.73–44.25)	5.84 ± 0.28	-22.04 ± 1.05
1e	10.6	crystal	47.67 (46.91–48.44)	6.35 ± 0.30	-24.53 ± 1.16

1f	10.6	crystal	65.00[c] (63.80–66.21)	5.52 ± 0.24	-23.02 ± 0.99
1g	10.6	crystal	51.67 (50.82–52.54)	6.12 ± 0.28	-24.13 ± 1.09
1h	10.6	crystal	89.02 (87.87–90.19)	7.80 ± 0.37	-35.00 ± 1.65
1i	10.6	crystal	110.29 (108.07–114.80)	5.05 ± 0.22	-23.73 ± 1.02
1j	10.6	liquid	85.45[c] (83.65–87.30)	4.79 ± 0.21	-21.30 ± 0.94
1k	10.6	liquid	68.57 (67.27–69.89)	5.36 ± 0.24	-22.67 ± 1.01
1q	10.6	crystal	84.02[c] (82.76–85.29)	6.76 ± 0.30	-29.95 ± 1.35
1r	10.6	liquid	85.63[c] (83.89–87.43)	4.96 ± 0.22	-22.06 ± 0.96
1s	10.6	liquid	84.54[c] (83.10–85.67)	7.70 ± 0.36	-34.18 ± 1.60
1z	10.6	crystal	334.47 (332.45–336.50)	16.82 ± 0.73	-97.74 ± 4.24
5	10.6	liquid	37.14[a] (36.20–38.09)	4.02 ± 0.18	-14.53 ± 0.65
2a	10.6	amorph	99.94 (97.27–102.70)	3.77 ± 0.16	-17.38 ± 0.75
2b	10.6	liquid	65.00[c] (63.52–66.52)	4.44 ± 0.20	-18.52 ± 0.84
2c	10.6	amorph	320.55 (315.36–325.84)	6.27 ± 0.29	-36.20 ± 1.68
2d	10.6	amorph	119.86 (117.30–122.46)	4.76 ± 0.21	-22.77 ± 1.01
2e	10.6	liquid	77.31 (74.99–79.70)	3.37 ± 0.15	-14.64 ± 0.65
Deltamethrin	10.6	crystal	30.28[b] (29.81–30.76)	6.52 ± 0.30	-22.24 ± 1.02
Deltamethrin	2.65	crystal	70.25 (68.29–72.25)	3.63 ± 0.16	-15.42 ± 0.67
Deltamethrin	0.53	crystal	93.73 (91.21–96.31)	3.76 ± 0.16	-17.06 ± 0.74

Procedures for the synthesis of compounds 1a–1z, (*R*)-1a, (*S*)-1a, (*R*)-1b, (*S*)-1b, 2a–2e, 3a, 3b, and 4–6.

1. General procedures for the synthesis of compounds 1a–1z.

Benzaldehyde (15.0 mmol) was dissolved in 20 mL of DMF, after cooling to 0 °C, trichloroacetic acid (3.68 g, 22.5 mmol) was added to the solution. This was followed by the addition of sodium trichloroacetate (4.17 g, 22.5 mmol). Then the reaction mixture was stirred at room temperature and monitored by thin layer chromatography (TLC) (hexane/ethyl acetate: 1/4), during which there was an evolution of gas bubbles and complete dissolution of sodium trichloroacetate. After the reaction was completed, 50 mL of water was added to the solution. The mixture was extracted with ethyl acetate (3 x 40 mL). Organic extracts were combined and washed with saturated sodium bicarbonate solution (2 x 50 mL).[1] The organic phase was dried with sodium sulfate and concentrated under reduced pressure. The resulting residue was purified by silica column chromatography (hexane/ethyl acetate: 1/19) to afford the corresponding product.

2. General procedures for the synthesis of compounds 2a–2e.

Halogenobenzene (36 mmol) was dissolved in concentrated sulfuric acid (12 mL) at room temperature. After cooling to 0 °C, trichlorocarbinol (12 mmol) was added dropwise to the solution. After the addition was complete, the mixture was allowed to reflux at 60 °C and monitored by TLC (hexane/ethyl acetate: 1/15).[2] After the reaction was complete (12 h), the mixture was cooled to room temperature, poured into ice-cold water (50 mL), and extracted with dichloromethane (3 x 25 mL). Organic extracts were combined and washed with 5% sodium hydroxide (2 x 50 mL) until neutral. The organic phase was dried with sodium sulfate and concentrated under reduced pressure. The resulting residue was purified by silica column chromatography (hexane/ethyl acetate: 1/99) to afford the corresponding product.

3. General procedures for the synthesis of compounds 3a, 3b.

1-phenyl-2,2,2-trichloro-ethanol (21.0 mmol) was added to a stirred suspension of Dess-Martin reagent (10.8 g, 22.5 mmol) in 100 mL CH_2Cl_2, and the reaction mixture was stirred at room temperature for 3 hours. The reaction was quenched with 50 mL saturated aqueous sodium bicarbonate and 50 mL sodium thiosulfate (20%) solution, and then stirred for 10 min.[3] The aqueous layer was extracted with CH_2Cl_2 (50 mL x 3). Organic extracts were combined and washed with saturated sodium chloride solution (2 x 50 mL). The organic phase was dried with sodium sulfate and concentrated under reduced pressure. The resulting residue was purified by silica column chromatography (hexane/ethyl acetate: 1/9) to afford the corresponding product.

4. General procedures for the synthesis of compounds (*R*)-1a, (*S*)-1a, (*R*)-1b, and (*S*)-1b.

Under nitrogen, to a solution of fluorinated 1-phenyl-2,2,2-trichloro-ethanone (**3a** or **3b**; 3.20 mmol) in toluene (25.0 mL) was added a 1M solution of oxazaborolidine catalyst in THF (0.32 mL) at room temperature ((*R*)-oxazaborolidine catalyst for the production of **(*S*)-1a** or **(*S*)-1b**; ((*S*)-oxazaborolidine catalyst for the production of **(*R*)-1a** or **(*R*)-1b**). The reaction mixture was cooled to -78 °C, and 6.40 mL 1M solution of catecholborane in THF was added dropwise. The reaction was stirred at -78 °C for 6 hours and then at room temperature for 10 hours.4 The mixture was quenched by 150 mL of water and extracted with ethyl acetate (3 x 50 mL). Organic extracts were combined and washed with a 1M aqueous sodium hydroxide solution (3 x 75 mL), a 1 M aqueous hydrochloric acid solution (3 x 50 mL), and a saturated sodium chloride solution (2 x 50 mL). The organic phase was dried with sodium sulfate and concentrated under reduced pressure. The resulting residue was purified by silica column chromatography (hexane/ethyl acetate: 1/49) to afford the corresponding product. The enantiomeric excess was determined by HPLC analysis.

5. General procedures for the synthesis of compound 4.

To a solution of 1-(perfluorophenyl)-2,2,2-trichloro-ethanol (**1a**; 0.50 g, 1.6 mmol) and DMAP (0.20 g, 1.6 mmol) in pyridine (0.16 g, 2.0 mmol) at 0 °C was added acetic anhydride (0.49 g, 4.8 mmol).5 The reaction mixture was stirred at room temperature for three hours. The mixture was then quenched by 50 mL of water and extracted with ethyl acetate (3 x 50 mL). Organic extracts were combined and washed with dilute hydrochloric acid (2 x 50 mL), saturated sodium bicarbonate solution (2 x 50 mL), and saturated sodium chloride solution (2 x 50 mL). The organic phase was dried with sodium sulfate and concentrated under reduced pressure. The resulting residue was purified by silica column chromatography (hexane/ethyl acetate: 1/49) to afford the product (0.52 g, 1.46 mmol).

6. General procedures for the synthesis of compound 5.

1-(perfluorophenyl)-ethan-1-one (3.15 g, 15 mmol) was dissolved in 20 mL of DMF, after cooling to 0 °C, trichloroacetic acid (3.68 g, 22.5 mmol) was added to the solution. This was followed by the addition of sodium trichloroacetate (4.17 g, 22.5 mmol).[1] Then the reaction mixture was stirred at room temperature and monitored by TLC (hexane/ethyl acetate: 1/4), during which there was an evolution of gas bubbles and complete dissolution of sodium trichloroacetate. After the reaction was completed, 50 mL of water was added to the solution. The mixture was extracted with ethyl acetate (3 x 40 mL). Organic extracts were combined and washed with saturated sodium bicarbonate solution (2 x 50 mL). The organic phase was dried with sodium sulfate and concentrated under reduced pressure. The resulting residue was purified by silica column chromatography (hexane/ethyl acetate: 1/19) to afford the product (4.54 g, 13.8 mmol).

7. General procedures for the synthesis of compound 6.

4-(trifluoromethyl)benzaldehyde (2.61 g, 15 mmol) was dissolved in 20 mL of DMSO, after cooling to 0 °C, tribromoacetic acid (6.05 g, 22.5 mmol) was added to the solution.6 Then the reaction mixture was stirred overnight at room temperature. After the reaction was completed, 50 mL of water was added to the solution. The mixture was extracted with ethyl acetate (3 x 50 mL). Organic extracts were combined and washed with saturated sodium chloride solution (2 x 50 mL). The organic phase was dried with sodium sulfate and concentrated under reduced pressure. The resulting residue was purified by silica column chromatography (hexane/ethyl acetate: 1/19) to afford the product (5.83 g, 13.7 mmol).

Elution data for asymmetric synthesis of 1a and 1b enantiomers.

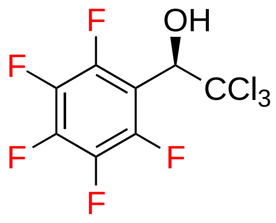

Compound (*R*)-1a

(*R*)-1-perfluorophenyl-2,2,2-trichloro-ethanol

Colorless crystals in 95% yield (0.96 g, 3.04 mmol), 99.58% ee.

All physical and spectroscopic data were identical to racemic 1-perfluorophenyl-2,2,2-trichloro-ethanol.

HPLC CHIRALPAK OJ-H column (5% isopropanol in hexane, 0.7 mL/min), detection: UV 210, retention time: t_r = 7.364 min (minor), 16.486 min (major), 99.58% ee.

The absolute stereochemistry was assigned by the single crystal X-ray determination.

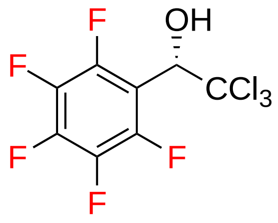

Compound (*S*)-1a

(*S*)-1-perfluorophenyl-2,2,2-trichloro-ethanol

Colorless crystals in 95% yield (0.96 g, 3.04 mmol), 98.12% ee.

All physical and spectroscopic data were identical to racemic 1-perfluorophenyl-2,2,2-trichloro-ethanol. HPLC CHIRALPAK OJ-H column (5% isopropanol in hexane, 0.7 mL/min), detection: UV 210, retention time: t_r = 7.465 min (major), 17.042 min (minor), 98.12% ee.

The absolute stereochemistry was assigned by the single crystal X-ray determination.

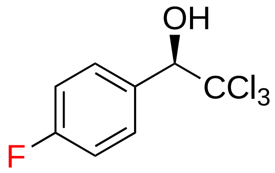

(*R*)-1b

(*R*)-1-(4-fluorophenyl)-2,2,2-trichloro-ethanol

Colorless oil in 90% yield (0.70 g, 2.88 mmol), 95.28% ee.

All physical and spectroscopic data were identical to racemic 1-(4-fluorophenyl)-2,2,2-trichloro-ethanol.

HPLC CHIRALPAK OD-H column (5% isopropanol in hexane, 0.7 mL/min), detection: UV 210, retention time: t_r = 14.160 min (major), 17.715 min (minor), 95.28% ee.

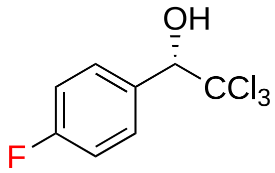

Compound (*S*)-1b

(*S*)-1-(4-fluorophenyl)-2,2,2-trichloro-ethanol

Colorless oil in 90% yield (0.70 g, 2.88 mmol), 94.38% ee.

All physical and spectroscopic data were identical to racemic 1-(4-fluorophenyl)-2,2,2-trichloro-ethanol.

HPLC CHIRALPAK OD-H column (5% isopropanol in hexane, 0.7 mL/min), detection: UV 210, retention time: t_r = 13.355 min (minor), 16.503 min (major), 94.38% ee.

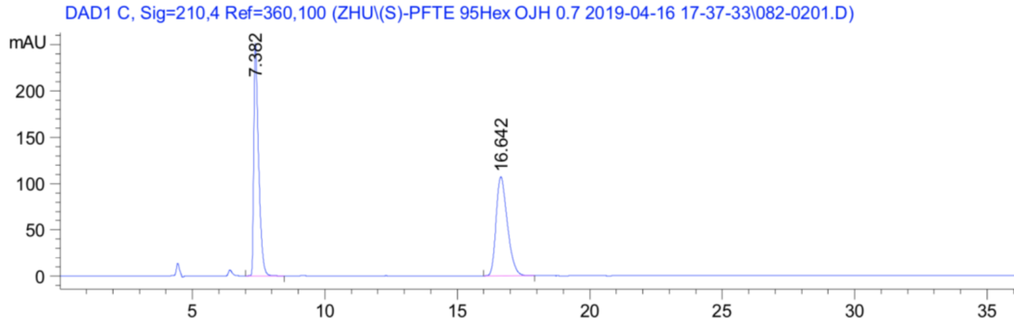

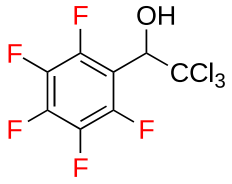

1a

Peak #	RetTime [min]	Type	Width [min]	Area [mAU*s]	Height [mAU]	Area %
1	7.382	BB	0.1793	3121.33447	250.70918	49.6354
2	16.642	BB	0.4538	3167.18848	106.85397	50.3646

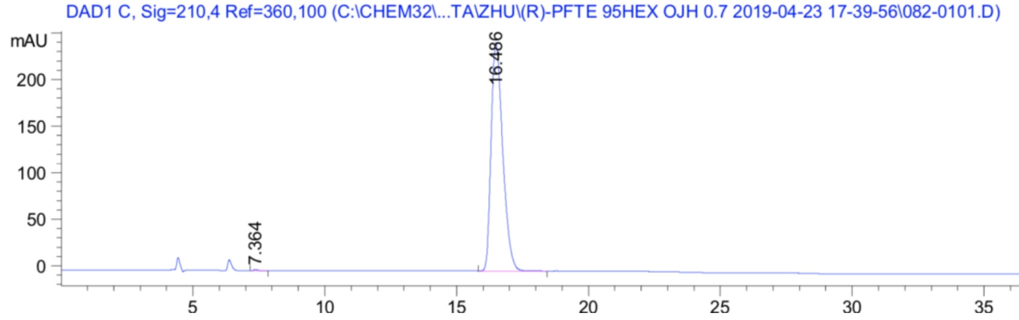

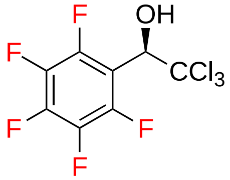

(*R*)-1a

Peak #	RetTime [min]	Type	Width [min]	Area [mAU*s]	Height [mAU]	Area %
1	7.364	BB	0.1832	16.17741	1.31800	0.2145
2	16.486	BB	0.4741	7525.25293	245.15503	99.7855

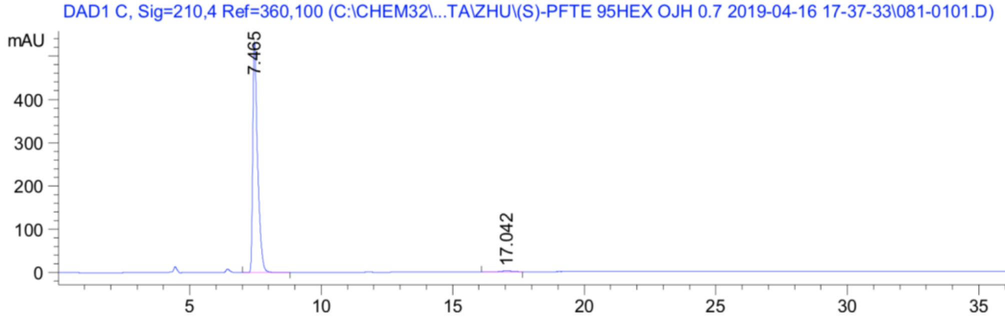

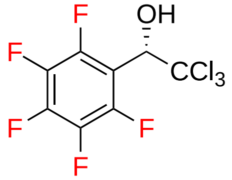

(*S*)-1a

Peak #	RetTime [min]	Type	Width [min]	Area [mAU*s]	Height [mAU]	Area %
1	7.465	BB	0.1844	6767.76123	531.92261	99.0568
2	17.042	BB	0.4630	64.43958	2.16646	0.9432

DAD1 C, Sig=210,4 Ref=360,100 (C:\CHEM32\...TA\ZHU\(S)-MFTE 95HEX ODH 0.7 2019-04-18 14-42-59\082-0201.D)

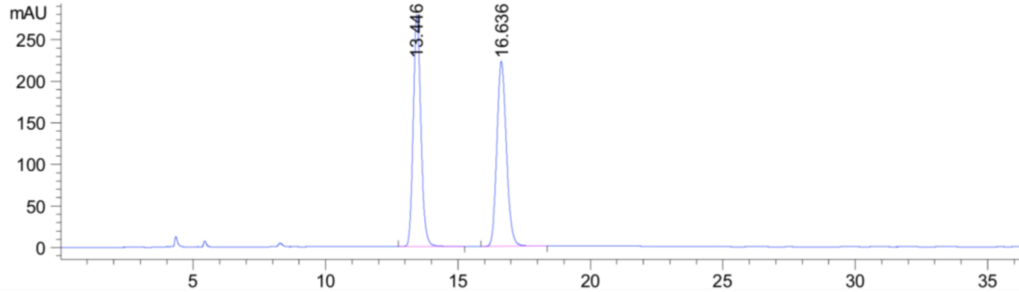

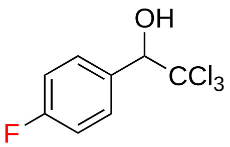

1b

Peak #	RetTime [min]	Type	Width [min]	Area [mAU*s]	Height [mAU]	Area %
1	13.446	BB	0.3101	5584.45801	278.25452	49.9986
2	16.636	BB	0.3887	5584.77979	222.56502	50.0014

DAD1 C, Sig=210,4 Ref=360,100 (C:\CHEM32\...TA\ZHU\(R)-MFTE 95HEX ODH 0.7 2019-04-23 16-23-47\081-0101.D)

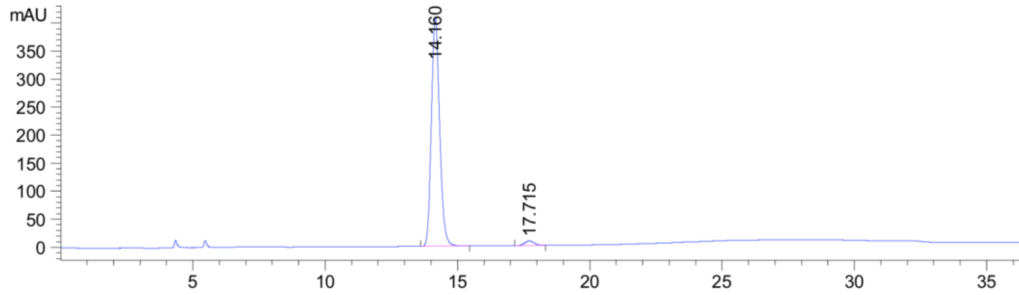

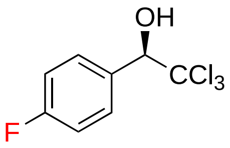

(*R*)-1b

Peak #	RetTime [min]	Type	Width [min]	Area [mAU*s]	Height [mAU]	Area %
1	14.160	BB	0.3317	8828.21680	409.27985	97.6408
2	17.715	BB	0.4018	213.31161	8.24716	2.3592

DAD1 C, Sig=210,4 Ref=360,100 (C:\CHEM32\...\ZHU\(S)-MFTE 2 95HEX ODH 0.7 2019-04-18 17-22-46\081-0101.D)

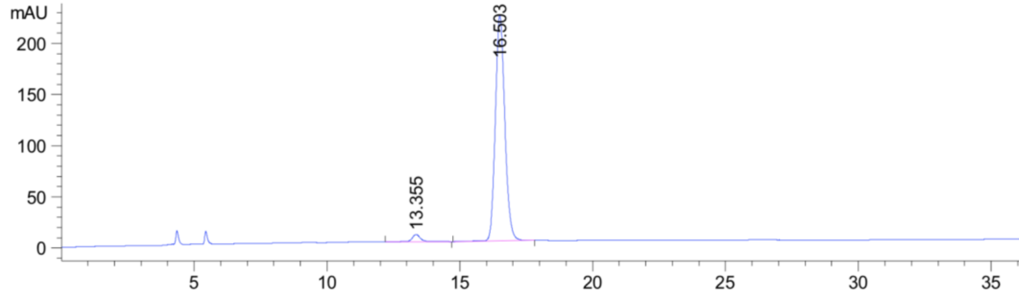

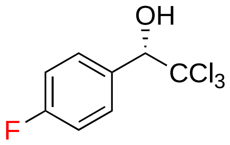

(*S*)-1b

Peak #	RetTime [min]	Type	Width [min]	Area [mAU*s]	Height [mAU]	Area %
1	13.355	BB	0.3430	158.56972	6.92917	2.8144
2	16.503	BB	0.3853	5475.70508	220.84013	97.1856

NMR data.

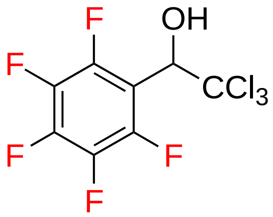

Compound 1a

1-perfluorophenyl-2,2,2-trichloro-ethanol

Colorless crystals in 96% yield (4.54 g, 14.4 mmol).

^{1}H NMR (400 MHz, $CDCl_3$): δ = 5.58 (d, *J* = 7.8 Hz, 1H), 3.72 (dt, *J* = 9.8, 4.0 Hz, 1H);

^{13}C NMR (100 MHz, $CDCl_3$, only nonaromatic carbons reported): δ = 101.08, 79.41 (q, *J* = 1.7 Hz);

^{19}F NMR (377 MHz, $CDCl_3$): δ = -137.06 (s, 2F), -151.61 (tt, *J* = 21.0, 3.8 Hz, 1F), -161.69 (s, 2F).

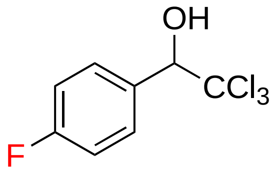

Compound 1b

1-(4-fluorophenyl)-2,2,2-trichloro-ethanol

Colorless oil in 90% yield (3.27 g, 13.5 mmol).

^{1}H NMR (400 MHz, $CDCl_3$): δ = 7.62-7.58 (m, 2H), 7.11-7.06 (m, 2H), 5.20 (s, 1H), 3.49 (s, 1H);

^{13}C NMR (100 MHz, $CDCl_3$): δ = 163.59 (d, *J* = 248.7 Hz), 131.24 (d, *J* = 8.4 Hz), 130.78 (d, *J* = 3.3 Hz), 115.04 (d, *J* = 21.7 Hz), 103.22 (d, *J* = 2.1 Hz), 84.01;

^{19}F NMR (377 MHz, $CDCl_3$): δ = -112.72 (s, 1F).

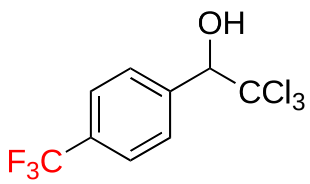

Compound 1c

1-(4-(trifluoromethyl)phenyl)-2,2,2-trichloro-ethanol

Colorless crystals in 95% yield (4.20 g, 14.3 mmol).

^{1}H NMR (400 MHz, $CDCl_3$): δ = 7.76 (d, *J* = 8.2 Hz, 2H), 7.66 (d, *J* = 8.2 Hz, 2H), 5.27 (d, *J* = 2.9 Hz, 1H), 3.54 (d, *J* = 3.8 Hz, 1H);

^{13}C NMR (100 MHz, $CDCl_3$): δ = 138.70 (d, *J* = 1.5 Hz), 131.74 (q, *J* = 32.6 Hz), 129.93, 124.93 (q, *J* = 3.8 Hz), 124.10 (q, *J* = 272.3 Hz), 102.64, 84.03;

^{19}F NMR (377 MHz, $CDCl_3$): δ = -63.72 (s, 3F).

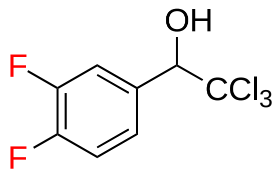

Compound 1d

1-(3,4-difluorophenyl)-2,2,2-trichloro-ethanol

Colorless oil in 95% yield (3.74 g, 14.3 mmol).

^{1}H NMR (400 MHz, $CDCl_3$): δ = 7.50 (ddd, *J* = 11.3, 7.6, 2.2 Hz, 1H), 7.35 (ddt, *J* = 8.1, 3.9, 1.8 Hz, 1H), 7.18 (dt, *J* = 10.0, 8.3 Hz, 1H), 5.19 (s, 1H), 3.44-3.19 (m, 1H);

^{13}C NMR (100 MHz, $CDCl_3$): δ = 151.24 (dd, *J* = 250.0, 13.0 Hz), 149.99 (dd, *J* = 246.0, 12.0 Hz), 131.17 (dd, *J* = 5.7, 3.9 Hz), 125.94 (dd, *J* = 6.7, 3.8 Hz), 118.58 (d, *J* = 18.7 Hz), 116.79 (d, *J* = 17.6 Hz), 102.82, 83.54 (d, *J* = 1.6 Hz);

^{19}F NMR (377 MHz, $CDCl_3$): δ = -137.14 (d, *J* = 21.1 Hz, 1F), -138.30 (d, *J* = 21.2 Hz, 1F).

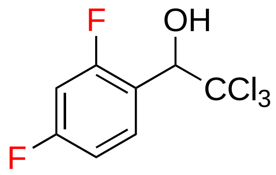

Compound 1e

1-(2,4-difluorophenyl)-2,2,2-trichloro-ethanol

Colorless crystals in 92% yield (3.61 g, 13.8 mmol).

^{1}H NMR (400 MHz, $CDCl_3$): δ = 7.79 (td, *J* = 8.4, 6.4 Hz, 1H), 6.97 (tdd, *J* = 7.9, 2.6, 1.1 Hz, 1H), 6.91-6.82 (m, 1H), 5.57 (s, 1H), 3.31 (d, *J* = 2.0 Hz);

^{13}C NMR (100 MHz, $CDCl_3$): δ = 163.77 (dd, *J* = 250.0, 13.0 Hz), 161.03 (dd, *J* = 250.0, 12.0 Hz), 131.10 (dd, *J* = 10.0, 4.2 Hz), 119.10 (dd, *J* = 12.1, 4.0 Hz), 111.60 (dd, *J* = 21.5, 3.6 Hz), 103.80 (dd, *J* = 26.4, 25.5 Hz), 102.72 (t, *J* = 2.2 Hz), 77.20 (d, *J* = 1.8 Hz);

^{19}F NMR (377 MHz, $CDCl_3$): δ = -108.64 (d, *J* = 8.9 Hz, 1F), -110.50 (d, *J* = 8.9 Hz, 1F).

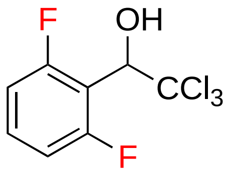

Compound 1f

1-(2,6-difluorophenyl)-2,2,2-trichloro-ethanol

Colorless crystals in 90% yield (3.53 g, 13.5 mmol).

^{1}H NMR (400 MHz, $CDCl_3$): δ = 7.39 (tt, *J* = 8.5, 6.3 Hz, 1H), 7.04-6.90 (m, 2H), 5.58 (d, *J* = 11.4 Hz, 1H), 3.92 (dt, *J* = 11.5, 5.0 Hz, 1H);

^{13}C NMR (100 MHz, $CDCl_3$): δ = 161.38 (dd, *J* = 251.4, 6.2 Hz), 131.79 (t, *J* = 11.2 Hz), 112.44, 112.43 (d, *J* = 24.0 Hz), 102.12 (d, *J* = 2.1 Hz), 79.52;

^{19}F NMR (377 MHz, $CDCl_3$): δ = -109.41 (s, 2F).

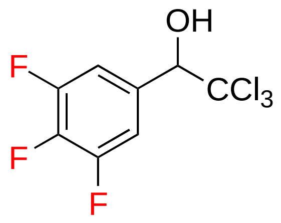

Compound 1g

1-(3,4,5-trifluorophenyl)-2,2,2-trichloro-ethanol

Colorless oil in 95% yield (4.00 g, 14.3 mmol).

^{1}H NMR (400 MHz, $CDCl_3$): δ = 7.50-7.06 (m, 2H), 5.15 (d, *J* = 3.5 Hz, 1H), 3.48 (d, *J* = 3.6 Hz, 1H); ^{13}C NMR (100 MHz, $CDCl_3$): δ = 150.77 (ddd, *J* = 250.1, 10.1, 3.8Hz), 140.61 (dt, *J* = 254.3, 15.2 Hz), 130.88 (td, *J* = 7.6, 4.7 Hz), 115.10=112.15 (m), 102.36, 83.13 (d, *J* = 2.1 Hz);

^{19}F NMR (377 MHz, $CDCl_3$): δ = -134.05 (d, *J* = 20.5 Hz, 2F), -158.51 (t, *J* = 20.5 Hz, 1F).

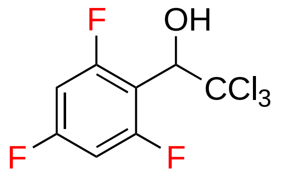

Compound 1h

1-(2,4,6-trifluorophenyl)-2,2,2-trichloro-ethanol

Colorless crystals in 97% yield (4.08 g, 14.6 mmol).

^{1}H NMR (400 MHz, $CDCl_3$): δ = 6.80-6.69 (m, 2H), 5.52 (d, *J* = 10.8 Hz, 1H), 3.81 (dt, *J* = 11.0, 3.9 Hz, 1H);

^{13}C NMR (100 MHz, $CDCl_3$): δ = 163.57 (dt, *J* = 253.5, 16.6 Hz), 161.80 (dt, *J* = 255.3, 12.2 Hz), 108.99 (td, *J* = 14.9, 5.1 Hz), 101.99 (d, *J* = 2.0 Hz), 101.41 (t, *J* = 27.2 Hz), 79.27 (, *J* = Hz);

^{19}F NMR (377 MHz, $CDCl_3$): δ = -104.91 (t, *J* = 8.2 Hz, 1F), -105.07 (s, 2F).

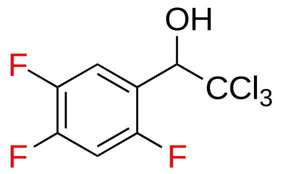

Compound 1i

1-(2,4,5-trifluorophenyl)-2,2,2-trichloro-ethanol

Colorless crystals in 93% yield (3.91 g, 14.0 mmol).

^{1}H NMR (400 MHz, $CDCl_3$): δ = 7.62 (ddd, *J* = 10.8, 8.8, 6.3 Hz, 1H), 6.96 (td, *J* = 9.6, 6.4 Hz, 1H), 5.55 (s, 1H), 3.42 (s, 1H);

^{13}C NMR (100 MHz, $CDCl_3$): δ = 156.05 (ddd, *J* = 248.3, 9.7, 2.7 Hz), 151.12 (ddd, *J* = 254.4, 14.5, 12.7 Hz), 146.93 (ddd, *J* = 245.5, 12.6, 3.5 Hz), 119.33 (ddd, *J* = 14.3, 5.5, 4.3 Hz), 118.11 (ddd, *J* = 20.8, 4.2, 1.6 Hz), 105.51 (dd, *J* = 28.7, 21.0 Hz), 102.32, 76.85;

^{19}F NMR (377 MHz, $CDCl_3$): δ = -115.74 (dd, *J* = 15.1, 5.3 Hz, 1F), -131.79 (dd, *J* = 21.5, 5.3 Hz, 1F), -142.46 (dd, *J* = 21.7, 15.0 Hz, 1F).

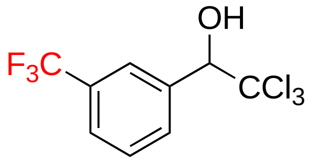

Compound 1j

1-(3-(trifluoromethyl)phenyl)-2,2,2-trichloro-ethanol

Colorless liquid in 91% yield (4.02 g, 13.7 mmol).

^{1}H NMR (400 MHz, $CDCl_3$): δ = 7.91 (s, 1H), 7.82 (d, *J* = 7.9 Hz, 1H), 7.68 (d, *J* = 7.9 Hz, 1H), 7.52 (t, *J* = 7.8 Hz, 1H), 5.28 (d, *J* = 3.6 Hz, 1H), 3.42 (d, *J* = 3.7 Hz, 1H);

^{13}C NMR (100 MHz, $CDCl_3$): δ = 135.82, 132.86 (d, *J* = 1.4 Hz), 130.54 (q, *J* = 32.6 Hz), 128.48, 126.49 (q, *J* = 3.8 Hz), 126.38 (q, *J* = 4.0 Hz), 124.13 (q, *J* = 272.3 Hz), 102.72, 84.05;

^{19}F NMR (377 MHz, $CDCl_3$): δ = -63.61 (s, 3F).

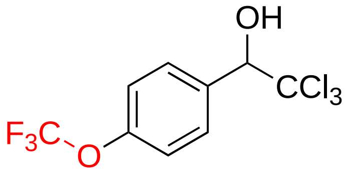

Compound 1k

1-(4-(trifluoromethoxy)phenyl)-2,2,2-trichloro-ethanol

Colorless oil in 97% yield (4.52 g, 14.6 mmol).

^{1}H NMR (400 MHz, $CDCl_3$): δ = 7.68-7.65 (m, 2H), 7.25-7.22 (m, 2H), 5.23 (s, 1H), 3.42 (s, 1H);

^{13}C NMR (100 MHz, $CDCl_3$): δ = 150.19 (q, *J* = 2.0 Hz), 133.43, 131.03, 120.61 (q, *J* = 256.0 Hz), 120.27, 102.98, 83.93;

^{19}F NMR (377 MHz, $CDCl_3$): δ = -58.73 (s, 3F).

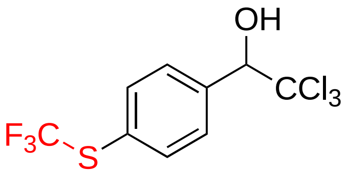

Compound 1l

1-(4-(trifluoromethyl)thio)phenyl)-2,2,2-trichloro-ethanol

Colorless crystals in 88% yield (4.30 g, 13.2 mmol).

^{1}H NMR (400 MHz, $CDCl_3$): δ = 7.69 (s, 4H), 5.26 (d, *J* = 3.7 Hz, 1H), 3.36 (d, *J* = 3.8 Hz, 1H);

^{13}C NMR (100 MHz, $CDCl_3$): δ = 137.68, 135.62, 130.55, 129.67 (q, *J* = 306.0 Hz), 126.09 (q, *J* = 2.1 Hz), 102.70, 84.06;

^{19}F NMR (377 MHz, $CDCl_3$): δ = -43.28 (s, 3F).

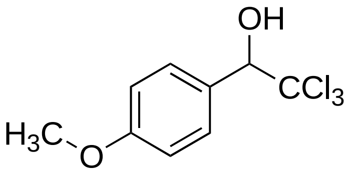

Compound 1m

1-(4-methoxyphenyl)-2,2,2-trichloro-ethanol

Colorless crystals in 81% yield (3.12 g, 12.2 mmol).

^{1}H NMR (400 MHz, $CDCl_3$): δ = 7.60-7.49 (m, 2H), 6.96-6.87 (m, 2H), 5.17 (s, 1H), 3.83 (s, 3H), 3.28 (s, 1H);

^{13}C NMR (100 MHz, $CDCl_3$): δ = 160.62, 130.60, 127.10, 113.45, 103.72, 84.40, 55.49.

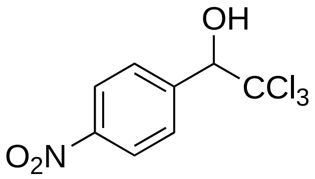

Compound 1n

1-(4-nitrophenyl)-2,2,2-trichloro-ethanol

Yellow crystals in 85% yield (3.46 g, 12.8 mmol).

^{1}H NMR (400 MHz, $CDCl_3$): δ = 8.29-8.20 (m, 2H), 7.89-7.79 (m, 2H), 5.34 (s, 1H), 3.57 (s, 1H);

^{13}C NMR (100 MHz, $CDCl_3$): δ = 148.72, 141.58, 130.60, 123.05, 102.55, 83.65.

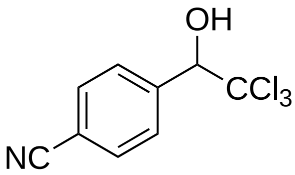

Compound 1o

1-(4-cyanophenyl)-2,2,2-trichloro-ethanol

Colorless crystals in 85% yield (3.21 g, 12.8 mmol).

^{1}H NMR (400 MHz, $CDCl_3$): δ = 7.80-7.73 (m, 2H), 7.72-7.65 (m, 2H), 5.27 (d, *J* = 3.0 Hz, 1H), 3.64 (d, *J* = 3.7 Hz, 1H);

^{13}C NMR (100 MHz, $CDCl_3$): δ = 139.96, 131.69, 130.31, 118.56, 113.40, 102.33, 83.78.

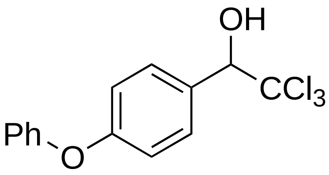

Compound 1p

1-(4-phenoxyphenyl)-2,2,2-trichloro-ethanol

Colorless crystals in 80% yield (3.81 g, 12.0 mmol).

^{1}H NMR (400 MHz, $CDCl_3$): δ = 7.59-7.54 (m, 2H), 7.40-7.33 (m, 2H), 7.18-7.12(m, 1H), 7.08-7.03 (m, 2H), 7.02-6.97 (m, 2H), 5.21 (d, *J* = 3.7 Hz, 1H), 3.29 (d, *J* = 3.9 Hz, 1H);

^{13}C NMR (100 MHz, $CDCl_3$): δ = 158.76, 156.61, 130.87, 130.08, 129.35, 124.07, 119.77, 117.71, 103.50, 84.32.

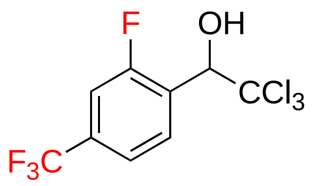

Compound 1q

1-(2-fluoro-4-(trifluoromethyl)phenyl)-2,2,2-trichloro-ethanol

Colorless crystals in 90% yield (4.20 g, 13.5 mmol).

^{1}H NMR (400 MHz, $CDCl_3$): δ = 7.94 (t, J = 7.4 Hz, 1H), 7.50 (dd, J = 8.2, 1.6 Hz, 1H), 7.37 (dd, J = 9.9, 1.7 Hz, 1H), 5.66 (d, J = 4.6 Hz, 1H), 3.40 (d, J = 4.6 Hz, 1H);

^{13}C NMR (100 MHz, $CDCl_3$): δ = 160.43 (d, J = 251.6 Hz), 133.67 (qd, J = 33.6, 8.5 Hz), 131.05 (d, J = 2.9 Hz), 126.81 (d, J = 11.1 Hz), 123.22 (qd, J = 272.7, 2.8 Hz), 121.02 (p, J = 3.8 Hz), 113.09 (dq, J = 26.0, 3.9 Hz), 102.18, 77.20 (d, J = 1.8 Hz);

^{19}F NMR (377 MHz, $CDCl_3$): δ = -64.01 (s, 3F), -111.84 (s, 1F).

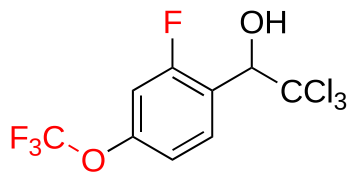

Compound 1r

1-(2-fluoro-4-(trifluoromethoxy)phenyl)-2,2,2-trichloro-ethanol

Colorless oil in 90% yield (4.42 g, 13.5 mmol).

^{1}H NMR (400 MHz, $CDCl_3$): δ = 7.83 (t, J = 8.2 Hz, 1H), 7.09 (dq, J = 7.7, 1.2 Hz, 1H), 6.99 (ddd, J = 10.4, 2.3, 1.1 Hz, 1H), 5.60 (d, J = 4.2 Hz, 1H), 3.54 (d, J = 4.4 Hz, 1H);

^{13}C NMR (100 MHz, $CDCl_3$): δ = 160.81 (d, J = 251.9 Hz), 150.79 (dq, J = 11.4, 1.7 Hz), 133.55-128.84 (m), 121.60 (d, J = 12.1 Hz), 120.49 (q, J = 258.9 Hz), 116.34 (d, J = 3.6 Hz), 108.53 (dd, J = 26.8, 1.5 Hz), 102.47 (d, J = 2.5 Hz), 77.19 (d, J = 1.7 Hz);

^{19}F NMR (377 MHz, $CDCl_3$): δ = -58.98 (s, 3F), -110.56 (s, 1F).

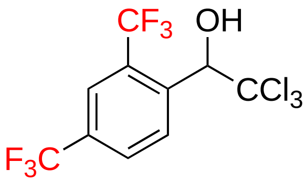

Compound 1s

1-(2,4-bis(trifluoromethyl)phenyl)-2,2,2-trichloro-ethanol

Colorless oil in 95% yield (5.17 g, 14.3 mmol).

^{1}H NMR (400 MHz, $CDCl_3$): δ = 8.36 (d, *J* = 8.4 Hz, 1H), 7.98 (d, *J* = 1.8 Hz, 1H), 7.90 (dd, *J* = 8.4, 1.8 Hz, 1H), 5.81 (d, *J* = 4.3 Hz, 1H), 3.46 (d, *J* = 4.6 Hz, 1H);

^{13}C NMR (100 MHz, $CDCl_3$): δ = 138.34, 132.28 (q, *J* = 33.8 Hz), 130.78 (q, *J* = 30.7 Hz), 130.44, 128.80 (d, *J* = 3.9 Hz), 123.60 (td, *J* = 8.5, 7.7, 4.9 Hz), 123.45 (q, *J* = 274.8 Hz), 123.26 (q, *J* = 272.6 Hz), 101.73, 78.95 (q, *J* = 3.0 Hz);

^{19}F NMR (377 MHz, $CDCl_3$): δ = -57.21 (s, 3F), -64.71 (s, 3F).

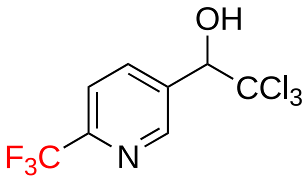

Compound 1t

1-(6-(trifluoromethyl)pyridin-3-yl)-2,2,2-trichloro-ethanol

Colorless crystals in 97% yield (4.30 g, 14.6 mmol).

^{1}H NMR (400 MHz, $CDCl_3$): δ = 8.94 (d, *J* = 2.1 Hz, 1H), 8.20 (dd, *J* = 8.2, 2.1 Hz, 1H), 7.74 (dd, *J* = 8.2, 0.8 Hz, 1H), 5.36 (d, *J* = 3.2 Hz, 1H), 3.69 (d, *J* = 3.7 Hz, 1H);

^{13}C NMR (100 MHz, $CDCl_3$): δ = 150.88, 149.03 (d, *J* = 34.9 Hz), 138.55, 133.99, 121.53 (q, *J* = 274.4 Hz), 119.94 (q, *J* = 2.7 Hz), 102.14, 82.22;

^{19}F NMR (377 MHz, $CDCl_3$): δ = -68.89 (s, 3F).

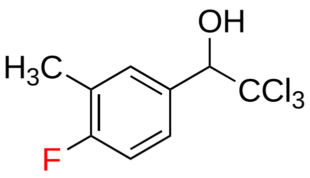

Compound 1u

1-(4-fluoro-3-methylphenyl)-2,2,2-trichloro-ethanol

Colorless crystals in 80% yield (3.09 g, 12.0 mmol).

^{1}H NMR (400 MHz, $CDCl_3$): δ = 7.45 (d, J = 7.4 Hz, 1H), 7.41 (dd, J = 9.0, 4.7 Hz, 1H), 7.02 (t, J = 8.9 Hz, 1H), 5.17 (s, 1H), 3.29 (s, 1H), 2.31 (s, 3H);

^{13}C NMR (100 MHz, $CDCl_3$): δ = 162.12 (d, J = 247.5 Hz), 132.48 (d, J = 5.6 Hz), 130.39 (d, J = 3.7 Hz), 128.58 (d, J = 8.4 Hz), 124.67 (d, J = 17.6 Hz), 114.66 (d, J = 22.8 Hz), 103.33 (d, J = 2.0 Hz), 84.17, 14.87 (d, J = 3.6 Hz);

^{19}F NMR (377 MHz, $CDCl_3$): δ = 116.98 (s, 1F).

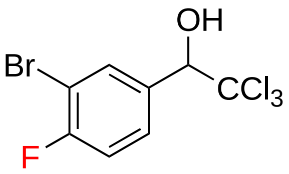

Compound 1v

1-(3-bromo-4-fluorophenyl)-2,2,2-trichloro-ethanol

Colorless crystals in 90% yield (4.35 g, 13.5 mmol).

^{1}H NMR (400 MHz, $CDCl_3$): δ = 7.84 (dd, J = 6.5, 2.2 Hz, 1H), 7.55 (ddd, J = 8.6, 4.6, 2.2 Hz, 1H), 7.14 (t, J = 8.4 Hz, 1H), 5.18 (d, J = 3.4 Hz, 1H), 3.42 (d, J = 3.6 Hz, 1H);

^{13}C NMR (100 MHz, $CDCl_3$): δ = 159.85 (d, J = 250.1 Hz), 134.56, 132.22 (d, J = 3.9 Hz), 130.26 (d, J = 7.7 Hz), 115.99 (d, J = 22.7 Hz), 108.78 (d, J = 21.3 Hz), 102.82 (d, J = 2.0 Hz), 83.37;

^{19}F NMR (377 MHz, $CDCl_3$): δ = -106.76 (s, 1F).

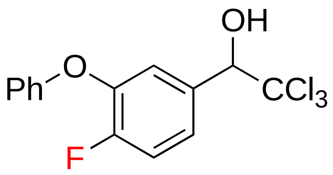

Compound 1w

1-(4-fluoro-3-phenoxyphenyl)-2,2,2-trichloro-ethanol

Colorless crystals in 85% yield (4.30 g, 12.8 mmol).

^{1}H NMR (400 MHz, $CDCl_3$): δ = 7.45-7.29 (m, 4H), 7.20 (dd, *J* = 10.2, 8.4 Hz, 1H), 7.15-7.06 (m, 1H), 7.04-6.94 (m, 2H), 5.15 (d, *J* = 2.8 Hz, 1H), 3.38 (d, *J* = 3.4 Hz, 1H);

^{13}C NMR (100 MHz, $CDCl_3$): δ = 157.32, 155.03 (d, *J* = 251.7 Hz), 143.33 (d, *J* = 11.8 Hz), 131.63 (d, *J* = 3.7 Hz), 129.98, 125.82 (d, *J* = 7.3 Hz), 123.58, 123.07 (d, *J* = 1.8 Hz), 117.52, 116.60 (d, *J* = 18.9 Hz), 103.07 (d, *J* = 2.2 Hz), 83.77;

^{19}F NMR (377 MHz, $CDCl_3$): δ = -130.32 (s, 1F).

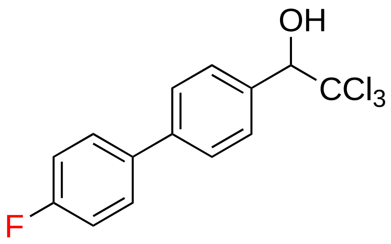

Compound 1x

1-(4'-fluoro-[1,1'-biphenyl]-4-yl)-2,2,2-trichloro-ethanol

Colorless crystals in 85% yield (4.09 g, 12.8 mmol).

^{1}H NMR (400 MHz, $CDCl_3$): δ = 7.71-7.67 (m, 2H), 7.56 (ddd, *J* = 8.8, 3.8, 2.1 Hz, 4H), 7.17-7.10 (m, 2H), 5.27 (d, *J* = 3.8 Hz, 1H), 3.34 (d, *J* = 4.0 Hz, 1H);

^{13}C NMR (100 MHz, $CDCl_3$): δ = 162.87 (d, *J* = 246.9 Hz), 141.55, 136.73 (d, *J* = 3.3 Hz), 133.95, 129.90, 128.97 (d, *J* = 8.1 Hz), 126.60, 115.95 (d, *J* = 21.4 Hz), 103.32, 84.51;

^{19}F NMR (377 MHz, $CDCl_3$): δ = 116.09 (s, 1F).

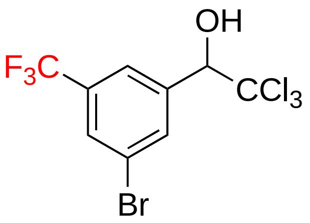

Compound 1y

1-(3-bromo-5-(trifluoromethyl)phenyl)-2,2,2-trichloro-ethanol

Colorless crystals in 85% yield (4.77 g, 12.8 mmol).

^{1}H NMR (400 MHz, $CDCl_3$): δ = 7.98 (d, *J* = 1.8 Hz, 1H), 7.85-7.78 (m, 2H), 5.24 (s, 1H), 3.50 (s, 1H); ^{13}C NMR (100 MHz, $CDCl_3$): δ = 137.85, 135.84, 132.07 (q, *J* = 33.3 Hz), 129.68 (q, *J* = 3.8 Hz), 125.23 (q, *J* = 3.8 Hz), 123.15 (q, *J* = 273.0 Hz), 122.31, 102.22, 83.35;

^{19}F NMR (377 MHz, $CDCl_3$): δ = -63.75 (s, 3F).

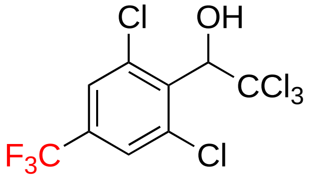

Compound 1z

1-(2,6-dichloro-4-(trifluoromethyl)phenyl)-2,2,2-trichloro-ethanol

Colorless crystals in 80% yield (4.35 g, 12.0 mmol).

^{1}H NMR (400 MHz, $CDCl_3$): δ = 7.68 (d, *J* = 1.8 Hz, 1H), 7.62 (d, *J* = 1.8 Hz, 1H), 6.20 (d, *J* = 11.7 Hz, 1H), 4.53 (d, *J* = 11.7 Hz, 1H);

^{13}C NMR (100 MHz, $CDCl_3$): δ = 139.44, 135.05, 134.40, 133.12, (q, *J* = 34.5 Hz), 128.12 (q, *J* = 3.7 Hz), 126.31 (q, *J* = 3.7 Hz), 122.30 (q, *J* = 272 Hz), 101.64, 83.44;

^{19}F NMR (377 MHz, $CDCl_3$): δ = -64.37 (s, 3F).

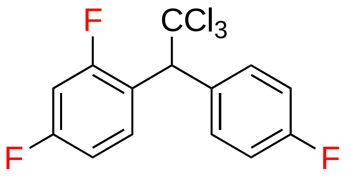

Compound 2a

***o*-F-DFDT (1,1,1-trichloro-2,2-(4-fluorophenyl)-(2,4-difluorophenyl)-ethane)**

Colorless liquid in 65% yield (2.65 g, 7.80 mmol).

^{1}H-NMR (400 MHz, $CDCl_3$): δ = 7.97 (td, *J* = 8.7, 6.2 Hz, 1H), 7.62-7.54 (m, 2H), 7.10 - 7.02 (m, 2H), 6.94 (dddd, *J* = 9.0, 7.8, 2.7, 1.3 Hz, 1H), 6.84 (ddd, *J* = 10.4, 8.6, 2.7 Hz, 1H), 5.49 (s, 1H);

^{13}C-NMR (100 MHz, $CDCl_3$): δ = 162.71 (d, *J* = 248.4 Hz), 161.79 (dd, *J* = 250.0, 188.0 Hz), 161.68 (dd, *J* = 249.0, 186.0 Hz), 132.82 (d, *J* = 3.4 Hz), 132.30 (d, *J* = 8.2 Hz), 130.61 (dd, *J* = 9.7, 3.7 Hz), 121.11 (dd, *J* = 12.6, 4.1 Hz), 115.57 (d, *J* = 21.5 Hz), 111.59 (dd, *J* = 21.2, 3.8 Hz), 104.32 (dd, *J* = 27.2, 25.2 Hz), 101.09, 60.66;

^{19}F-NMR (377 MHz, $CDCl_3$): δ = -110.47 (d, *J* = 8.7 Hz, 1F), -112.13 (d, *J* = 8.5 Hz, 1F), -114.19 (s, 1F).

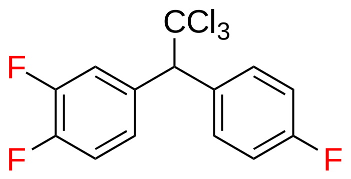

Compound 2b

***m*-F-DFDT (1,1,1-trichloro-2,2-(4-fluorophenyl)-(bis(3,4-difluorophenyl)-ethane)**

Colorless liquid in 65% yield (2.65 g, 7.80 mmol).

^{1}H-NMR (400 MHz, $CDCl_3$): δ = 7.59 - 7.51 (m, 2H), 7.46 (ddd, *J* = 11.6, 7.5, 2.4 Hz, 1H), 7.30 (ddt, *J* = 8.2, 3.9, 1.8 Hz, 1H), 7.14 (dt, *J* = 10.0, 8.4 Hz, 1H), 7.10 - 7.02 (m, 2H), 5.02 (s, 1H);

^{13}C-NMR (100 MHz, $CDCl_3$): δ = 162.69 (d, *J* = 248.6 Hz), 151.43 (dd, *J* = 12.7, 10.9 Hz), 148.95 (dd, *J* = 12.6, 9.0 Hz), 135.08 – 134.90 (m), 133.40 (d, *J* = 3.4 Hz), 131.89 (d, *J* = 8.1 Hz), 126.55 (dd, *J* = 6.3, 3.7 Hz), 119.15 (d, *J* = 18.5 Hz), 117.38 (d, *J* = 17.3 Hz), 115.72 (d, *J* = 21.6 Hz), 101.04, 69.36 (d, *J* = 1.3 Hz);

^{19}F-NMR (377 MHz, $CDCl_3$): δ = -141.22 (s, 1F), -137.56 (d, *J* = 21.3 Hz, 1F), -139.00 (d, *J* = 21.3 Hz, 1F).

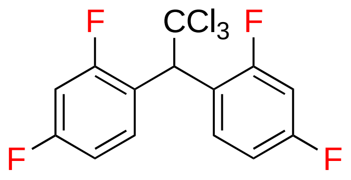

Compound 2c

***o,o', p,p'*-TFDT (1,1,1-trichloro-2,2-bis(2,4-difluorophenyl)-ethane)**

Colorless crystals in 60% yield (2.56 g, 7.20 mmol).

^{1}H-NMR (400 MHz, $CDCl_3$): δ = 7.85 (td, *J* = 8.6, 6.2 Hz, 2H), 6.92 (dddd, *J* = 8.9, 7.8, 2.7, 1.3 Hz, 2H), 6.85 (ddd, *J* = 10.3, 8.6, 2.7 Hz, 2H), 5.90 (s, 1H);

^{13}C-NMR (100 MHz, $CDCl_3$): δ = 162.06 (dd, *J* = 250.0, 175.0 Hz), 161.94 (dd, *J* = 249.0, 173.0 Hz), 131.10 (dd, *J* = 9.7, 3.7 Hz), 121.02 (dd, *J* = 12.8, 4.0 Hz), 111.53 (dd, *J* = 21.3, 3.7 Hz), 104.37 (dd, *J* = 27.1, 25.3 Hz), 100.52, 52.47 (t, *J* = 3.57 Hz);

^{19}F-NMR (377 MHz, $CDCl_3$): δ = -110.03 (d, *J* = 8.9 Hz, 2F), -111.11 (d, *J* = 8.8 Hz, 2F).

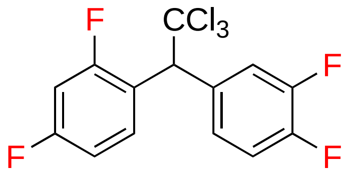

Compound 2d

***o,m′,p,p′*-TFDT (1,1,1-trichloro-2,2-(2,4-difluorophenyl)-(3,4-difluorophenyl)-ethane)**

Colorless crystals in 70% yield (3.00 g, 8.40 mmol).

^{1}H-NMR (400 MHz, $CDCl_3$): δ = 7.91 (td, *J* = 8.7, 6.2 Hz, 1H), 7.45 (ddd, *J* = 11.5, 7.5, 2.4 Hz, 1H), 7.30 (ddt, *J* = 8.1, 3.9, 1.8 Hz, 1H), 7.14 (dt, *J* = 10.0, 8.4 Hz, 1H), 6.95 (dddd, *J* = 9.0, 7.8, 2.7, 1.3 Hz, 1H), 6.85 (ddd, *J* = 10.9, 8.6, 2.7 Hz, 1H), 5.44 (s, 1H);

^{13}C-NMR (100 MHz, $CDCl_3$): δ = 151.30 (dd, *J* = 32.7, 12.5 Hz), 148.82 (dd, *J* = 30.6, 12.5 Hz), 135.67 - 132.95 (m), 130.33 (dd, *J* = 9.7, 3.6 Hz), 126.80 (dd, *J* = 6.5, 3.6 Hz), 121.34 (dd, *J* = 12.5, 4.2 Hz), 119.30 (d, *J* = 18.6 Hz), 117.16 (d, *J* = 17.3 Hz), 111.56 (dd, *J* = 21.3, 3.7 Hz), 104.23 (dd, *J* = 27.2, 25.3 Hz), 100.33, 60.25 (dd, *J* = 3.8, 1.5 Hz);

^{19}F-NMR (377 MHz, $CDCl_3$): δ = -109.97 (d, *J* = 8.8 Hz, 1F), -112.10 (d, *J* = 8.9 Hz, 1F), -137.53 (d, *J* = 21.4 Hz, 1F), -138.52 (d, *J* = 21.4 Hz, 1F).

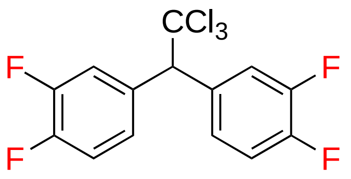

Compound 2e

***m,m′,p,p′*-TFDT (1,1,1-trichloro-2,2-bis(3,4-difluorophenyl)-ethane)**

Colorless liquid in 35% yield (1.50 g, 4.20 mmol).

^{1}H-NMR (400 MHz, $CDCl_3$): δ = 7.44 (ddd, *J* = 11.4, 7.4, 2.4 Hz, 2H), 7.28 (ddd, *J* = 8.5, 4.0, 1.8 Hz, 2H), 7.16 (dt, *J* = 9.9, 8.3 Hz, 2H), 4.99 (s, 1H);

^{13}C-NMR (100 MHz, $CDCl_3$): δ = 151.55 (dd, *J* = 20.8, 12.6 Hz), 149.07 (dd, *J* = 18.9, 12.5 Hz), 138.66 - 132.27 (m), 126.54 (dd, *J* = 6.5, 3.7 Hz), 119.17 (d, *J* = 18.7 Hz), 117.55 (d, *J* = 17.3 Hz), 100.50, 69.06; ^{19}F-NMR (377 MHz, $CDCl_3$): δ = -137.20 (d, *J* = 21.4 Hz, 2F), -138.44 (d, *J* = 21.4 Hz, 2F).

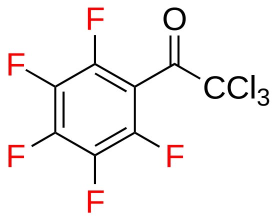

Compound 3a

1-(perfluorophenyl)-2,2,2-trichloro-ethanone

Colorless liquid in 95% yield (6.27 g, 20.0 mmol).

^{13}C NMR (100 MHz, $CDCl_3$, only nonaromatic carbons reported): δ = 177.61, 94.40;

^{19}F NMR (377 MHz, $CDCl_3$): δ = -134.61--134.90 (m, 2F), -148.54 (tt, *J* = 20.6, 3.9 Hz, 1F), -159.78--160.04 (m, 2F).

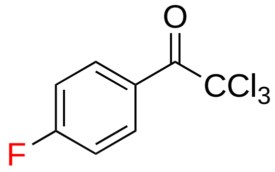

Compound 3b

1-(4-fluorophenyl)-2,2,2-trichloro-ethanone

Colorless liquid in 93% yield (4.71 g, 19.5 mmol).

^{1}H NMR (400 MHz, $CDCl_3$): δ = 8.36-8.26 (m, 2H), 7.21-7.13 (m, 2H);

^{13}C NMR (100 MHz, $CDCl_3$): δ = 179.76, 166.19 (d, *J* = 258.3 Hz), 134.50 (d, *J* = 9.6 Hz), 125.22 (d, *J* = 3.2 Hz), 115.78 (d, *J* = 22.0 Hz), 95.25;

^{19}F NMR (377 MHz, $CDCl_3$): δ = -102.86 (s, 1F).

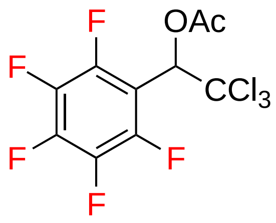

Compound 4

1-(perfluorophenyl)-2,2,2-trichloro-ethyl acetate

Colorless crystals in 91% yield (4.90 g, 13.7 mmol).

^{1}H NMR (400 MHz, $CDCl_3$): δ = 6.64 (s, 1H), 2.25 (s, 3H);

^{13}C NMR (100 MHz, $CDCl_3$, only nonaromatic carbons reported): δ = 168.84, 97.53, 76.56, 20.56;

^{19}F NMR (377 MHz, $CDCl_3$): δ = -135.49 (s, 2F), -150.86 (tt, *J* = 21.1, 4.2 Hz, 1F), -161.68 (s, 2F).

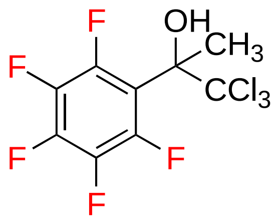

Compound 5

2-(perfluorophenyl)-1,1,1-trichloro-propan-2-ol

Colorless oil in 92% yield (4.55 g, 13.8 mmol).

^{1}H NMR (400 MHz, $CDCl_3$): δ = 4.39-4.23 (m, 1H), 2.12 (t, *J* = 3.3 Hz, 3H);

^{13}C NMR (100 MHz, $CDCl_3$, only nonaromatic carbons reported): δ = 106.74, 86.64, 25.76 (t, *J* = 6.6 Hz);

^{19}F NMR (377 MHz, $CDCl_3$): δ = -128.93 (s, 1F), -137.24 (s, 1F), -151.47 (tt, *J* = 21.4, 5.1Hz, 1F), -160.76 (s, 2F).

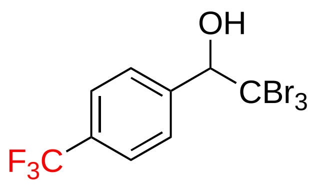

Compound 6

1-(4-(trifluoromethyl)phenyl)-2,2,2-tribromo-ethanol

Colorless crystals in 91% yield (5.85 g, 13.7 mmol).

^{1}H NMR (400 MHz, $CDCl_3$): δ = 7.86 (d, *J* = 8.1 Hz, 2H), 7.65 (d, *J* = 8.2 Hz, 2H), 5.25 (d, *J* = 3.4 Hz, 1H), 3.62 (d, *J* = 3.7 Hz, 1H);

^{13}C NMR (100 MHz, $CDCl_3$): δ = 139.02 (d, *J* = 1.4 Hz), 131.62 (q, *J* = 32.6 Hz), 130.36, 124.75 (q, *J* = 3.8 Hz), 124.13 (q, *J* = 272.3 Hz), 85.25, 53.29;

^{19}F NMR (377 MHz, $CDCl_3$): δ = -63.72 (s, 3F).

NMR spectra.

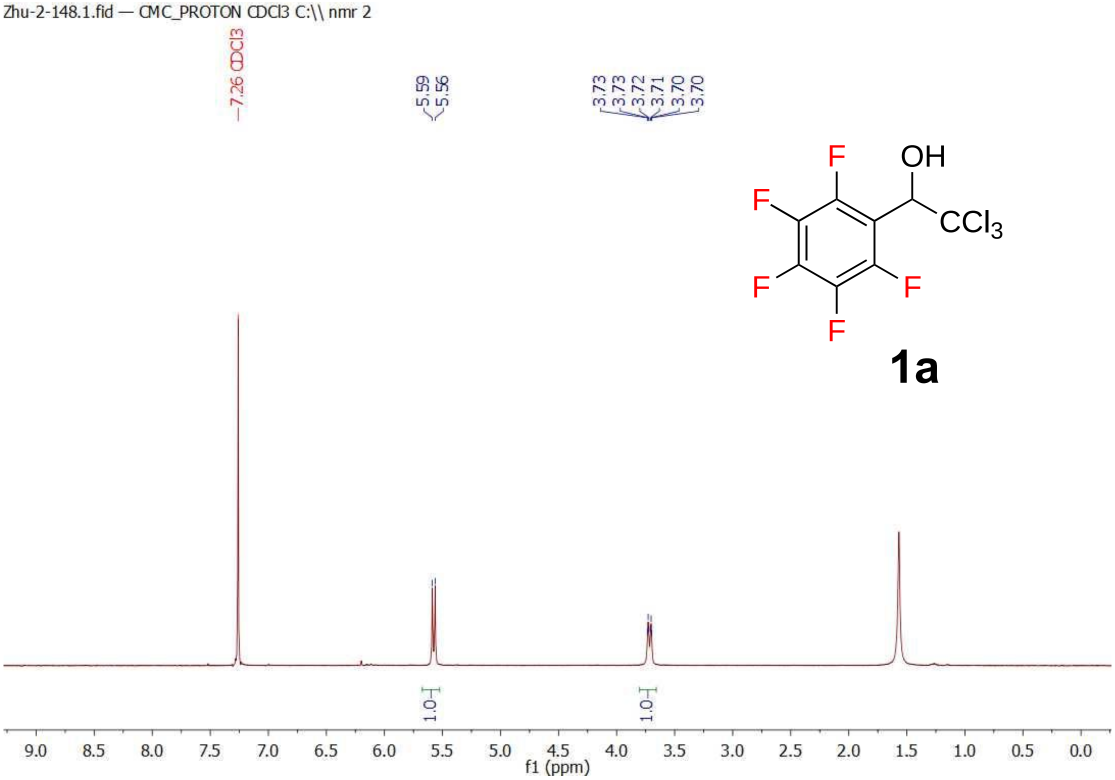
Zhu-2-148.1.fid — CMC_PROTON CDCl3 C:\\ nmr 2
7.26 CDCl3
5.59
5.56
3.73
3.73
3.72
3.71
3.70
3.70
F
F
F
F
F
OH
CCl3
1a
1.0
1.0
9.0 8.5 8.0 7.5 7.0 6.5 6.0 5.5 5.0 4.5 4.0 3.5 3.0 2.5 2.0 1.5 1.0 0.5 0.0
f1 (ppm)

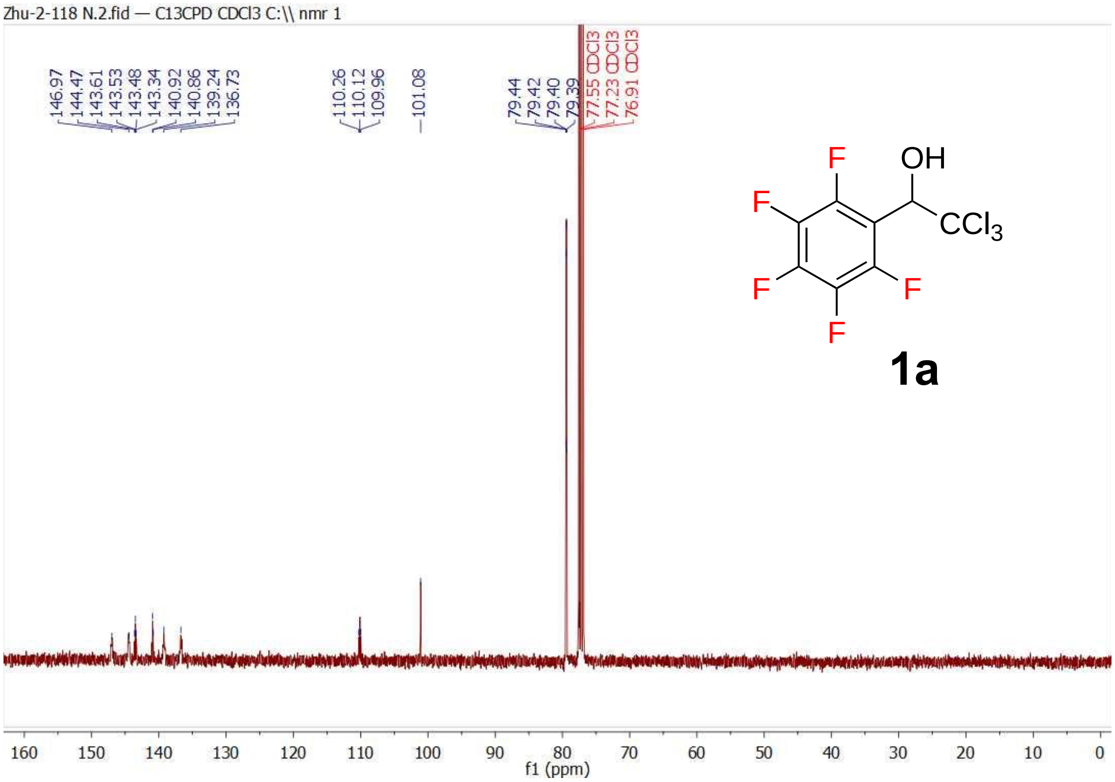
Zhu-2-118 N.2.fid — C13CPD CDCl3 C:\\ nmr 1
146.97
144.47
143.61
143.53
143.48
143.34
140.92
140.86
139.24
136.73
110.26
110.12
109.96
101.08
79.44
79.42
79.40
79.39
77.55 CDCl3
77.23 CDCl3
76.91 CDCl3
F
F
F
F
F
OH
CCl3
1a
160 150 140 130 120 110 100 90 80 70 60 50 40 30 20 10 0
f1 (ppm)

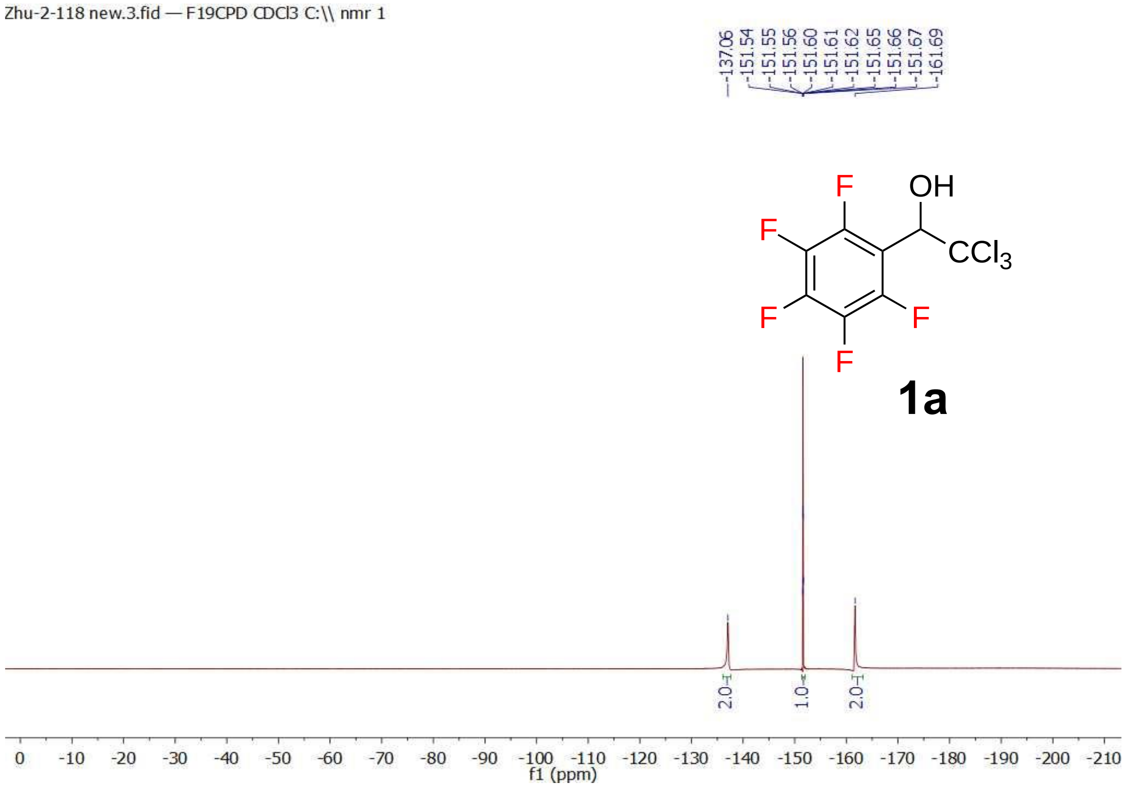
Zhu-2-118 new.3.fid — F19CPD CDCl3 C:\\ nmr 1
-137.06
-151.54
-151.55
-151.56
-151.60
-151.61
-151.62
-151.65
-151.66
-151.67
-161.69
F
OH
F
CCl3
F
F
F
1a
2.0
1.0
2.0
0 -10 -20 -30 -40 -50 -60 -70 -80 -90 -100 -110 -120 -130 -140 -150 -160 -170 -180 -190 -200 -210
f1 (ppm)

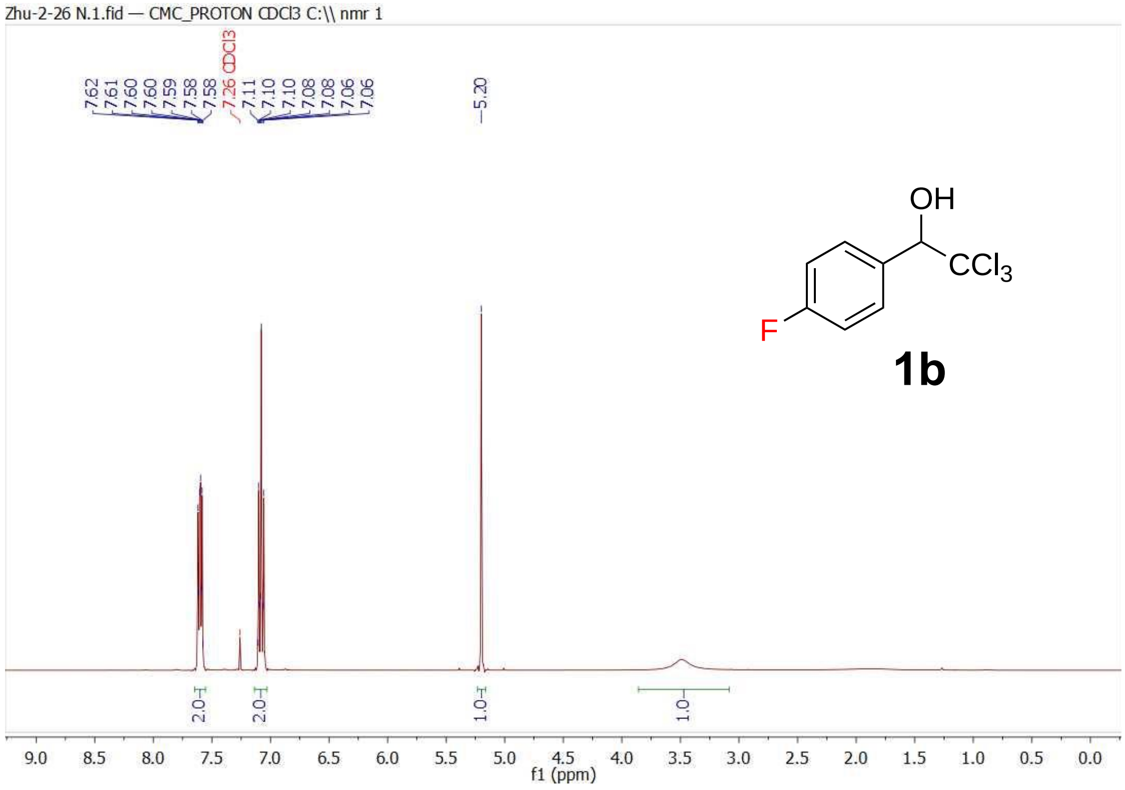
Zhu-2-26 N.1.fid — CMC_PROTON CDCl3 C:\\ nmr 1
7.62
7.61
7.60
7.60
7.59
7.58
7.58
7.26 CDCl3
7.11
7.10
7.10
7.08
7.08
7.06
7.06
5.20
OH
CCl3
F
1b
2.0
2.0
1.0
1.0
9.0 8.5 8.0 7.5 7.0 6.5 6.0 5.5 5.0 4.5 4.0 3.5 3.0 2.5 2.0 1.5 1.0 0.5 0.0
f1 (ppm)

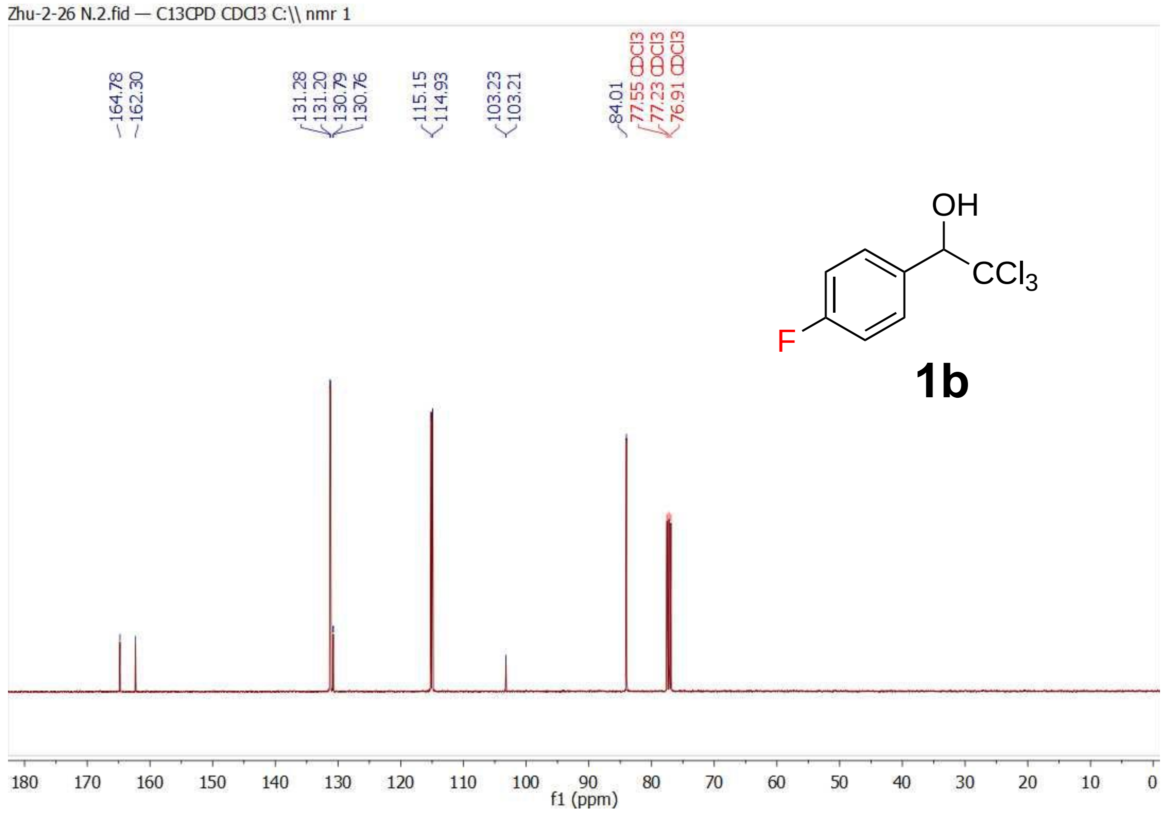
Zhu-2-26 N.2.fid — C13CPD CDCl3 C:\\ nmr 1
164.78
162.30
131.28
131.20
130.79
130.76
115.15
114.93
103.23
103.21
84.01
77.55 CDCl3
77.23 CDCl3
76.91 CDCl3
OH
CCl3
F
1b
180 170 160 150 140 130 120 110 100 90 80 70 60 50 40 30 20 10 0
f1 (ppm)

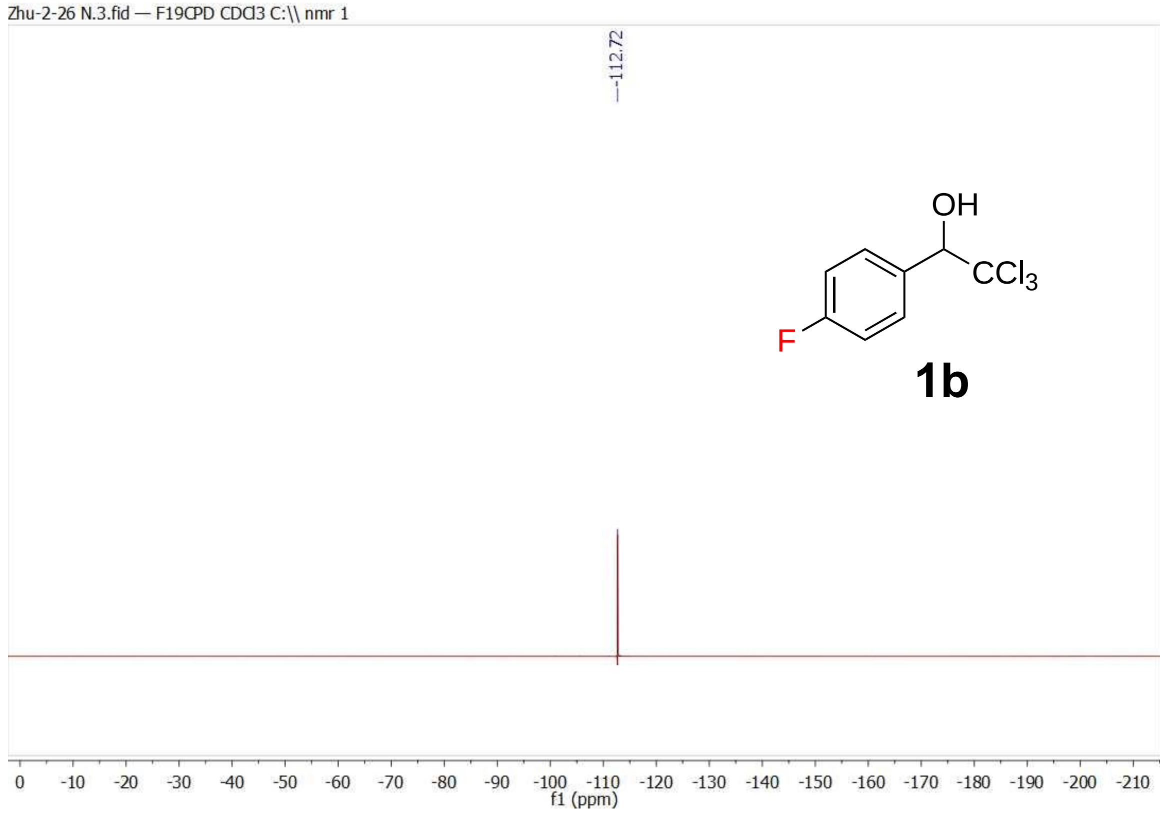
Zhu-2-26 N.3.fid — F19CPD CDCl3 C:\\ nmr 1
-112.72
OH
CCl3
F
1b
0 -10 -20 -30 -40 -50 -60 -70 -80 -90 -100 -110 -120 -130 -140 -150 -160 -170 -180 -190 -200 -210
f1 (ppm)

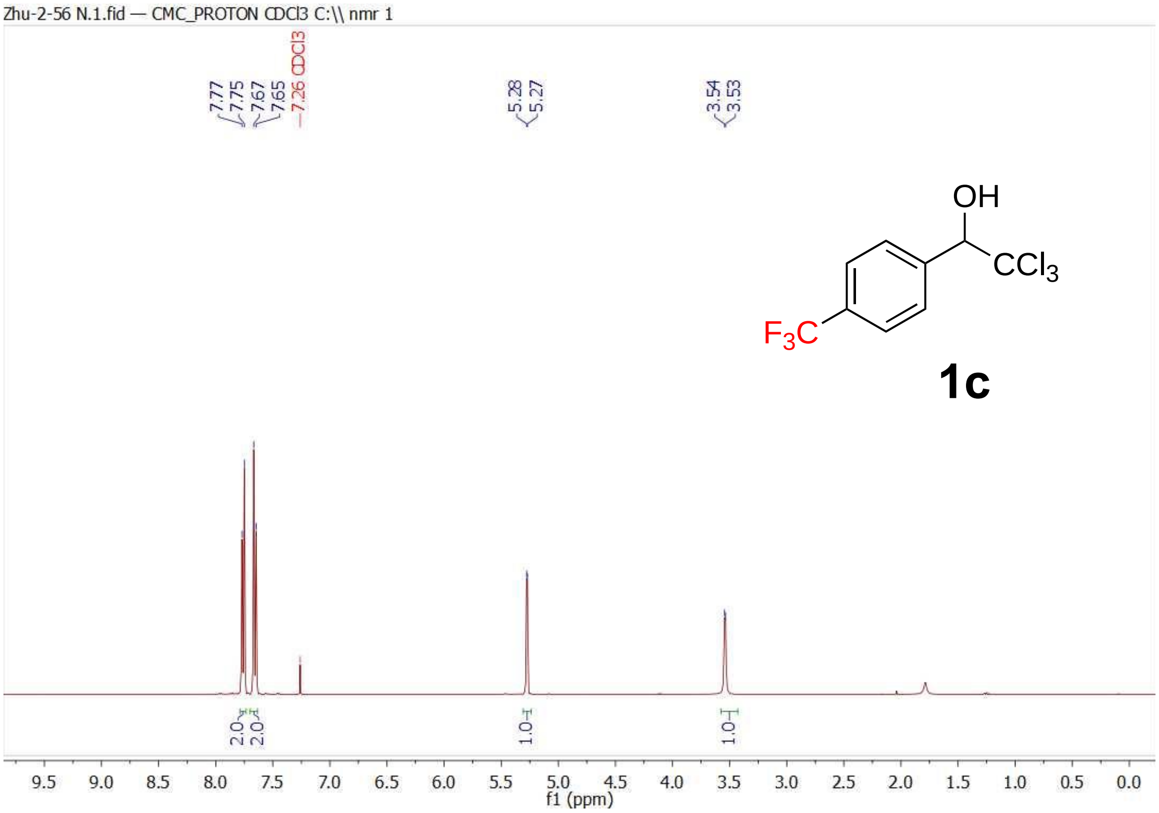
Zhu-2-56 N.1.fid — CMC_PROTON CDCl3 C:\\ nmr 1
7.77
7.75
7.67
7.65
7.26 CDCl3
5.28
5.27
3.54
3.53
OH
CCl3
F3C
1c
2.0
2.0
1.0
1.0
9.5 9.0 8.5 8.0 7.5 7.0 6.5 6.0 5.5 5.0 4.5 4.0 3.5 3.0 2.5 2.0 1.5 1.0 0.5 0.0
f1 (ppm)

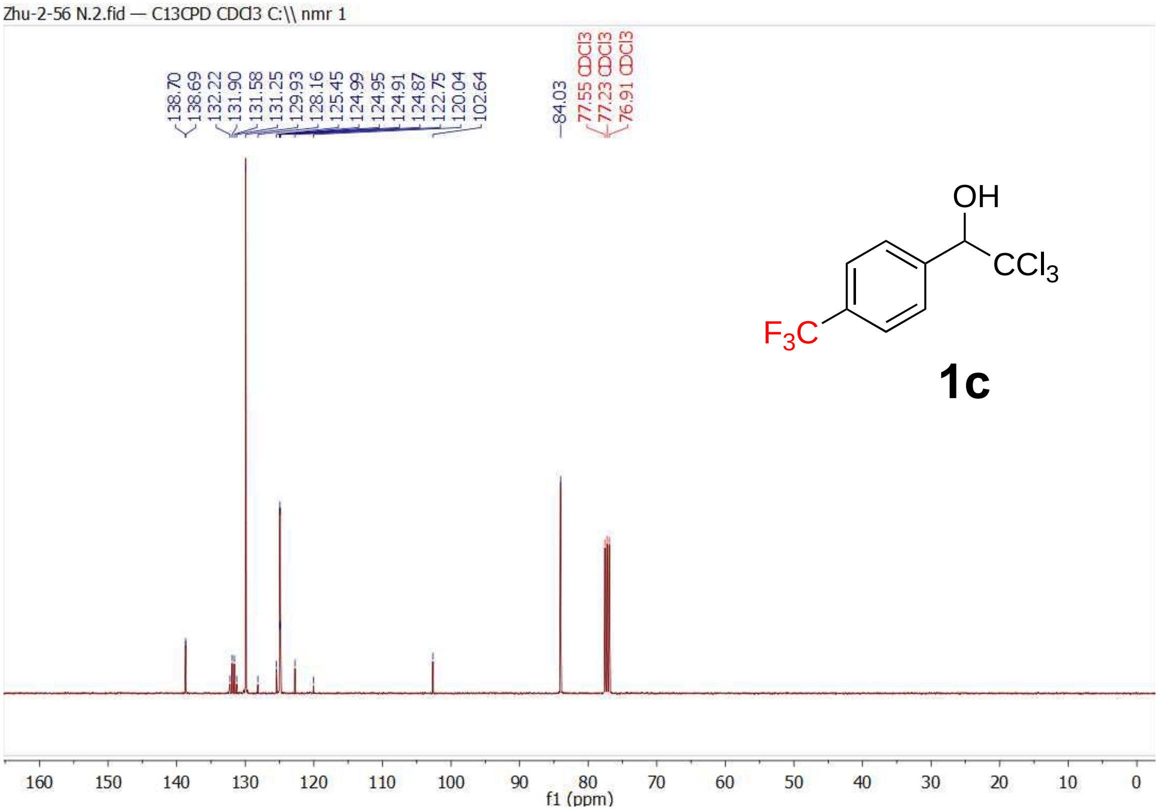
Zhu-2-56 N.2.fid — C13CPD CDCl3 C:\\ nmr 1
138.70
138.69
132.22
131.90
131.58
131.25
129.93
128.16
125.45
124.99
124.95
124.91
124.87
122.75
120.04
102.64
84.03
77.55 CDCl3
77.23 CDCl3
76.91 CDCl3
OH
CCl3
F3C
1c
160 150 140 130 120 110 100 90 80 70 60 50 40 30 20 10 0
f1 (ppm)

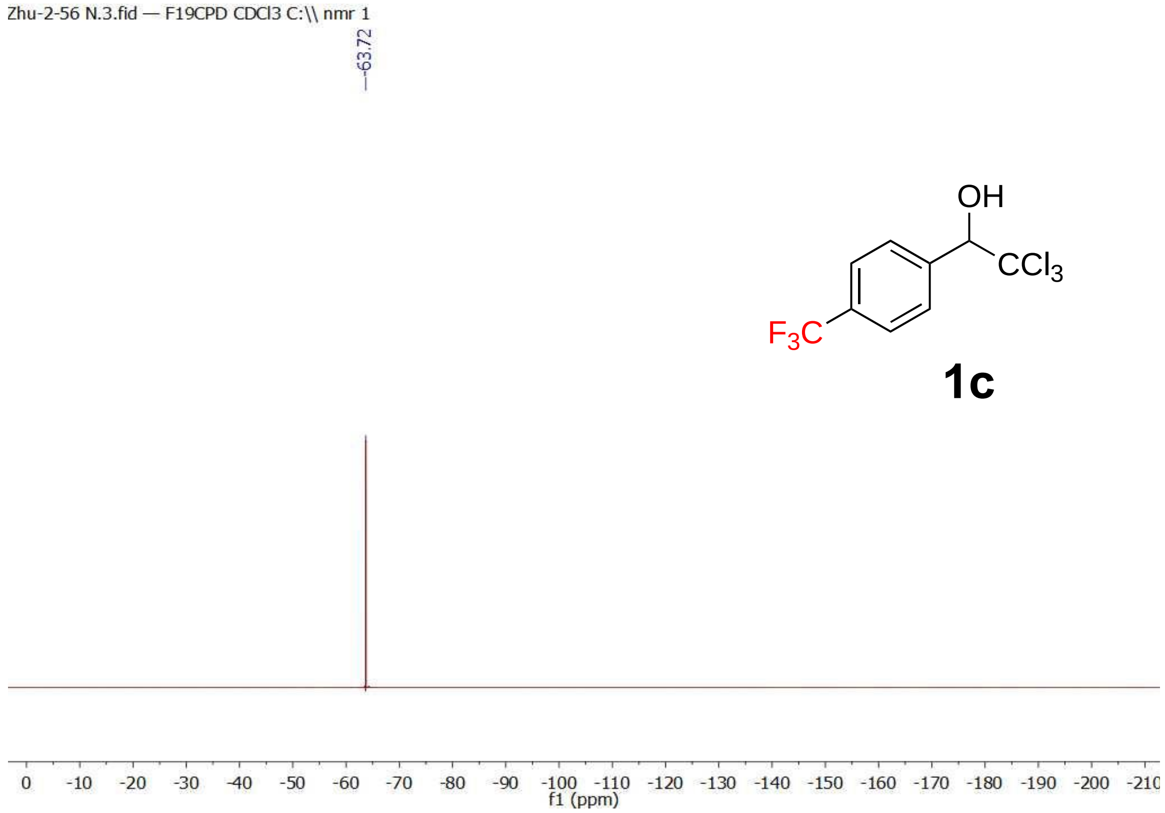
Zhu-2-56 N.3.fid — F19CPD CDCl3 C:\\ nmr 1
-63.72
OH
CCl3
F3C
1c
0 -10 -20 -30 -40 -50 -60 -70 -80 -90 -100 -110 -120 -130 -140 -150 -160 -170 -180 -190 -200 -210
f1 (ppm)

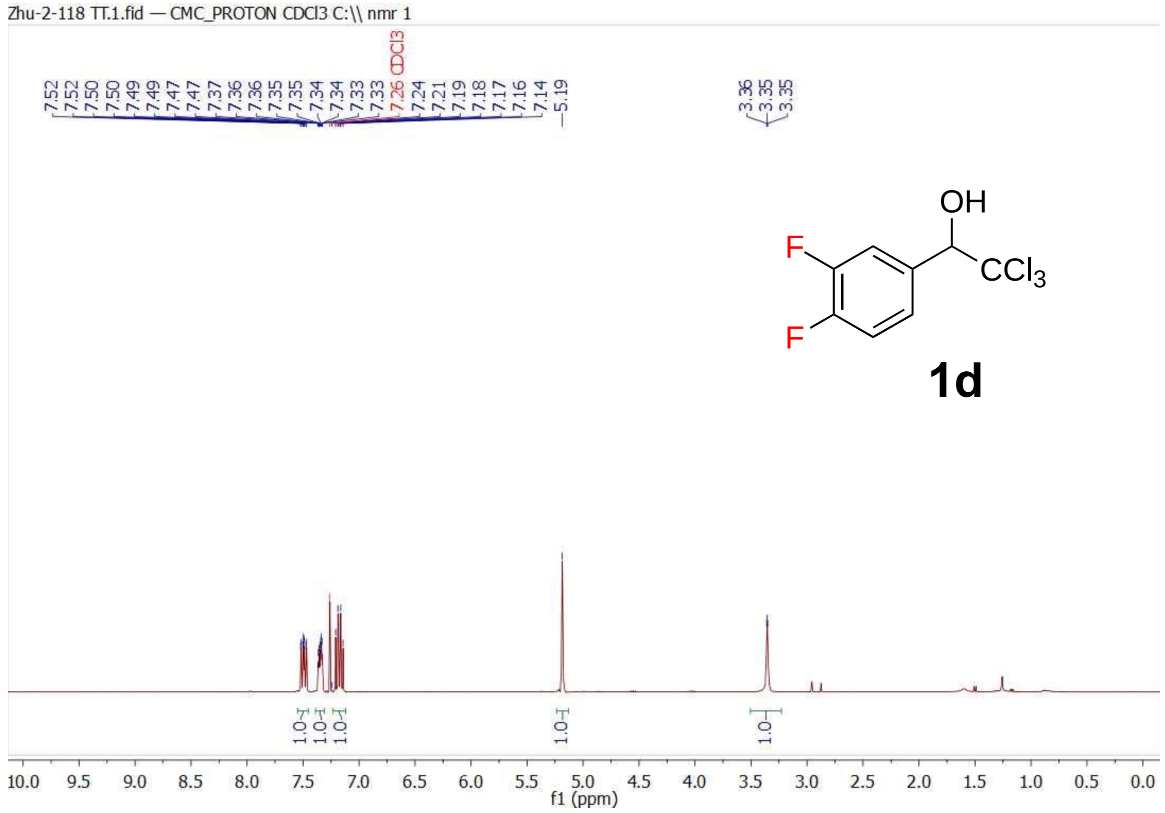
Zhu-2-118 TT.1.fid — CMC_PROTON CDCl3 C:\\ nmr 1
7.52 7.52 7.50 7.49 7.49 7.47 7.37 7.36 7.36 7.35 7.35 7.34 7.34 7.33 7.33 7.26 CDCl3 7.24 7.21 7.19 7.18 7.17 7.16 7.14 5.19
3.36 3.35 3.35
OH
F
CCl3
F
1d
1.0 1.0 1.0 1.0 1.0
10.0 9.5 9.0 8.5 8.0 7.5 7.0 6.5 6.0 5.5 5.0 4.5 4.0 3.5 3.0 2.5 2.0 1.5 1.0 0.5 0.0
f1 (ppm)

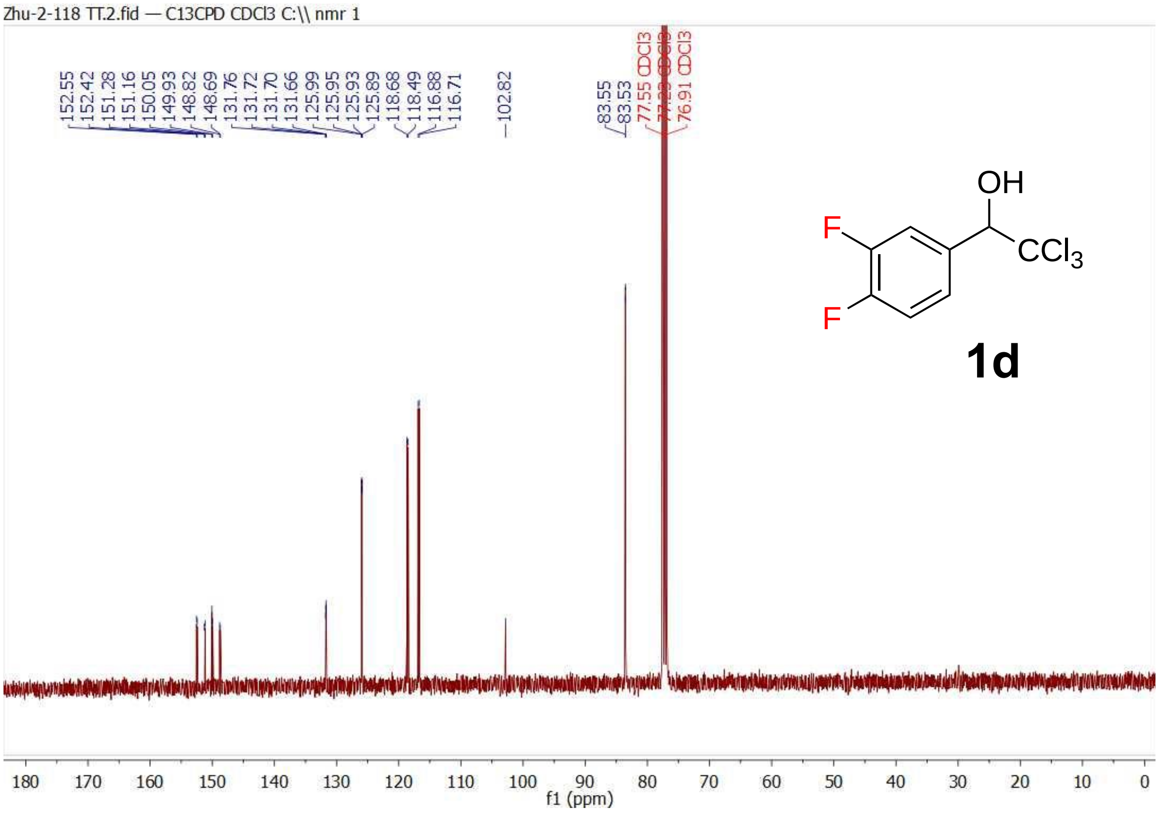
Zhu-2-118 TT.2.fid — C13CPD CDCl3 C:\\ nmr 1
152.55
152.42
151.28
151.16
150.05
149.93
148.82
148.69
131.76
131.72
131.70
131.66
125.99
125.95
125.93
125.89
118.68
118.49
116.88
116.71
102.82
83.55
83.53
77.55 CDCl3
77.23 CDCl3
76.91 CDCl3
OH
F
CCl3
F
1d
f1 (ppm)

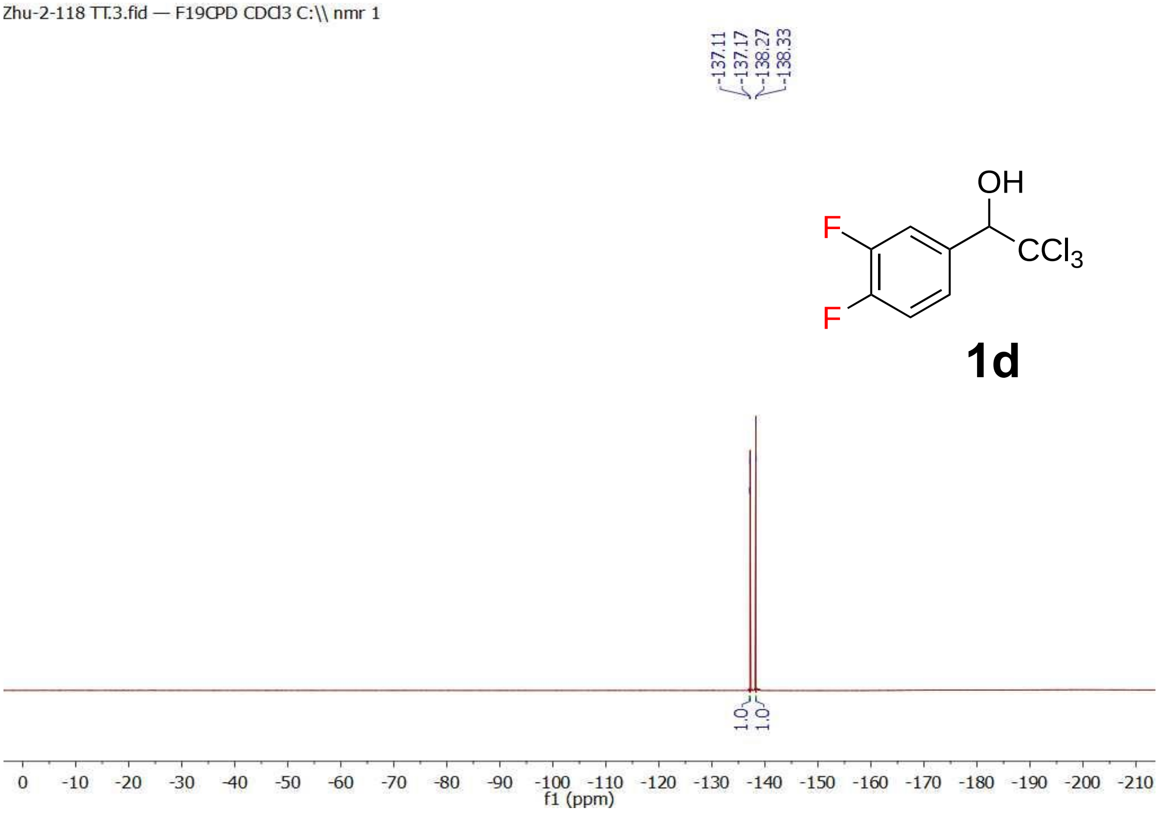
Zhu-2-118 TT.3.fid — F19CPD CDCl3 C:\\ nmr 1
-137.11
-137.17
-138.27
-138.33
OH
F
CCl3
F
1d
1.0
1.0
f1 (ppm)

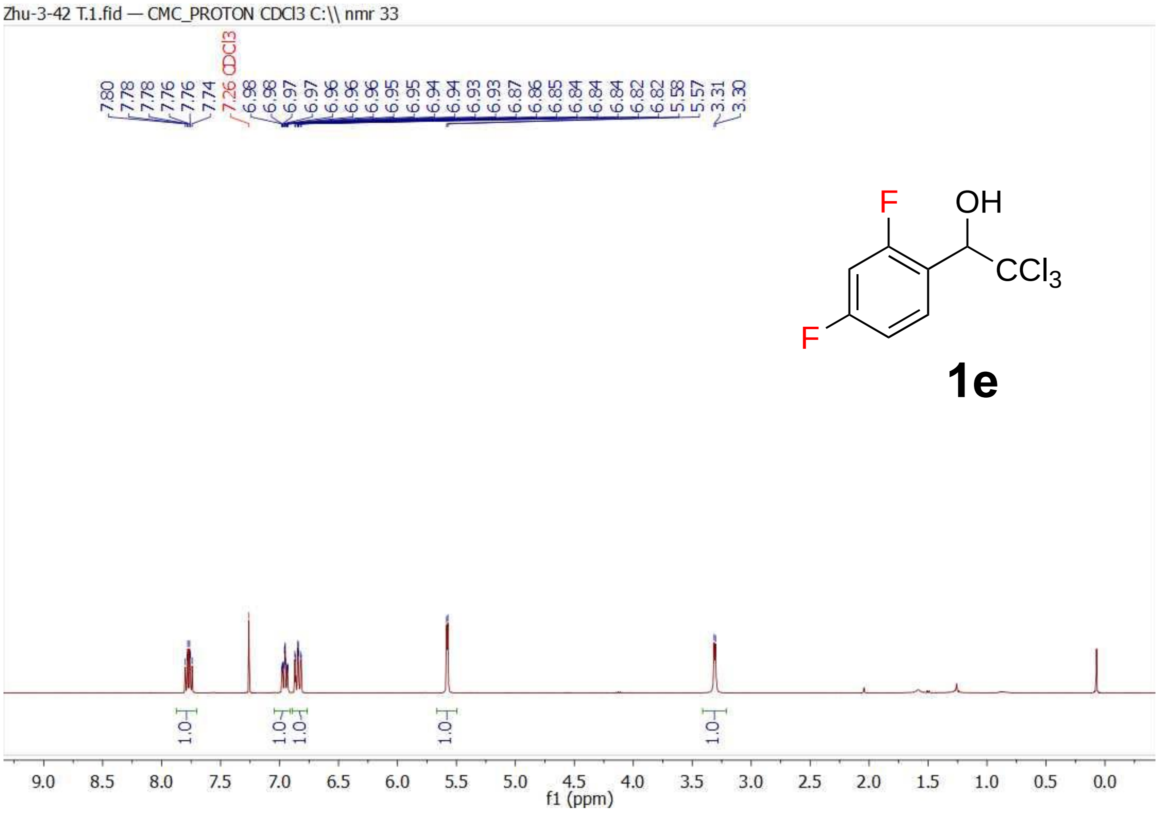
Zhu-3-42 T.1.fid — CMC_PROTON CDCl3 C:\\ nmr 33
7.80 7.78 7.78 7.76 7.76 7.74 7.26 CDCl3 6.98 6.98 6.97 6.97 6.96 6.96 6.96 6.95 6.95 6.94 6.94 6.93 6.93 6.87 6.86 6.85 6.84 6.84 6.82 6.82 5.58 5.57 3.31 3.30
1.0 1.0 1.0 1.0 1.0
9.0 8.5 8.0 7.5 7.0 6.5 6.0 5.5 5.0 4.5 4.0 3.5 3.0 2.5 2.0 1.5 1.0 0.5 0.0
f1 (ppm)
F
OH
CCl3
F
1e

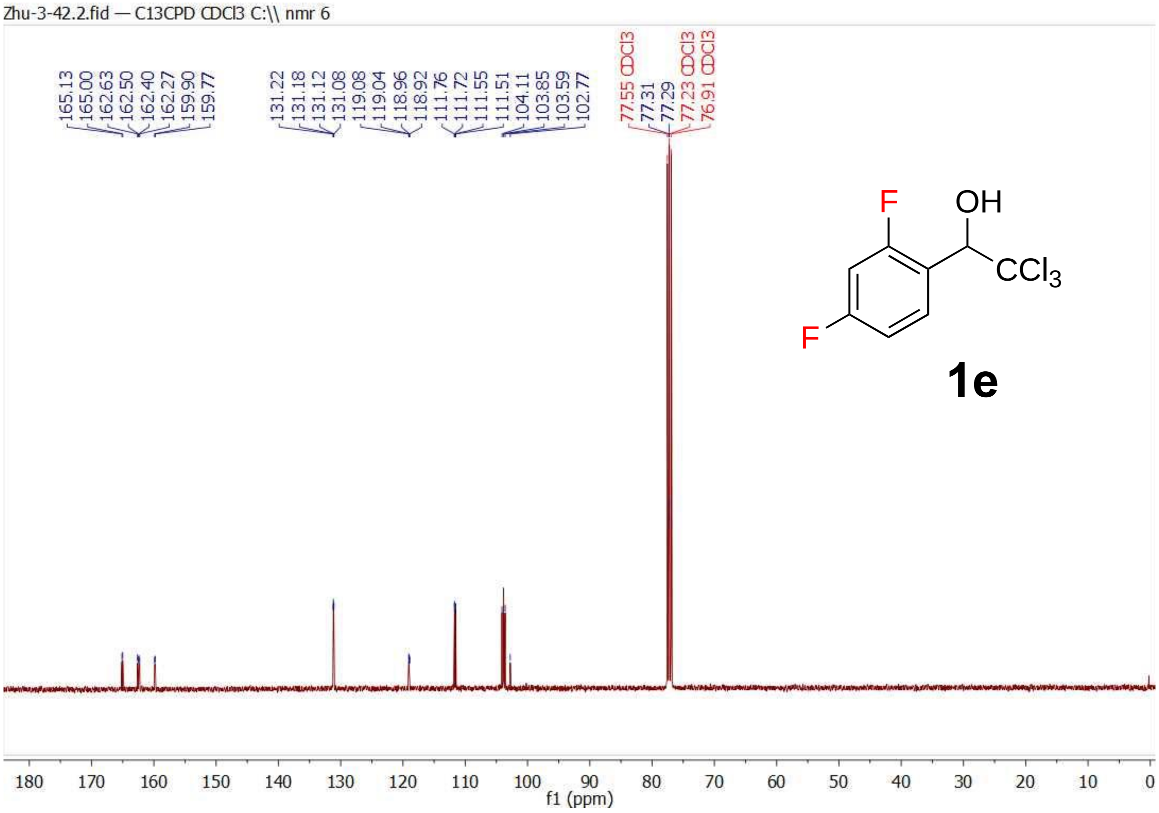
Zhu-3-42.2.fid — C13CPD CDCl3 C:\\ nmr 6
165.13 165.00 162.63 162.50 162.40 162.27 159.90 159.77
131.22 131.18 131.12 131.08 119.08 119.04 118.96 118.92 111.76 111.72 111.55 111.51 104.11 103.85 103.59 102.77
77.55 CDCl3 77.31 77.29 77.23 CDCl3 76.91 CDCl3
180 170 160 150 140 130 120 110 100 90 80 70 60 50 40 30 20 10 0
f1 (ppm)
F
OH
CCl3
F
1e

Zhu-3-42 T.3.fid — F19CPD CDCl3 C:\\ nmr 33

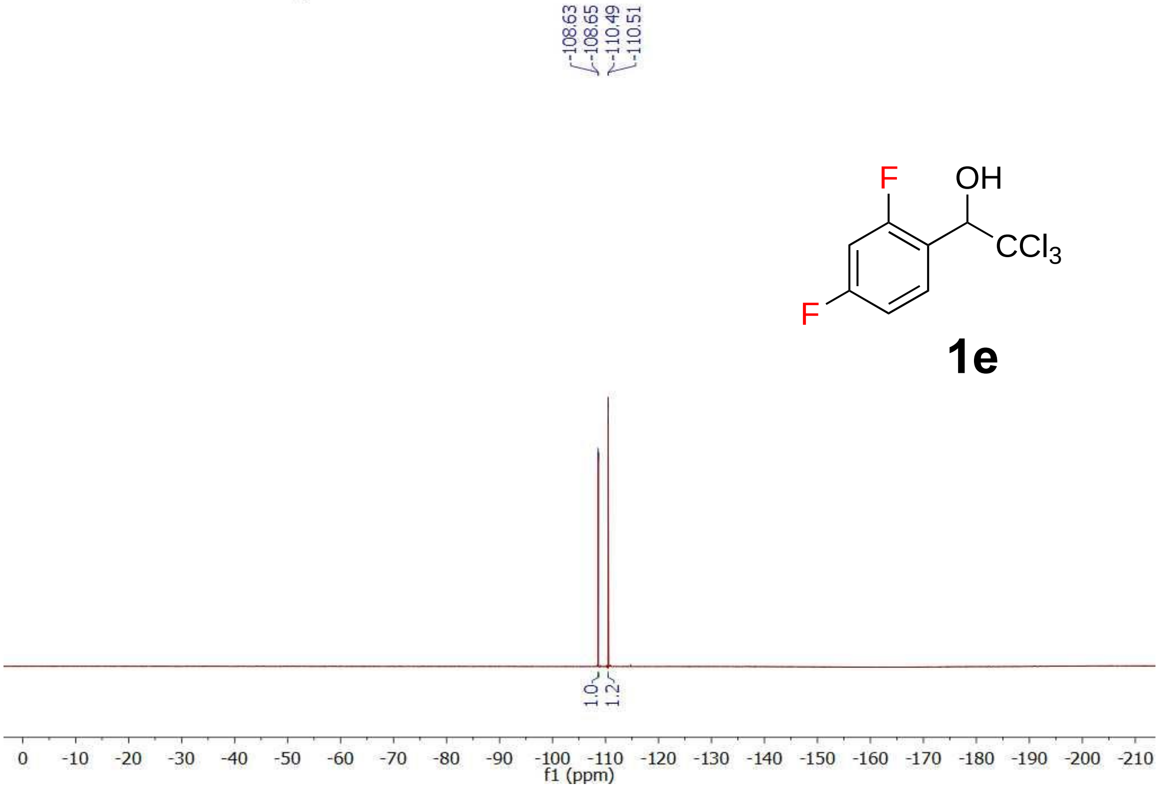

Zhu-3-92.1.fid — CMC_PROTON CDCl3 C:\\ nmr 36

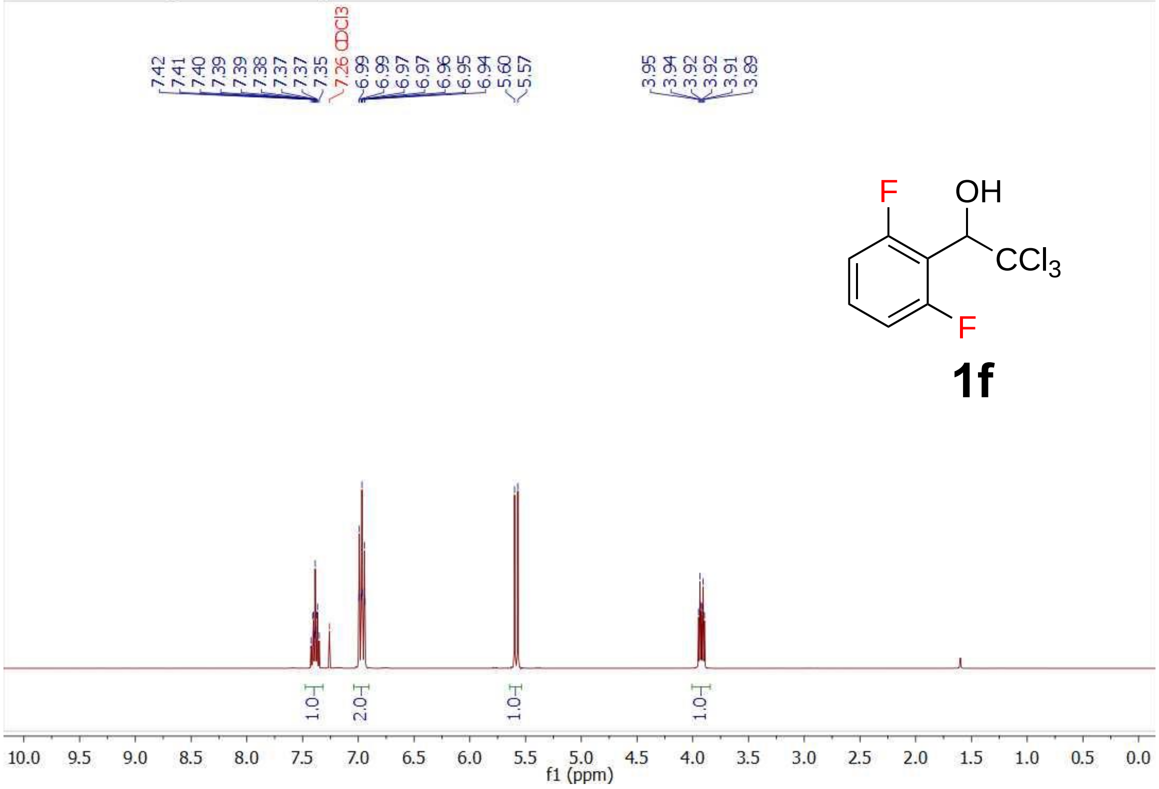

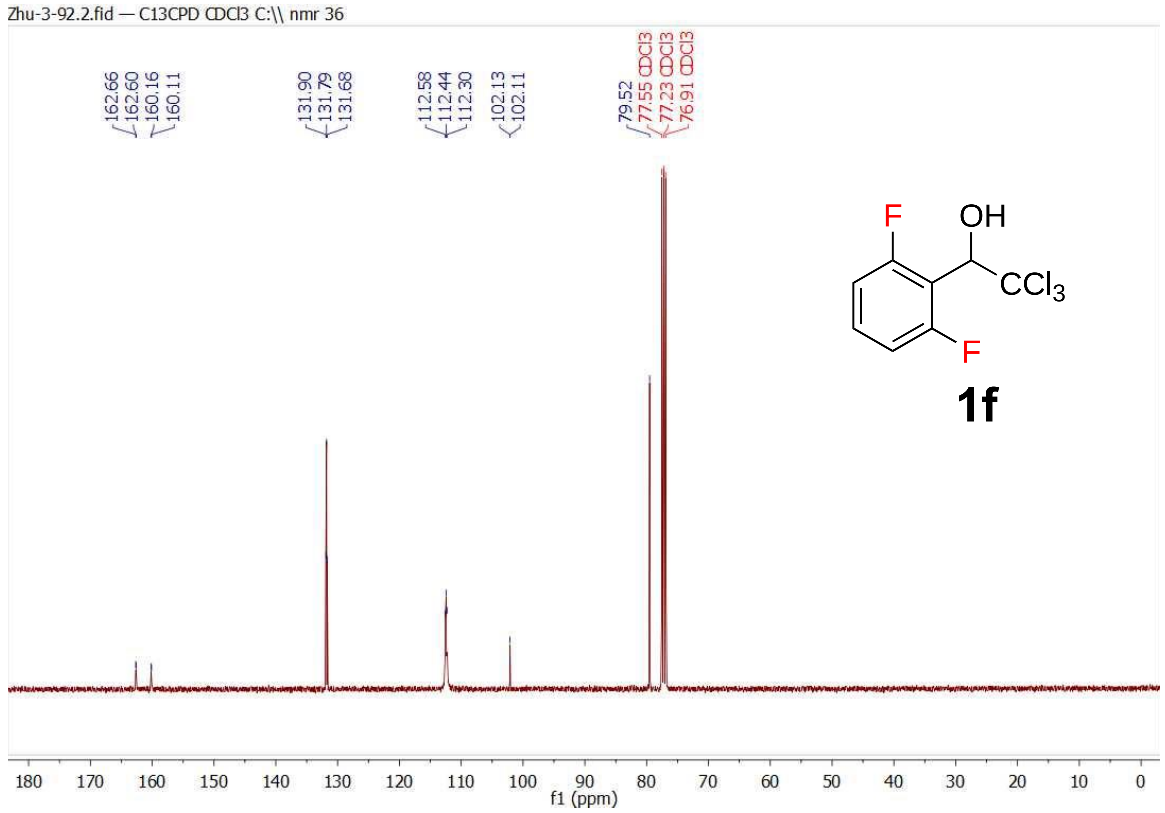
Zhu-3-92.2.fid — C13CPD CDCl3 C:\\ nmr 36
162.66
162.60
160.16
160.11
131.90
131.79
131.68
112.58
112.44
112.30
102.13
102.11
79.52
77.55 CDCl3
77.23 CDCl3
76.91 CDCl3
F
OH
CCl3
F
1f
180 170 160 150 140 130 120 110 100 90 80 70 60 50 40 30 20 10 0
f1 (ppm)

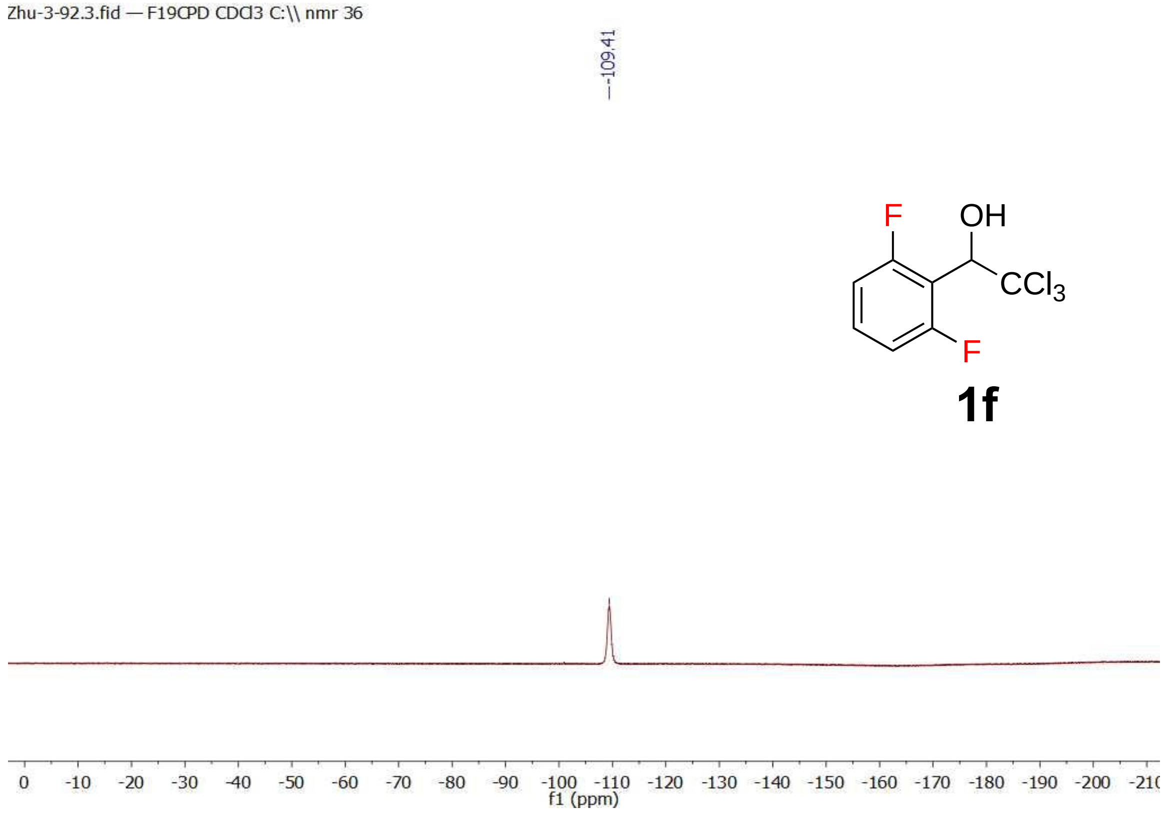
Zhu-3-92.3.fid — F19CPD CDCl3 C:\\ nmr 36
-109.41
F
OH
CCl3
F
1f
0 -10 -20 -30 -40 -50 -60 -70 -80 -90 -100 -110 -120 -130 -140 -150 -160 -170 -180 -190 -200 -210
f1 (ppm)

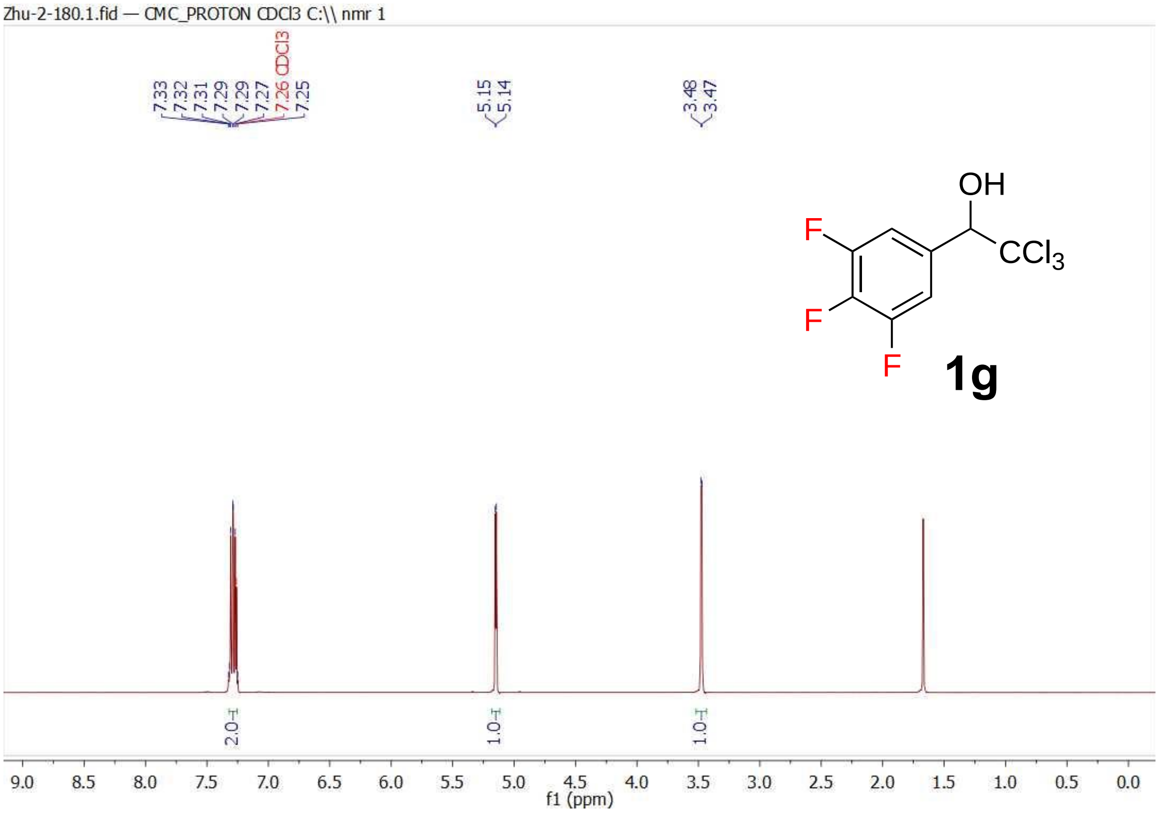
Zhu-2-180.1.fid — CMC_PROTON CDCl3 C:\\ nmr 1
7.33
7.32
7.31
7.29
7.29
7.27
7.26 CDCl3
7.25
5.15
5.14
3.48
3.47
OH
F
F
F
CCl3
1g
2.0
1.0
1.0
9.0 8.5 8.0 7.5 7.0 6.5 6.0 5.5 5.0 4.5 4.0 3.5 3.0 2.5 2.0 1.5 1.0 0.5 0.0
f1 (ppm)

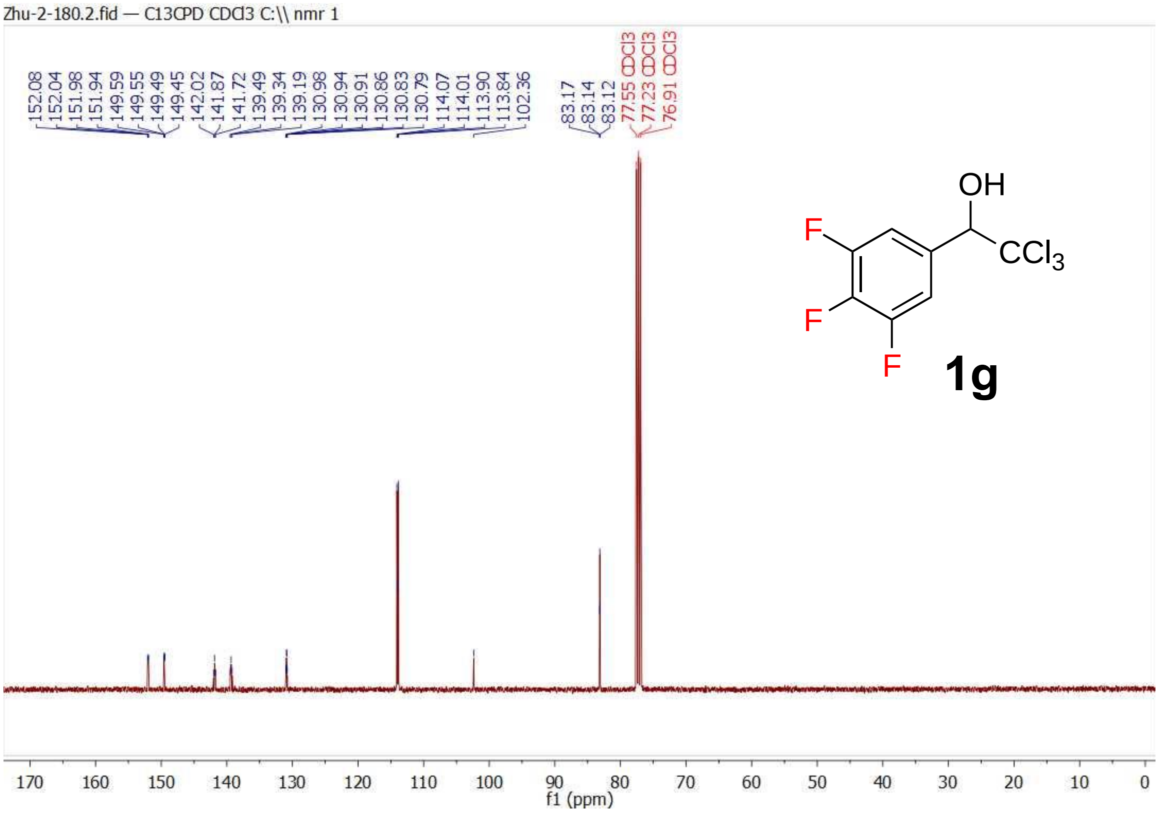
Zhu-2-180.2.fid — C13CPD CDCl3 C:\\ nmr 1
152.08
152.04
151.98
151.94
149.59
149.55
149.49
149.45
142.02
141.87
141.72
139.49
139.34
139.19
130.98
130.94
130.91
130.86
130.83
130.79
114.07
114.01
113.90
113.84
102.36
83.17
83.14
83.12
77.55 CDCl3
77.23 CDCl3
76.91 CDCl3
OH
F
F
F
CCl3
1g
170 160 150 140 130 120 110 100 90 80 70 60 50 40 30 20 10 0
f1 (ppm)

Zhu-2-180.3.fid — F19CPD CDCl3 C:\\ nmr 1

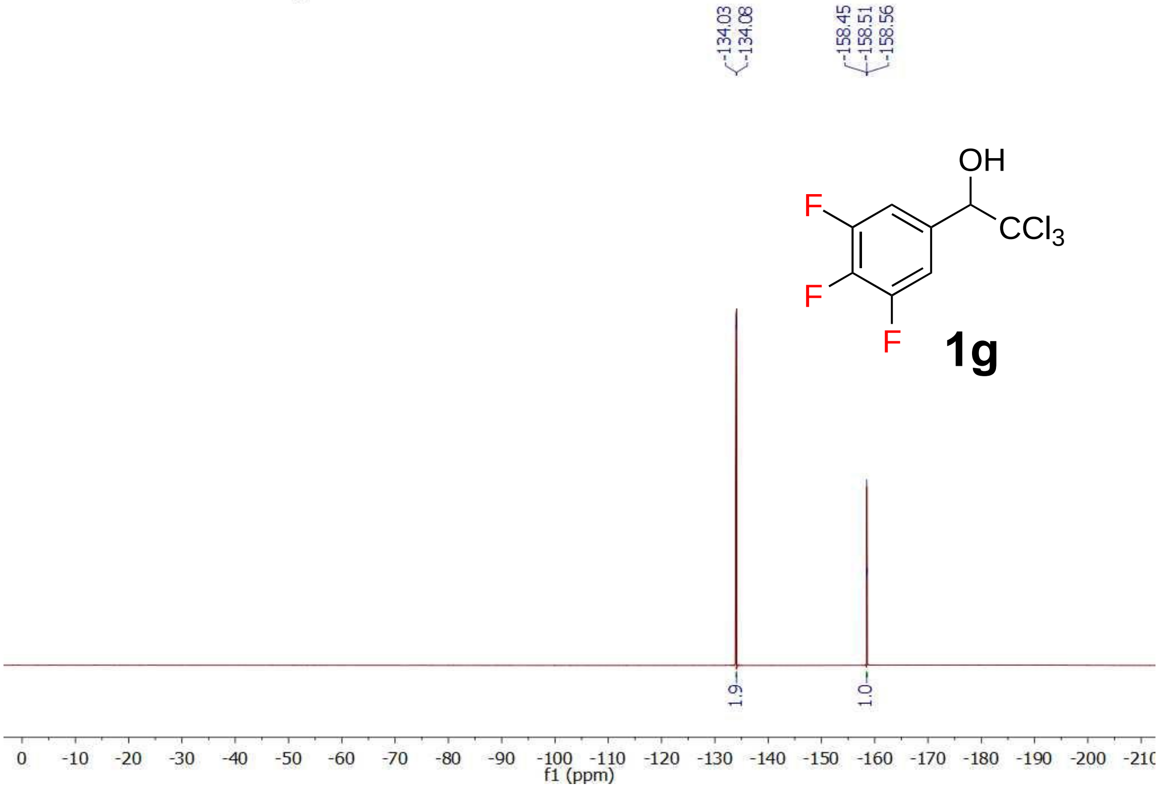

1g

Zhu-3-62.1.fid — CMC_PROTON CDCl3 C:\\ nmr 6

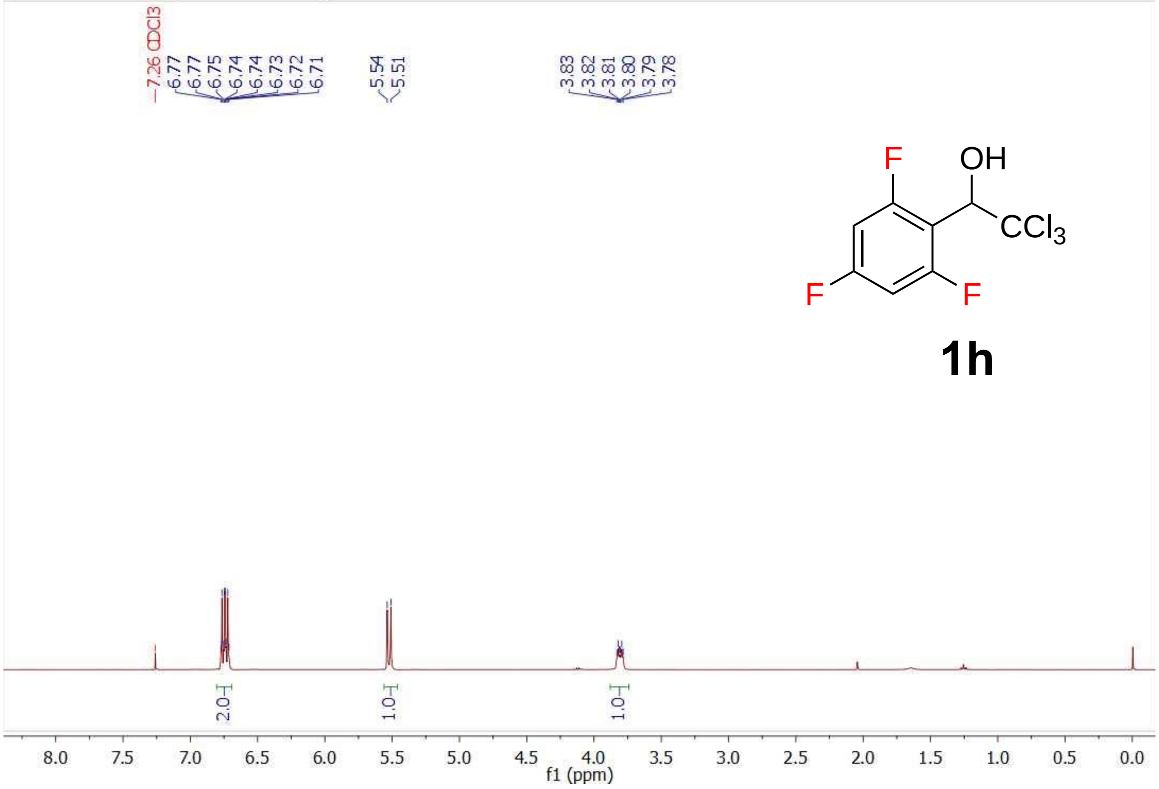

1h

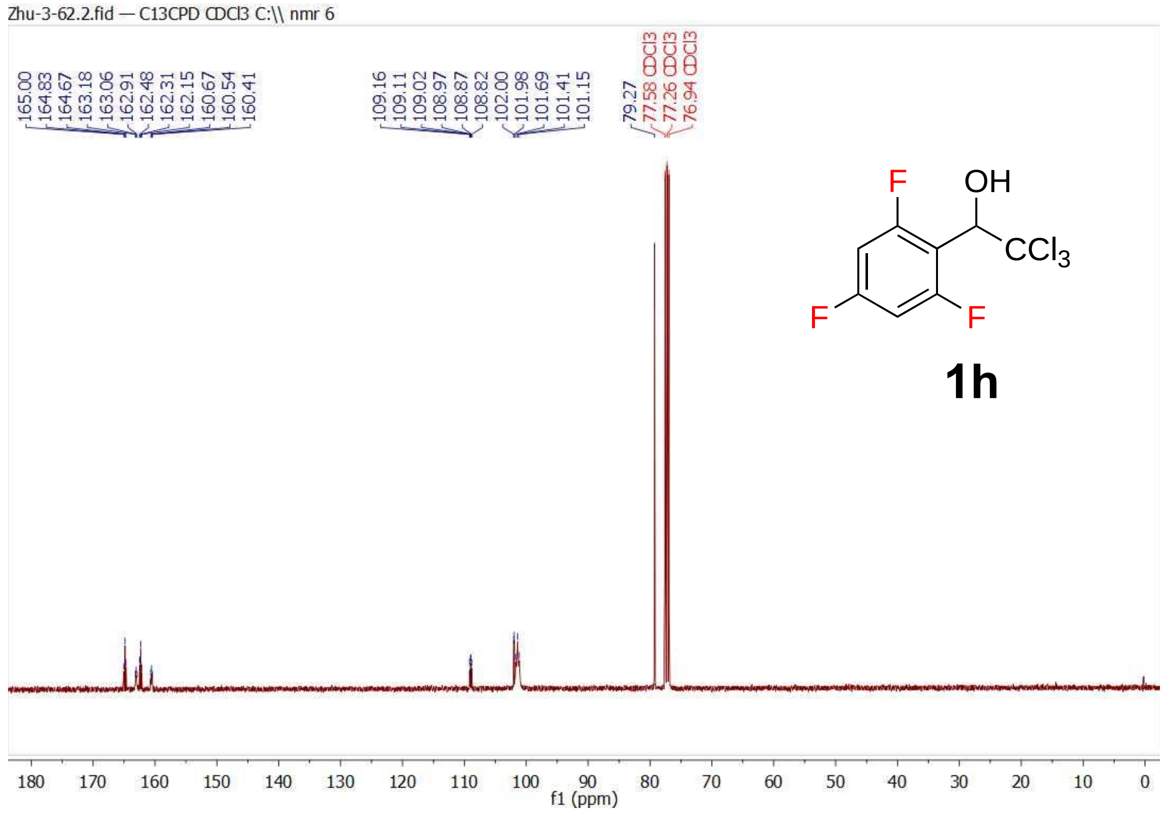
Zhu-3-62.2.fid — C13CPD CDCl3 C:\\ nmr 6
165.00
164.83
164.67
163.18
163.06
162.91
162.48
162.31
162.15
160.67
160.54
160.41
109.16
109.11
109.02
108.97
108.87
108.82
102.00
101.98
101.69
101.41
101.15
79.27
77.58 CDCl3
77.26 CDCl3
76.94 CDCl3
F
OH
CCl3
F
F
1h
180
170
160
150
140
130
120
110
100
90
80
70
60
50
40
30
20
10
0
f1 (ppm)

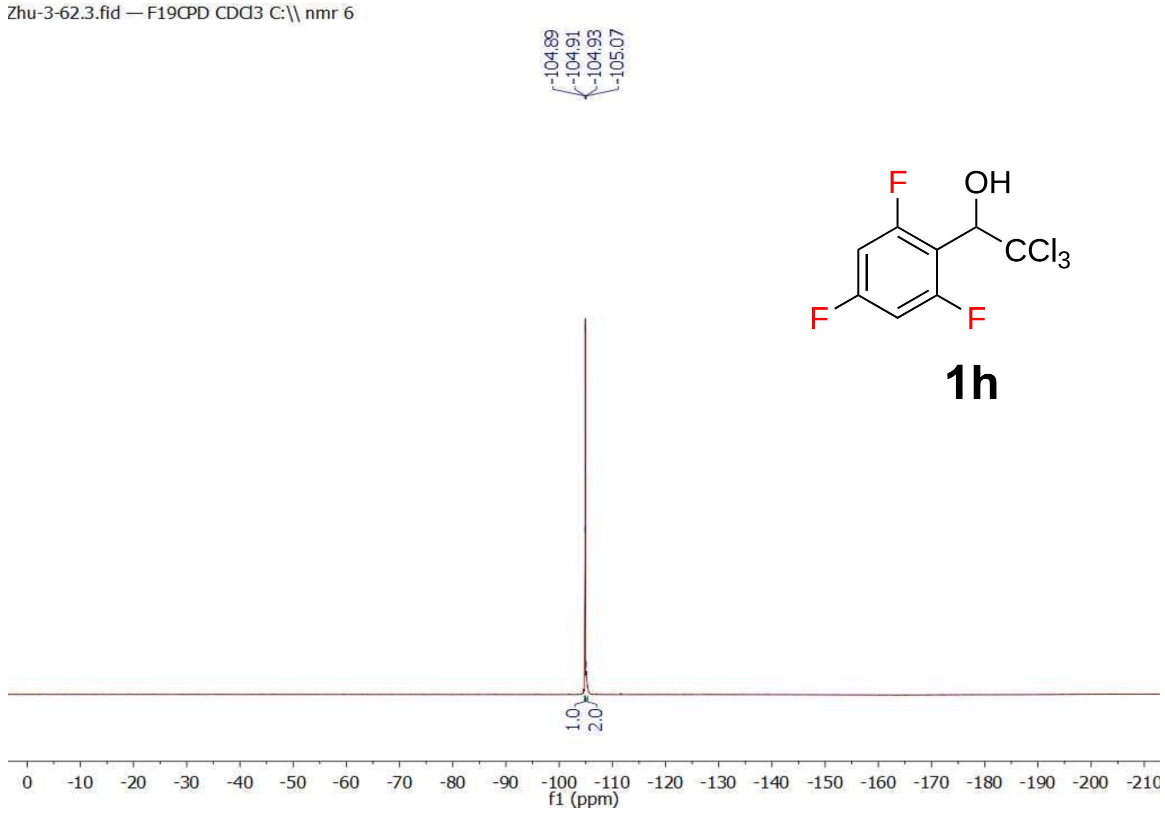
Zhu-3-62.3.fid — F19CPD CDCl3 C:\\ nmr 6
-104.89
-104.91
-104.93
-105.07
F
OH
CCl3
F
F
1h
1.0
2.0
0
-10
-20
-30
-40
-50
-60
-70
-80
-90
-100
-110
-120
-130
-140
-150
-160
-170
-180
-190
-200
-210
f1 (ppm)

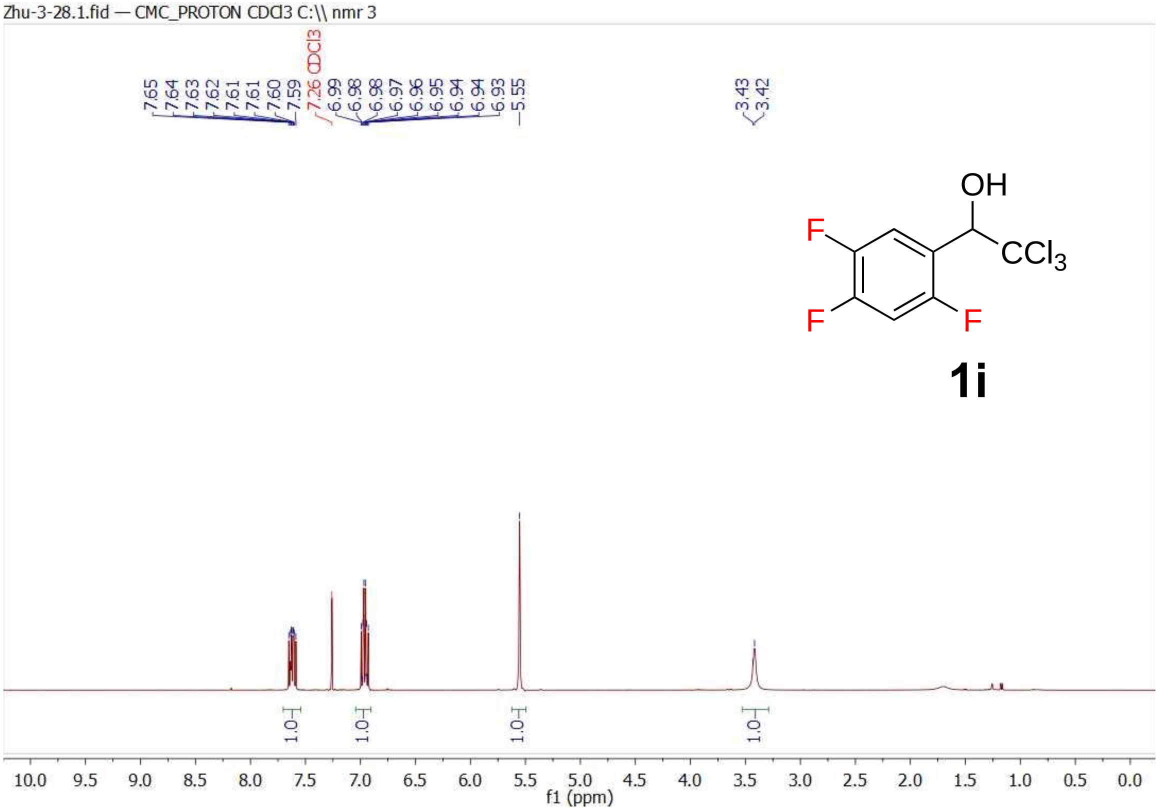
Zhu-3-28.1.fid — CMC_PROTON CDCl3 C:\\ nmr 3
7.65 7.64 7.63 7.62 7.61 7.61 7.60 7.59 7.26 CDCl3 6.99 6.98 6.98 6.97 6.96 6.95 6.94 6.94 6.93 5.55
3.43 3.42
OH
F
CCl3
F
F
1i
1.0
1.0
1.0
1.0
f1 (ppm)

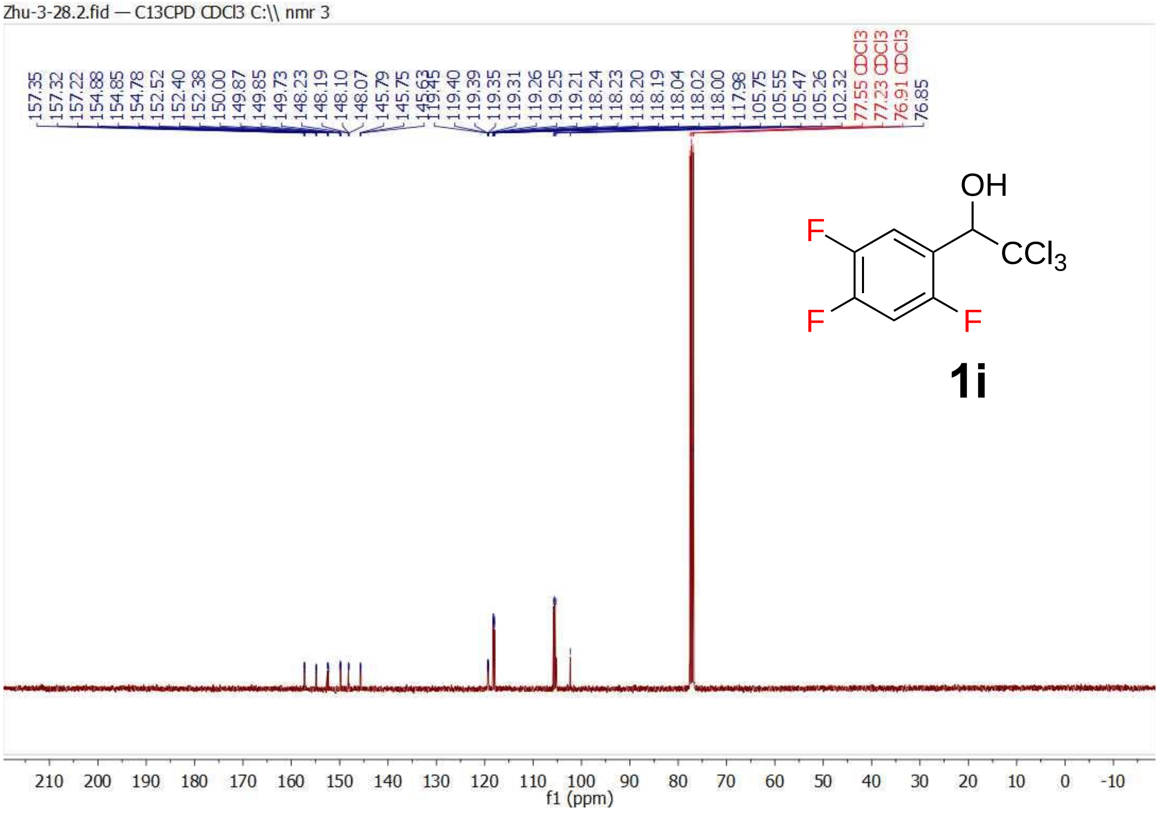
Zhu-3-28.2.fid — C13CPD CDCl3 C:\\ nmr 3
157.35 157.32 157.22 154.88 154.85 154.78 152.52 152.40 152.38 150.00 149.87 149.85 149.73 148.23 148.19 148.10 148.07 145.79 145.75 145.63 119.45 119.40 119.39 119.35 119.31 119.26 119.25 119.21 118.24 118.23 118.20 118.19 118.04 118.02 118.00 117.98 105.75 105.55 105.47 105.26 102.32 77.55 CDCl3 77.23 CDCl3 76.91 CDCl3 76.85
OH
F
CCl3
F
F
1i
f1 (ppm)

Zhu-3-28.3.fid — F19CPD CDCl3 C:\\ nmr 3

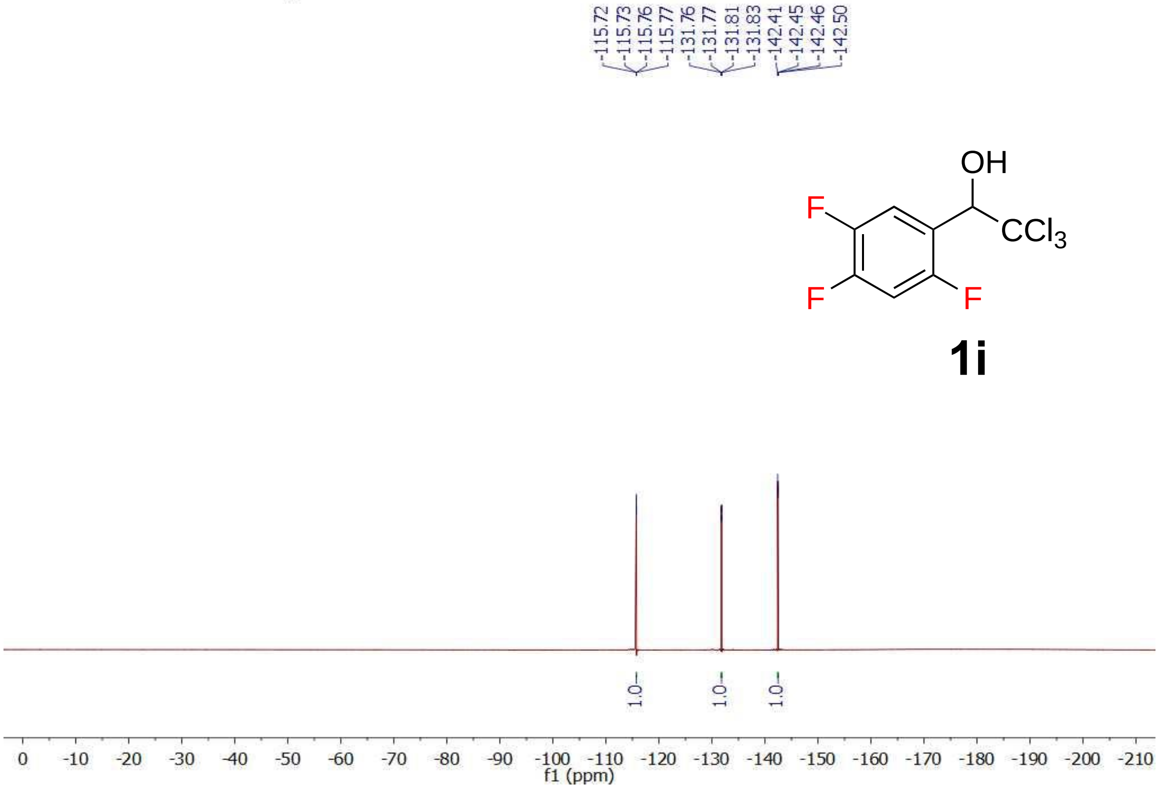

1i

Zhu-3-132.1.fid — CMC_PROTON CDCl3 C:\\ nmr 1

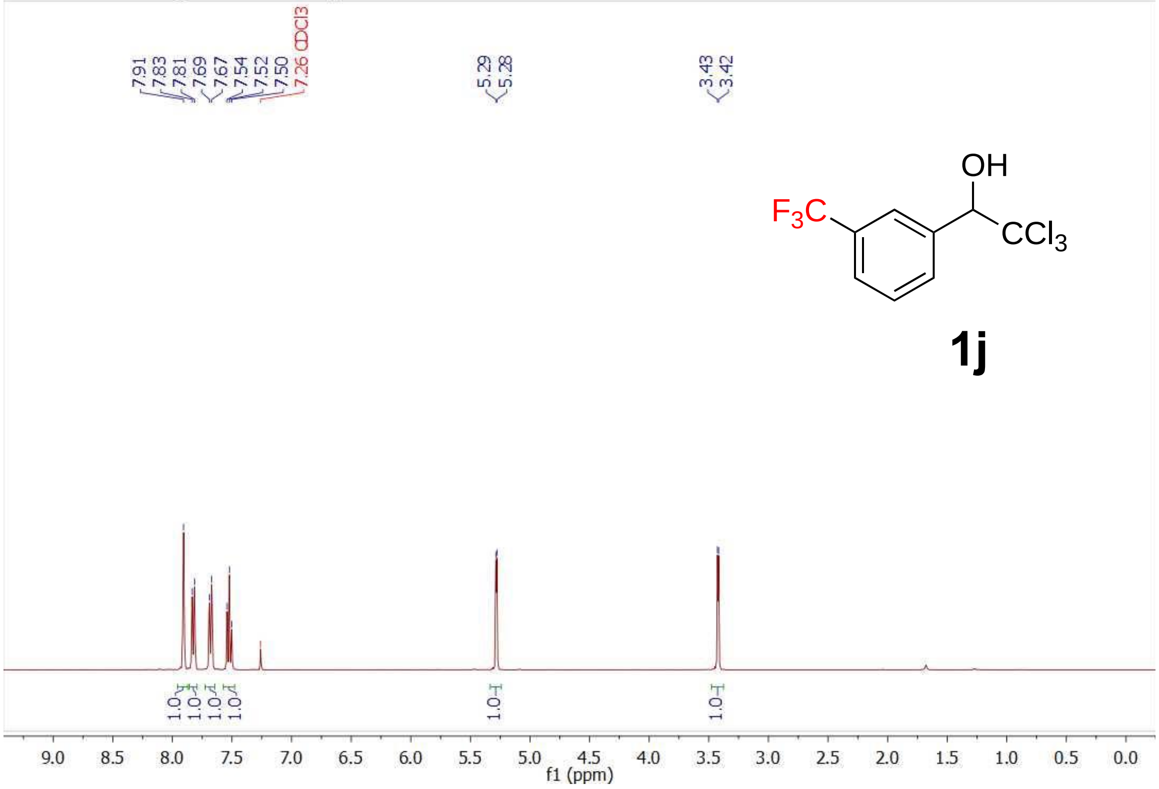

1j

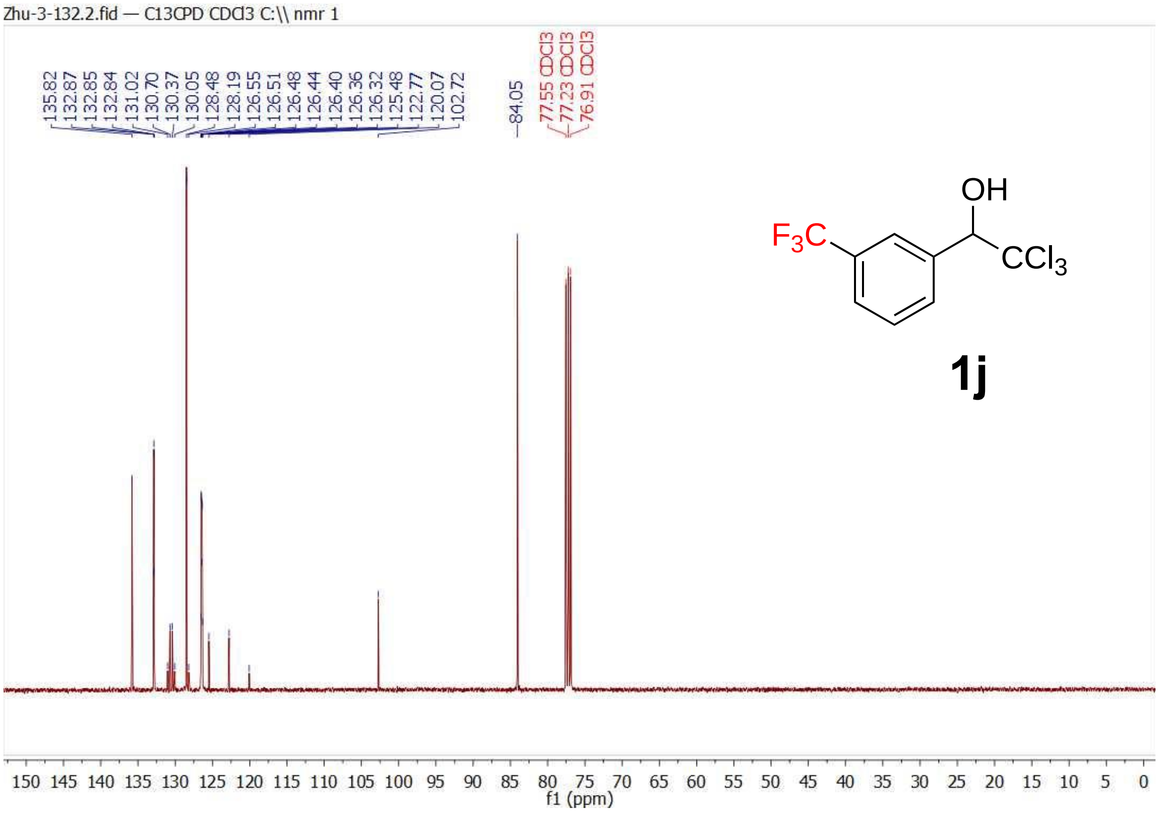
Zhu-3-132.2.fid — C13CPD CDCl3 C:\\ nmr 1
135.82
132.87
132.85
132.84
131.02
130.70
130.37
130.05
128.48
128.19
126.55
126.51
126.48
126.44
126.40
126.36
126.32
125.48
122.77
120.07
102.72
84.05
77.55 CDCl3
77.23 CDCl3
76.91 CDCl3
OH
F3C
CCl3
150 145 140 135 130 125 120 115 110 105 100 95 90 85 80 75 70 65 60 55 50 45 40 35 30 25 20 15 10 5 0
f1 (ppm)

1j

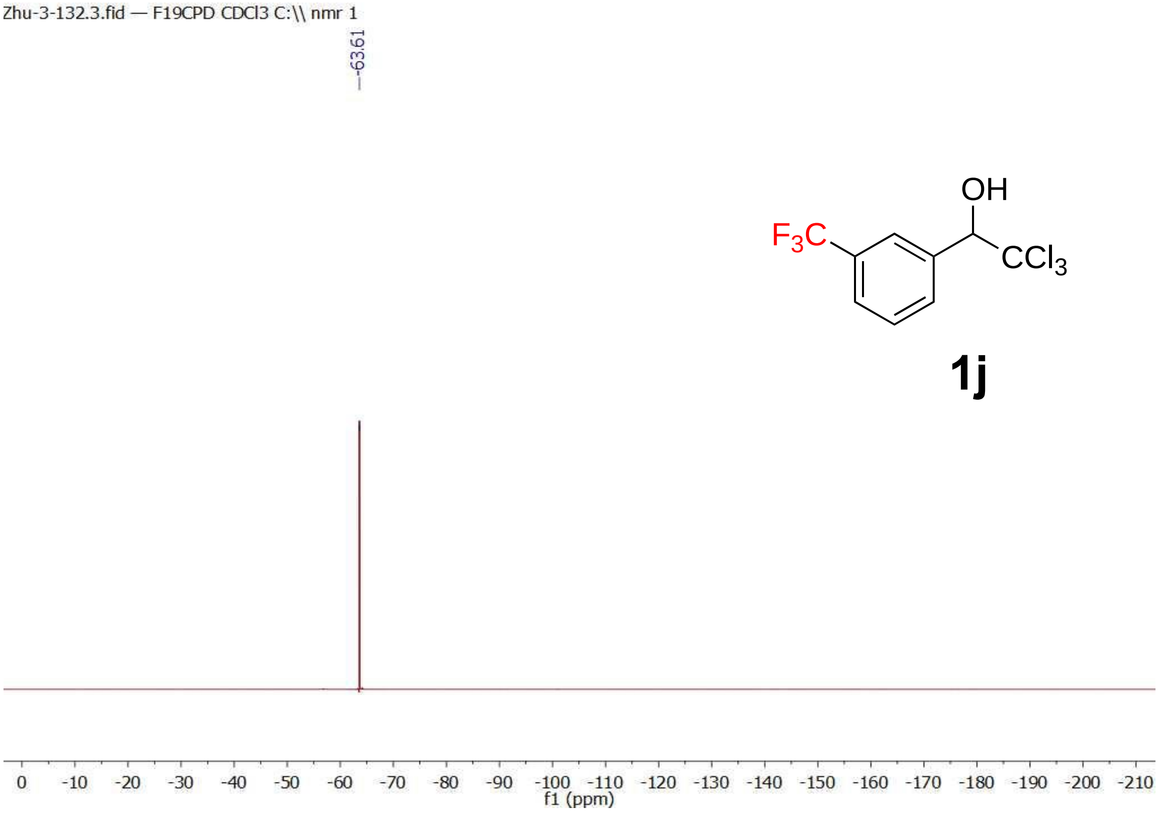
Zhu-3-132.3.fid — F19CPD CDCl3 C:\\ nmr 1
-63.61
OH
F3C
CCl3
0 -10 -20 -30 -40 -50 -60 -70 -80 -90 -100 -110 -120 -130 -140 -150 -160 -170 -180 -190 -200 -210
f1 (ppm)

1j

Zhu-3-14.1.fid — CMC_PROTON CDCl3 C:\\ nmr 3

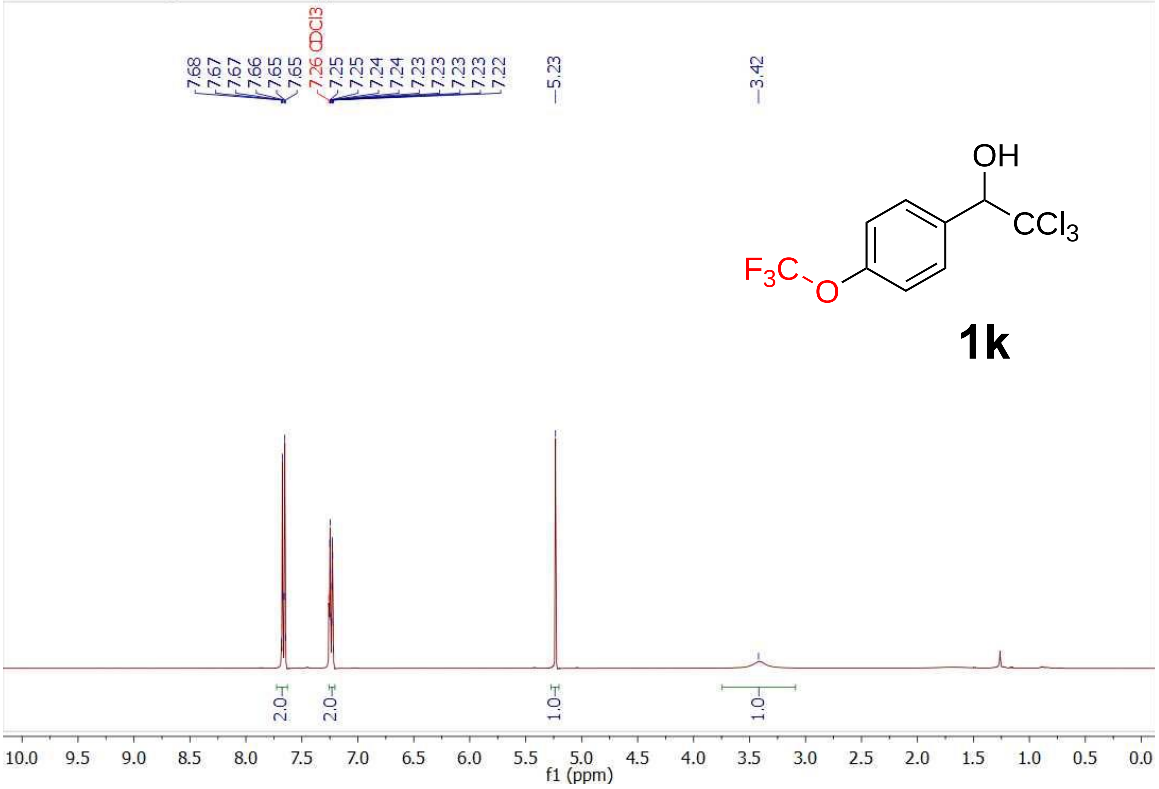

1k

Zhu-3-14.2.fid — C13CPD CDCl3 C:\\ nmr 3

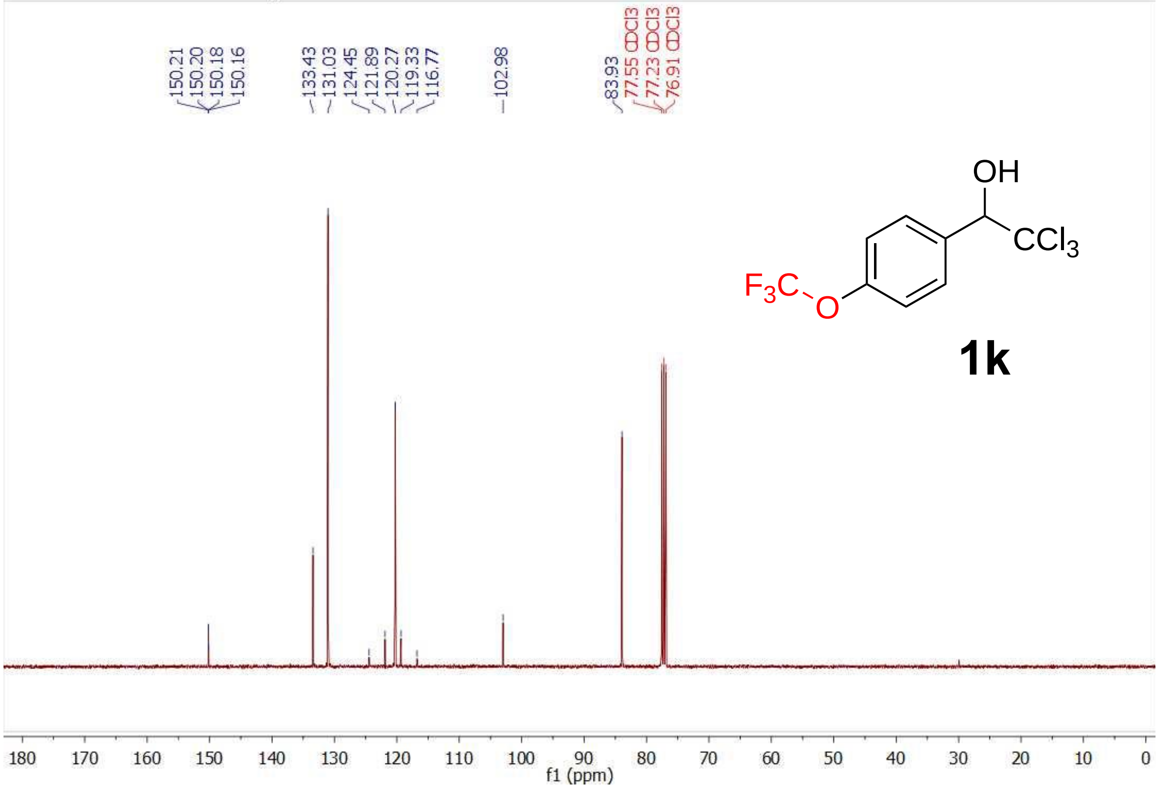

1k

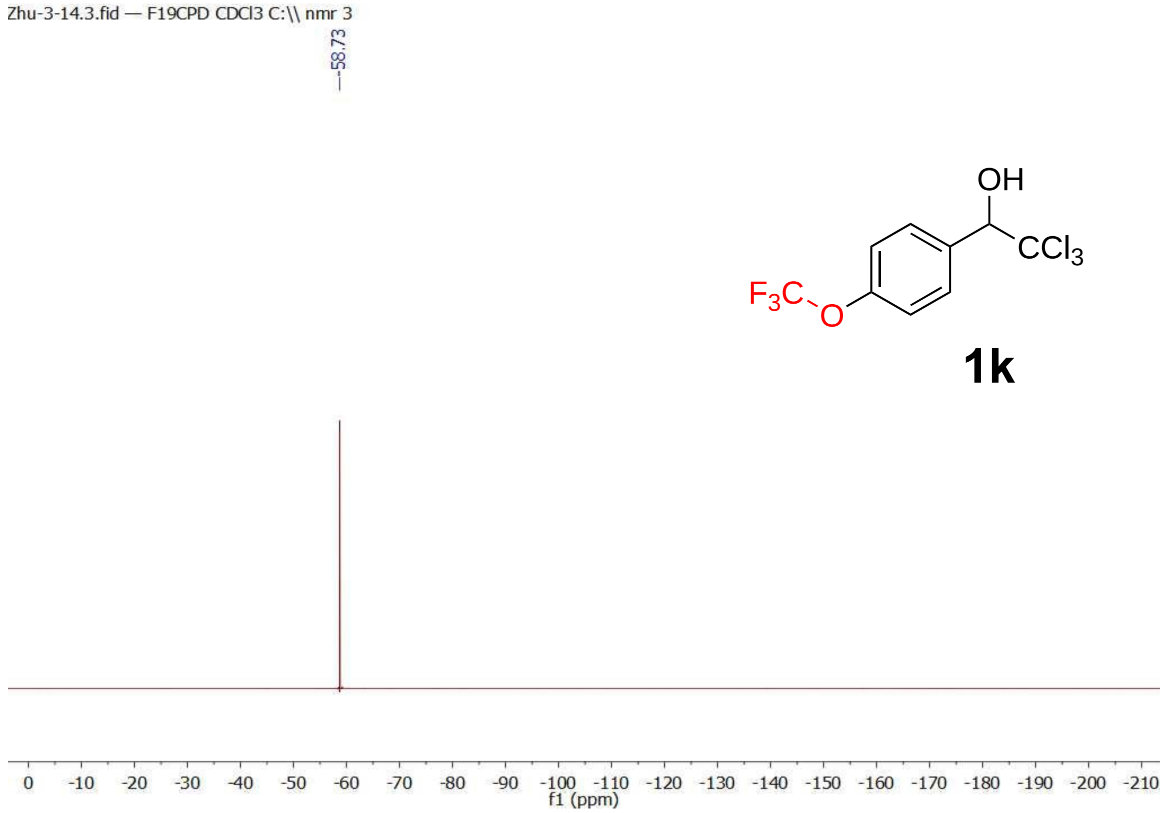

Zhu-3-14.3.fid — F19CPD CDCl3 C:\\ nmr 3
-58.73
OH
CCl3
F3C
O
1k
0
-10
-20
-30
-40
-50
-60
-70
-80
-90
-100
-110
-120
-130
-140
-150
-160
-170
-180
-190
-200
-210
f1 (ppm)

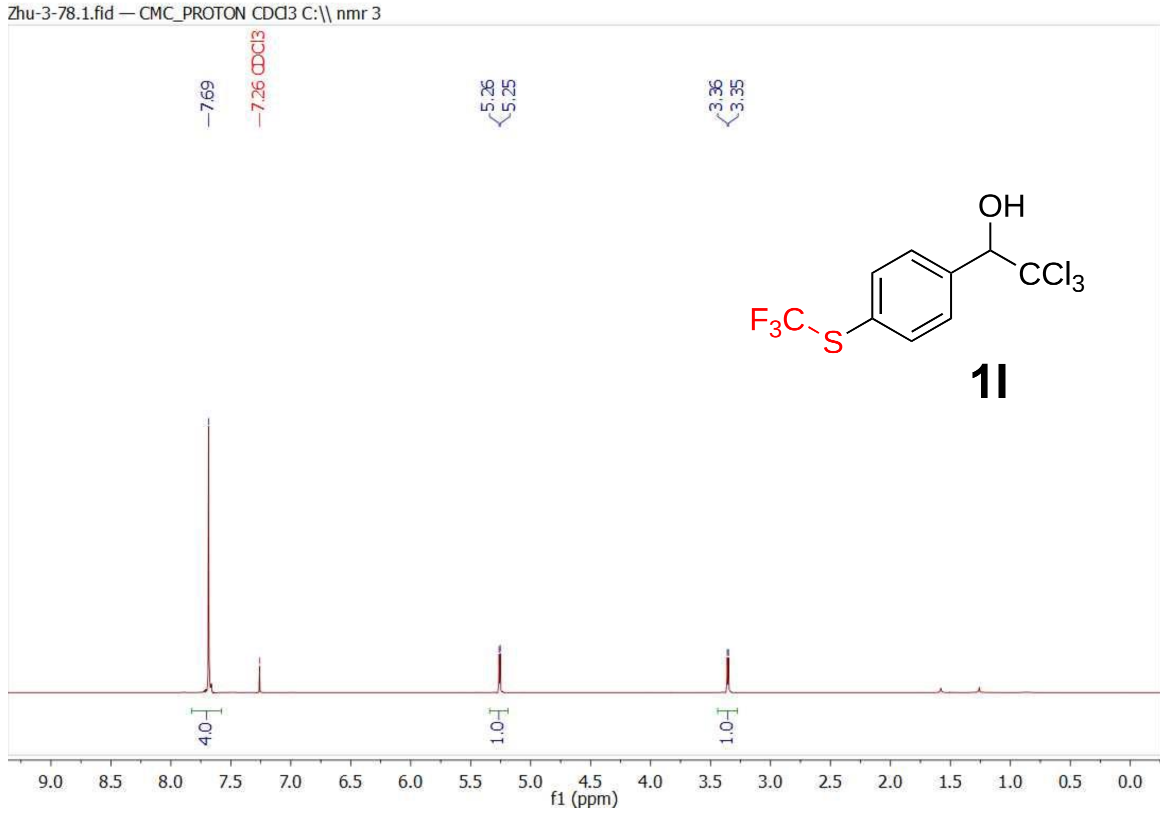

Zhu-3-78.1.fid — CMC_PROTON CDCl3 C:\\ nmr 3
7.69
7.26 CDCl3
5.26
5.25
3.36
3.35
OH
CCl3
F3C
S
1l
4.0
1.0
1.0
9.0
8.5
8.0
7.5
7.0
6.5
6.0
5.5
5.0
4.5
4.0
3.5
3.0
2.5
2.0
1.5
1.0
0.5
0.0
f1 (ppm)

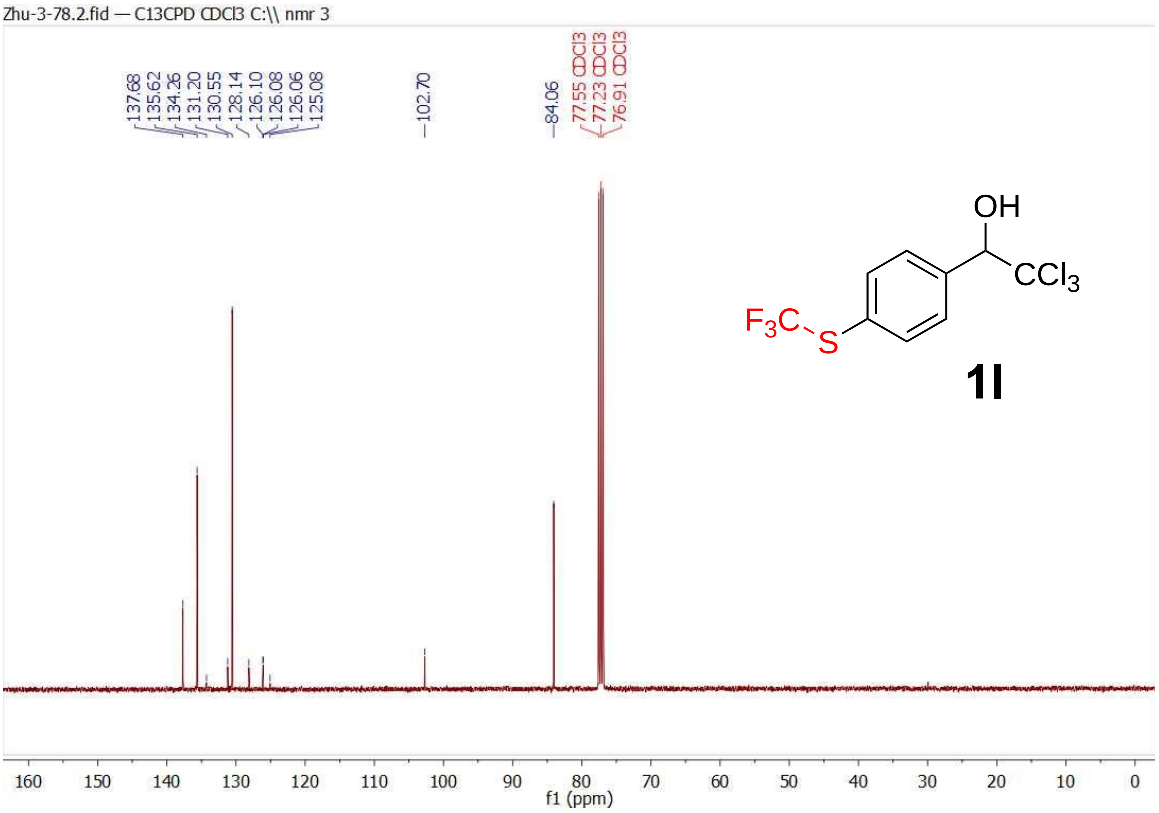
Zhu-3-78.2.fid — C13CPD CDCl3 C:\\ nmr 3
137.68
135.62
134.26
131.20
130.55
128.14
126.10
126.08
126.06
125.08
102.70
84.06
77.55 CDCl3
77.23 CDCl3
76.91 CDCl3
OH
CCl3
F3C
S
1l
160
150
140
130
120
110
100
90
80
70
60
50
40
30
20
10
0
f1 (ppm)

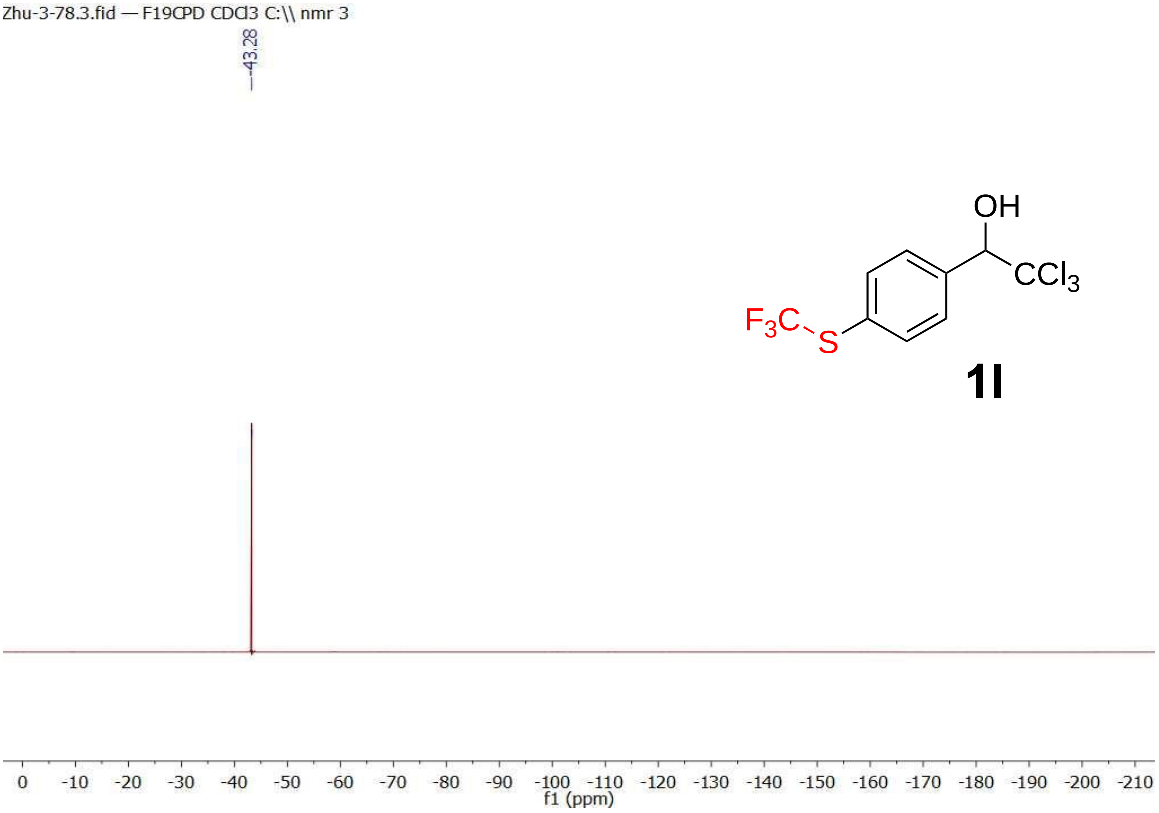
Zhu-3-78.3.fid — F19CPD CDCl3 C:\\ nmr 3
-43.28
OH
CCl3
F3C
S
1l
0
-10
-20
-30
-40
-50
-60
-70
-80
-90
-100
-110
-120
-130
-140
-150
-160
-170
-180
-190
-200
-210
f1 (ppm)

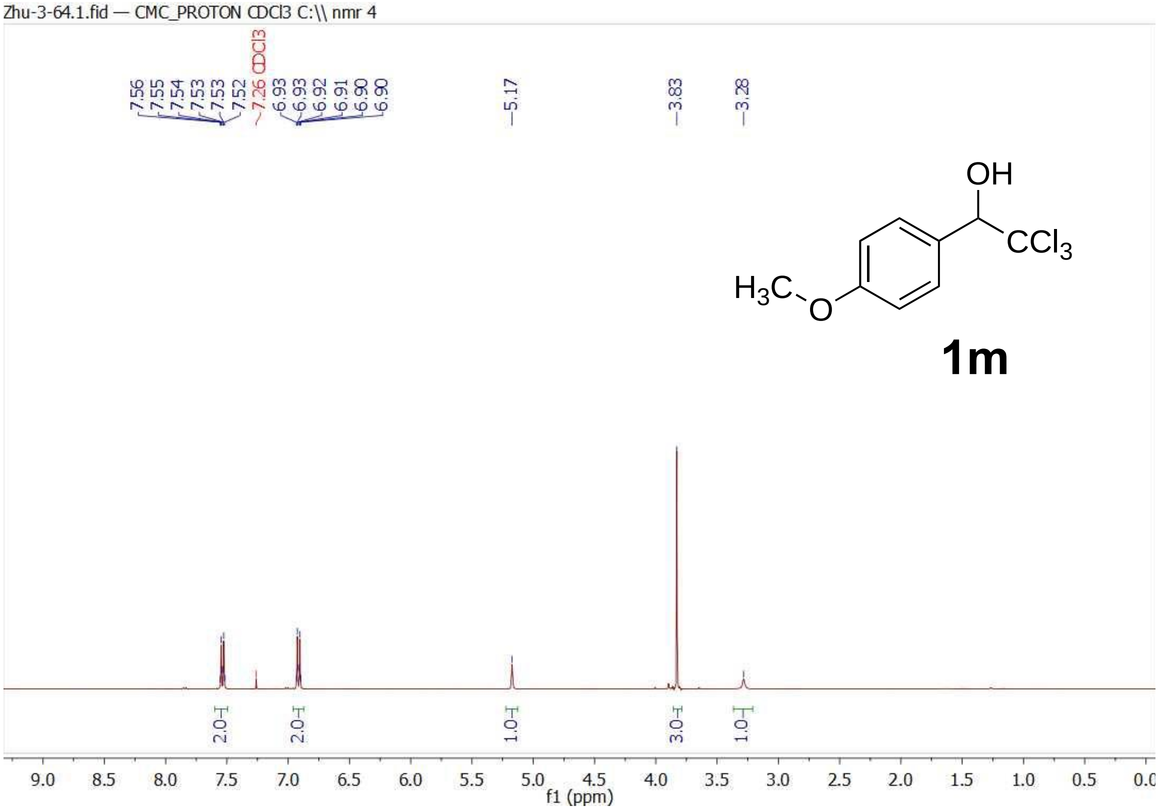
Zhu-3-64.1.fid — CMC_PROTON CDCl3 C:\\ nmr 4
7.56
7.55
7.54
7.53
7.53
7.52
7.26 CDCl3
6.93
6.93
6.92
6.91
6.90
6.90
5.17
3.83
3.28
OH
CCl3
H3C
O
1m
2.0
2.0
1.0
3.0
1.0
9.0 8.5 8.0 7.5 7.0 6.5 6.0 5.5 5.0 4.5 4.0 3.5 3.0 2.5 2.0 1.5 1.0 0.5 0.0
f1 (ppm)

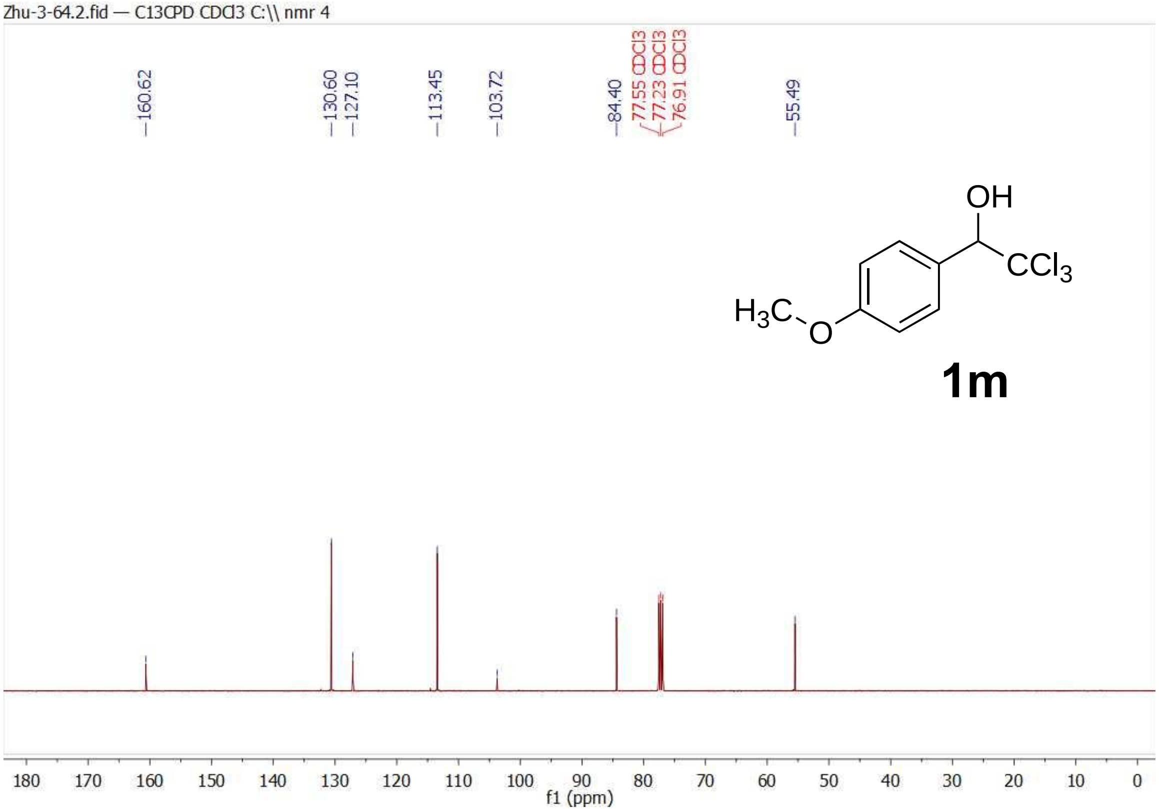
Zhu-3-64.2.fid — C13CPD CDCl3 C:\\ nmr 4
160.62
130.60
127.10
113.45
103.72
84.40
77.55 CDCl3
77.23 CDCl3
76.91 CDCl3
55.49
OH
CCl3
H3C
O
1m
180 170 160 150 140 130 120 110 100 90 80 70 60 50 40 30 20 10 0
f1 (ppm)

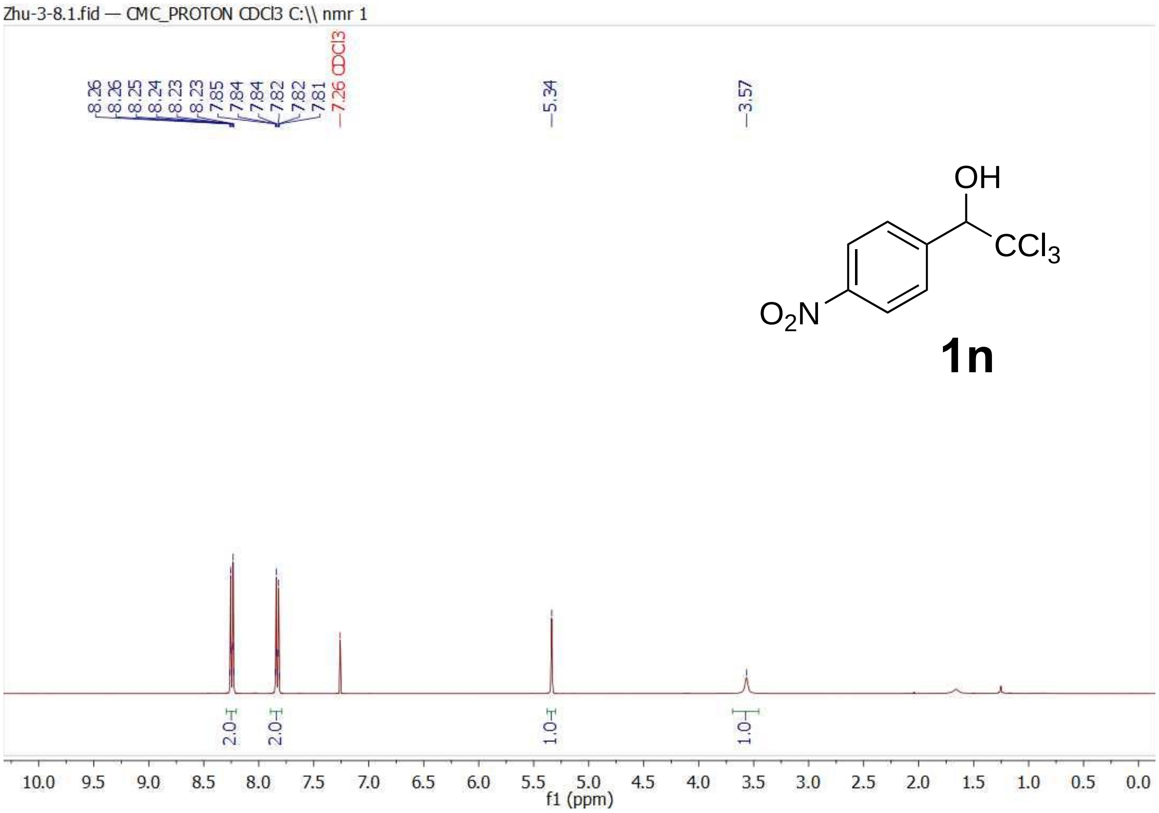
Zhu-3-8.1.fid — CMC_PROTON CDCl3 C:\\ nmr 1
8.26 8.26 8.25 8.24 8.23 8.23 7.85 7.84 7.84 7.82 7.82 7.81
7.26 CDCl3
5.34
3.57
OH
CCl3
O2N
1n
2.0 2.0 1.0 1.0
10.0 9.5 9.0 8.5 8.0 7.5 7.0 6.5 6.0 5.5 5.0 4.5 4.0 3.5 3.0 2.5 2.0 1.5 1.0 0.5 0.0
f1 (ppm)

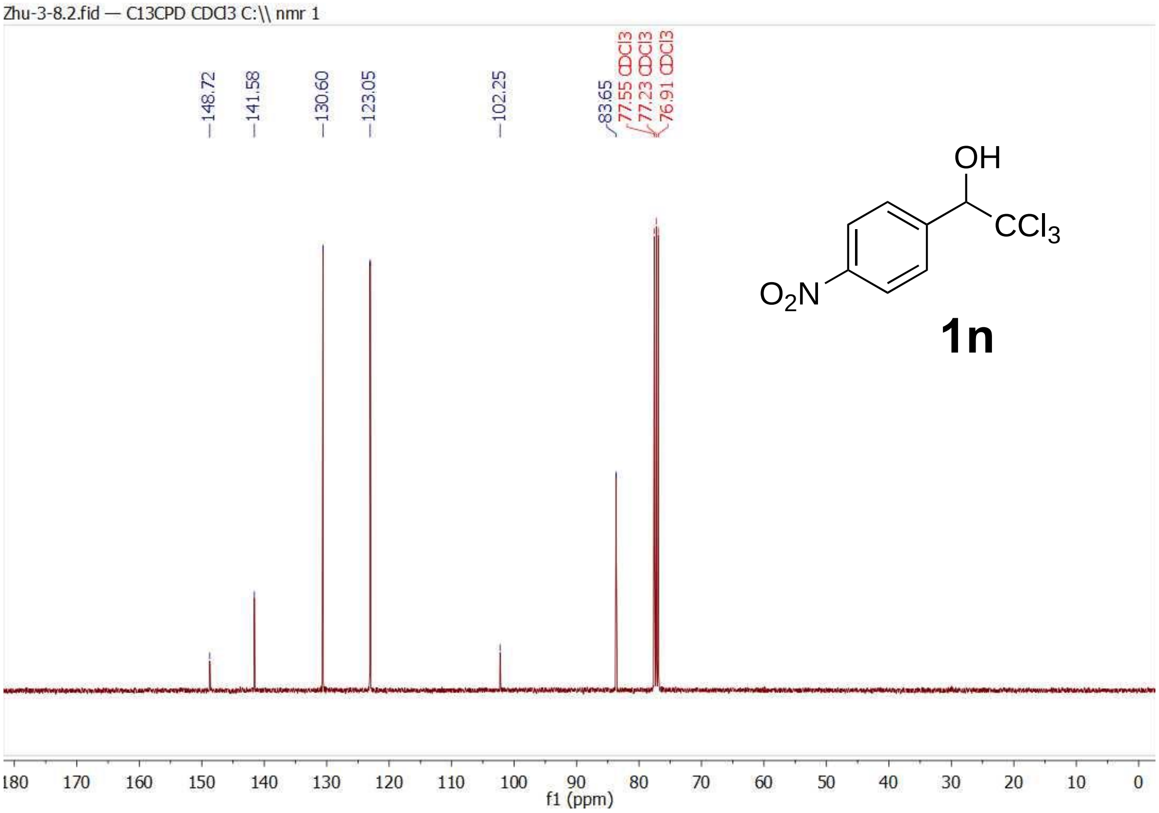
Zhu-3-8.2.fid — C13CPD CDCl3 C:\\ nmr 1
148.72
141.58
130.60
123.05
102.25
83.65
77.55 CDCl3
77.23 CDCl3
76.91 CDCl3
OH
CCl3
O2N
1n
180 170 160 150 140 130 120 110 100 90 80 70 60 50 40 30 20 10 0
f1 (ppm)

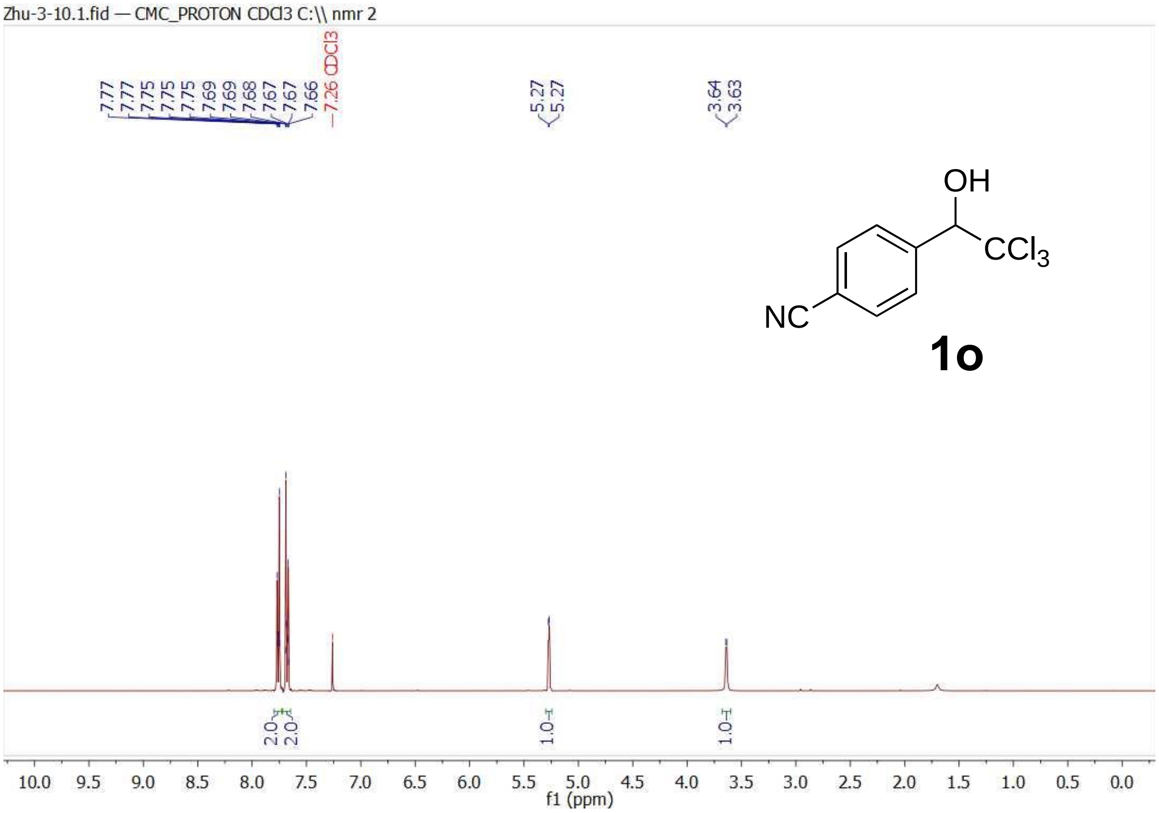
Zhu-3-10.1.fid — CMC_PROTON CDCl3 C:\\ nmr 2
7.77
7.77
7.75
7.75
7.75
7.69
7.69
7.68
7.67
7.67
7.66
7.26 CDCl3
5.27
5.27
3.64
3.63
OH
CCl3
NC
1o
2.0
2.0
1.0
1.0
10.0 9.5 9.0 8.5 8.0 7.5 7.0 6.5 6.0 5.5 5.0 4.5 4.0 3.5 3.0 2.5 2.0 1.5 1.0 0.5 0.0
f1 (ppm)

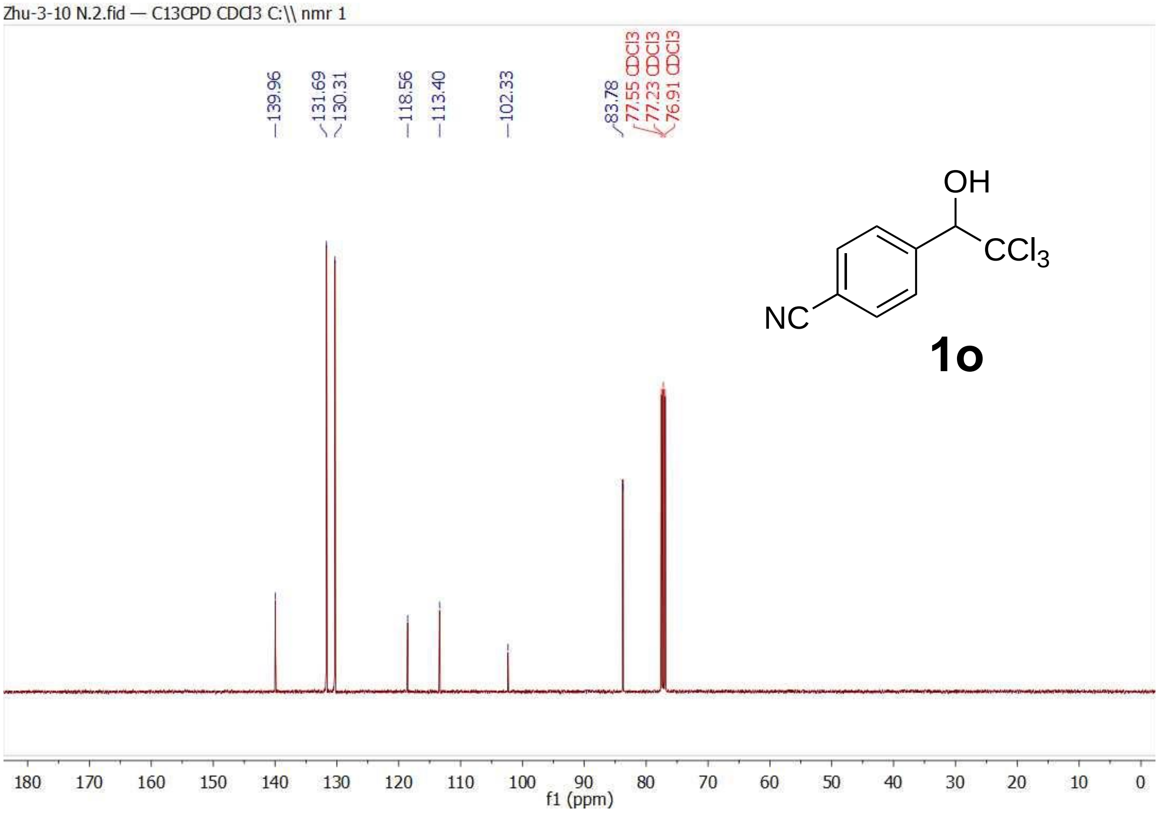
Zhu-3-10 N.2.fid — C13CPD CDCl3 C:\\ nmr 1
139.96
131.69
130.31
118.56
113.40
102.33
83.78
77.55 CDCl3
77.23 CDCl3
76.91 CDCl3
OH
CCl3
NC
1o
180 170 160 150 140 130 120 110 100 90 80 70 60 50 40 30 20 10 0
f1 (ppm)

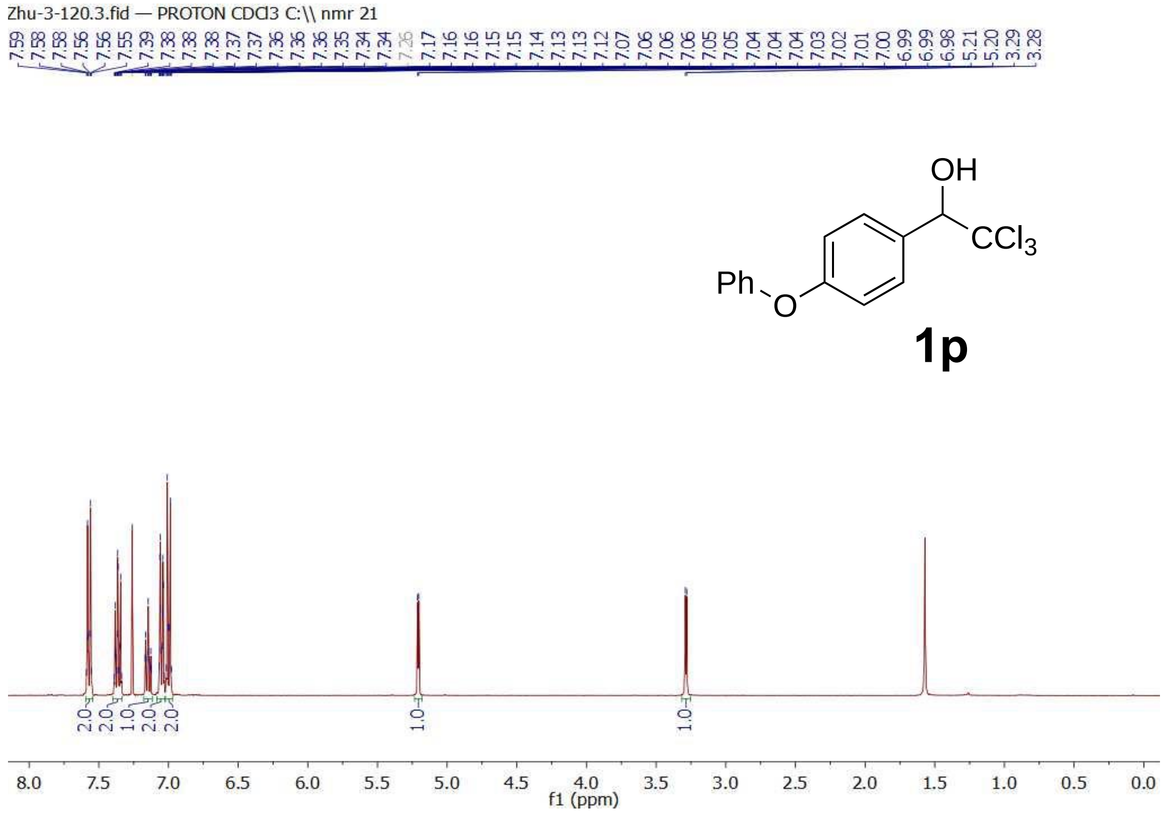
Zhu-3-120.3.fid — PROTON CDCl3 C:\\ nmr 21
7.59 7.58 7.58 7.56 7.56 7.55 7.39 7.38 7.38 7.38 7.37 7.37 7.36 7.36 7.36 7.35 7.34 7.34 7.26 7.17 7.16 7.16 7.15 7.15 7.14 7.13 7.13 7.12 7.07 7.06 7.06 7.06 7.05 7.05 7.04 7.04 7.04 7.03 7.02 7.01 7.00 6.99 6.99 6.98 5.21 5.20 3.29 3.28
OH
CCl3
Ph
O
1p
2.0 2.0 1.0 2.0 2.0 1.0 1.0
f1 (ppm)

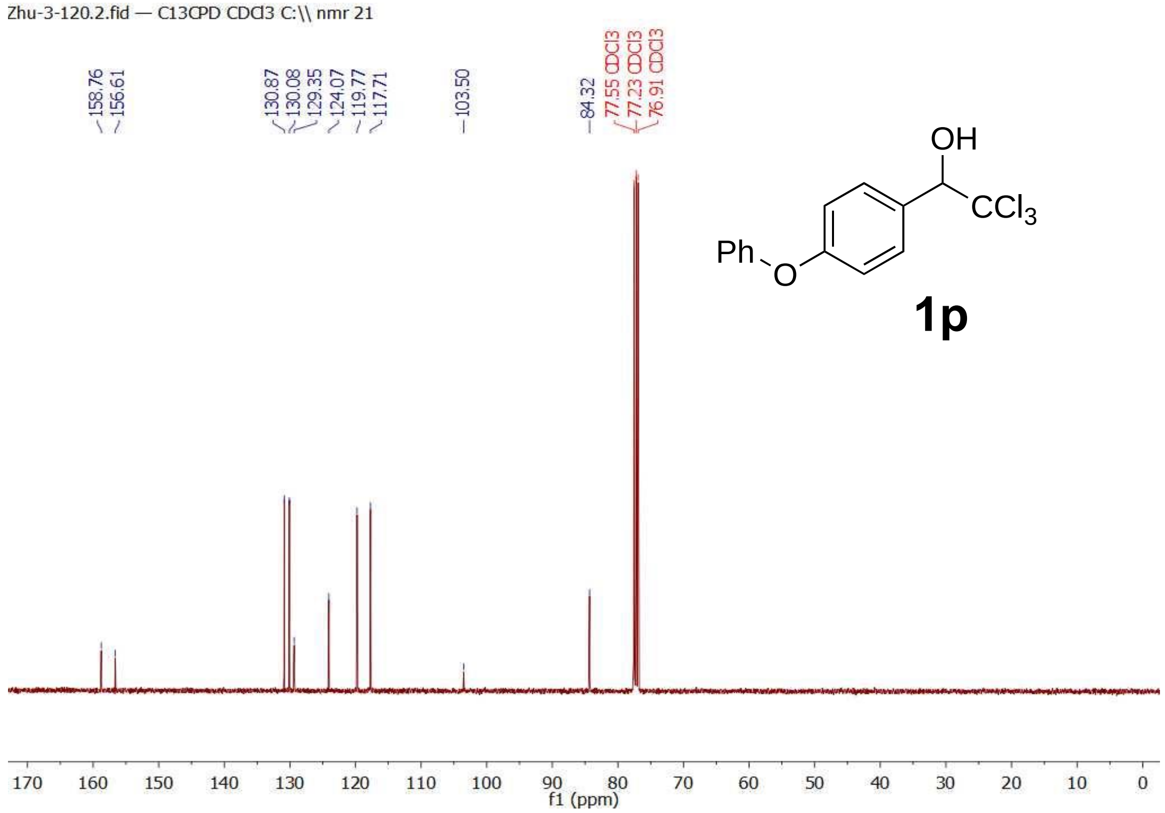
Zhu-3-120.2.fid — C13CPD CDCl3 C:\\ nmr 21
158.76 156.61 130.87 130.08 129.35 124.07 119.77 117.71 103.50 84.32 77.55 CDCl3 77.23 CDCl3 76.91 CDCl3
OH
CCl3
Ph
O
1p
f1 (ppm)

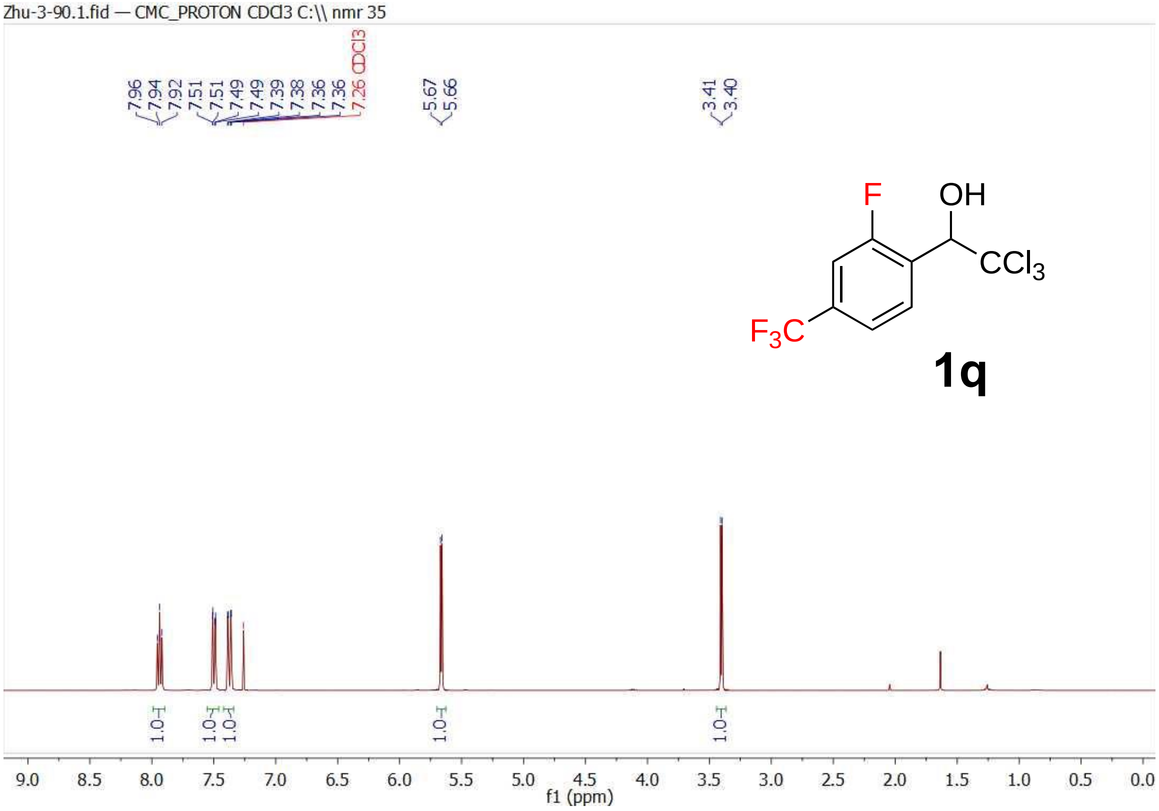
Zhu-3-90.1.fid — CMC_PROTON CDCl3 C:\\ nmr 35
7.96
7.94
7.92
7.51
7.51
7.49
7.49
7.39
7.38
7.36
7.36
7.26 CDCl3
5.67
5.66
3.41
3.40
F
OH
CCl3
F3C
1q
1.0
1.0
1.0
1.0
1.0
f1 (ppm)

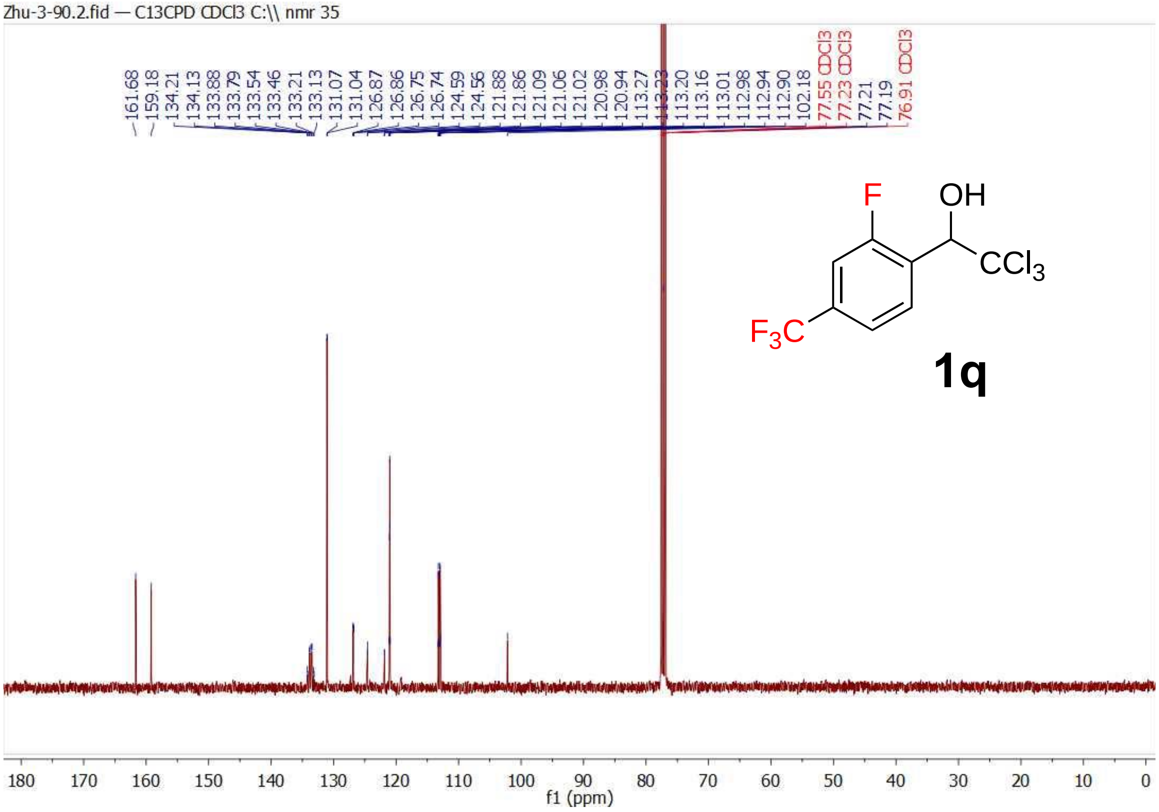
Zhu-3-90.2.fid — C13CPD CDCl3 C:\\ nmr 35
161.68
159.18
134.21
134.13
133.88
133.79
133.54
133.46
133.21
133.13
131.07
131.04
126.87
126.86
126.75
126.74
124.59
124.56
121.88
121.86
121.09
121.06
121.02
120.98
120.94
113.27
113.23
113.20
113.16
113.01
112.98
112.94
112.90
102.18
77.55 CDCl3
77.23 CDCl3
77.21
77.19
76.91 CDCl3
F
OH
CCl3
F3C
1q
f1 (ppm)

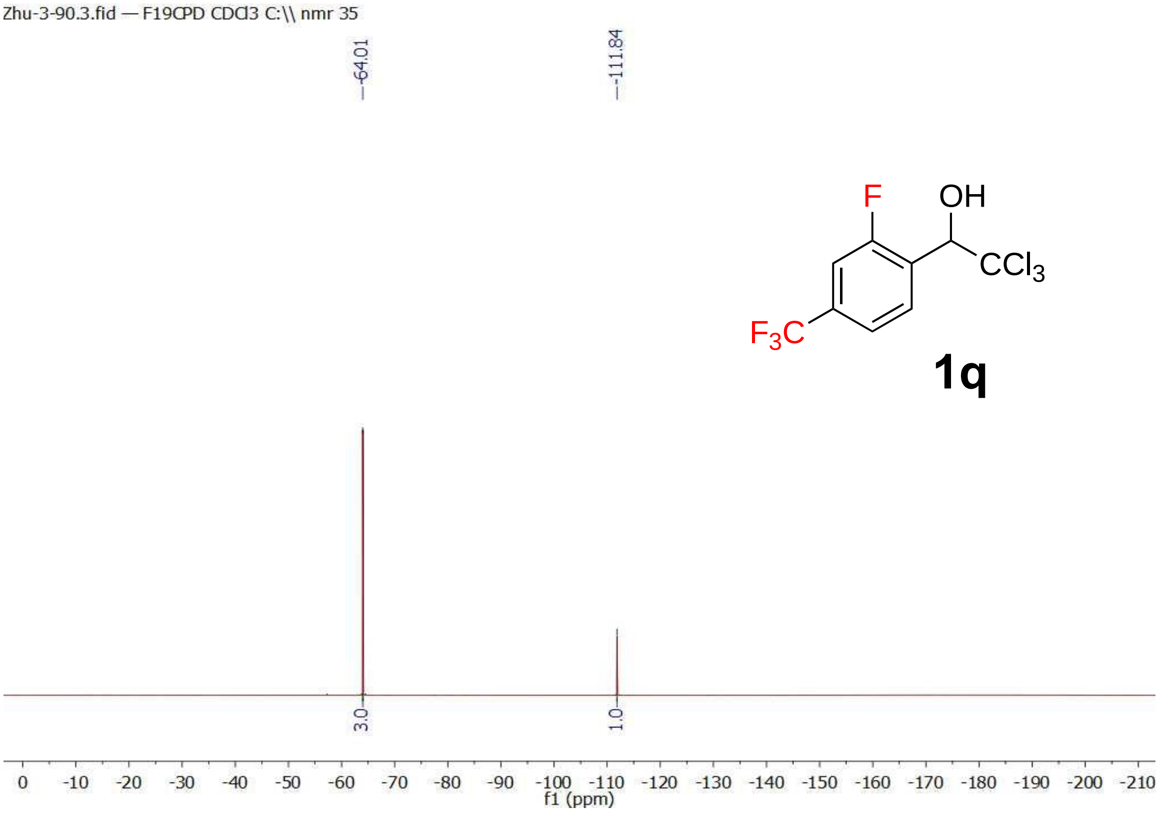
Zhu-3-90.3.fid — F19CPD CDCl3 C:\\ nmr 35
-64.01
-111.84
F
OH
CCl3
F3C
1q
3.0
1.0
0 -10 -20 -30 -40 -50 -60 -70 -80 -90 -100 -110 -120 -130 -140 -150 -160 -170 -180 -190 -200 -210
f1 (ppm)

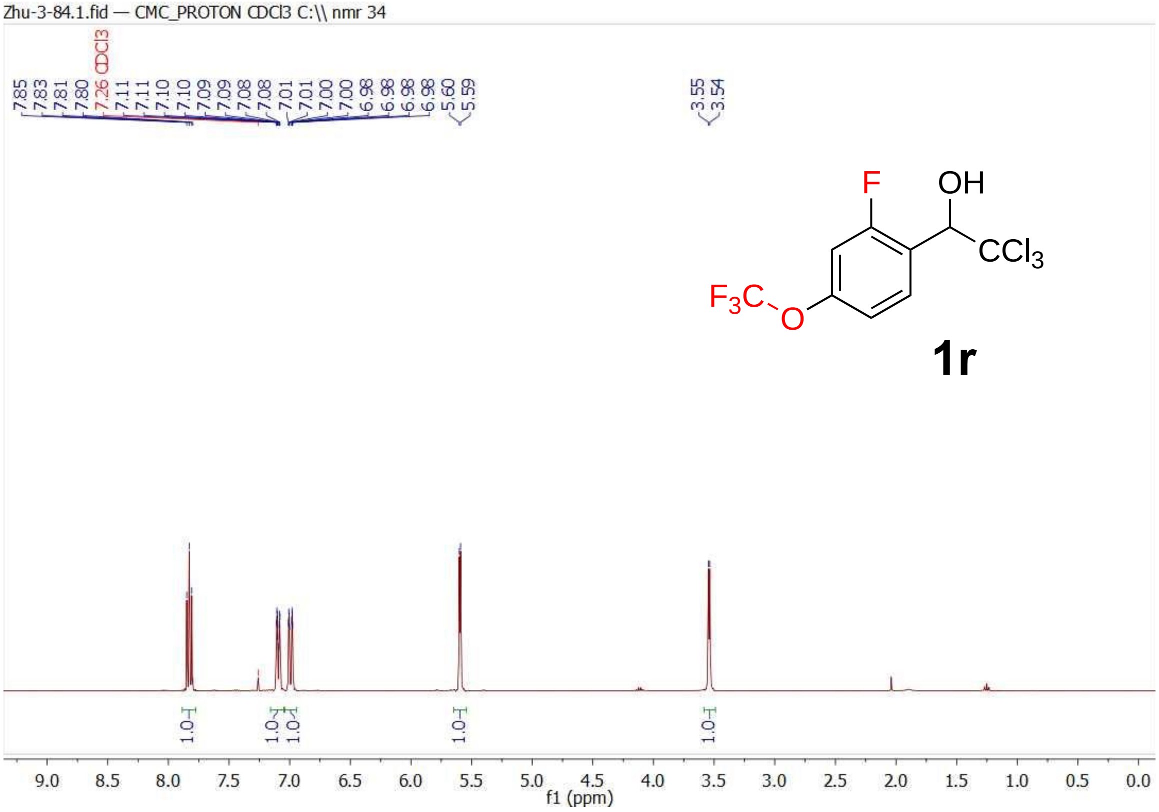
Zhu-3-84.1.fid — CMC_PROTON CDCl3 C:\\ nmr 34
7.85 7.83 7.81 7.80 7.26 CDCl3 7.11 7.11 7.10 7.10 7.09 7.09 7.08 7.08 7.01 7.01 7.00 7.00 6.98 6.98 6.98 6.98 5.60 5.59
3.55 3.54
F
OH
CCl3
F3C
O
1r
1.0 1.0 1.0 1.0 1.0
9.0 8.5 8.0 7.5 7.0 6.5 6.0 5.5 5.0 4.5 4.0 3.5 3.0 2.5 2.0 1.5 1.0 0.5 0.0
f1 (ppm)

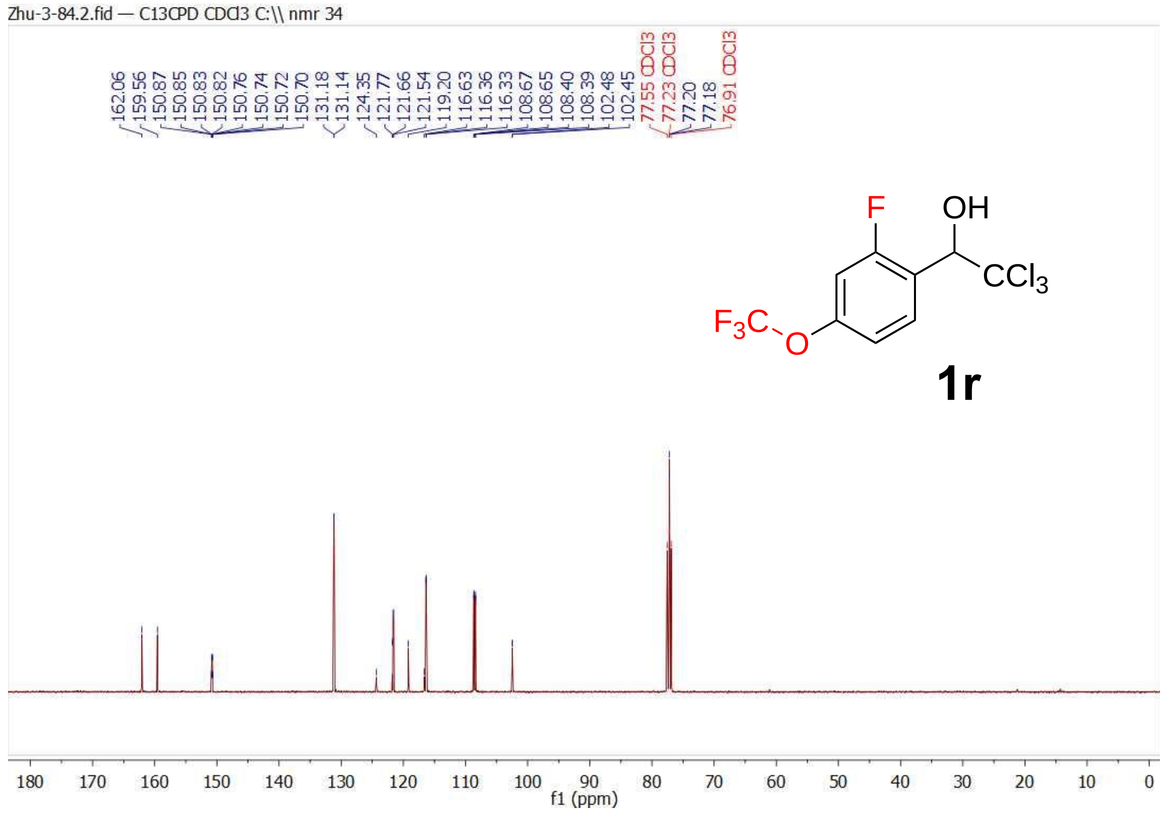
Zhu-3-84.2.fid — C13CPD CDCl3 C:\\ nmr 34
162.06
159.56
150.87
150.85
150.83
150.82
150.76
150.74
150.72
150.70
131.18
131.14
124.35
121.77
121.66
121.54
119.20
116.63
116.36
116.33
108.67
108.65
108.40
108.39
102.48
102.45
77.55 CDCl3
77.23 CDCl3
77.20
77.18
76.91 CDCl3
F
OH
CCl3
F3C
O
1r
f1 (ppm)

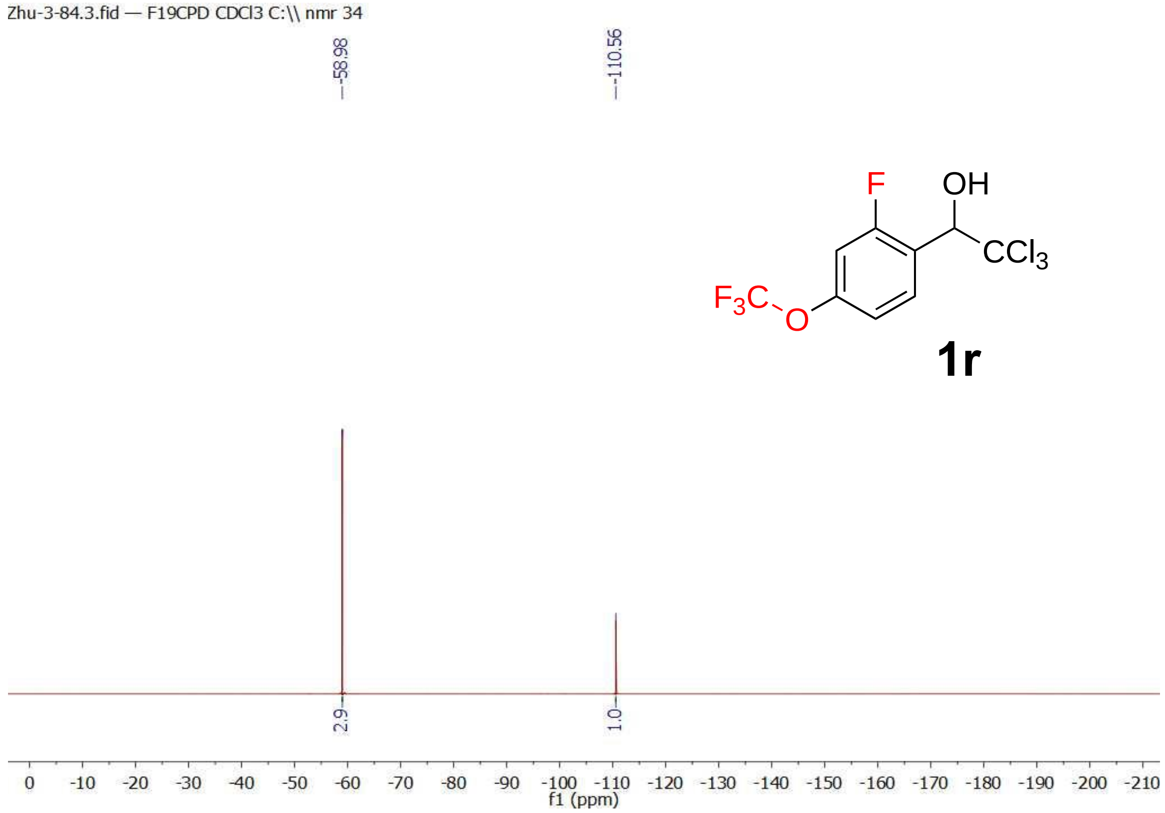
Zhu-3-84.3.fid — F19CPD CDCl3 C:\\ nmr 34
-58.98
-110.56
F
OH
CCl3
F3C
O
1r
2.9
1.0
f1 (ppm)

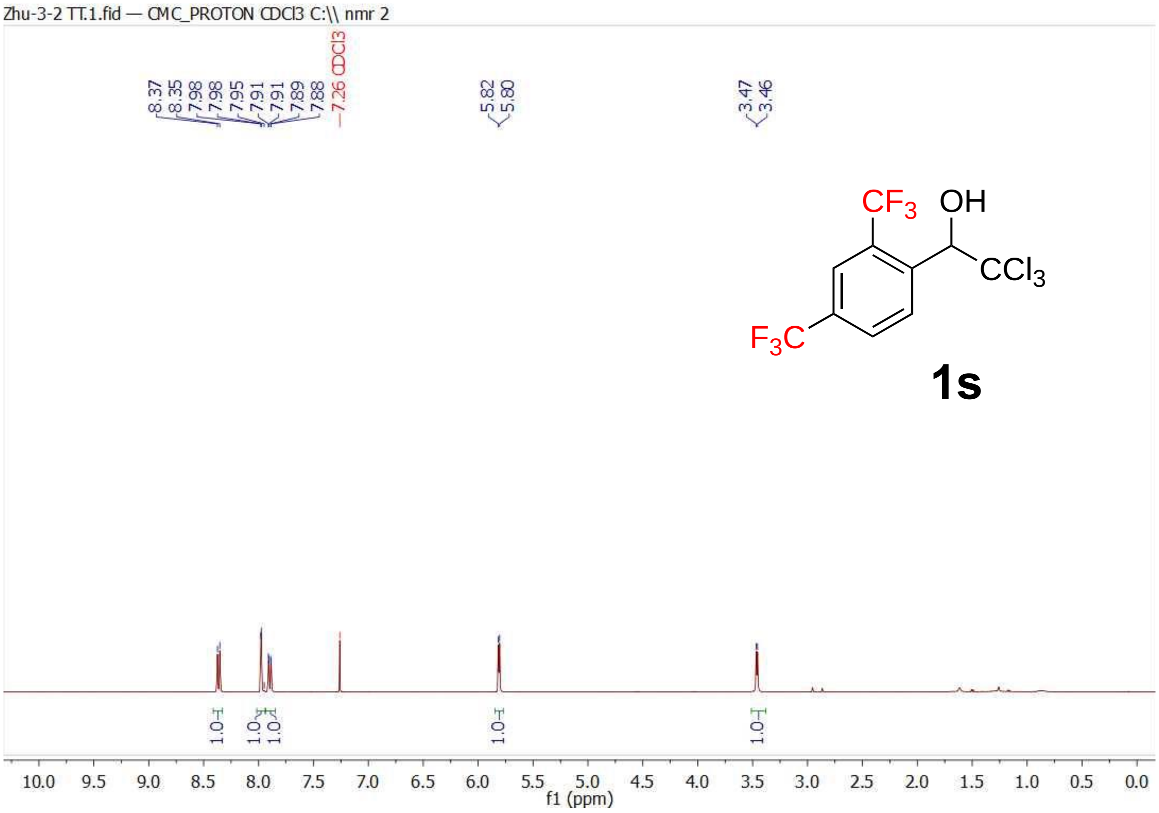
Zhu-3-2 TT.1.fid — CMC_PROTON CDCl3 C:\\ nmr 2
8.37
8.35
7.98
7.98
7.95
7.91
7.91
7.89
7.88
7.26 CDCl3
5.82
5.80
3.47
3.46
CF3
OH
CCl3
F3C
1s
1.0
1.0
1.0
1.0
1.0
10.0 9.5 9.0 8.5 8.0 7.5 7.0 6.5 6.0 5.5 5.0 4.5 4.0 3.5 3.0 2.5 2.0 1.5 1.0 0.5 0.0
f1 (ppm)

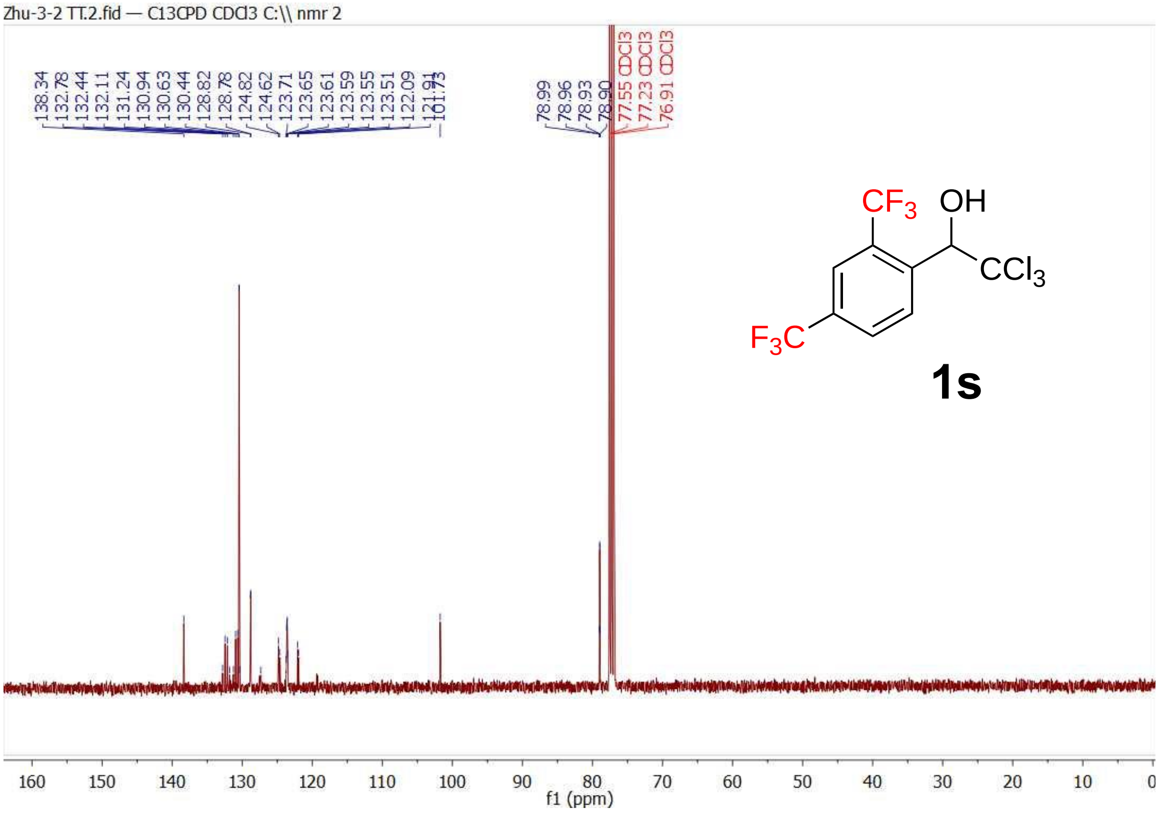
Zhu-3-2 TT.2.fid — C13CPD CDCl3 C:\\ nmr 2
138.34
132.78
132.44
132.11
131.24
130.94
130.63
130.44
128.82
128.78
124.82
124.62
123.71
123.65
123.61
123.59
123.55
123.51
122.09
121.91
101.73
78.99
78.96
78.93
78.90
77.55 CDCl3
77.23 CDCl3
76.91 CDCl3
CF3
OH
CCl3
F3C
1s
160 150 140 130 120 110 100 90 80 70 60 50 40 30 20 10 0
f1 (ppm)

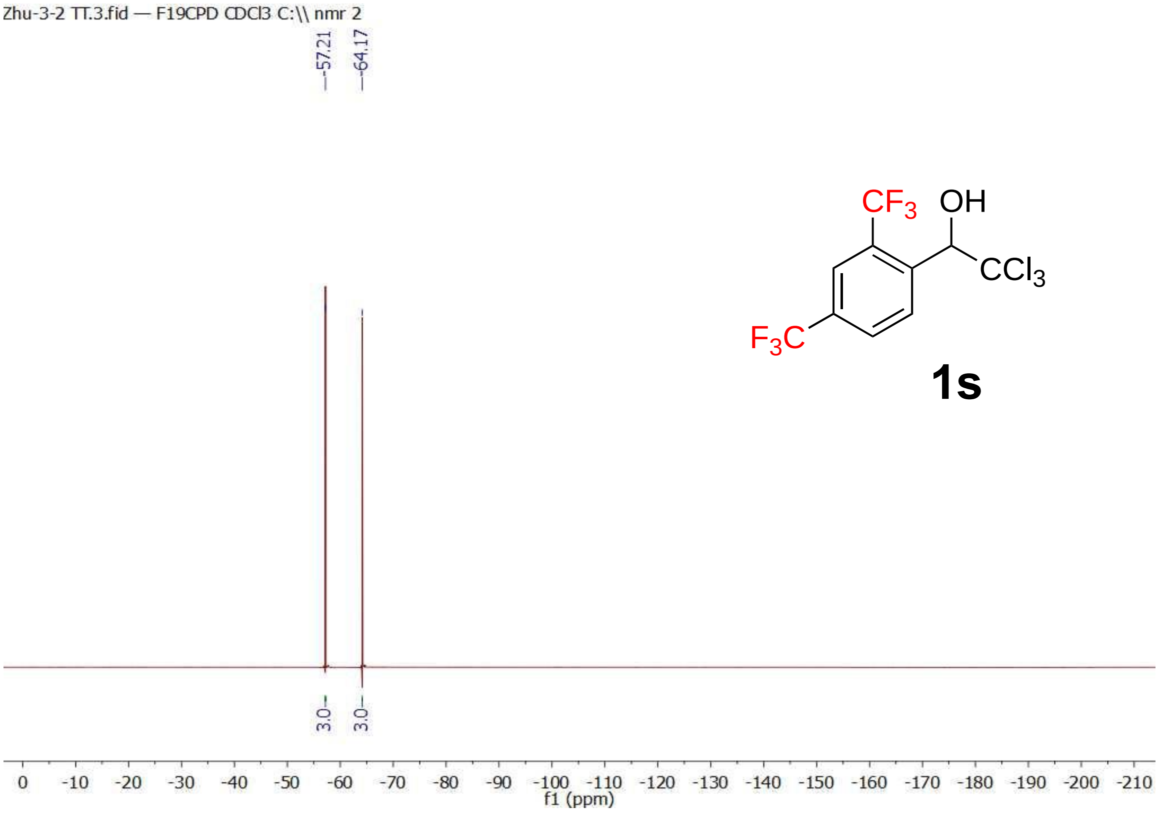
Zhu-3-2 TT.3.fid — F19CPD CDCl3 C:\\ nmr 2
-57.21
-64.17
CF3
OH
CCl3
F3C
1s
3.0
3.0
0 -10 -20 -30 -40 -50 -60 -70 -80 -90 -100 -110 -120 -130 -140 -150 -160 -170 -180 -190 -200 -210
f1 (ppm)

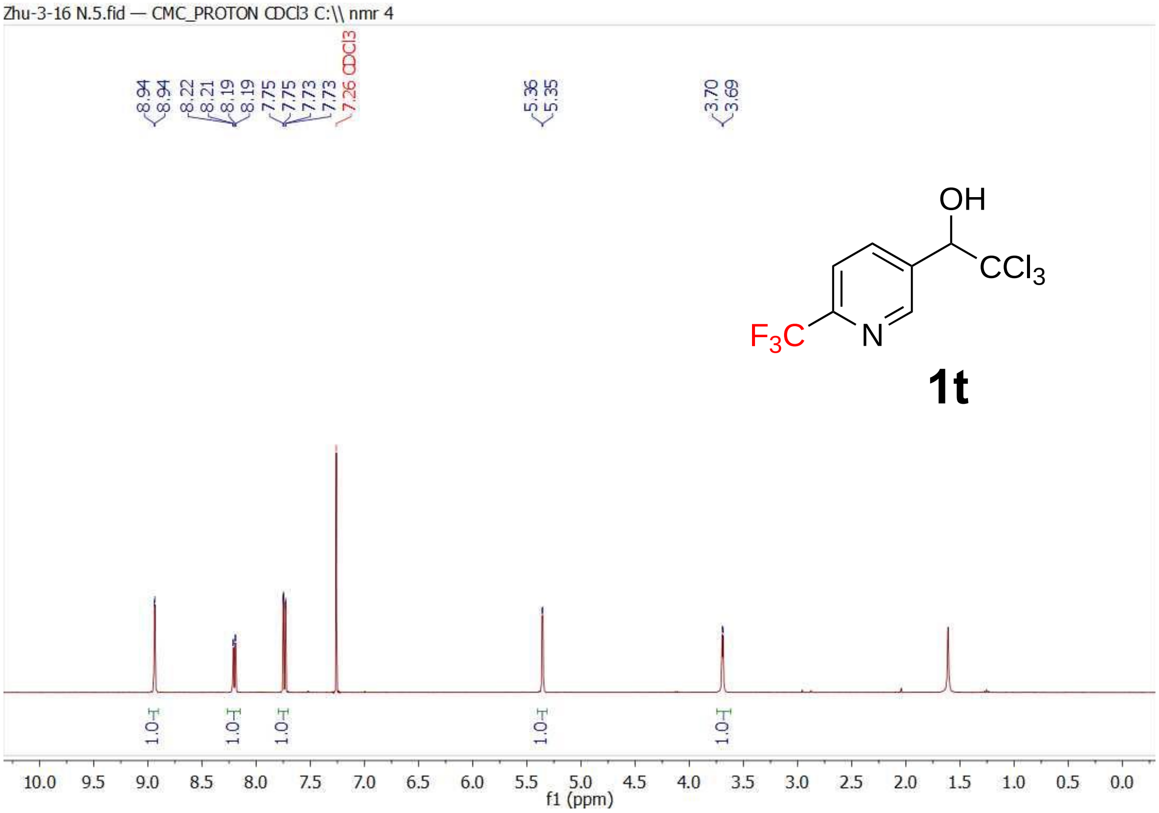
Zhu-3-16 N.5.fid — CMC_PROTON CDCl3 C:\\ nmr 4
8.94
8.94
8.22
8.21
8.19
8.19
7.75
7.75
7.73
7.73
7.26 CDCl3
5.36
5.35
3.70
3.69
OH
CCl3
F3C
N
1t
1.0
1.0
1.0
1.0
1.0
10.0 9.5 9.0 8.5 8.0 7.5 7.0 6.5 6.0 5.5 5.0 4.5 4.0 3.5 3.0 2.5 2.0 1.5 1.0 0.5 0.0
f1 (ppm)

Zhu-3-16.6.fid — C13CPD CDCl3 C:\\ nmr 4

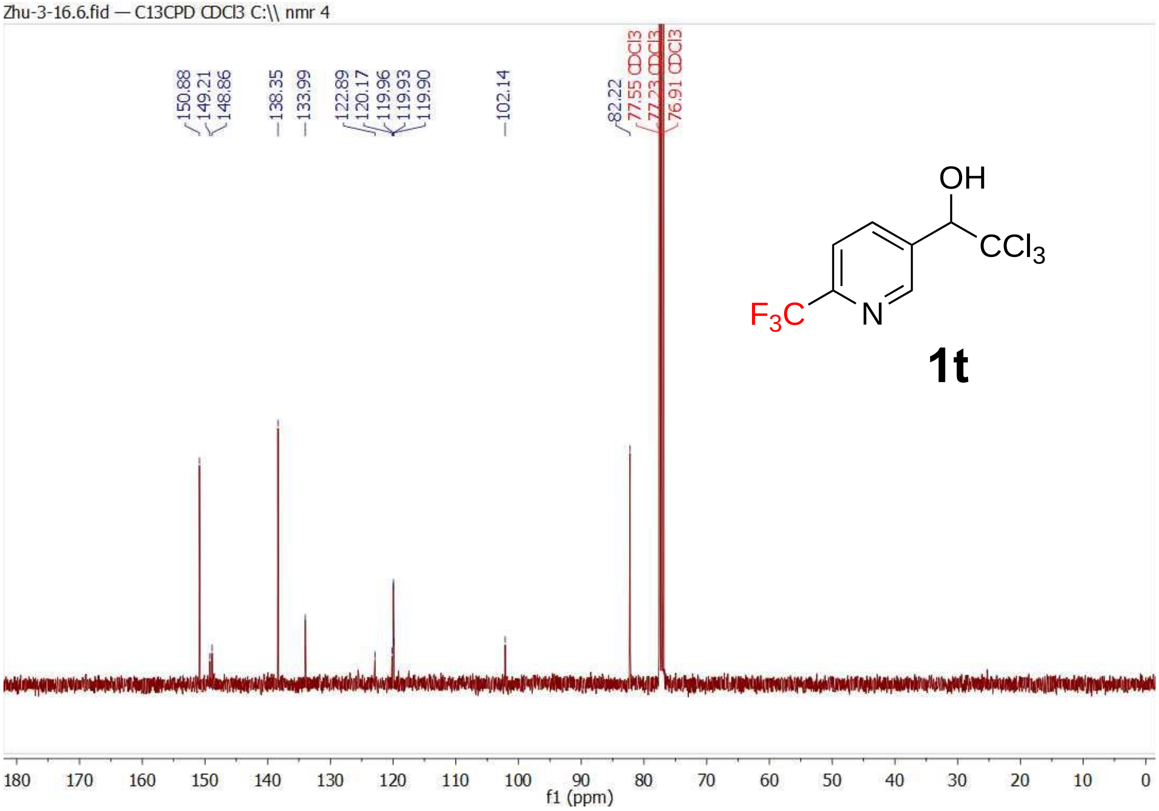

1t

Zhu-3-16.3.fid — F19CPD CDCl3 C:\\ nmr 2

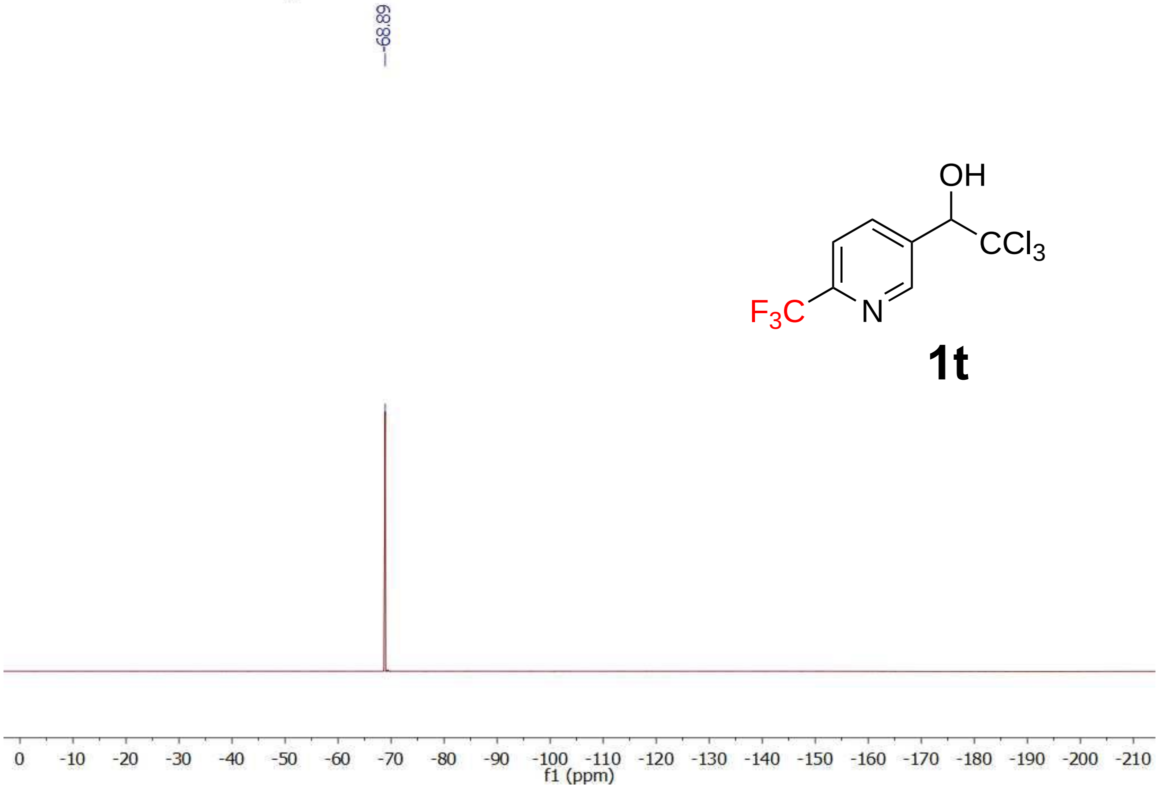

1t

Zhu-3-128.1.fid — CMC_PROTON CDCl3 C:\\ nmr 1

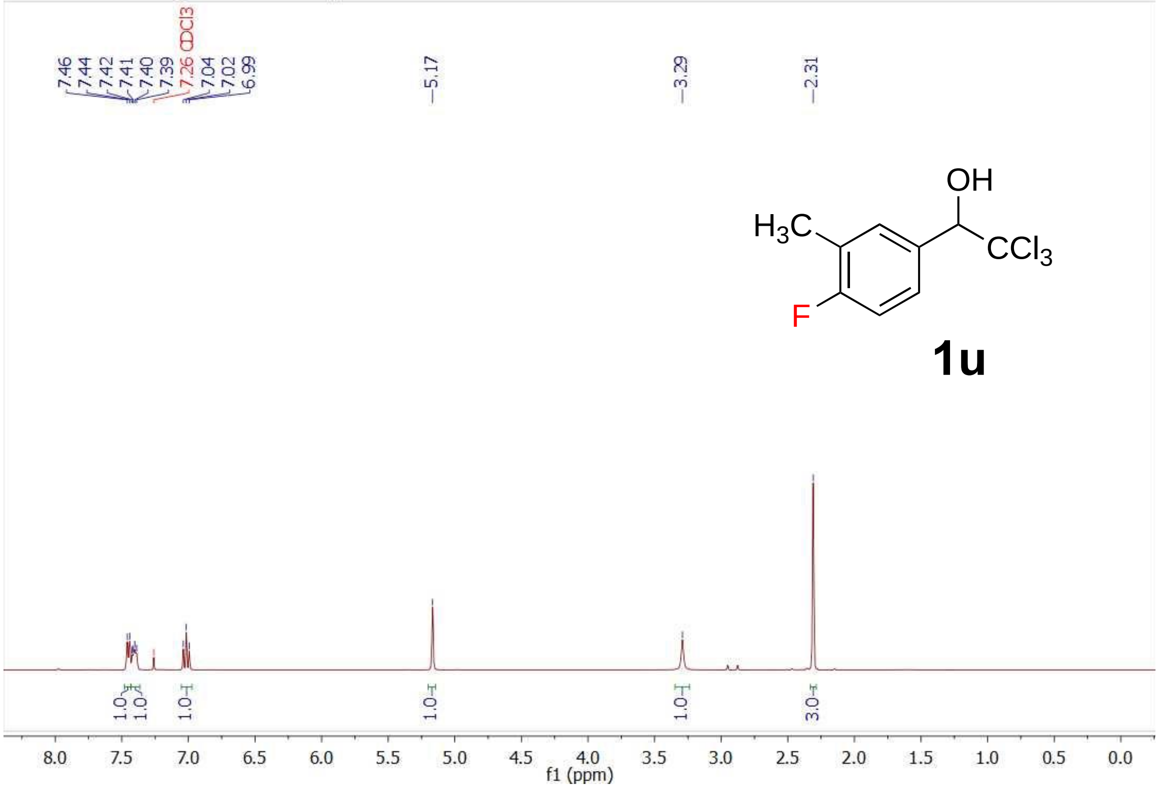

Zhu-3-128.2.fid — C13CPD CDCl3 C:\\ nmr 1

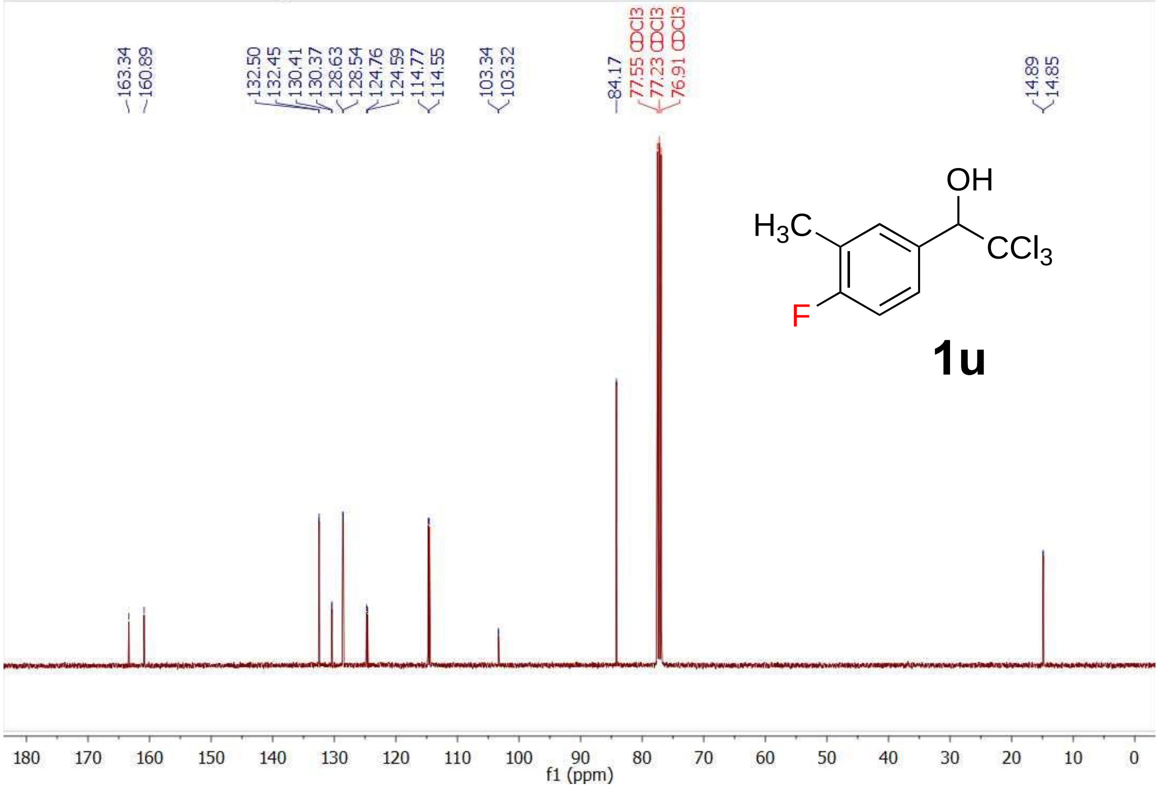

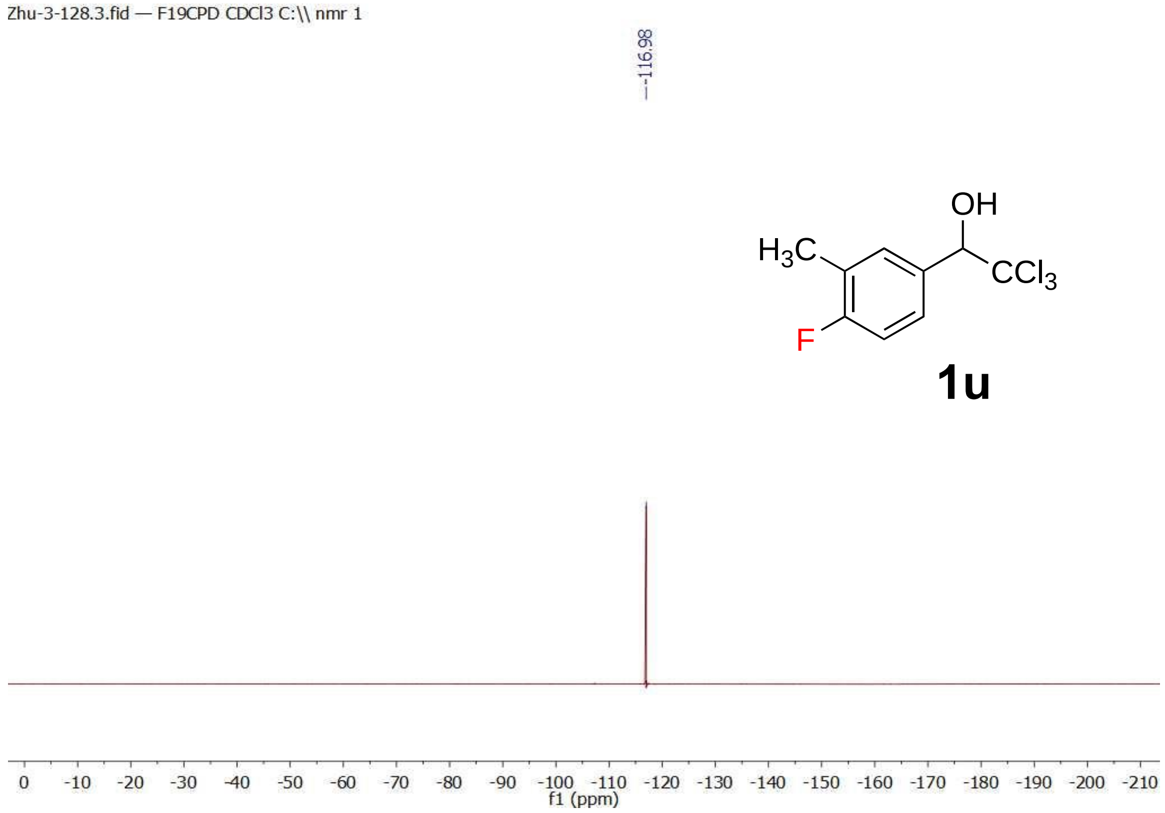
Zhu-3-128.3.fid — F19CPD CDCl3 C:\\ nmr 1
-116.98
OH
H3C
CCl3
F
1u
0 -10 -20 -30 -40 -50 -60 -70 -80 -90 -100 -110 -120 -130 -140 -150 -160 -170 -180 -190 -200 -210
f1 (ppm)

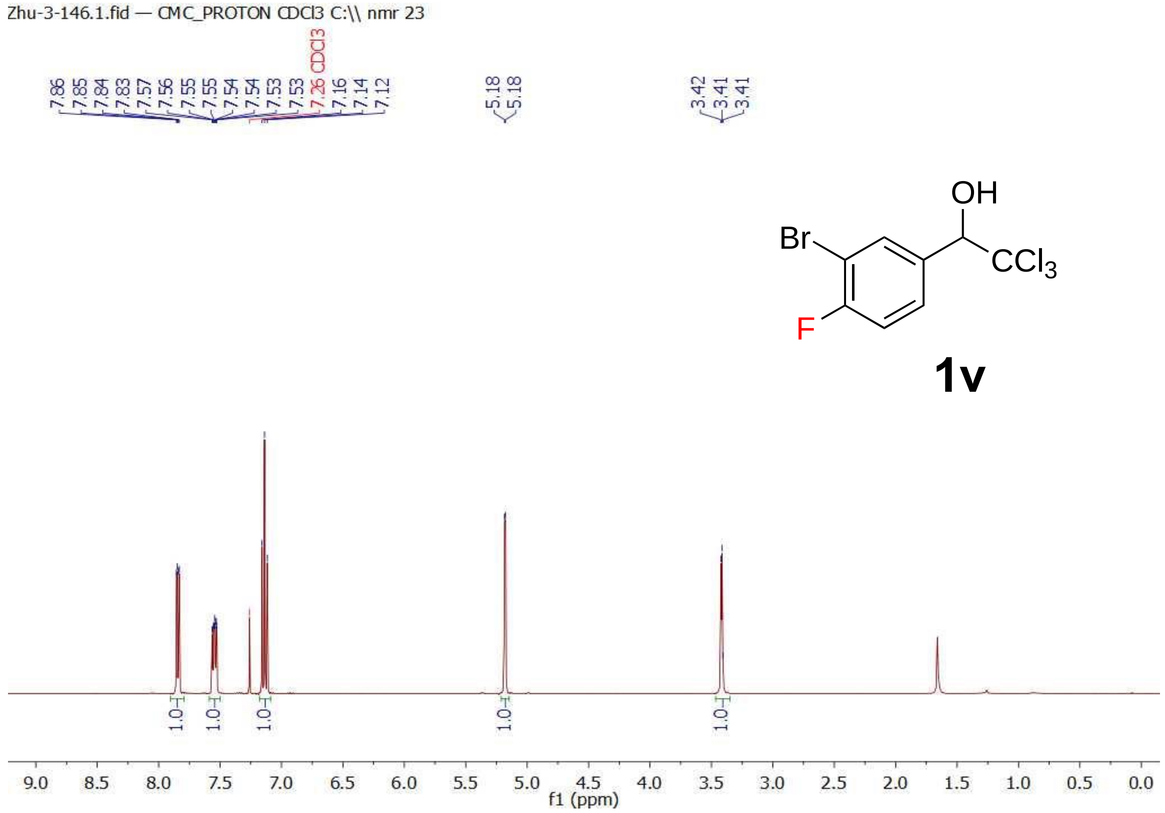
Zhu-3-146.1.fid — CMC_PROTON CDCl3 C:\\ nmr 23
7.86 7.85 7.84 7.83 7.57 7.56 7.55 7.55 7.54 7.54 7.53 7.53 7.26 CDCl3 7.16 7.14 7.12
5.18 5.18
3.42 3.41 3.41
OH
Br
CCl3
F
1v
1.0 1.0 1.0 1.0 1.0
9.0 8.5 8.0 7.5 7.0 6.5 6.0 5.5 5.0 4.5 4.0 3.5 3.0 2.5 2.0 1.5 1.0 0.5 0.0
f1 (ppm)

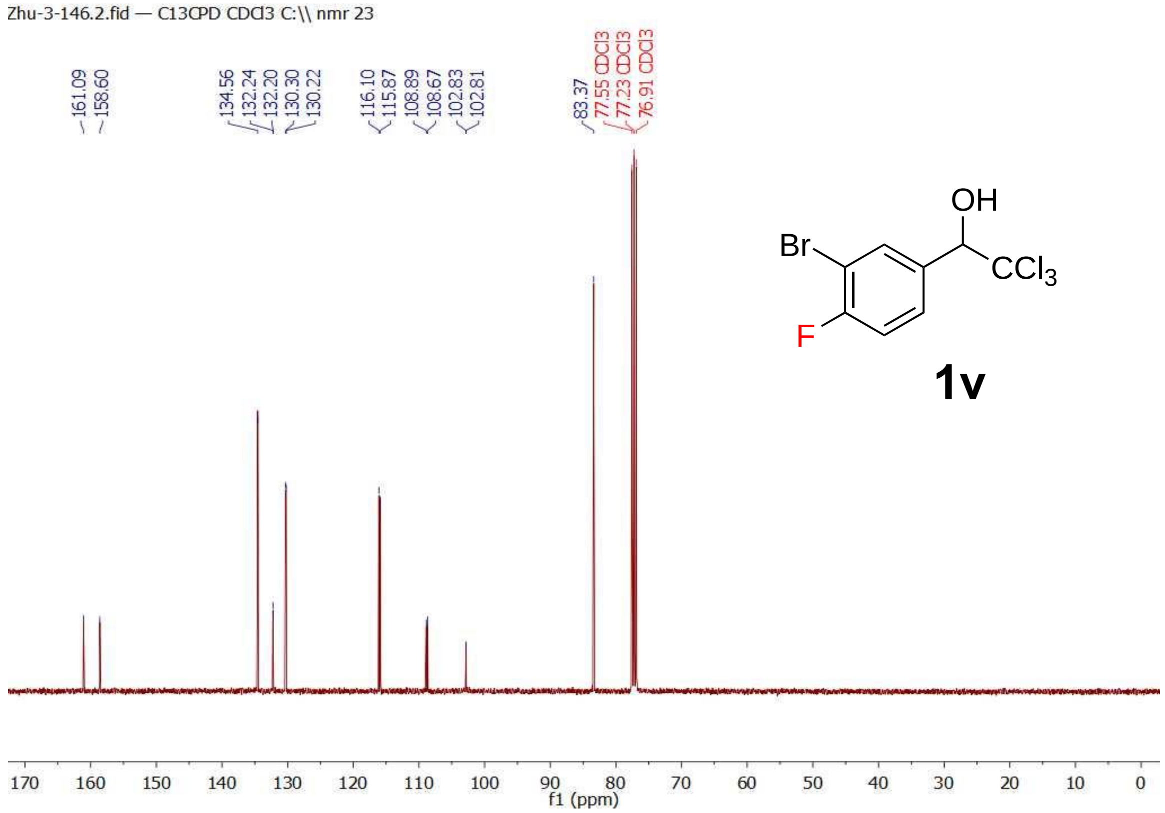
Zhu-3-146.2.fid — C13CPD CDCl3 C:\\ nmr 23
161.09
158.60
134.56
132.24
132.20
130.30
130.22
116.10
115.87
108.89
108.67
102.83
102.81
83.37
77.55 CDCl3
77.23 CDCl3
76.91 CDCl3
OH
Br
CCl3
F
1v
f1 (ppm)

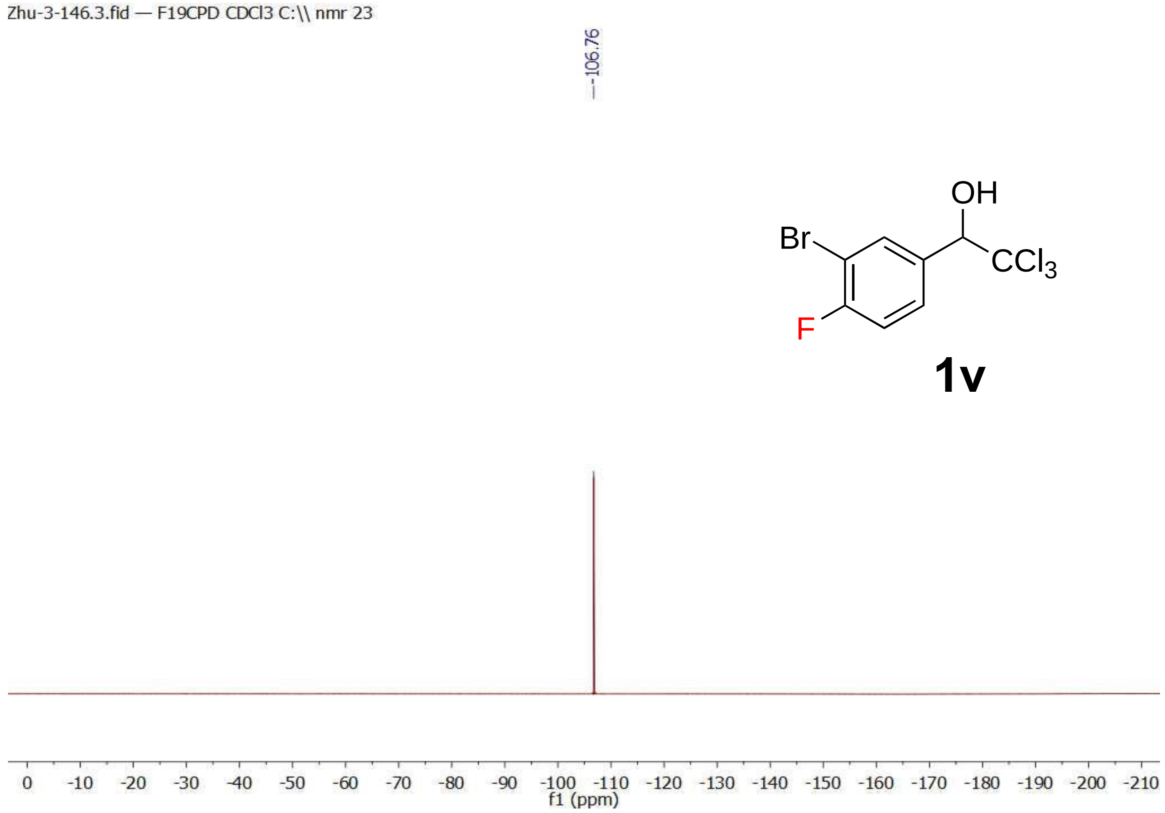
Zhu-3-146.3.fid — F19CPD CDCl3 C:\\ nmr 23
-106.76
OH
Br
CCl3
F
1v
f1 (ppm)

Zhu-3-114.1.fid — CMC_PROTON CDCl3 C:\\ nmr 5

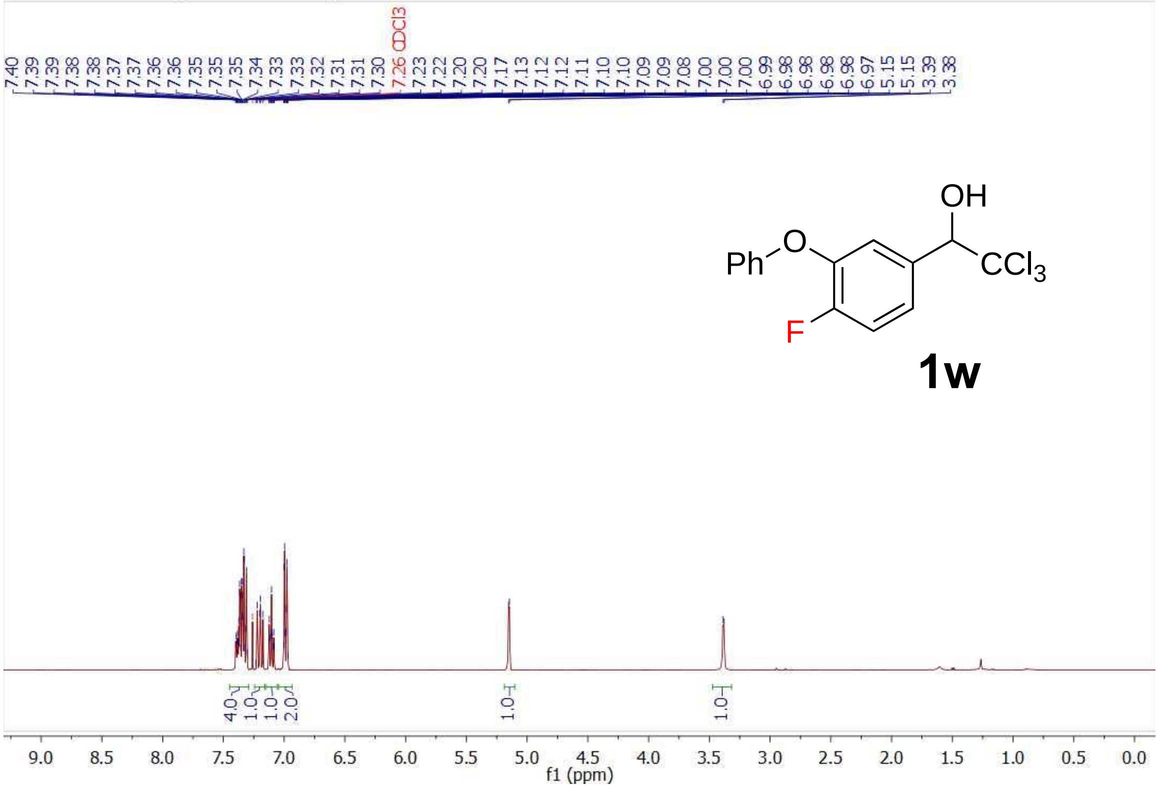

Zhu-3-114.2.fid — C13CPD CDCl3 C:\\ nmr 5

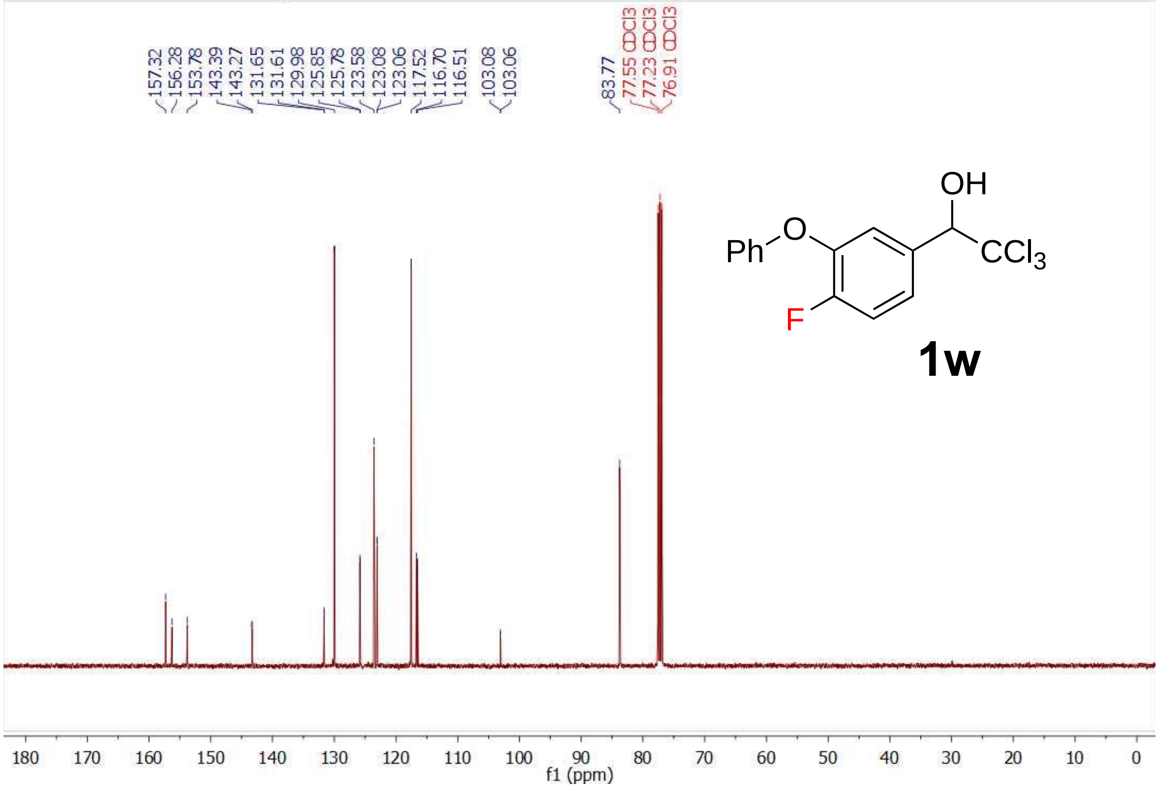

Zhu-3-114.3.fid — F19CPD CDCl3 C:\\ nmr 5

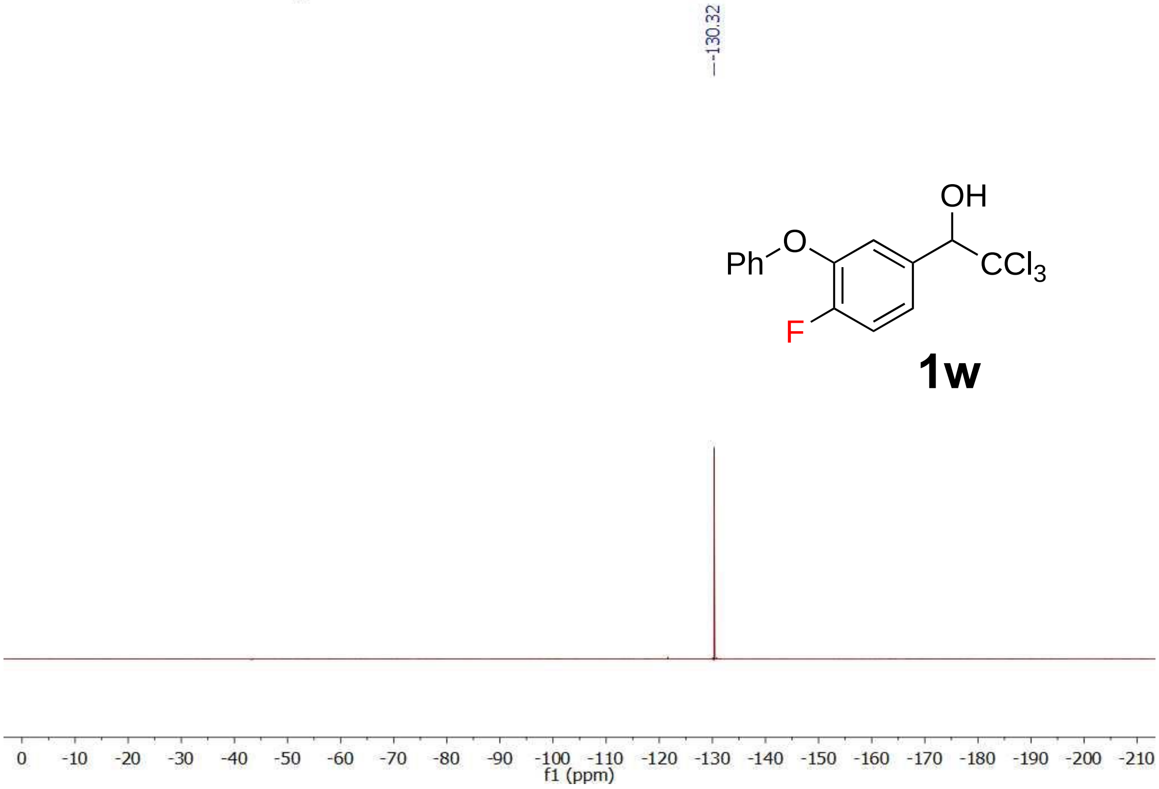

Zhu-3-142.1.fid — CMC_PROTON CDCl3 C:\\ nmr 22

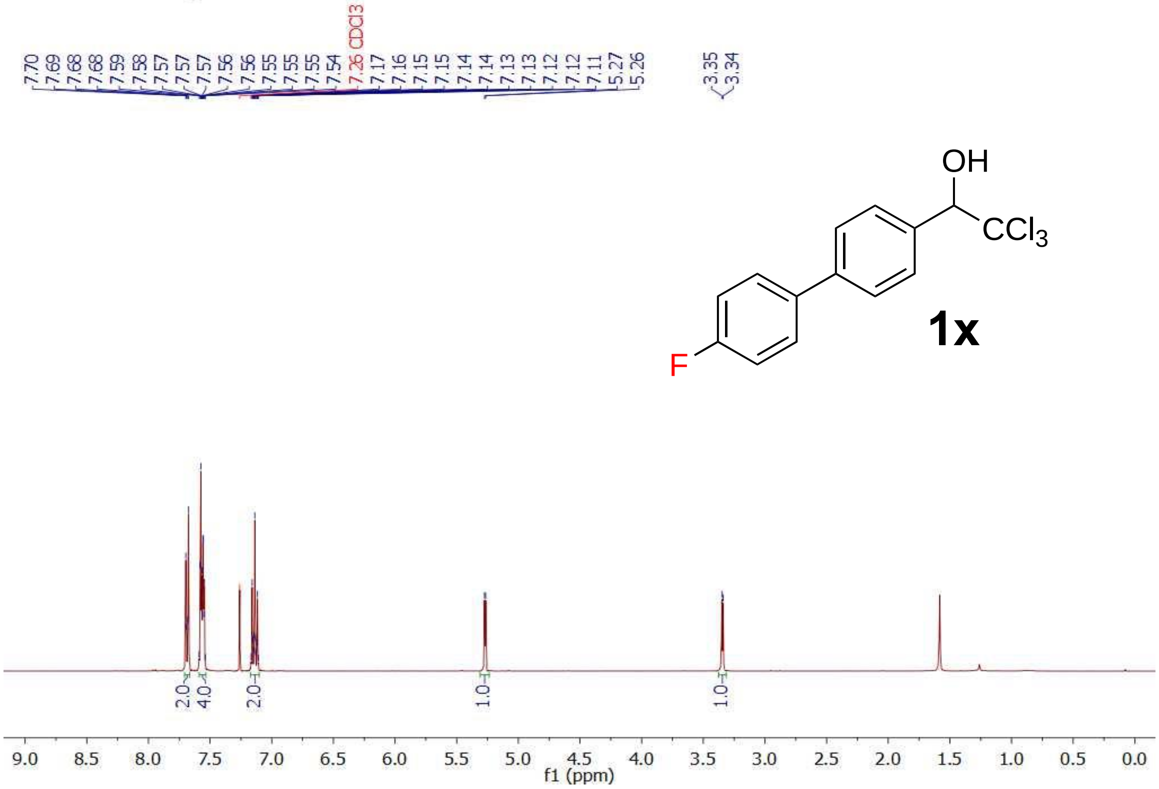

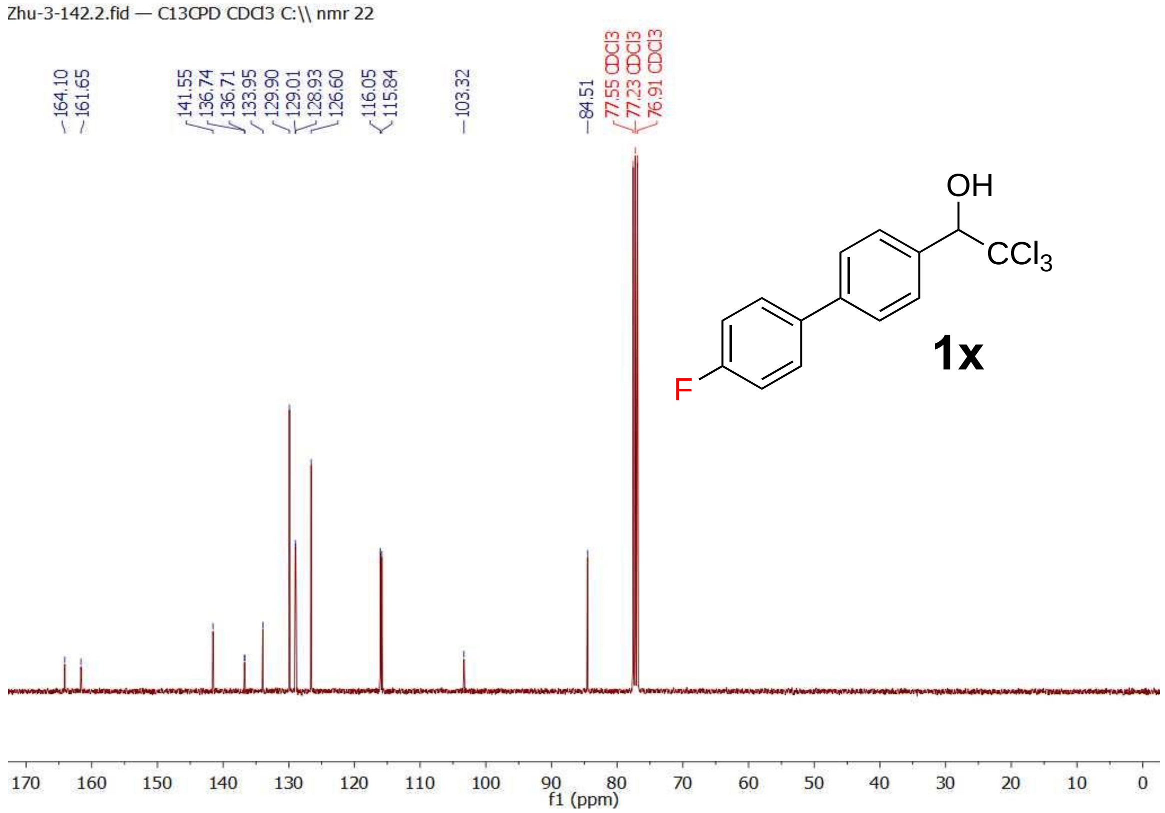
Zhu-3-142.2.fid — C13CPD CDCl3 C:\\ nmr 22
164.10
161.65
141.55
136.74
136.71
133.95
129.90
129.01
128.93
126.60
116.05
115.84
103.32
84.51
77.55 CDCl3
77.23 CDCl3
76.91 CDCl3
OH
CCl3
F
1x
170 160 150 140 130 120 110 100 90 80 70 60 50 40 30 20 10 0
f1 (ppm)

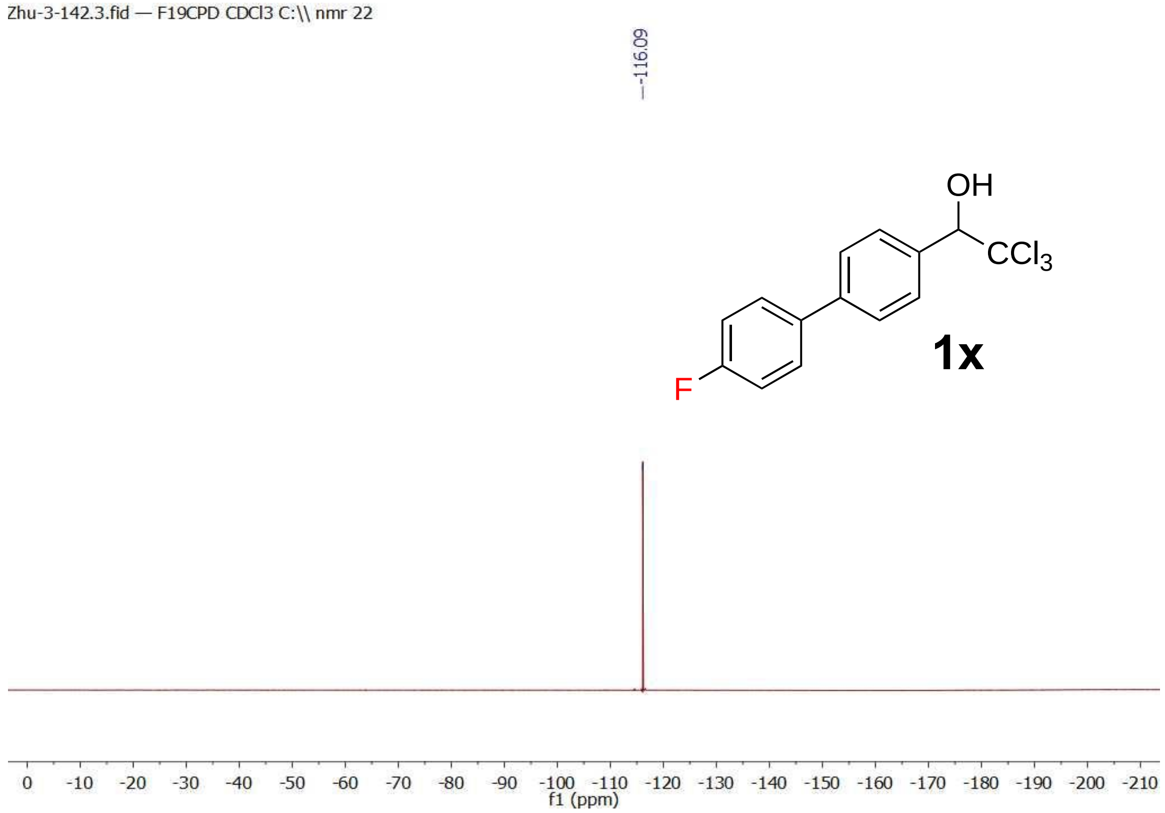
Zhu-3-142.3.fid — F19CPD CDCl3 C:\\ nmr 22
-116.09
OH
CCl3
F
1x
0 -10 -20 -30 -40 -50 -60 -70 -80 -90 -100 -110 -120 -130 -140 -150 -160 -170 -180 -190 -200 -210
f1 (ppm)

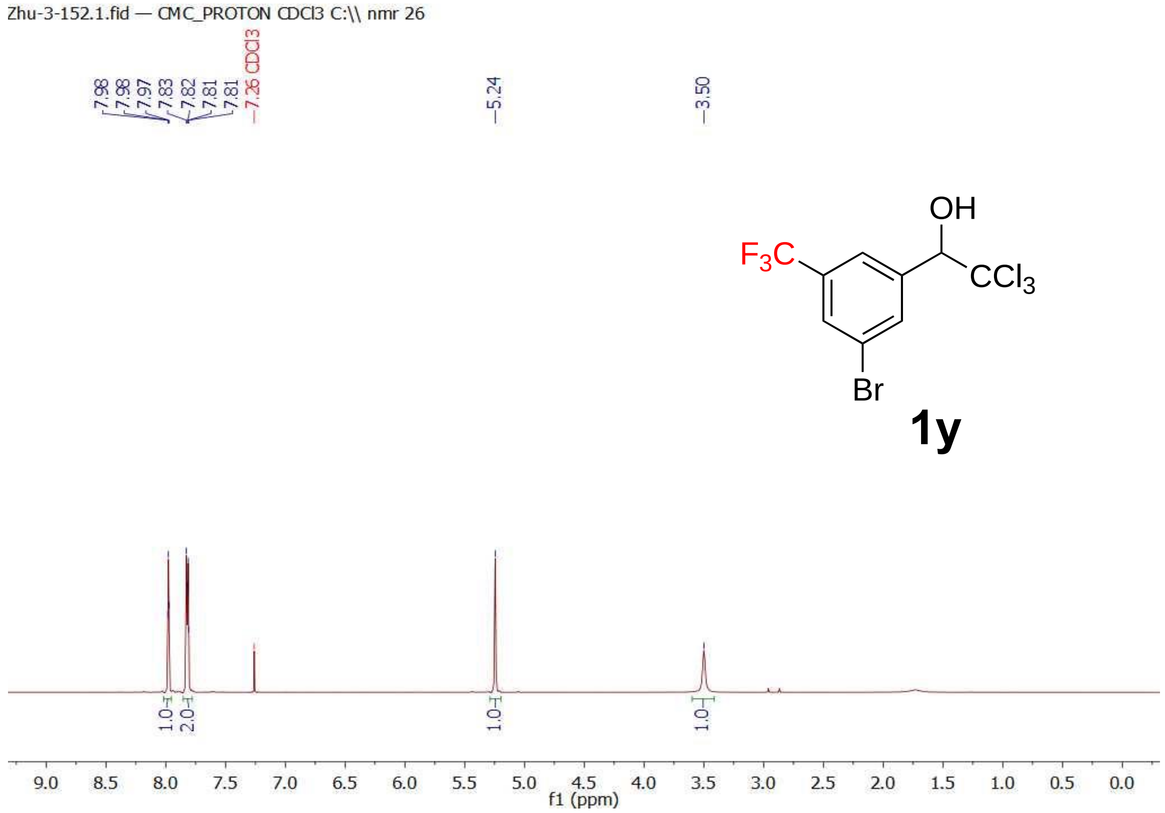
Zhu-3-152.1.fid — CMC_PROTON CDCl3 C:\\ nmr 26
7.98
7.98
7.97
7.83
7.82
7.81
7.81
7.26 CDCl3
5.24
3.50
OH
F3C
CCl3
Br
1.0
2.0
1.0
1.0
9.0 8.5 8.0 7.5 7.0 6.5 6.0 5.5 5.0 4.5 4.0 3.5 3.0 2.5 2.0 1.5 1.0 0.5 0.0
f1 (ppm)

1y

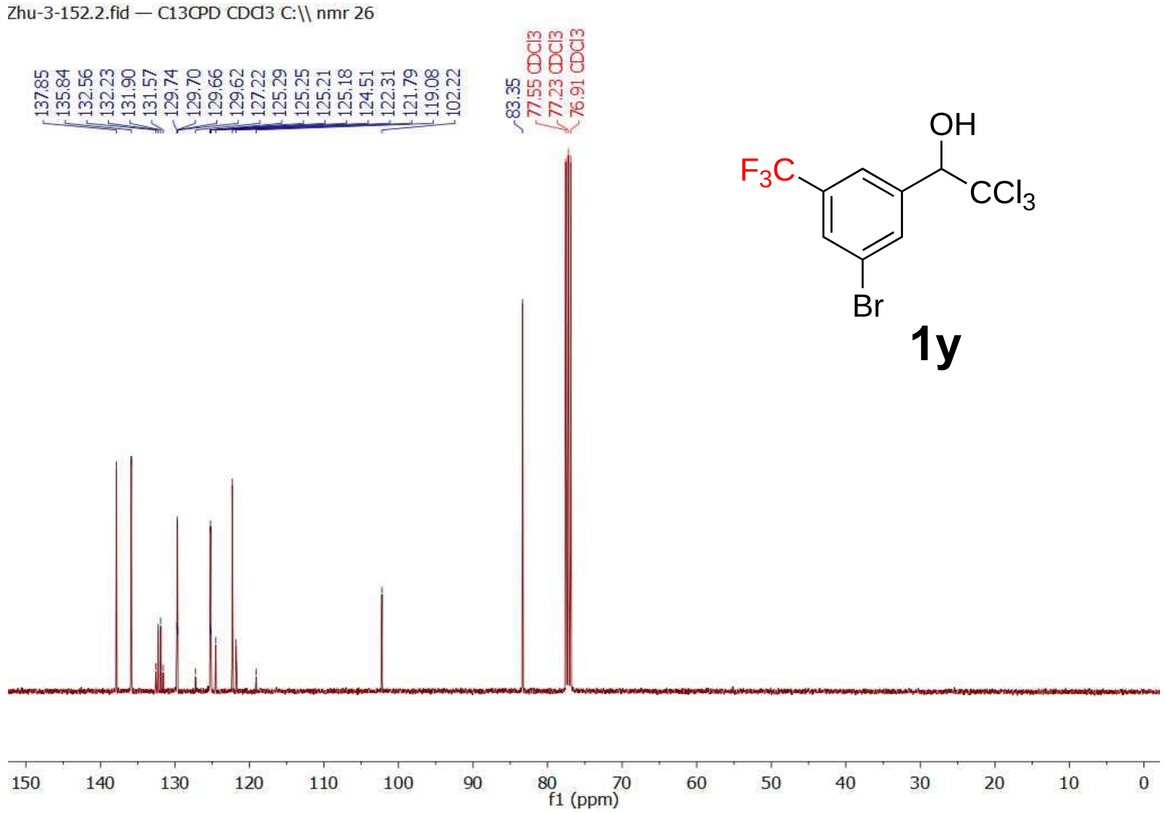
Zhu-3-152.2.fid — C13CPD CDCl3 C:\\ nmr 26
137.85
135.84
132.56
132.23
131.90
131.57
129.74
129.70
129.66
129.62
127.22
125.29
125.25
125.21
125.18
124.51
122.31
121.79
119.08
102.22
83.35
77.55 CDCl3
77.23 CDCl3
76.91 CDCl3
OH
F3C
CCl3
Br
150 140 130 120 110 100 90 80 70 60 50 40 30 20 10 0
f1 (ppm)

1y

Zhu-3-152.3.fid — F19CPD CDCl3 C:\\ nmr 26

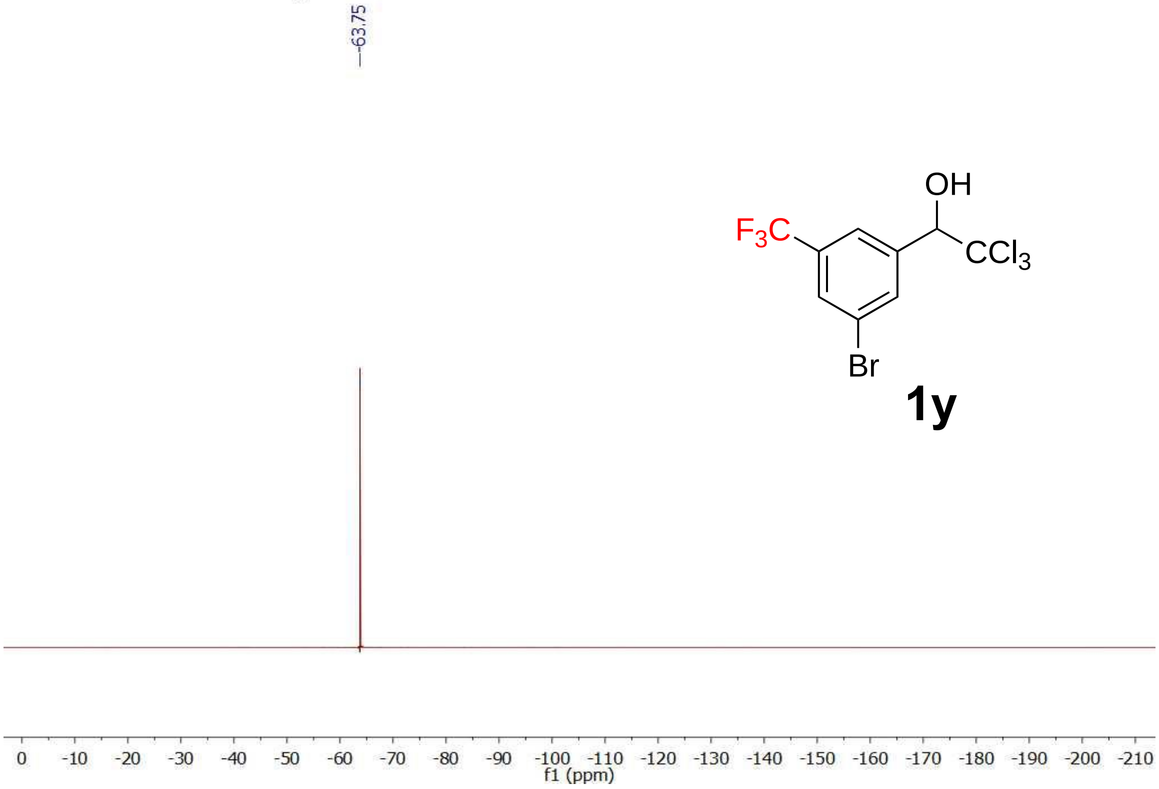

1y

Zhu-3-4.1.fid — CMC_PROTON CDCl3 C:\\ nmr 12

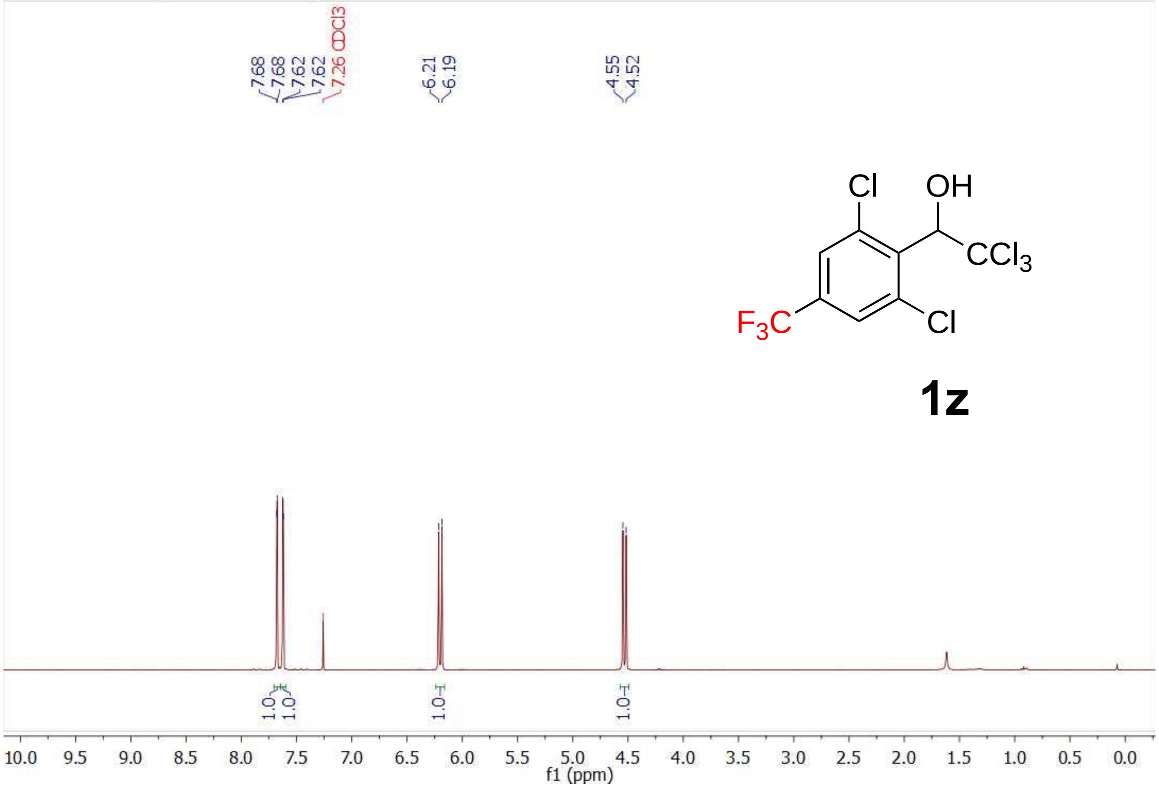

1z

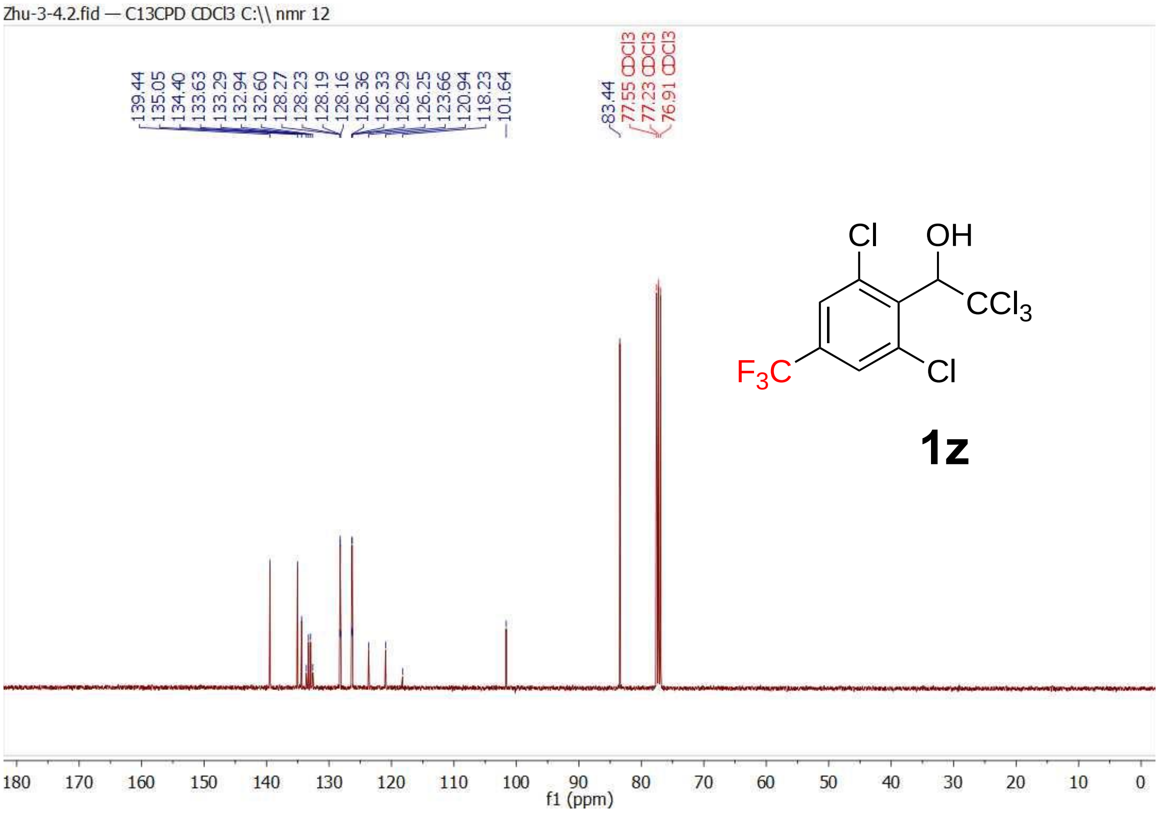
Zhu-3-4.2.fid — C13CPD CDCl3 C:\\ nmr 12
139.44
135.05
134.40
133.63
133.29
132.94
132.60
128.27
128.23
128.19
128.16
126.36
126.33
126.29
126.25
123.66
120.94
118.23
101.64
83.44
77.55 CDCl3
77.23 CDCl3
76.91 CDCl3
Cl
OH
CCl3
F3C
Cl
1z
180 170 160 150 140 130 120 110 100 90 80 70 60 50 40 30 20 10 0
f1 (ppm)

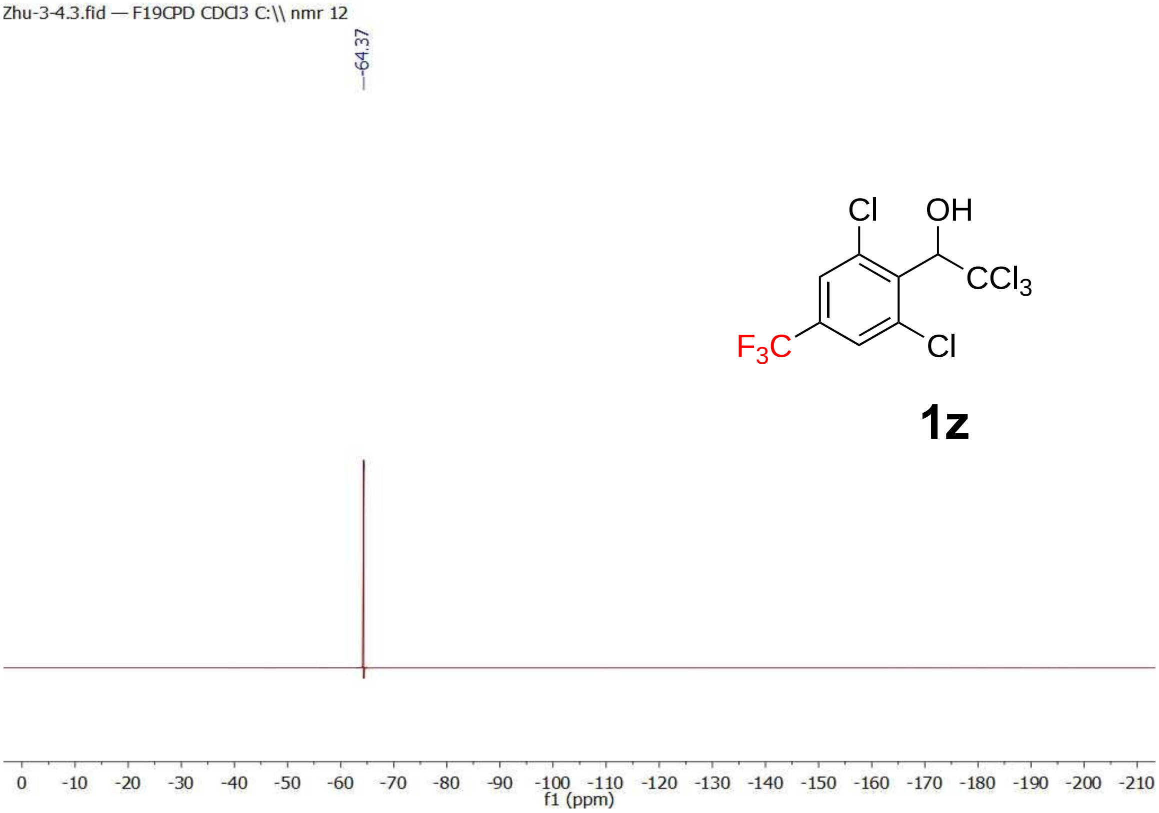
Zhu-3-4.3.fid — F19CPD CDCl3 C:\\ nmr 12
-64.37
Cl
OH
CCl3
F3C
Cl
1z
0 -10 -20 -30 -40 -50 -60 -70 -80 -90 -100 -110 -120 -130 -140 -150 -160 -170 -180 -190 -200 -210
f1 (ppm)

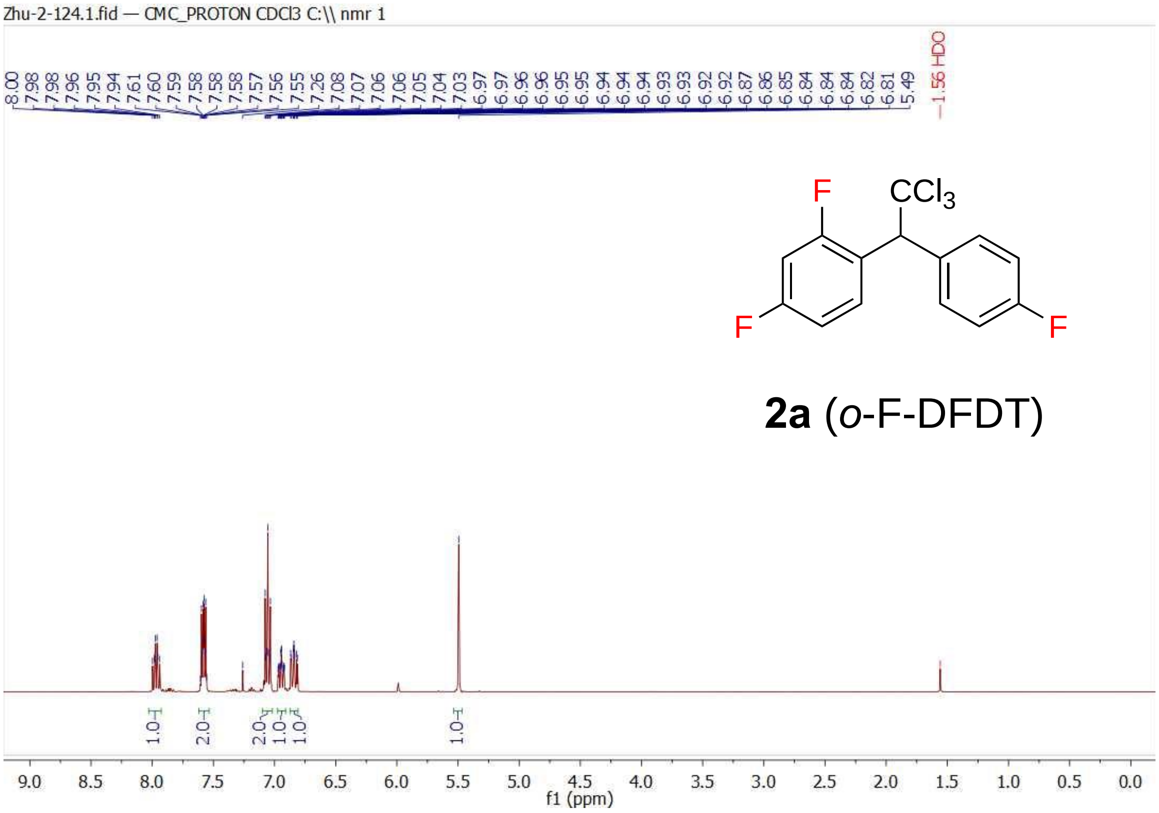
Zhu-2-124.1.fid — CMC_PROTON CDCl3 C:\\ nmr 1
8.00
7.98
7.98
7.96
7.95
7.94
7.61
7.60
7.59
7.58
7.58
7.58
7.57
7.56
7.55
7.26
7.08
7.07
7.06
7.06
7.05
7.04
7.03
6.97
6.97
6.96
6.96
6.95
6.95
6.94
6.94
6.94
6.93
6.93
6.92
6.92
6.87
6.86
6.85
6.84
6.84
6.84
6.82
6.81
5.49
1.56 HDO
F
CCl3
F
F
2a (o-F-DFDT)
1.0
2.0
2.0
1.0
1.0
1.0
9.0
8.5
8.0
7.5
7.0
6.5
6.0
5.5
5.0
4.5
4.0
3.5
3.0
2.5
2.0
1.5
1.0
0.5
0.0
f1 (ppm)

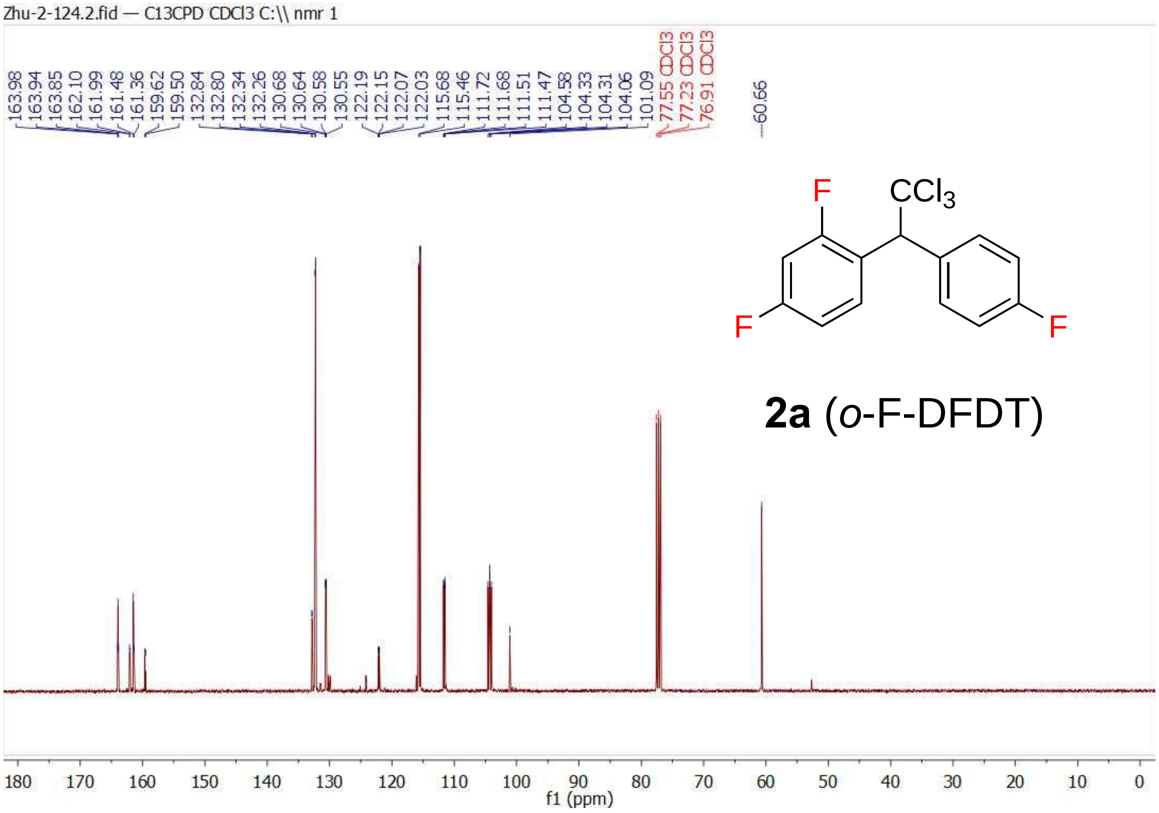
Zhu-2-124.2.fid — C13CPD CDCl3 C:\\ nmr 1
163.98
163.94
163.85
162.10
161.99
161.48
161.36
159.62
159.50
132.84
132.80
132.34
132.26
130.68
130.64
130.58
130.55
122.19
122.15
122.07
122.03
115.68
115.46
111.72
111.68
111.51
111.47
104.58
104.33
104.31
104.06
101.09
77.55 CDCl3
77.23 CDCl3
76.91 CDCl3
60.66
F
CCl3
F
F
2a (o-F-DFDT)
180
170
160
150
140
130
120
110
100
90
80
70
60
50
40
30
20
10
0
f1 (ppm)

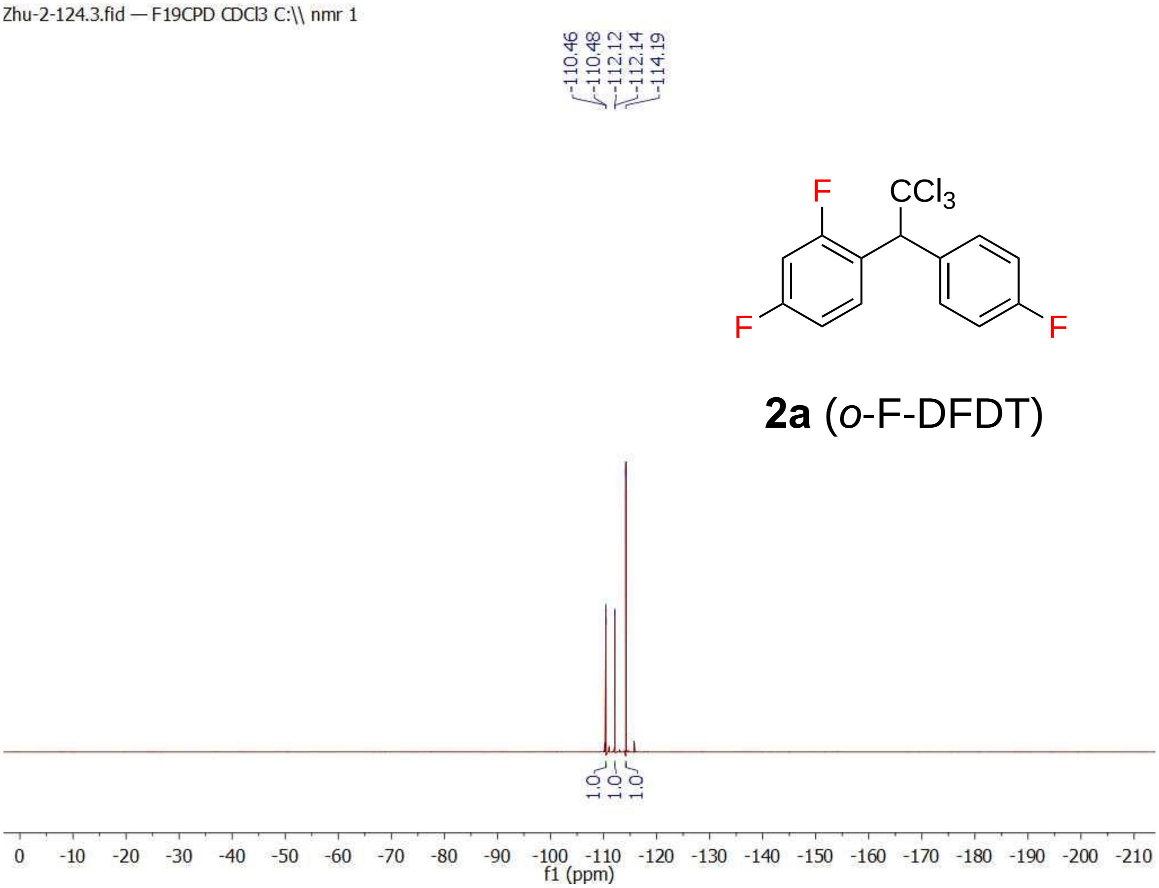
Zhu-2-124.3.fid — F19CPD CDCl3 C:\\ nmr 1
-110.46
-110.48
-112.12
-112.14
-114.19
F
CCl3
F
F
2a (o-F-DFDT)
1.0
1.0
1.0
0 -10 -20 -30 -40 -50 -60 -70 -80 -90 -100 -110 -120 -130 -140 -150 -160 -170 -180 -190 -200 -210
f1 (ppm)

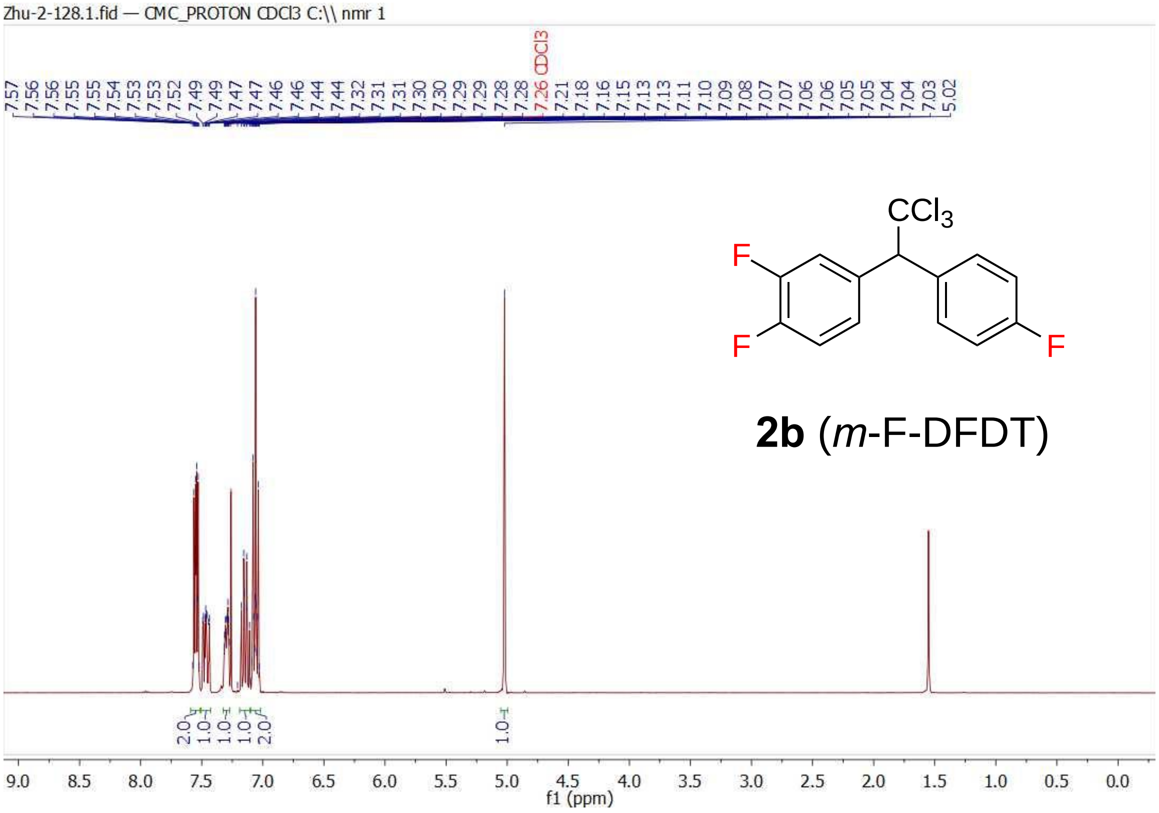
Zhu-2-128.1.fid — CMC_PROTON CDCl3 C:\\ nmr 1
7.57 7.56 7.56 7.55 7.55 7.54 7.53 7.53 7.52 7.49 7.49 7.47 7.47 7.46 7.46 7.44 7.44 7.32 7.31 7.30 7.30 7.29 7.29 7.28 7.26 CDCl3 7.21 7.18 7.16 7.15 7.13 7.13 7.11 7.10 7.09 7.08 7.07 7.07 7.06 7.06 7.05 7.05 7.04 7.04 7.03 5.02
CCl3
F
F
F
2b (m-F-DFDT)
2.0
1.0
1.0
1.0
2.0
1.0
9.0 8.5 8.0 7.5 7.0 6.5 6.0 5.5 5.0 4.5 4.0 3.5 3.0 2.5 2.0 1.5 1.0 0.5 0.0
f1 (ppm)

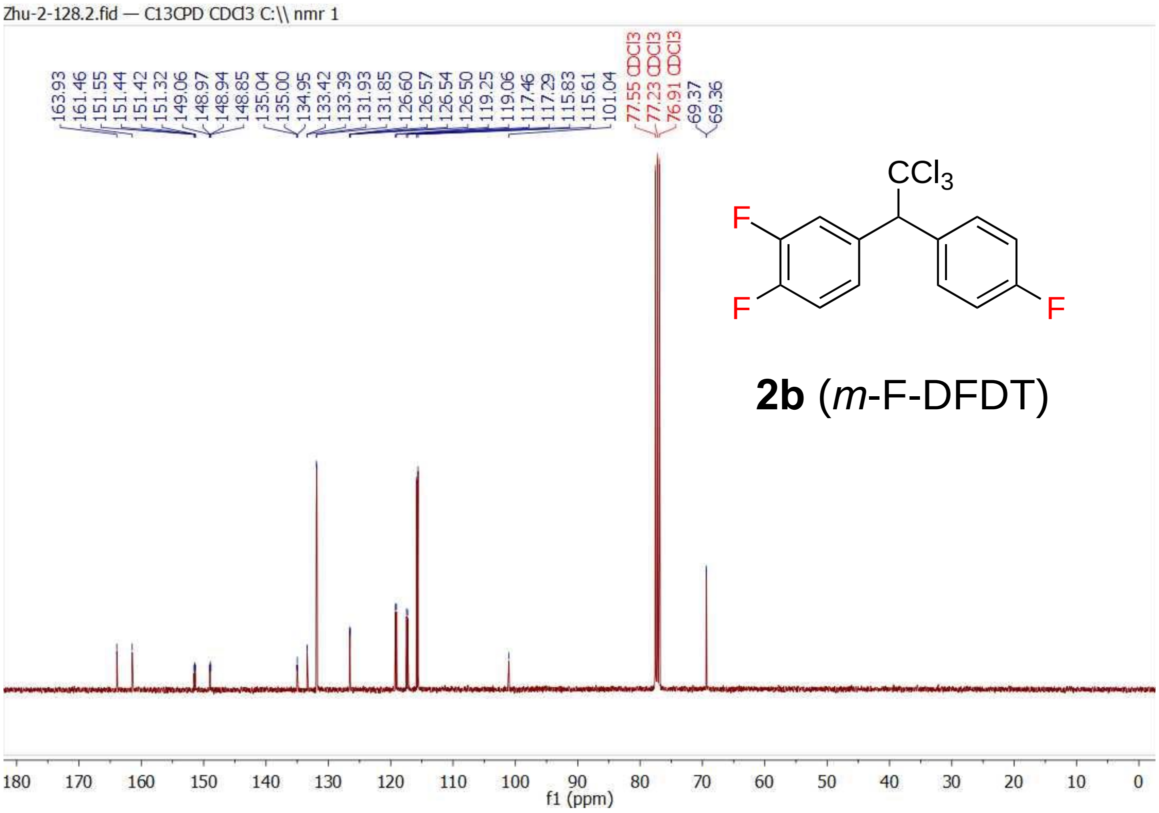
Zhu-2-128.2.fid — C13CPD CDCl3 C:\\ nmr 1
163.93
161.46
151.55
151.44
151.42
151.32
149.06
148.97
148.94
148.85
135.04
135.00
134.95
133.42
133.39
131.93
131.85
126.60
126.57
126.54
126.50
119.25
119.06
117.46
117.29
115.83
115.61
101.04
77.55 CDCl3
77.23 CDCl3
76.91 CDCl3
69.37
69.36
CCl3
F
F
F
2b (m-F-DFDT)
f1 (ppm)

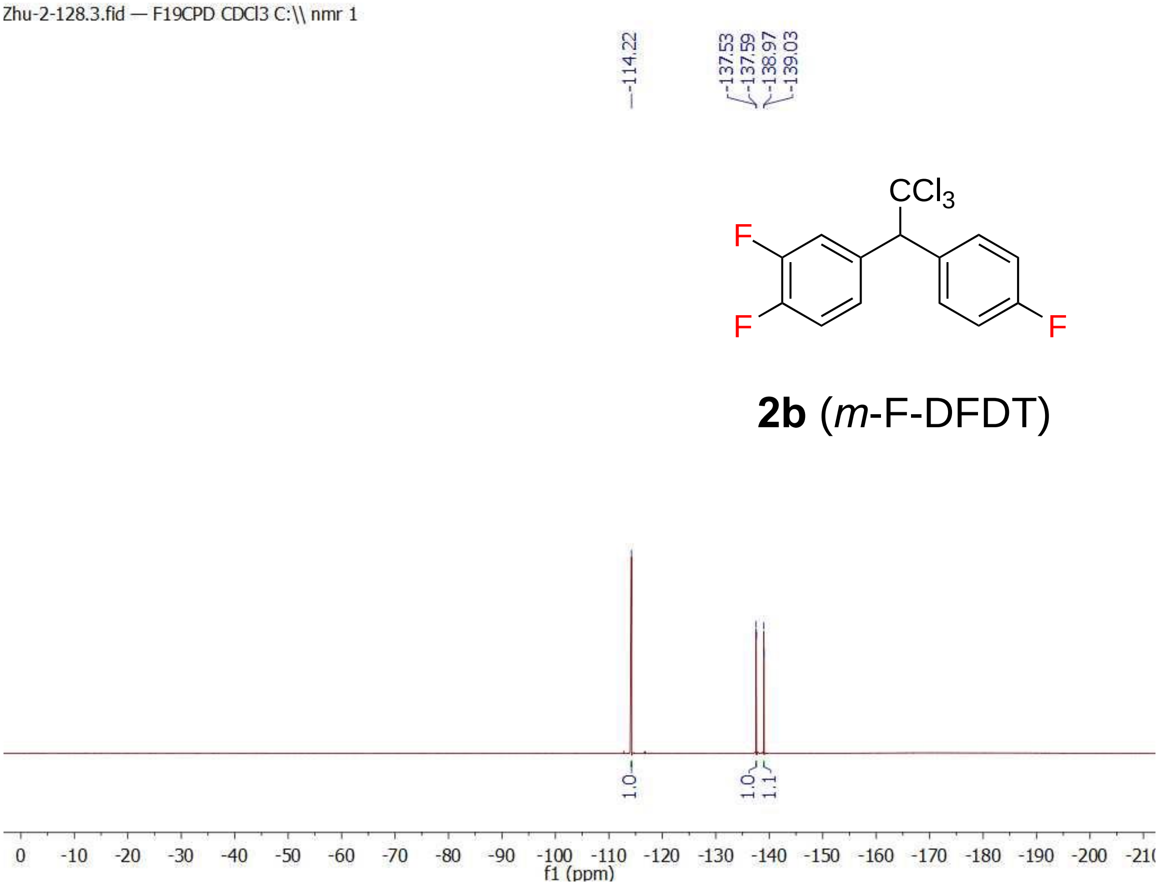
Zhu-2-128.3.fid — F19CPD CDCl3 C:\\ nmr 1
-114.22
-137.53
-137.59
-138.97
-139.03
CCl3
F
F
F
2b (m-F-DFDT)
1.0
1.0
1.1
f1 (ppm)

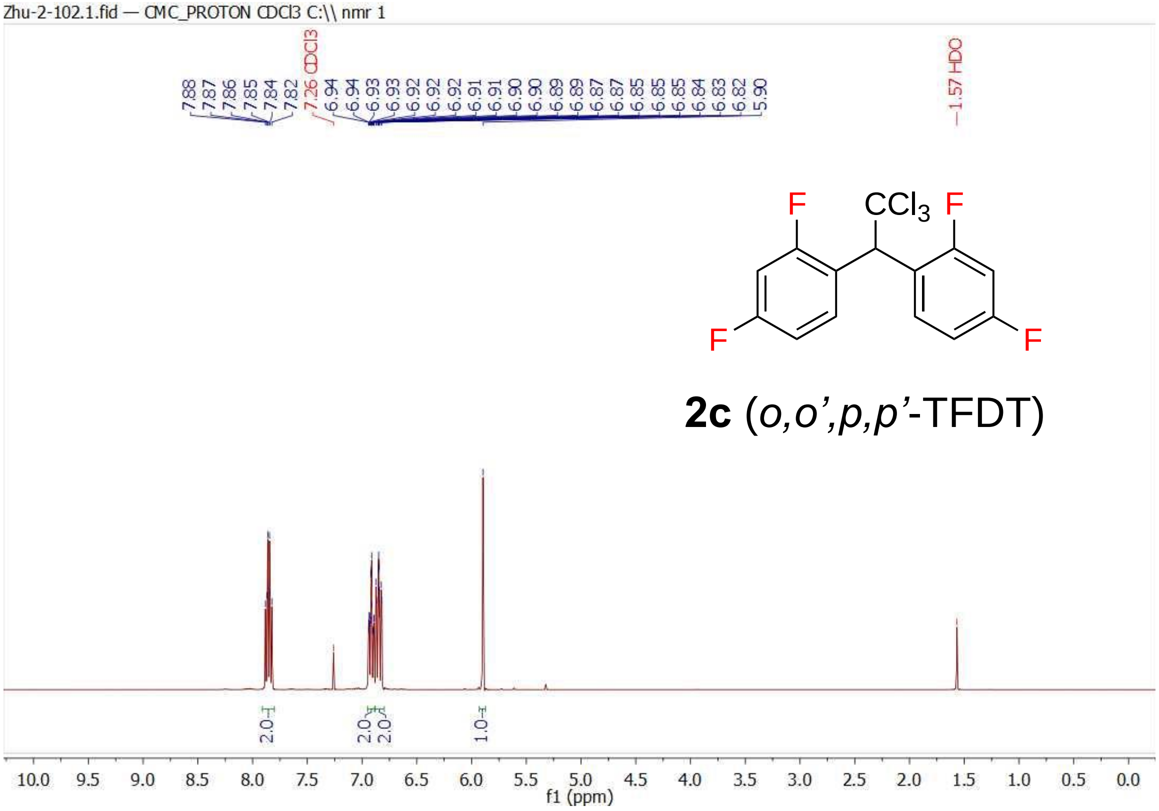
Zhu-2-102.1.fid — CMC_PROTON CDCl3 C:\\ nmr 1
7.88
7.87
7.86
7.85
7.84
7.82
7.26 CDCl3
6.94
6.94
6.93
6.93
6.92
6.92
6.92
6.91
6.91
6.90
6.90
6.89
6.89
6.87
6.85
6.85
6.85
6.84
6.83
6.82
5.90
1.57 HDO
F
CCl3
F
F
F
2c (o,o',p,p'-TFDT)
2.0
2.0
2.0
1.0
f1 (ppm)

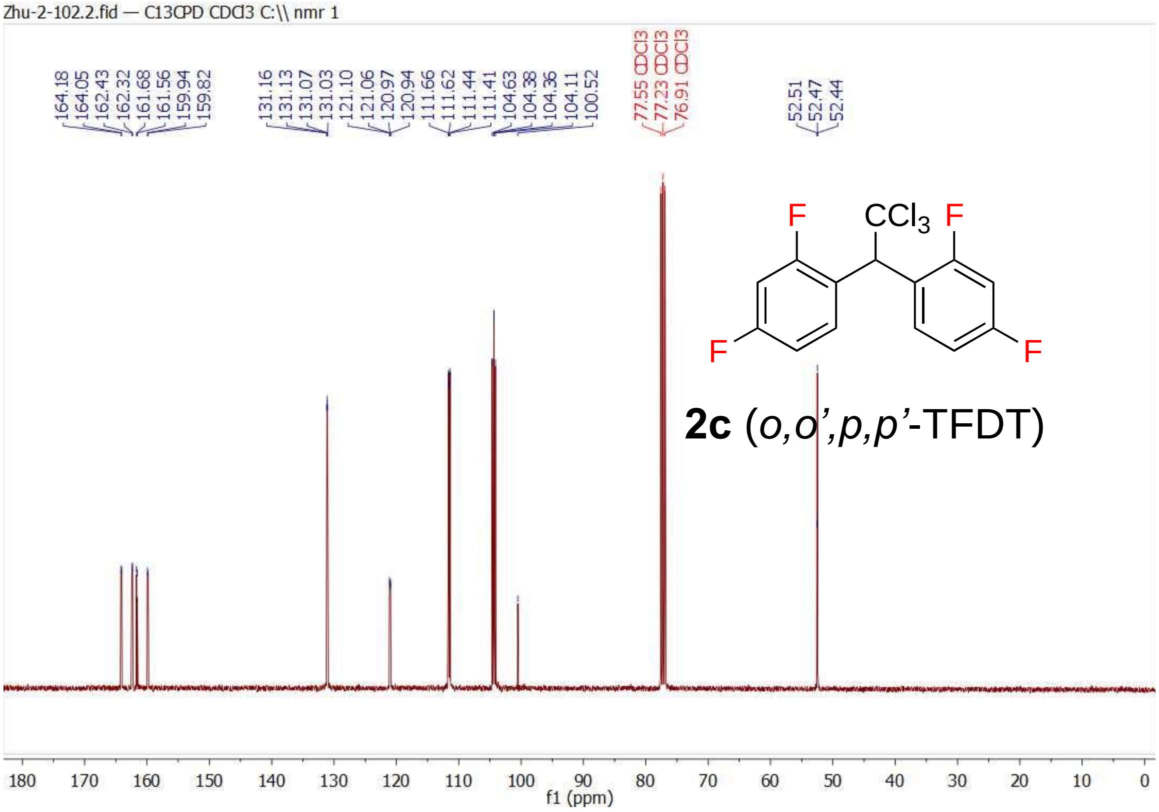
Zhu-2-102.2.fid — C13CPD CDCl3 C:\\ nmr 1
164.18
164.05
162.43
162.32
161.68
161.56
159.94
159.82
131.16
131.13
131.07
131.03
121.10
121.06
120.97
120.94
111.66
111.62
111.44
111.41
104.63
104.38
104.36
104.11
100.52
77.55 CDCl3
77.23 CDCl3
76.91 CDCl3
52.51
52.47
52.44
F
CCl3
F
F
F
2c (o,o',p,p'-TFDT)
f1 (ppm)

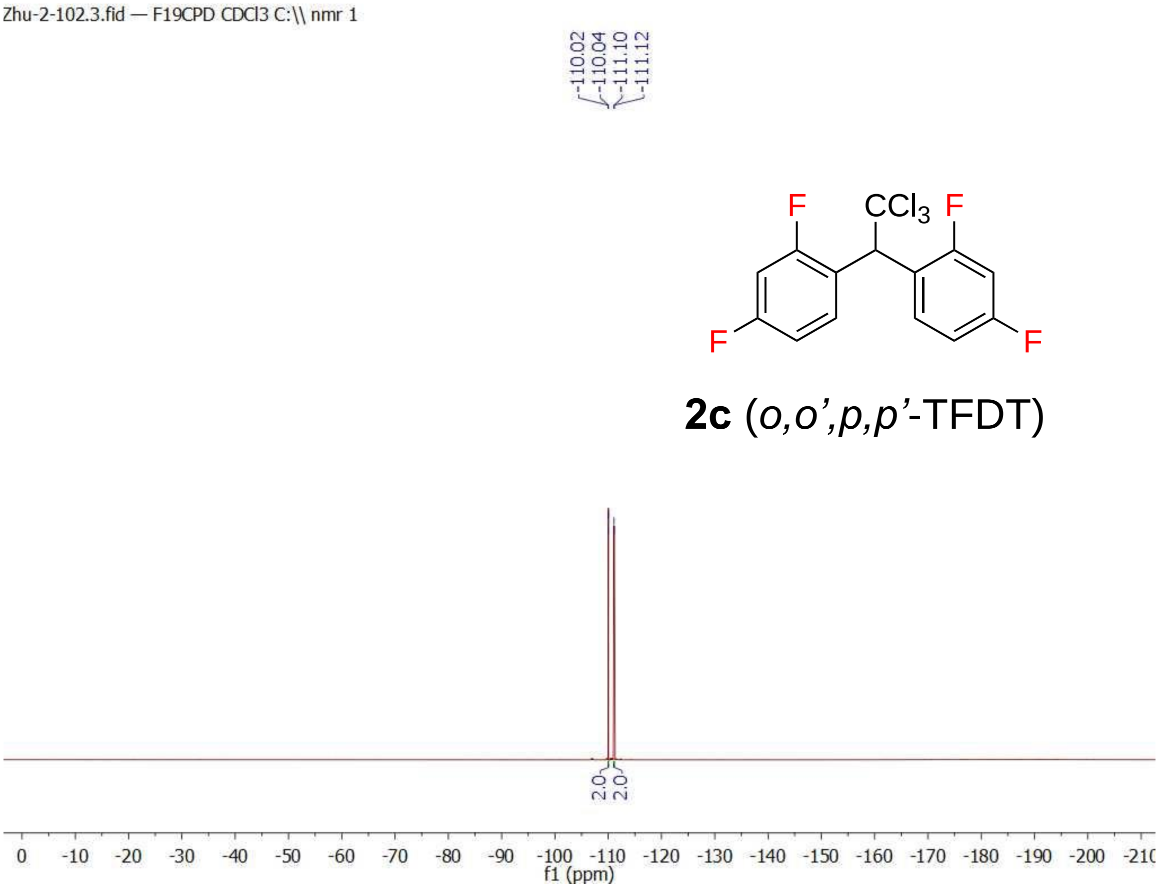

2c (*o,o',p,p'*-TFDT)

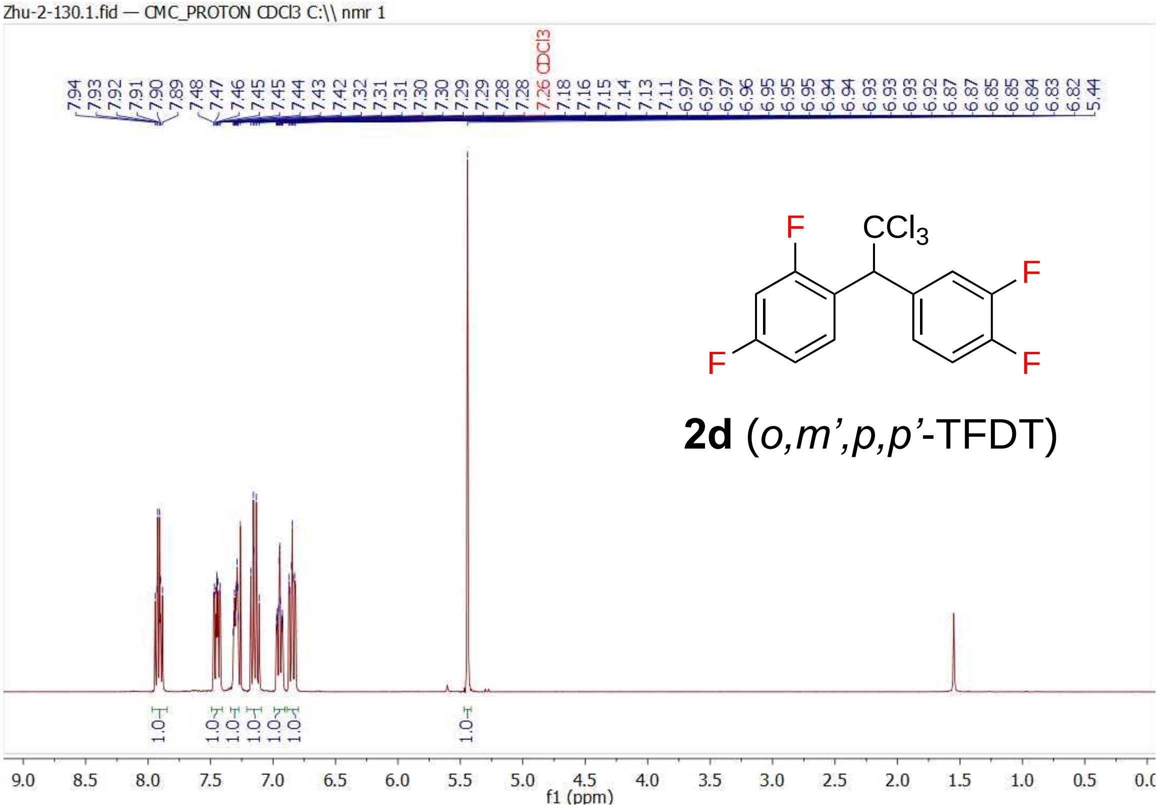

2d (*o,m',p,p'*-TFDT)

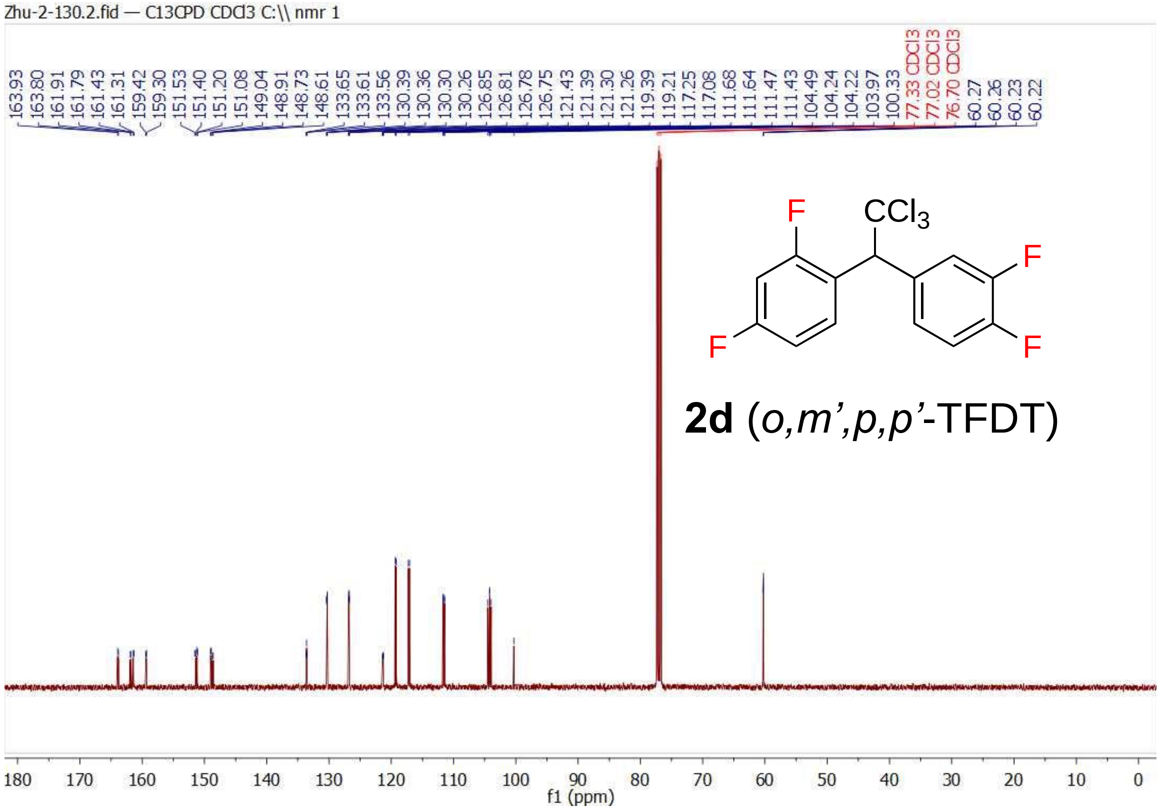
Zhu-2-130.2.fid — C13CPD CDCl3 C:\\ nmr 1
163.93 163.80 161.91 161.79 161.43 161.31 159.42 159.30 151.53 151.40 151.20 151.08 149.04 148.91 148.73 148.61 133.65 133.61 133.56 130.39 130.36 130.30 130.26 126.85 126.81 126.78 126.75 121.43 121.39 121.30 121.26 119.39 119.21 117.25 117.08 111.68 111.64 111.47 111.43 104.49 104.24 104.22 103.97 100.33
77.33 CDCl3 77.02 CDCl3 76.70 CDCl3
60.27 60.26 60.23 60.22
F
CCl3
F
F
F
2d (o,m’,p,p’-TFDT)
f1 (ppm)

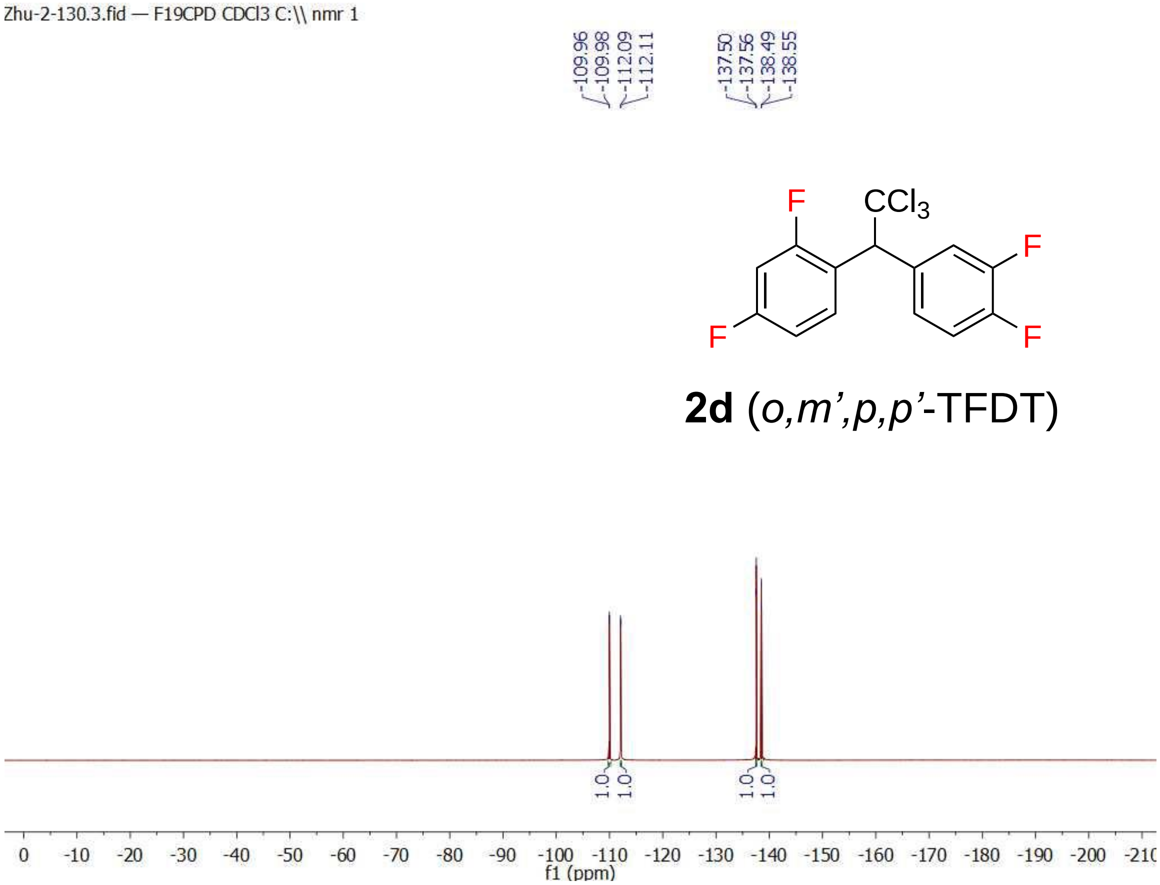
Zhu-2-130.3.fid — F19CPD CDCl3 C:\\ nmr 1
-109.96 -109.98 -112.09 -112.11
-137.50 -137.56 -138.49 -138.55
F
CCl3
F
F
F
2d (o,m’,p,p’-TFDT)
1.0 1.0 1.0 1.0
f1 (ppm)

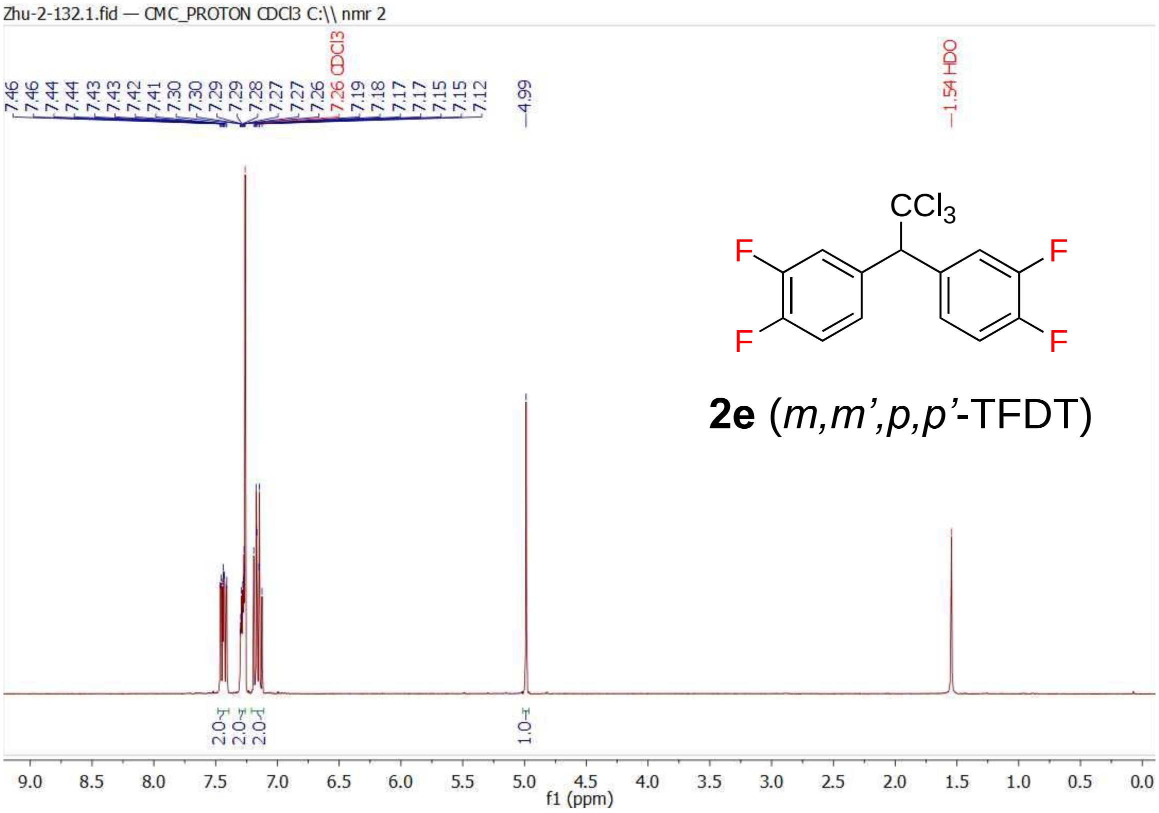

Zhu-2-132.1.fid — CMC_PROTON CDCl3 C:\\ nmr 2
7.46 7.46 7.44 7.44 7.43 7.43 7.42 7.41 7.30 7.30 7.29 7.29 7.28 7.27 7.27 7.26 7.26 CDCl3 7.19 7.18 7.17 7.17 7.15 7.15 7.12
4.99
1.54 HDO
CCl3
F
F
F
F
2e (m,m',p,p'-TFDT)
2.0 2.0 2.0
1.0
9.0 8.5 8.0 7.5 7.0 6.5 6.0 5.5 5.0 4.5 4.0 3.5 3.0 2.5 2.0 1.5 1.0 0.5 0.0
f1 (ppm)

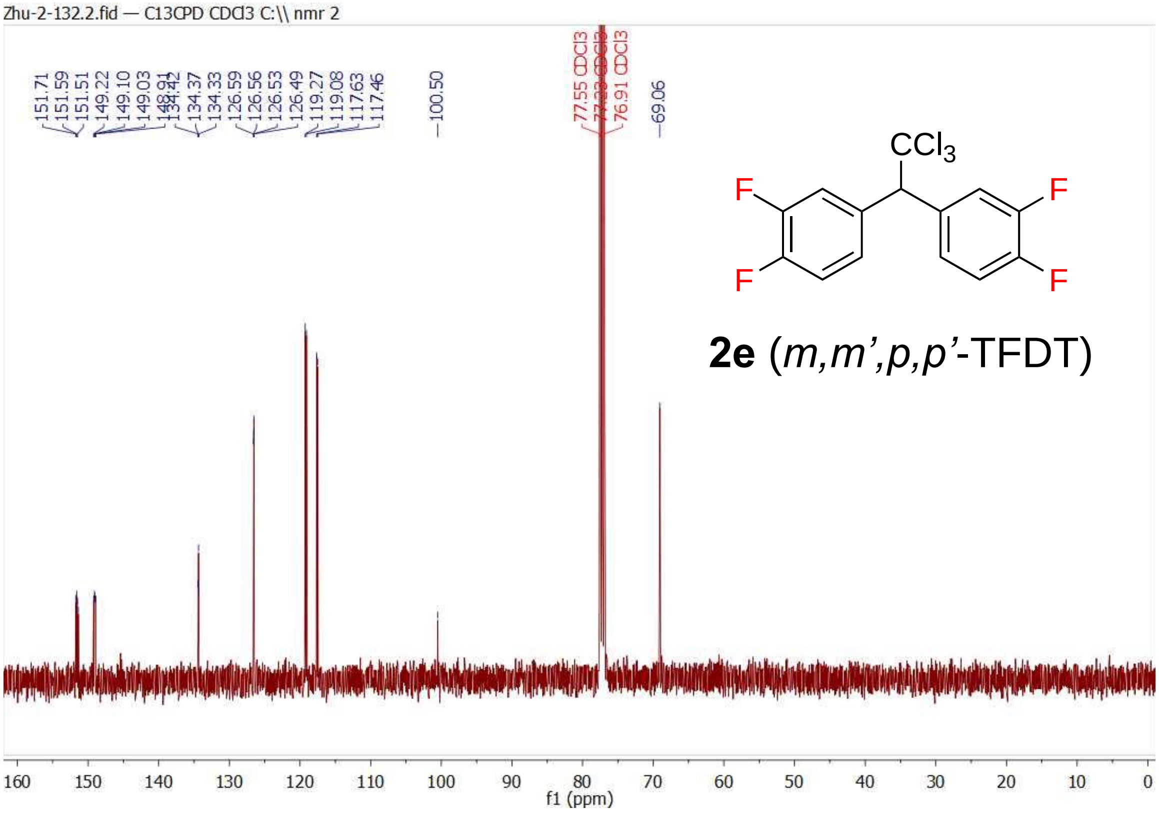

Zhu-2-132.2.fid — C13CPD CDCl3 C:\\ nmr 2
151.71 151.59 151.51 149.22 149.10 149.03 148.91 134.42 134.37 134.33 126.59 126.56 126.53 126.49 119.27 119.08 117.63 117.46
100.50
77.55 CDCl3 77.23 CDCl3 76.91 CDCl3
69.06
CCl3
F
F
F
F
2e (m,m',p,p'-TFDT)
160 150 140 130 120 110 100 90 80 70 60 50 40 30 20 10 0
f1 (ppm)

Zhu-2-132.3.fid — F19CPD CDCl3 C:\\ nmr 2

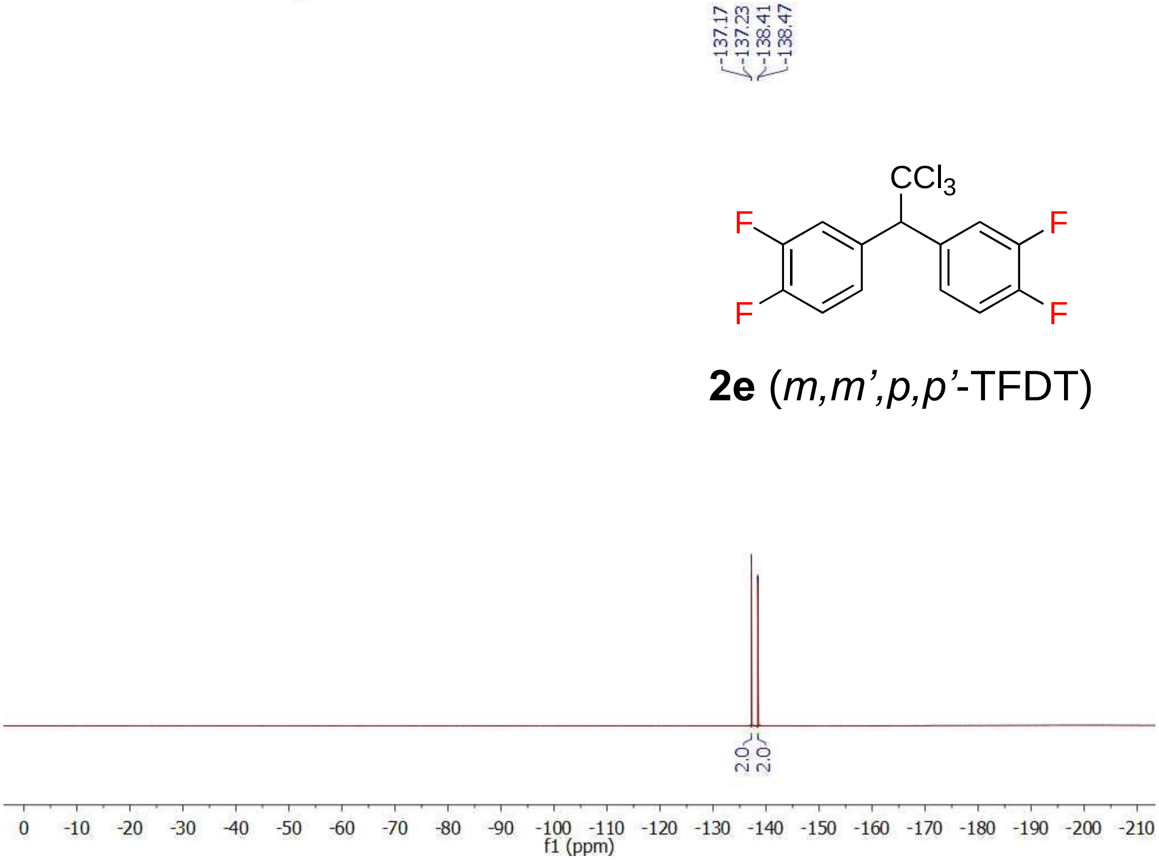

2e (*m,m',p,p'*-TFDT)

Zhu-3-184 tail.2.fid — C13CPD CDCl3 C:\\ nmr 10

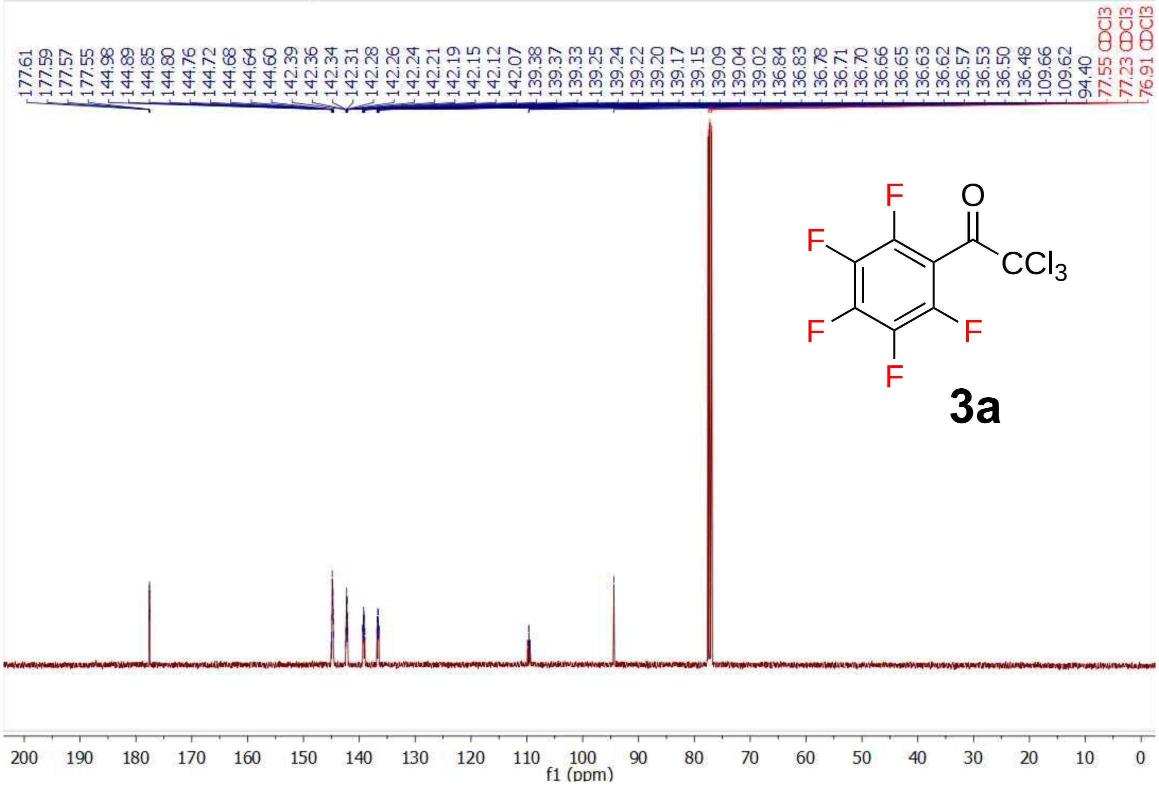

3a

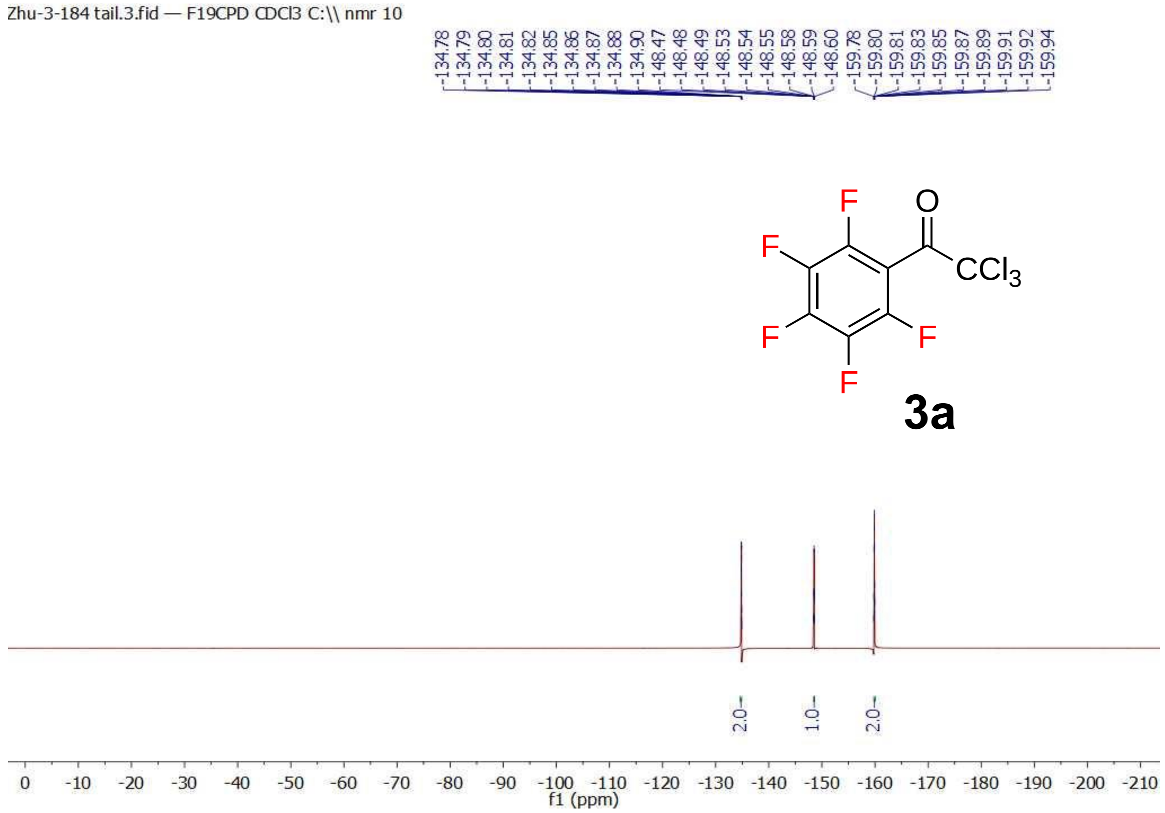
Zhu-3-184 tail.3.fid — F19CPD CDCl3 C:\\ nmr 10
-134.78
-134.79
-134.80
-134.81
-134.82
-134.85
-134.86
-134.87
-134.88
-134.90
-148.47
-148.48
-148.49
-148.53
-148.54
-148.55
-148.58
-148.59
-148.60
-159.78
-159.80
-159.81
-159.83
-159.85
-159.87
-159.89
-159.91
-159.92
-159.94
F
F
F
F
F
O
CCl3
3a
2.0
1.0
2.0
0 -10 -20 -30 -40 -50 -60 -70 -80 -90 -100 -110 -120 -130 -140 -150 -160 -170 -180 -190 -200 -210
f1 (ppm)

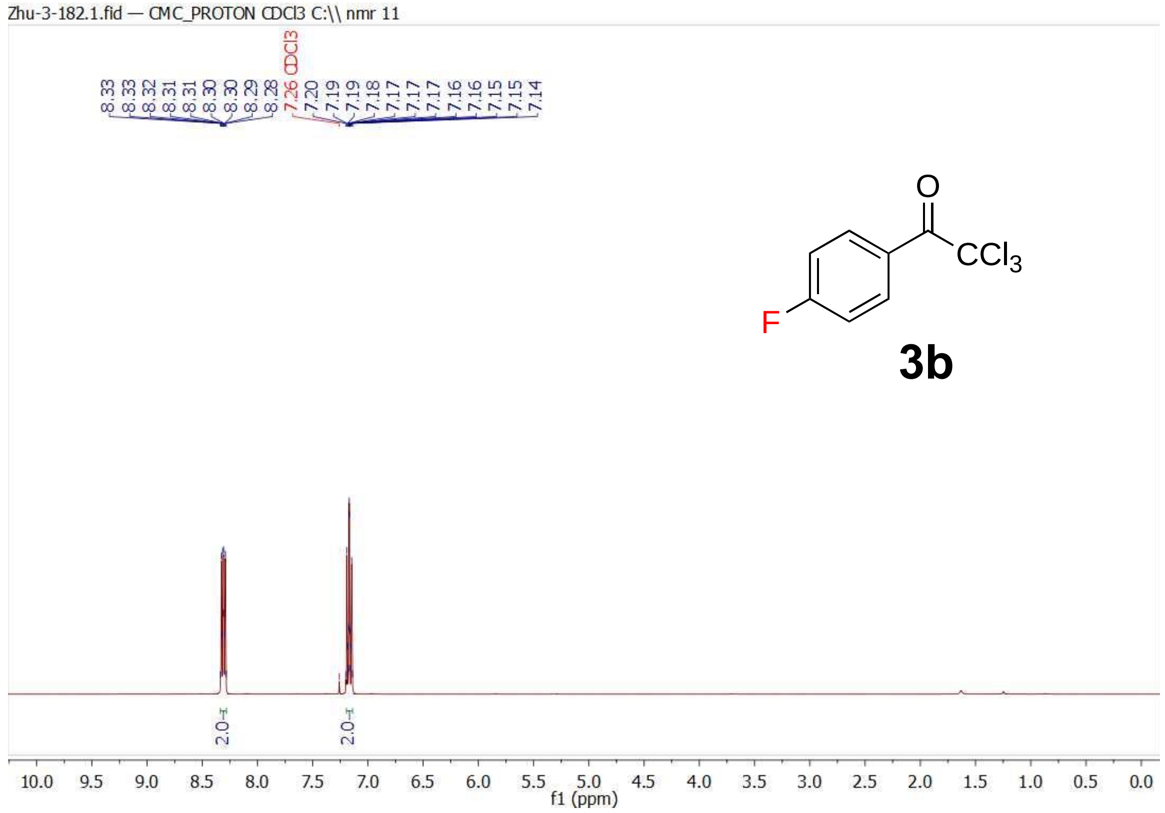
Zhu-3-182.1.fid — CMC_PROTON CDCl3 C:\\ nmr 11
8.33
8.33
8.32
8.31
8.31
8.30
8.30
8.29
8.28
7.26 CDCl3
7.20
7.19
7.19
7.18
7.17
7.17
7.17
7.16
7.16
7.15
7.15
7.14
O
CCl3
F
3b
2.0
2.0
10.0 9.5 9.0 8.5 8.0 7.5 7.0 6.5 6.0 5.5 5.0 4.5 4.0 3.5 3.0 2.5 2.0 1.5 1.0 0.5 0.0
f1 (ppm)

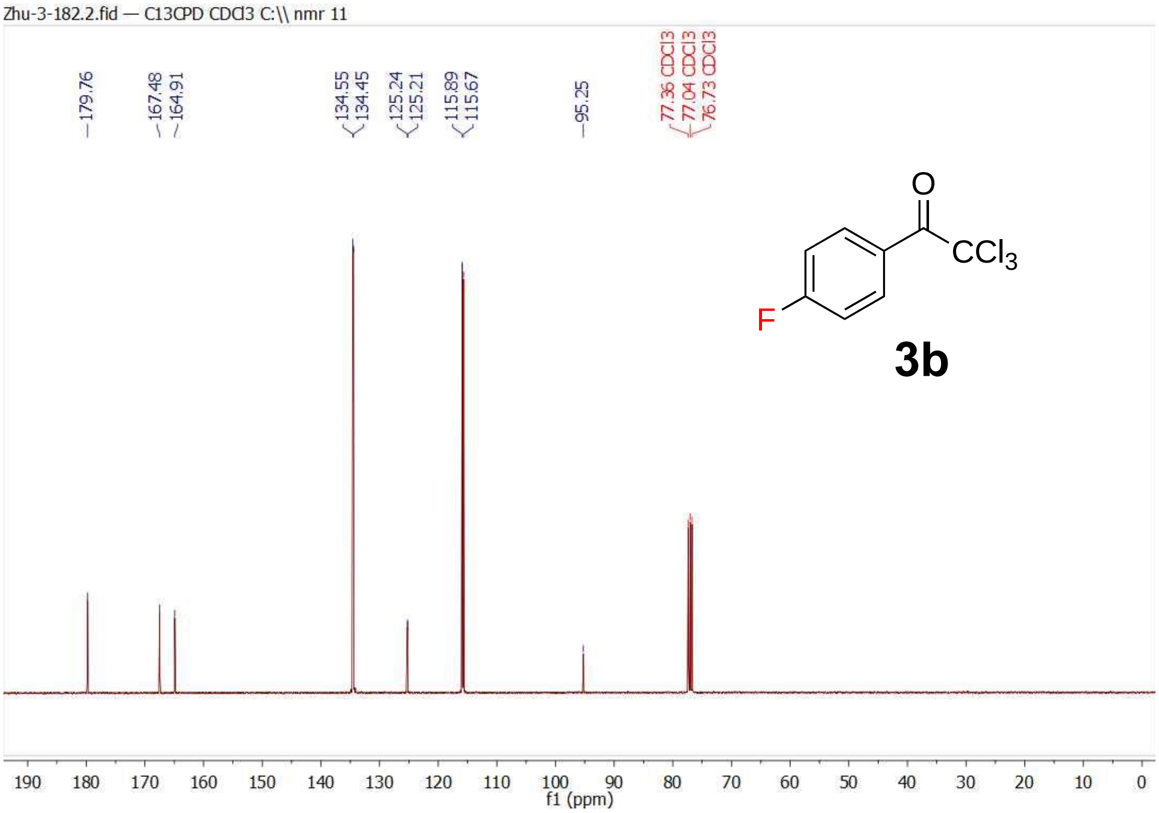
Zhu-3-182.2.fid — C13CPD CDCl3 C:\\ nmr 11
179.76
167.48
164.91
134.55
134.45
125.24
125.21
115.89
115.67
95.25
77.36 CDCl3
77.04 CDCl3
76.73 CDCl3
O
CCl3
F
3b
f1 (ppm)

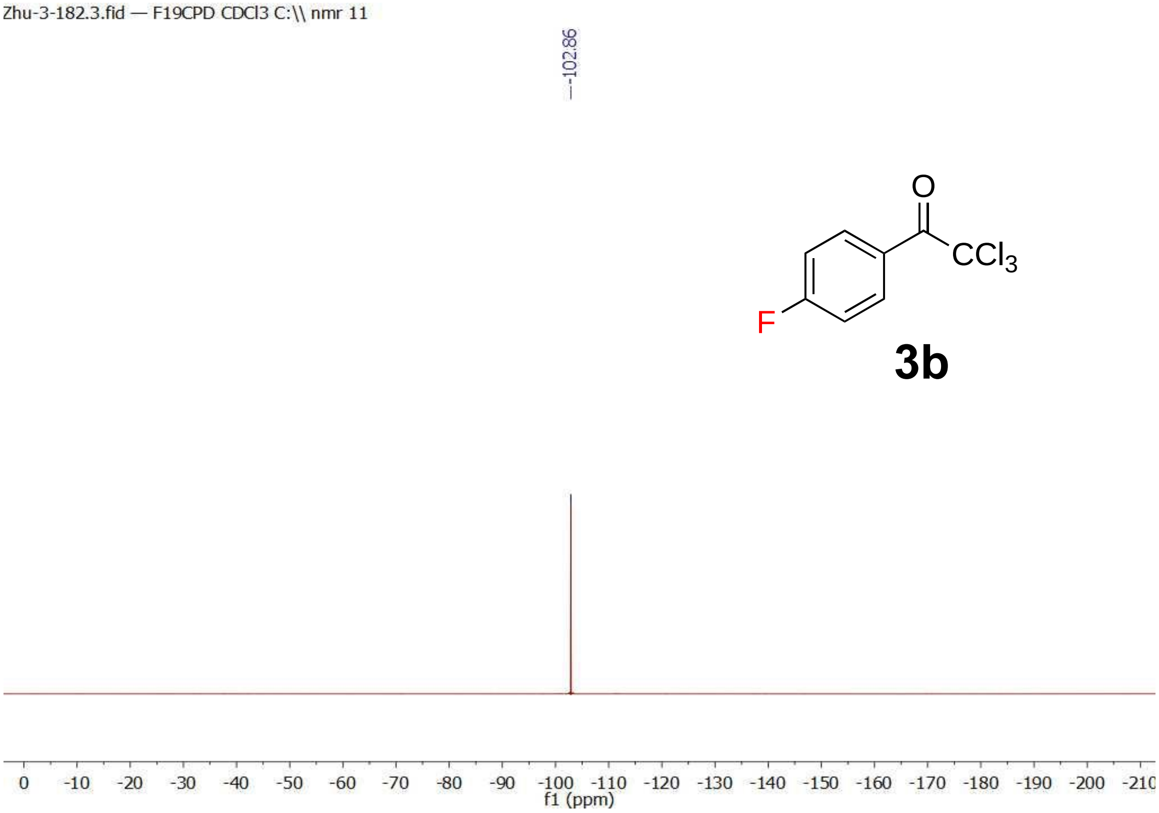
Zhu-3-182.3.fid — F19CPD CDCl3 C:\\ nmr 11
-102.86
O
CCl3
F
3b
f1 (ppm)

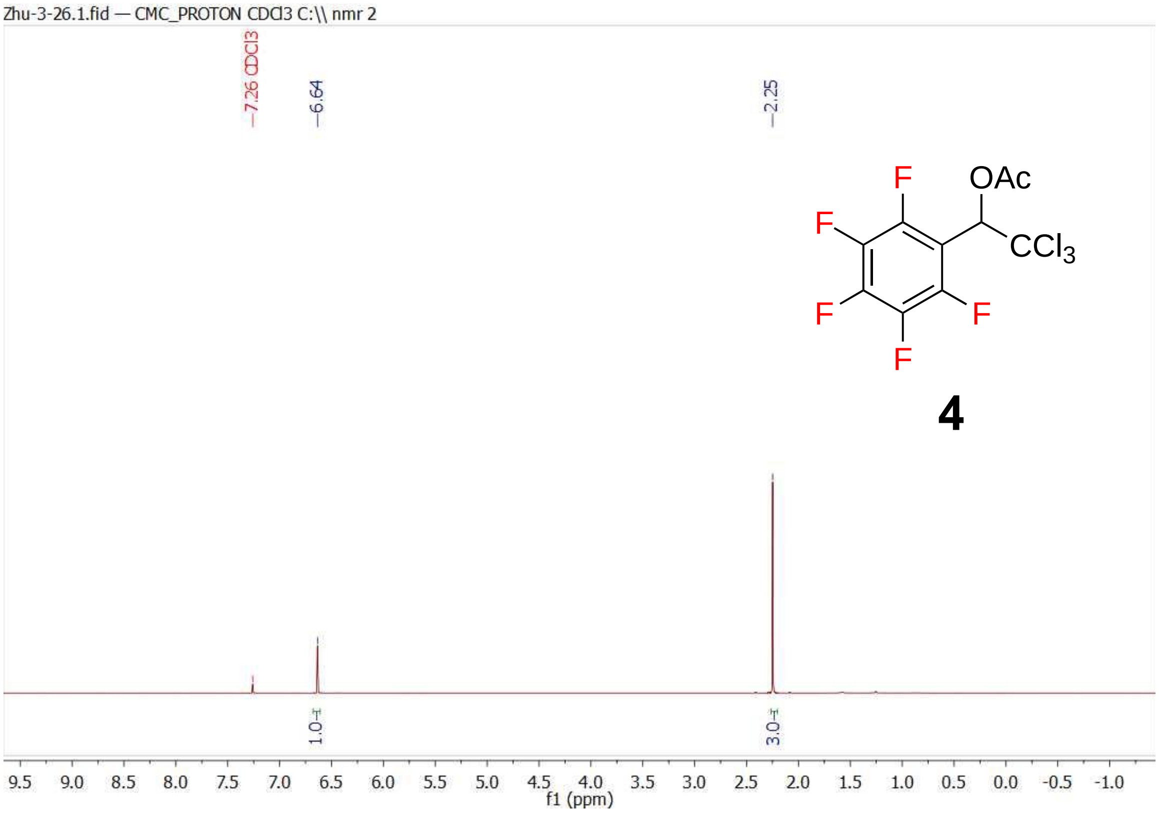
Zhu-3-26.1.fid — CMC_PROTON CDCl3 C:\\ nmr 2
7.26 CDCl3
6.64
2.25
F
OAc
CCl3
4
1.0
3.0
f1 (ppm)

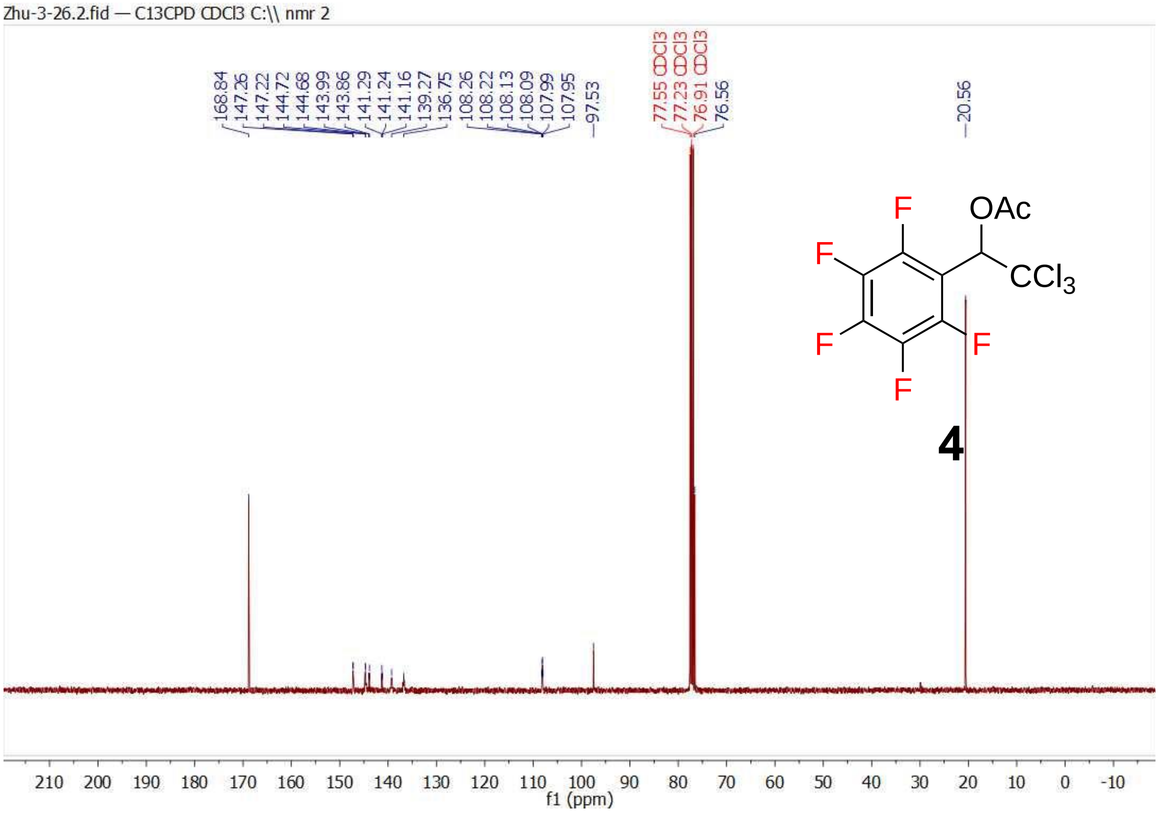
Zhu-3-26.2.fid — C13CPD CDCl3 C:\\ nmr 2
168.84
147.26
147.22
144.72
144.68
143.99
143.86
141.29
141.24
141.16
139.27
136.75
108.26
108.22
108.13
108.09
107.99
107.95
97.53
77.55 CDCl3
77.23 CDCl3
76.91 CDCl3
76.56
20.56
F
OAc
CCl3
4
f1 (ppm)

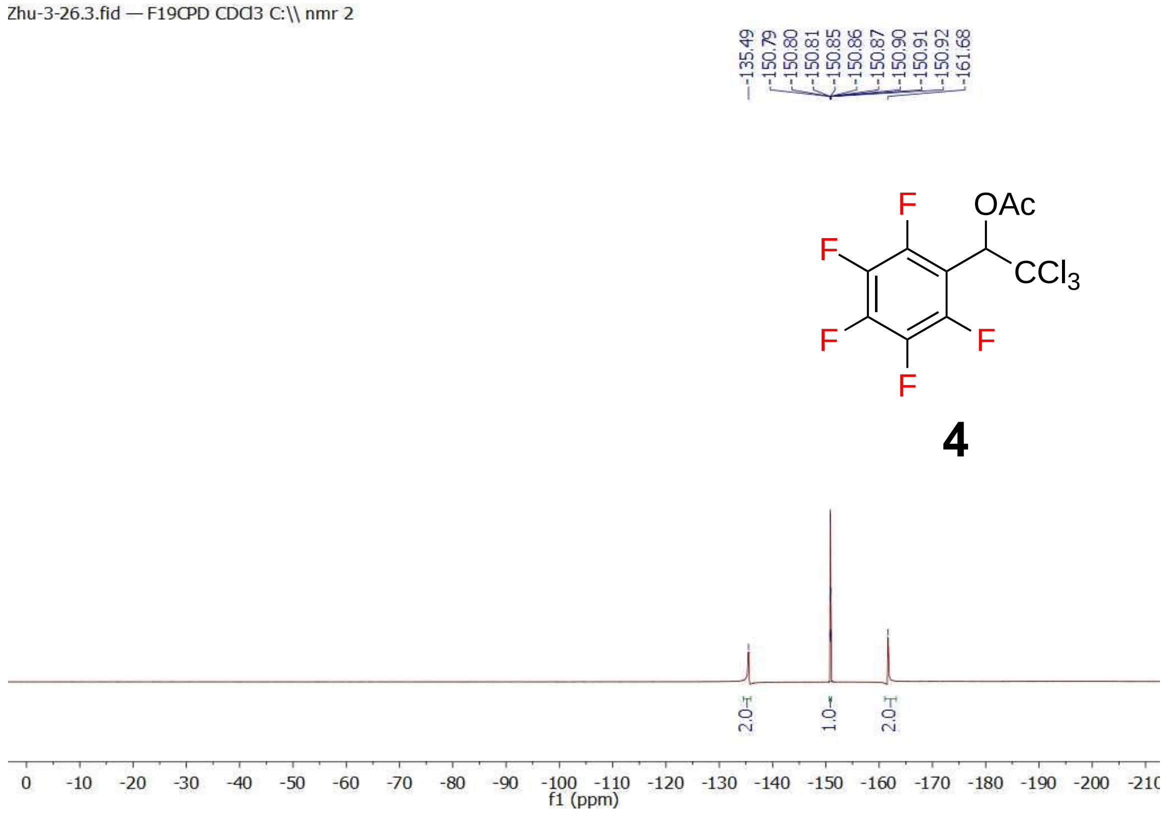
Zhu-3-26.3.fid — F19CPD CDCl3 C:\\ nmr 2
-135.49
-150.79
-150.80
-150.81
-150.85
-150.86
-150.87
-150.90
-150.91
-150.92
-161.68
F
OAc
F
CCl3
F
F
F
4
2.0
1.0
2.0
0 -10 -20 -30 -40 -50 -60 -70 -80 -90 -100 -110 -120 -130 -140 -150 -160 -170 -180 -190 -200 -210
f1 (ppm)

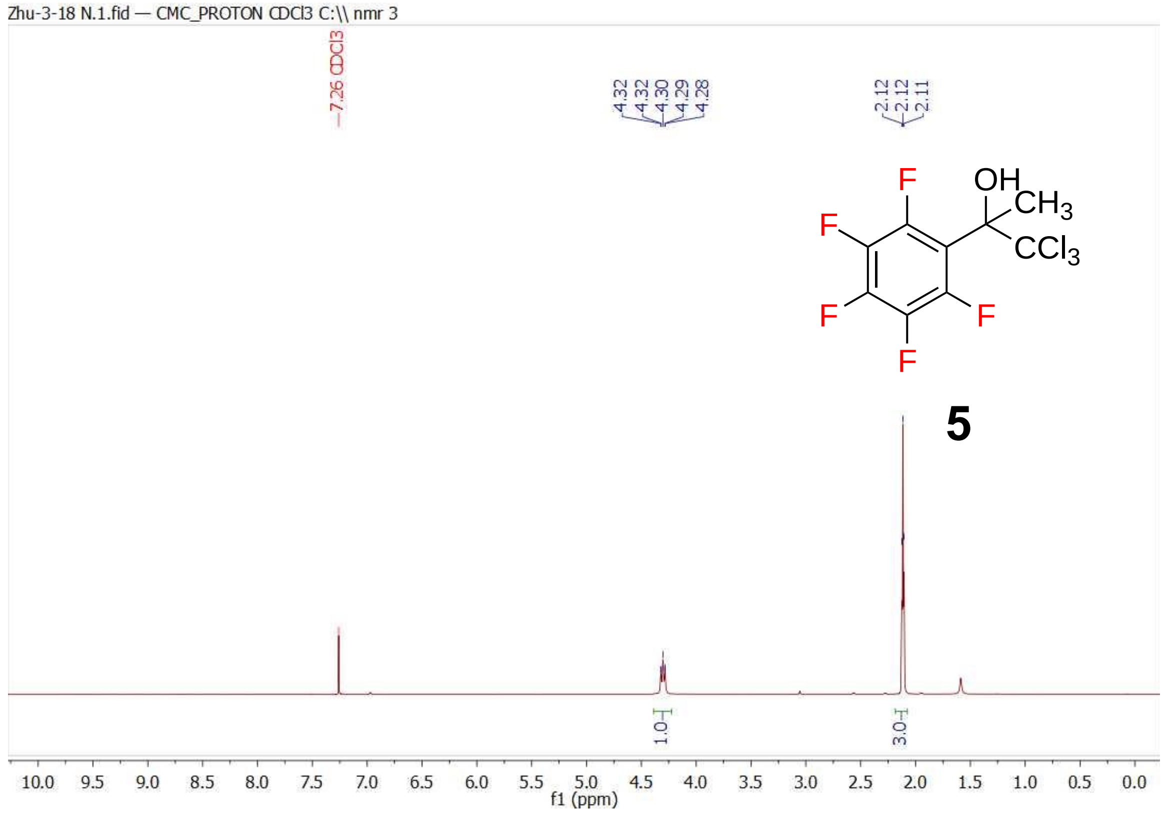
Zhu-3-18 N.1.fid — CMC_PROTON CDCl3 C:\\ nmr 3
7.26 CDCl3
4.32
4.32
4.30
4.29
4.28
2.12
2.12
2.11
F
OH
CH3
F
CCl3
F
F
F
5
1.0
3.0
10.0 9.5 9.0 8.5 8.0 7.5 7.0 6.5 6.0 5.5 5.0 4.5 4.0 3.5 3.0 2.5 2.0 1.5 1.0 0.5 0.0
f1 (ppm)

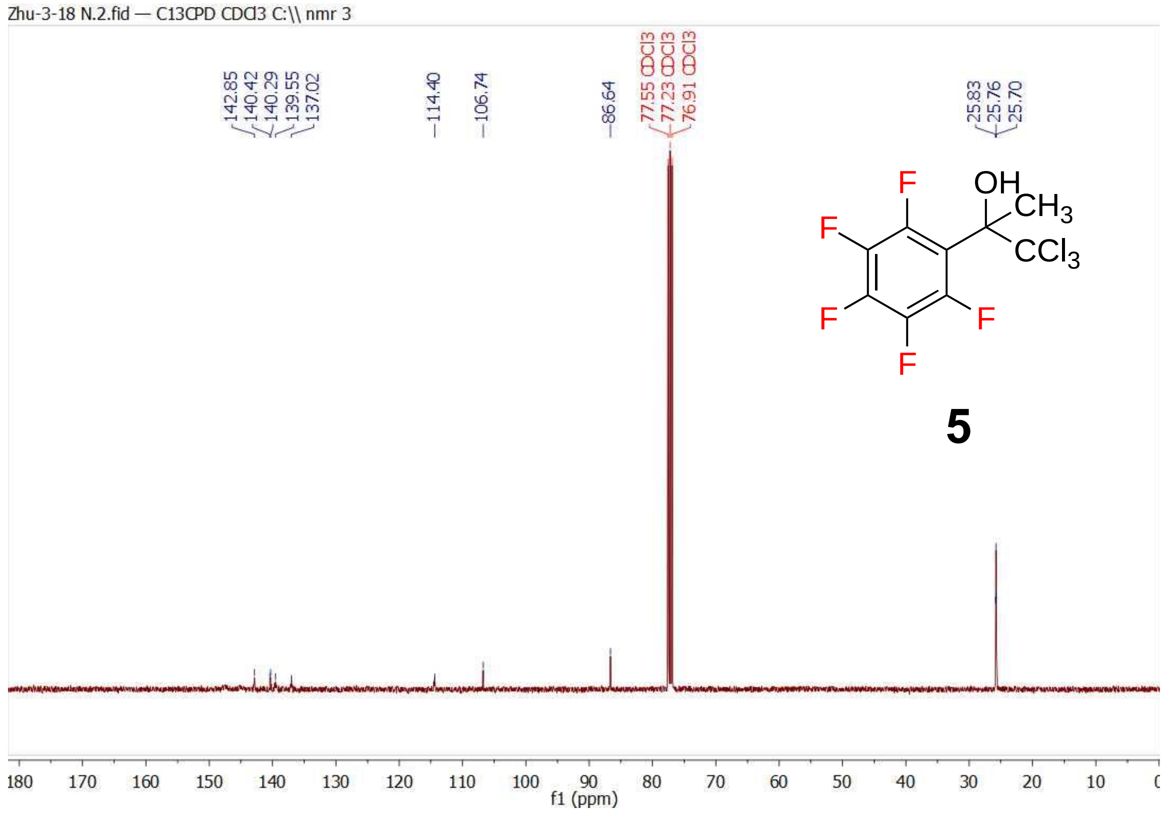
Zhu-3-18 N.2.fid — C13CPD CDCl3 C:\\ nmr 3
142.85
140.42
140.29
139.55
137.02
114.40
106.74
86.64
77.55 CDCl3
77.23 CDCl3
76.91 CDCl3
25.83
25.76
25.70
F
OH
CH3
CCl3
5
f1 (ppm)

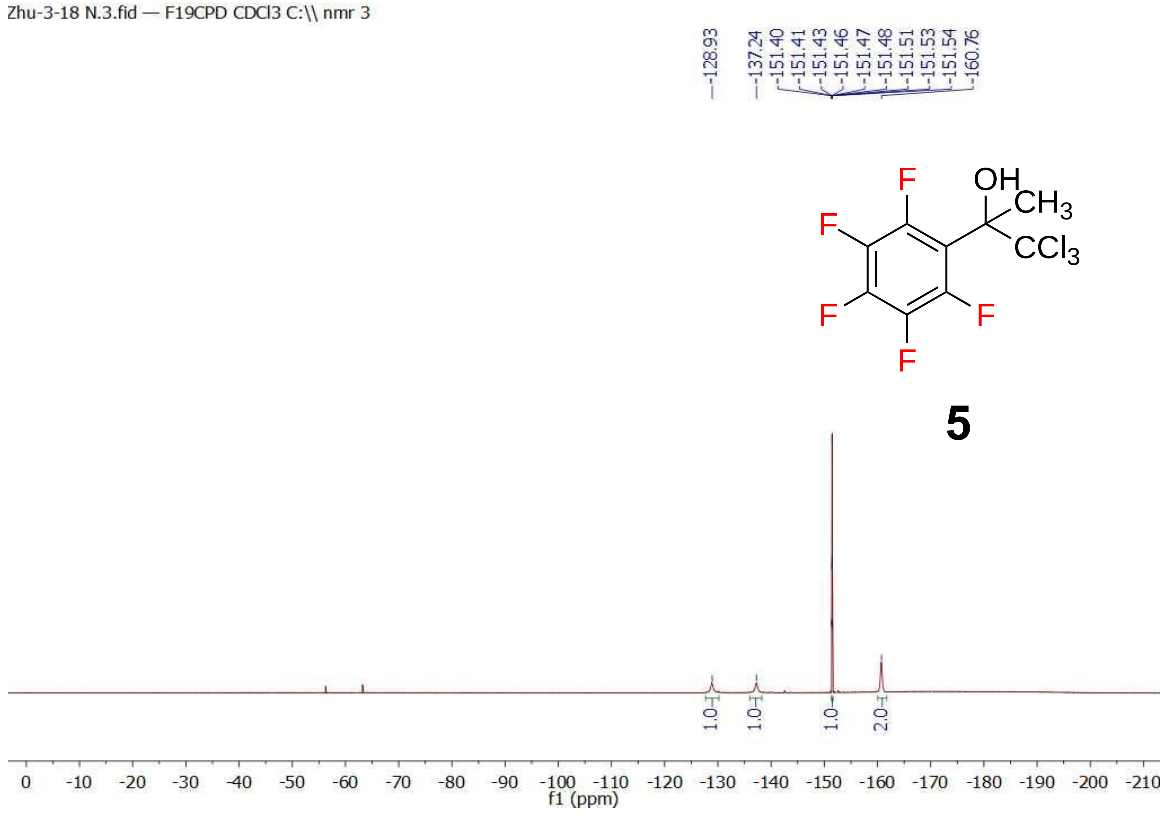
Zhu-3-18 N.3.fid — F19CPD CDCl3 C:\\ nmr 3
-128.93
-137.24
-151.40
-151.41
-151.43
-151.46
-151.47
-151.48
-151.51
-151.53
-151.54
-160.76
F
OH
CH3
CCl3
5
1.0
1.0
1.0
2.0
f1 (ppm)

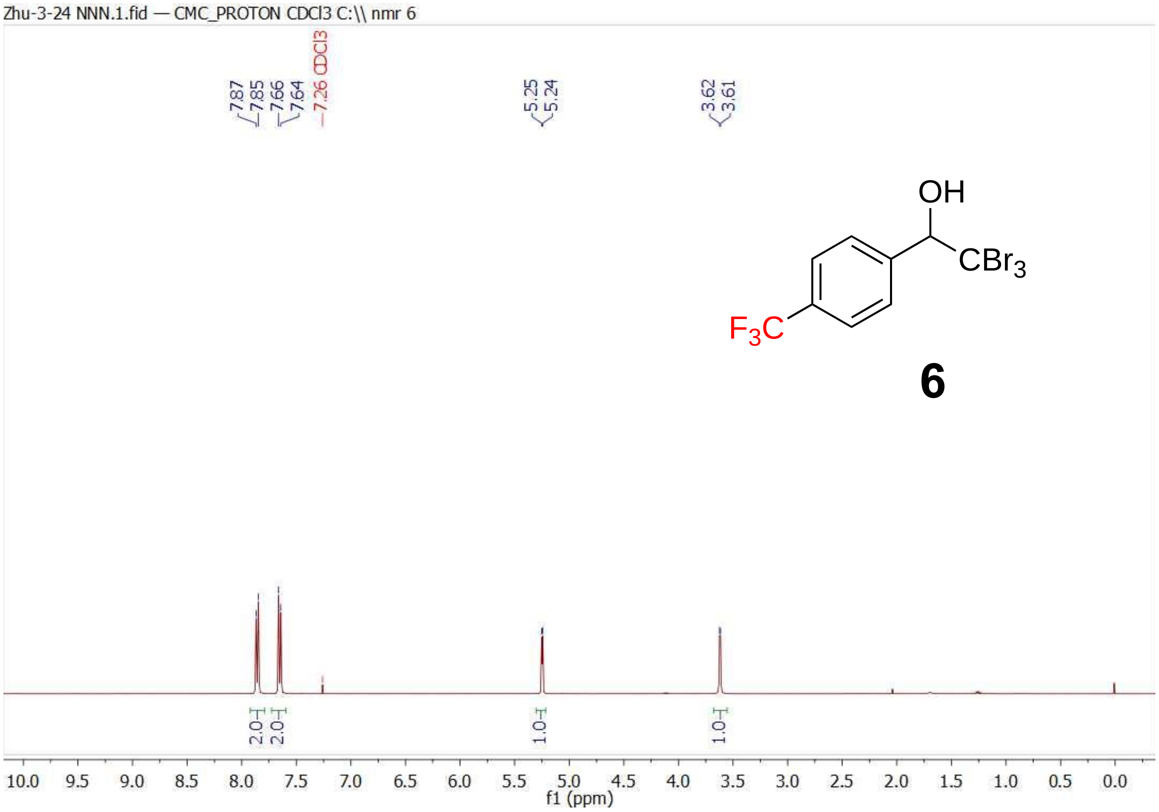
Zhu-3-24 NNN.1.fid — CMC_PROTON CDCl3 C:\\ nmr 6
7.87
7.85
7.66
7.64
7.26 CDCl3
5.25
5.24
3.62
3.61
OH
CBr3
F3C
6
2.0
2.0
1.0
1.0
10.0 9.5 9.0 8.5 8.0 7.5 7.0 6.5 6.0 5.5 5.0 4.5 4.0 3.5 3.0 2.5 2.0 1.5 1.0 0.5 0.0
f1 (ppm)

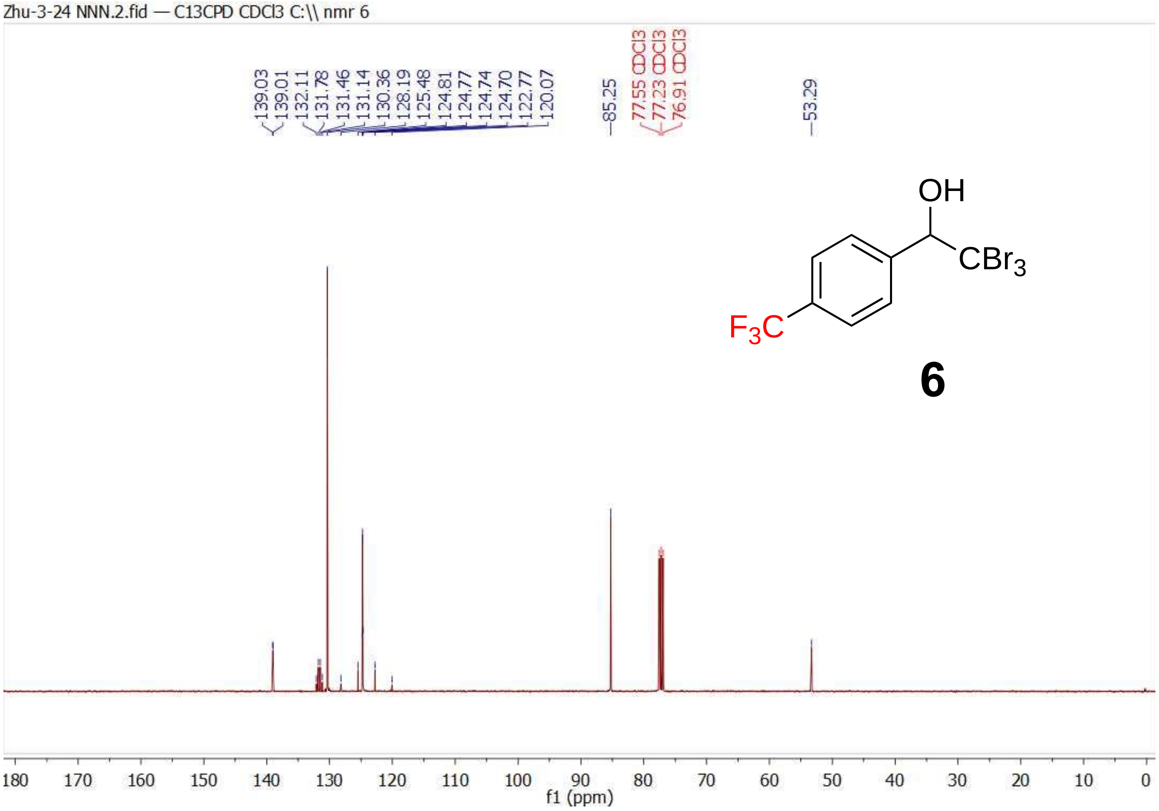
Zhu-3-24 NNN.2.fid — C13CPD CDCl3 C:\\ nmr 6
139.03
139.01
132.11
131.78
131.46
131.14
130.36
128.19
125.48
124.81
124.77
124.74
124.70
122.77
120.07
85.25
77.55 CDCl3
77.23 CDCl3
76.91 CDCl3
53.29
OH
CBr3
F3C
6
180 170 160 150 140 130 120 110 100 90 80 70 60 50 40 30 20 10 0
f1 (ppm)

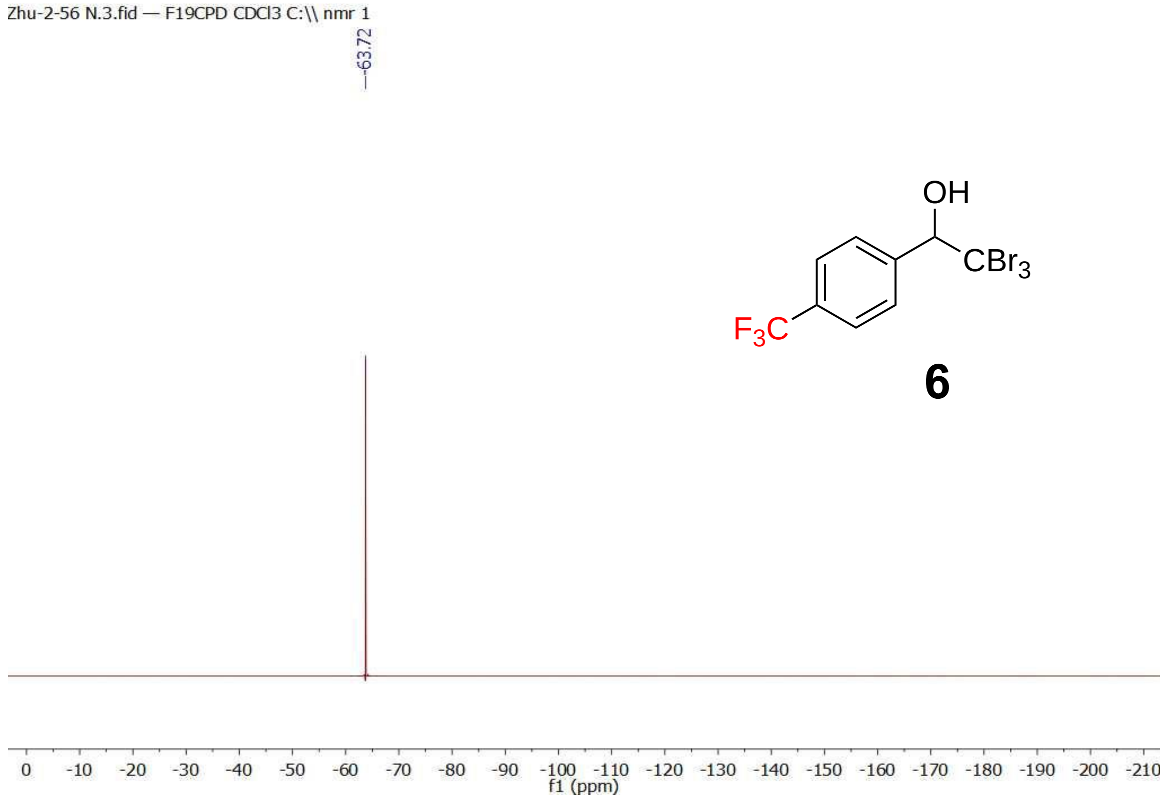

References

1. Perryman, M. S., Harris, M. E., Foster, J. L., Joshi, A., Clarkson, G. J., and Fox, D. J. (2013) Trichloromethyl ketones: asymmetric transfer hydrogenation and subsequent Jocic-type reactions with amines. *ChemComm 49*, 10022−10024.

2. Cantillana, T., Sundstrom, M., and Bergman, A. (2009) Synthesis of 2-(4- chlorophenyl)-2-(4-chloro-3-thiophenol)-1,1-dichloroethene (3-SH-DDE) via Newman−Kwart rearrangement−a precursor for synthesis of radiolabeled and unlabeled alkylsulfonyl-DDEs. *Chemosphere 76*, 805−810.

3. Linderman, R. J., and Graves, D. M. (1987) An efficient procedure for the oxidation of fluorinated carbinols. *Tetrahedron Lett. 28*, 4259–4262.

4. Lebel, H., Piras, H., and Bartholomeüs, J. (2014) Rhodium-catalyzed stereoselective amination of thioethers with N-mesyloxycarbamates: DMAP and bis(DMAP)CH_2Cl_2 as key additives. *Angew. Chem., Int. Ed. 53*, 7300–7304.

5. Ram, R. N., and Meher, N. K. (2003) Copper(I)-promoted dechlorinative Surzur−Tanner rearrangement of 2,2,2-trichloroethyl carboxylates. *Org. Lett. 5*, 145–147.

6. Morken, P. A., Bachand, P. C., Swenson, D. C., and Burton, D. J. (1993) Synthesis of fluorinated 1,2,3-butatrienes from a-halovinyl organometallic reagents. *J. Am. Chem. Soc. 115*, 5430–5439.
